# Copper-catalyzed remote C(*sp*^3^)–H azidation and oxidative trifluoromethylation of benzohydrazides

**DOI:** 10.1038/s41467-019-08741-w

**Published:** 2019-02-15

**Authors:** Xu Bao, Qian Wang, Jieping Zhu

**Affiliations:** Laboratory of Synthesis and Natural Products, Institute of Chemical Sciences and Engineering, Ecole Polytechnique Fédérale de Lausanne, EPFL-SB-ISIC-LSPN, BCH5304, CH-1015 Lausanne, Switzerland

## Abstract

The Hofmann-Löffler-Freytag (HLF) reaction is a prototypical example of radical-based remote functionalization of unactivated C(*sp*^3^)–H bond. While 1,5-hydrogen atom transfer (1,5-HAT) of the amidyl radical is thermodynamically favorable and is well-established, the method for the subsequent functionalization of the translocated carbon radical is still limited. We report herein two catalytic remote C(*sp*^3^)–H functionalization protocols. Cu(MeCN)_4_PF_6_-catalyzed reaction of 2-alkyl benzohydrazides **3** with TMSN_3_ in the presence of MeCO_2_O*t*Bu affords the γ-azido amides **4**, while CuCl-catalyzed reaction of **3** with Togni’s reagent provides 2-(β-trifluoromethylvinyl)benzamides **5** via an oxidative δ-trifluoromethylation of the alkyl group. Mechanistic studies suggest that the γ-azidation of benzohydrazides **3** goes through 1,5-HAT followed by a Cu-mediated azido transfer cascade, while the oxidative δ-trifluoromethylation of **3** proceeds via, after 1,5-HAT process, a radical-polar crossover mechanism.

## Abstract (web summary)

Functionalization of carbon radicals formed via 1,5-hydrogen atom transfer of amidyl radicals is an underdeveloped protocol for remote C–H functionalization. Here, the authors report a Cu-catalyzed γ-azidation and oxidative δ-trifluoromethylation of benzohydrazides for the synthesis of more functionalized amides.

## Introduction

The Hofmann–Löffler–Freytag (HLF) reaction^1,2^ that converts the *N*-haloamines to pyrrolidines constitutes a prototypical example of radical-based remote C(*sp*^3^)–H bond functionalization process^3^. The reaction, discovered in 1883, is a complex domino process involving the generation of the aminium radical followed by regioselective 1,5-hydrogen atom transfer (HAT), halogenation, and cyclization^4–8^. The instability of the *N*-haloamines, as well as the generally harsh conditions required for the generation and subsequent hydrogen atom abstraction of aminium radicals, limited nevertheless the full exploitation of its synthetic potential. A major breakthrough addressing this issue came from Suárez’s group. In a series of seminal papers, they reported that amides/sulfonamides can be converted to the corresponding amidyl and sulfonamidyl radicals with iodine/lead tetraacetate^9,10^ or iodine/phenyliodine diacetate^11^ as oxidants, and these electron-withdrawing group-attached nitrogen-centered radicals readily undergo intramolecular HAT under mild neutral conditions. Recently, Müniz et al.^12–15^ showed that the amidyl radical can be generated directly from the secondary amide under mild catalytic oxidative conditions, while the group of Knowles^16^ and Rovis^17,18^ demonstrated independently that photoredox conditions were very effective for the same purpose. Complementary to this work, significant efforts have also been made recently in identifying tailored precursors for the generation of the *N*-centered radicals^19–25^. The development of these easily available and stable amidyl radical precursors in conjunction with the recent advent of metal and visible-light photoredox catalysts allowed the execution of the HLF reaction under much milder conditions, therefore providing opportunities to expand the repertoire of the remote C(*sp*^3^)–H functionalization process. Indeed, besides the cyclization to *N*-heterocycles^9–11,26–32^, halogenation^22,23,33–36^ and C–S bond formation^21^ that are inherent to the classic radical chain mechanism of the HLF reaction, C–C bond formation via radical addition^16–18,37–39^, azidation/cyanation^19,20,22^, acetoxylation^40^, thiolation/alkynylation^20,22^, and arylation^24,25^ have been developed very recently.

**Fig. 1:**
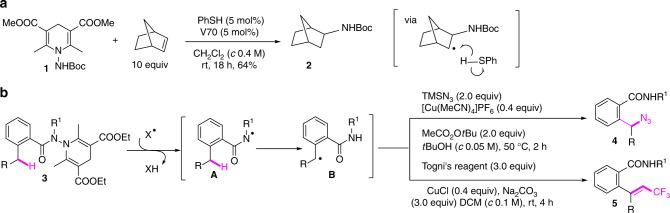
Amidyl radicals and HLF reaction. **a** Studer’s work on the use of *N*-aminated dihydropyridines as precursors of *N*-centered radicals: hydroamination of alkenes; **b** benzohydrazides in HLF reaction: functionalization of remote C(*sp*^3^)–H bond. V70 = 2,2’-azobis(2,4-dimethyl-4-methoxy valeronitrile); TMSN_3_ = trimethylsilyl azide; MeCO_2_O*t*Bu = *tert*-butyl peroxyacetate

Studer et al. demonstrated that amidyl radicals can be generated from *N*-aminated dihydropyridines **1** and be used in the hydroamination of olefins (Fig. 1a)^41,42^. Mechanistically, the process is initiated by the polarity-matched abstraction of the Hantzsch ester’s C-4 hydrogen by the electrophilic thiophenol radical to afford, after fragmentation, the amidyl radical and pyridine. The addition of the former to the double bond followed by hydrogen transfer from thiophenol to the resulting radical adduct afforded the product **2** with concurrent regeneration of the thiophenol radical to propagate the chain. Inspired by these results and in connection with our interest in remote C(*sp*^3^)–H functionalization processes^24,43,44^, we hypothesized that if the amidyl radical **A** was generated by action of an electrophilic radical species (X•) whose reduced form X–H was reluctant to transfer its hydrogen to the relayed *C*-centered radical **B**, then it would be possible to functionalize **B** by other reactive species to afford remote functionalized amides. We assumed that electrophilic trifluoromethyl and *tert*-butoxy radicals could satisfy these criteria as CF_3_–H and *t*BuO–H would not be able to propagate the chain reaction due to their high bond dissociation energies (CF_3_–H: 107.4 kcal/mol, *t*BuO–H: 106.3 kcal/mol). We report herein the Cu-catalyzed γ-azidation and oxidative δ-trifluoromethylation of benzohydrazides **3** for the synthesis of amides **4** and **5**, respectively (Fig. 1b). Conversion of ethyl to trifluoromethylvinyl group is, to the best of our knowledge, unknown.

## Results

### Cu-catalyzed γ-C(*sp*^3^)–H azidation of benzohydrazides

**Fig. 2:**
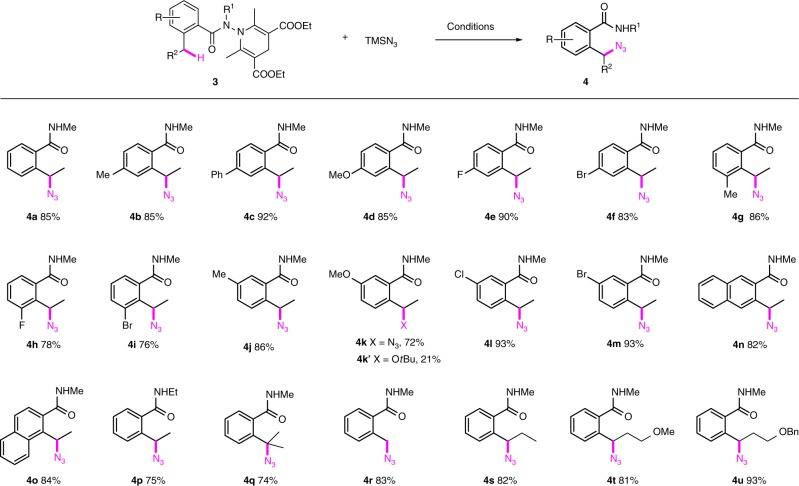
γ-Azidation of 2-alkyl benzohydrazides. **3** (0.1 mmol), TMSN_3_ (0.2 mmol), Cu(MeCN)_4_PF_6_ (0.04 mmol), MeCO_2_O*t*Bu (0.2 mmol), *t*BuOH (2.0 mL, *c* 0.05 M), 50 °C, 2 h

While azidation of alkenes are well developed^45–48^, direct azidation of C(*sp*^3^)–H bond remains challenging^49^. Indeed, the γ-azidation of amides was unknown at the outset of this work. However, Studer et al. have very recently reported such a transformation using TfN_3_ as an azide donor^19^. We focused on the use of stable and readily available TMSN_3_ as an azide source and the benzohydrazides **3**^41,42^ as precursors of amidyl radicals. In line with our working hypothesis and knowing that peroxide is compatible with the Cu-catalyzed carboazidation process^50,51^, the reaction of **3a** (R^2^ = Me, R = R^1^ = H) with TMSN_3_ was investigated in the presence of copper salts and peroxides. After systematic screening of the copper sources [CuCl, CuBr, CuI, CuOTf·benzene, Cu(MeCN)_4_PF_6_, Cu(OAc)_2_, Cu(OTf)_2_, CuSO_4_, Cu(acac)_2_, Cu(ClO_4_)_2_], the peroxides (*t*BuOOH, *t*BuOO*t*Bu, cumene hydroperoxide, PhCO_2_O*t*Bu, MeCO_2_O*t*Bu), and the solvents (*t*BuOH, DCE, 1,4-dioxane, MeCN, CF_3_CH_2_OH), the optimum conditions found consisted of heating a *t*BuOH solution of **3a** (*c* 0.05 M) and TMSN_3_ (2.0 equiv) at 50 °C in the presence of Cu(MeCN)_4_PF_6_ (0.4 equiv) and MeCO_2_O*t*Bu (2.0 equiv, 50 wt. % in odorless mineral spirits from Sigma). Under these conditions, the azido amide **4a** was isolated in 85% yield (Fig. 2). A small amount of 2-(1-(*tert*-butoxy)ethyl)-*N*-methylbenzamide (see Supplementary Information) was also isolated from the reaction mixture.

The scope of this azidation protocol was next examined (Fig. 2). A range of 2-alkyl substituted benzohydrazides were converted to the corresponding 2-(1-azidoalkyl)benzamides in good-to-excellent yields. Halides (F, Cl, Br) at different position of the aromatic ring was compatible with the reaction conditions affording the azido derivatives suitable for further functionalization. The primary, secondary, and tertiary benzylic carbons were all successfully functionalized to afford the corresponding azides in good to excellent yields. In the case of **3k** (R = 4-MeO), the desired azido compound **4k** (72%) was isolated together with a significant amount of **4k’** (X = O*t*Bu, 21%) probably due to the presence of a strong electron-donating *para*-methoxy group.

**Fig. 3:**
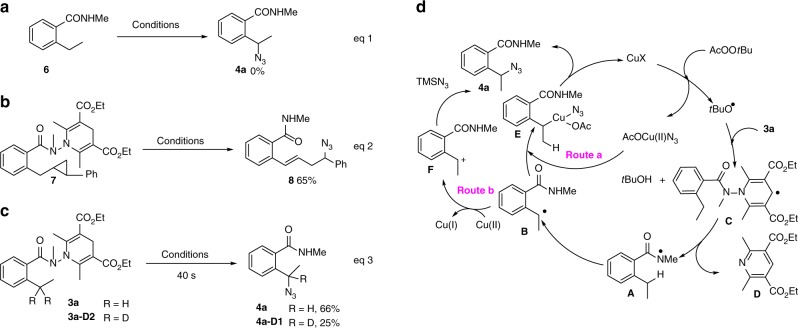
Mechanistic studies on the γ-azidation of 2-alkyl benzohydrazides. **a** Control experiment; **b** radical clock experiment; **c** KIE experiment; **d** possible reaction pathway; conditions: **6**, **7** or **3a/3a-D2** (0.1 mmol), TMSN_3_ (0.2 mmol), Cu(MeCN)_4_PF_6_ (0.04 mmol), MeCO_2_O*t*Bu (0.2 mmol), *t*BuOH (2.0 mL, *c* 0.05 M), 50 °C, 2 h

A series of control experiments were carried out to gain insight on the reaction mechanism. Treatment of *N*-methyl-2-ethylbenzamide (**6**) under standard conditions failed to produce even a trace amount of azido amide **4a** indicating that **6** was not an intermediate of the reaction (Fig. 3, **a**). In line with the radical mechanism, azidation of **3a** was completely inhibited in the presence of TEMPO or BHT, and a radical clock experiment involving cyclopropane-containing substrate **7** afforded azide **8** in 65% yield (Fig. 3, **b**). A side-by-side kinetic experiment using **3a** and **3a-D2** provided an intermolecular KIE value of 2.6 (Fig. 3, **c**) suggesting that the 1,5-HAT, an off-catalytic cycle process, might be a rate-limiting step in the present domino azidation process.

On the basis of these results, a possible reaction pathway for the γ-azidation of benzohydrazide **3a** is proposed (Fig. 3, **d**). Reduction of peroxide by Cu(I)X salt would produce AcOCuX salt and *tert*-butoxy radical, which would abstract the C-4 hydrogen from dihydropyridine to give *t*BuOH and dihydropyridine radical **C**. Fragmentation of the latter would lead to the amidyl radical **A** and pyridine **D**. Subsequent 1,5-HAT of **A** would generate the benzyl radical **B** which, upon radical rebound with AcOCu(II)N_3_ (Fig. 3, **d**, route a), would afford Cu(III) species **E**. Reductive elimination would then deliver the γ-azido amide **4a** with the concurrent release of Cu(I) salt. The formation of 2-(1-(*tert*-butoxy)ethyl)-*N*-methylbenzamide could be accounted for by the presence of minor amount of *t*BuOCuX species in the reaction mixture^52^.

An alternative pathway would involve the oxidation of radical **B** to benzyl cation **F** that could then be trapped by TMSN_3_ to provide **4a** (Fig. 3, **d**, route b). To verify this possibility, the reaction of **3o** (R = 5-OMe, *cf* Fig. 2) with TMSN_3_ was carried out in a mixture of solvent (*t*BuOH/MeOH = 1:1) under otherwise identical conditions. Azide **4o** was still isolated in 65% yield together with a small amount of 2-(1-(*tert*-butoxy)ethyl)-*N*-methylbenzamide (6%). Methoxylated product was not observed. This result together with the fact that isoindolinone^53–55^ was not formed under our conditions are in accord with the Cu-mediated redox azido transfer mechanism^50,51^.

### Cu-catalyzed oxidative δ-C(*sp*^3^)-trifluoromethylation

**Fig. 4:**
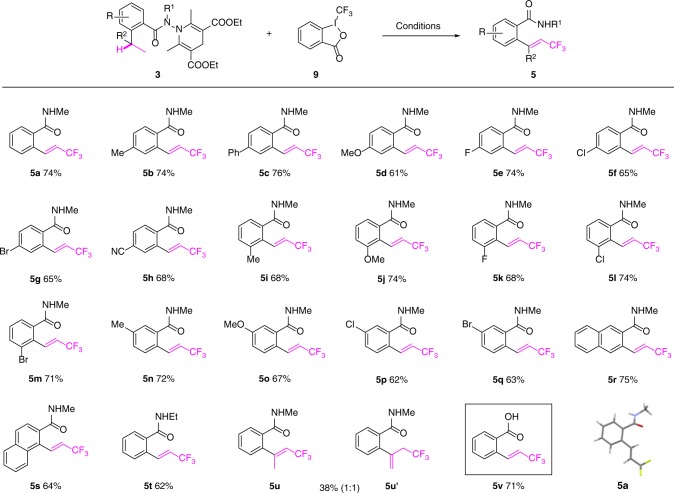
Oxidative δ-trifluoromethylation of 2-alkyl benzohydrazides.** 3** (0.1 mmol), Togni’s reagent **9** (0.3 mmol), CuCl (0.04 mmol), DCM (1.0 mL), Na_2_CO_3_ (0.3 mmol), rt, 4 h

Trifluoromethylation of 2-alkyl benzohydrazides was next examined. The reaction of **3a** with Togni’s reagent **9**^46–48^ in the presence of metal salts was chosen as a benchmark reaction. Instead of obtaining the *N*-methyl-2-(1,1,1-trifluoropropan-2-yl)benzamide, our initial experiments allowed us to isolate unexpectedly the compound **5a** whose structure was confirmed by X-ray crystallographic analysis (Fig. 4). Since the reaction converting formally an ethyl group to a trifluoromethylvinyl group was unknown^56^, conditions were further optimized toward its formation by varying the catalysts (Cu(I), Cu(II), Fe(II), Fe(III) salts), the solvents (DCM, DCE, toluene, 1,4-dioxane), and the additives (NaHCO_3_, Na_2_CO_3_, K_2_CO_3_, NaH_2_PO_4_, etc.) (see Supplementary Information). The optimum conditions found consisted of stirring a solution of **3a** and Togni’s reagent **9** (3.0 equiv.) in DCM (*c* 0.10 M) in the presence of CuCl (0.4 equiv.) and Na_2_CO_3_ (3.0 equiv.) at room temperature. Under these conditions, **5a** was isolated in 74% yield.

The generality of this oxidative γ-trifluoromethylation of 2-ethyl benzohydrazides was next examined (Fig. 4). The presence of the electron-donating (Me, OMe) and -withdrawing groups (CN) and halides (F, Cl, Br, I) at different positions of the aromatic ring were well tolerated to afford the corresponding 2-(β-trifluoromethylvinyl)benzamides **(5a–5q)** in good yields. In the case of **3** **m** (R = 3-Br), the corresponding isoindolinone resulting from the intramolecular C–N bond formation was isolated in 5% yield. *N*-methyl 3-ethyl and *N*-methyl 1-ethyl-2-naphthohydrazides were similarly transformed to the corresponding β-trifluoromethylvinyl substituted naphthoamides **5r** and **5** **s**. The *N*,2-diethyl benzohydrazide was chemoselectively converted to *N*-ethyl-2-(β-trifluoromethylvinyl)benzamide **5t** without event. Finally, 2-isopropyl benzohydrazide was converted to a mixture of two isomers **5** **u**/**5** **u’** (1:1) in a moderate yield. The methyl amide **5a** was readily hydrolyzed to the corresponding carboxylic acid **5** **v** (HOAc–H_2_O, 20% H_2_SO_4_), providing therefore a versatile functional group for further functionalization.

**Fig. 5:**
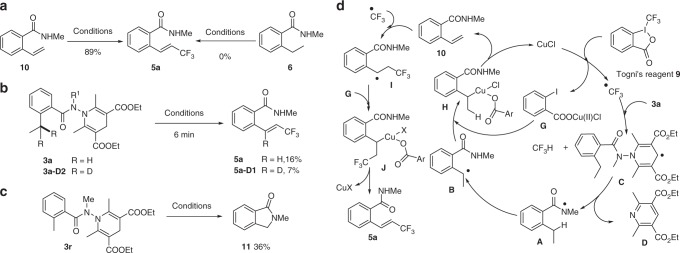
Mechanistic studies on the oxidative δ-trifluoromethylation of 2-alkyl benzohydrazides. **a** Control experiment; **b** KIE experiment; **c** isoindolinone formation; **d** possible reaction pathway; conditions: **10** or **6** or **3a/3a-D2** (0.1 mmol), Togni’s reagent **9** (0.3 mmol), CuCl (0.04 mmol), DCM (1.0 mL, *c* 0.1 M), Na_2_CO_3_ (0.3 mmol), rt, 4 h

Addition of TEMPO to the reaction of **3a** with **9** inhibited completely the process and only 2,2,6,6-tetramethyl-1-(trifluoromethoxy)piperidine was formed. Reducing the amount of Togni’s reagent **9** to 1.5 equiv. under otherwise identical conditions decreased the yield of **5a** (42% yield) with concurrent formation of *N*-methyl-2-vinyl benzamide (**10**) and *N*-methyl-2-ethylbenzamide (**6**) in yields of 12 and 4%, respectively (Fig. 5, **a**). Resubmitting **10** to the standard conditions afforded **5a** in 89% yield^57^, while **6** remained unchanged under standard conditions. A side-by-side experiment using **3a** and **3a-D2** provided an intermolecular KIE (k_H_/k_D_) value of 2.3 indicating that the 1,5-HAT step could be a rate-limiting step in our domino process (Fig. 5, **b**). Finally, treatment of **3r** under standard conditions furnished **11** in 36% yield. This result indicated that the 1,5-HAT of the in situ generated amidyl radical occurred. However, in contrast to the azidation process, the transfer of CF_3_ to the resulting translocated benzyl radical was not fast enough to compete with the intramolecular C–N bond formation (Fig. 5, **c**).

On the basis of the results of these control experiments, a possible reaction pathway is proposed (Fig. 5, **d**). Single-electron transfer between Togni’s reagent **9** and CuCl would produce the CF_3_ radical and Cu(II) salt **G**. The abstraction of the C-4 hydrogen of the Hantzsch ester **3a** by CF_3_• generated radical **C** which underwent fragmentation to provide the amidyl radical **A** and pyridine **D**. The 1,5-HAT of **A** furnished the then translocated benzyl radical **B**, which upon radical rebound with Cu(II) salt **G**, would afford **H**. Reductive elimination leading to the formation of C–Cl and C–OCOAr bonds may not be kinetic competent, the alternative β-hydride elimination took place to afford 2-vinyl benzamide (**10**). Subsequent addition of CF_3_ radical to the newly generated double bond would generate the benzyl radical **I** which, upon radical rebound and β-hydride elimination, would afford the observed product **5a** with concurrent generation of the Cu(I) species. Following this reaction pathway, at least two equivalents of Togni’s reagent **9** are needed to complete this domino process, which is in accordance with the result of our control experiment.

**Fig. 6:**
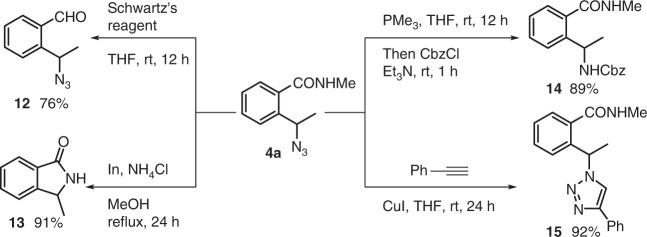
Transformation of azido amide **4a**. Schwartz’s reagent = bis(cyclopentadienyl)zirconium(IV) chloride hydride, CbzCl = benzyl chloroformate

Post-transformations of azido amide **4a** were carried out to illustrate its synthetic potential (Fig. 6). Chemoselective reduction of the amide function in **4a** with Schwartz’s reagent delivered azido aldehyde **12** in 76% yield. Heating to reflux a MeOH solution of **4a** with indium in the presence of NH_4_Cl afforded the isoindolinone **13** in 91% yield via probably a reduction/transamidation sequence. On the other hand, Staudinger reduction of azide followed by in situ acylation with CbzCl furnished carbamate **14**, while the click reaction of **4a** with phenylacetylene provided the triazole **15** in 92% yield.

## Discussion

The classic Hofmann–Löffler–Freytag (HLF) reaction converts the protonated *N*-chloroamines to pyrrolidines under thermal or photochemical conditions. In this transformation, the carbon-centered radical, resulting from the 1,5-HAT of the in situ generated aminium radical, abstracts a chlorine atom from the ammonium salt forming therefore a radical chain process. While many different transformations could in principle be envisaged for the functionalization of the translocated C-centered radical, only very few reaction types have actually been successfully combined with the 1,5-HAT process.

In this paper, we presented two copper-catalyzed remote C(*sp*^3^)–H functionalization processes in which the metal-catalyzed cross-coupling reactions were successfully merged with the radical 1,5-HAT process. The salient feature of these transformations was that the copper salts served not only as a reductant to generate the amidyl radicals from benzohydrazides **3**, but also catalyzed the functionalization of the translocated carbon radical. Specifically, Cu(MeCN)_4_PF_6_-catalyzed reaction of 2-alkyl benzohydrazides **3** with TMSN_3_ in the presence of MeCO_2_O*t*Bu affords the γ-azido amides **4**, while CuCl-catalyzed reaction of **3** with Togni’s reagent provides 2-(β-trifluoromethylvinyl)benzamides **5** via an oxidative δ-trifluoromethylation of the alkyl group. Mechanistic studies suggest that the γ-azidation of benzohydrazides **3** goes through 1,5-HAT followed by a Cu-mediated azido transfer cascade, while the oxidative δ-trifluoromethylation of **3** proceeds via, after 1,5-HAT process, a radical-polar crossover mechanism.

In view of the availability and reliability of a wide range of metal-catalyzed cross-coupling reactions, we believe that their combination with the 1,5-HAT process will open a new avenue for the development of powerful synthetic tools^58^.

## Methods

### Cu-catalyzed γ-C(*sp*^3^)–H azidation of benzohydrazides

A screw cap tube was charged with Cu(CH_3_CN)_4_PF_6_ (14.9 mg, 0.04 mmol, 0.4 equiv.), substrate **3** (0.1 mmol, 1.0 equiv.), TMSN_3_ (26.5 μL, 0.2 mmol, 2.0 equiv.), and *t*BuOH (2.0 mL). The mixture was stirred at 50 °C for 2 min, then MeCO_2_O*t*Bu (32.2 μL, 0.2 mmol, 2.0 equiv.) was added to the above mixture. After being stirred for 2 h at 50 °C under N_2_ atmosphere, the reaction mixture was quenched with water, extracted with EtOAc. The organic extracts were washed with brine, dried over Na_2_SO_4_. The solvent was removed under reduced pressure. The residue was purified by flash column chromatography (SiO_2_, eluent: petroleum ether/EtOAc = 2/1) to give **4**.

### Cu-catalyzed oxidative δ-C(*sp*^3^)–H trifluoromethylation

To a screw cap tube charged with CuCl (4.0 mg, 0.04 mmol, 0.4 equiv.) and Na_2_CO_3_ (31.8 mg, 0.3 mmol, 3.0 equiv.) was added a solution of Togni’s reagent **9** (94.8 mg, 0.3 mmol, 3.0 equiv.) in DCM (0.5 mL) under argon atmosphere. After stirring for 2 min, substrate **3** (0.1 mmol, 1.0 equiv.) in DCM (0.5 mL) was added to the above mixture. After being stirred for 4 h at room temperature under argon atmosphere, the reaction mixture was quenched with water, extracted with EtOAc. The organic extracts were washed with Na_2_CO_3_ solution and brine, dried over Na_2_SO_4_. The solvent was removed under reduced pressure. The residue was purified by flash column chromatography (SiO_2_, eluent: petroleum ether/EtOAc = 2/1) to give **5**.

## Supplementary information

Supplementary Information

## Data Availability

The authors declare that the data supporting the findings of this study are available within the paper and the Supplementary Information, as well as from the authors upon request. The X-ray crystallographic coordinates for structures reported in this study have been deposited at the Cambridge Crystallographic Data Centre (CCDC), under deposition numbers 1866223. These data can be obtained free of charge from The Cambridge Crystallographic Data Centre via https://www.ccdc.cam.ac.uk/structures/Search?Ccdc=1866223&Author=Bao%20Xu&Access = referee
