# Supplementary material for: Copper-catalyzed remote C(sp3)–H azidation and oxidative trifluoromethylation of benzohydrazides
Source: Nat Commun. 2019 Feb 15;10:769. doi: 10.1038/s41467-019-08741-w (PMC6377619; doi:10.1038/s41467-019-08741-w)
Supplement: Supplementary file 1 — Supplementary Information [file 41467_2019_8741_MOESM1_ESM.pdf]

Supplementary Information

Copper-catalyzed remote C(sp^3)-H azidation and oxidative trifluoromethylation of benzohydrazides

Bao, X., *et al*

Supplementary Figures

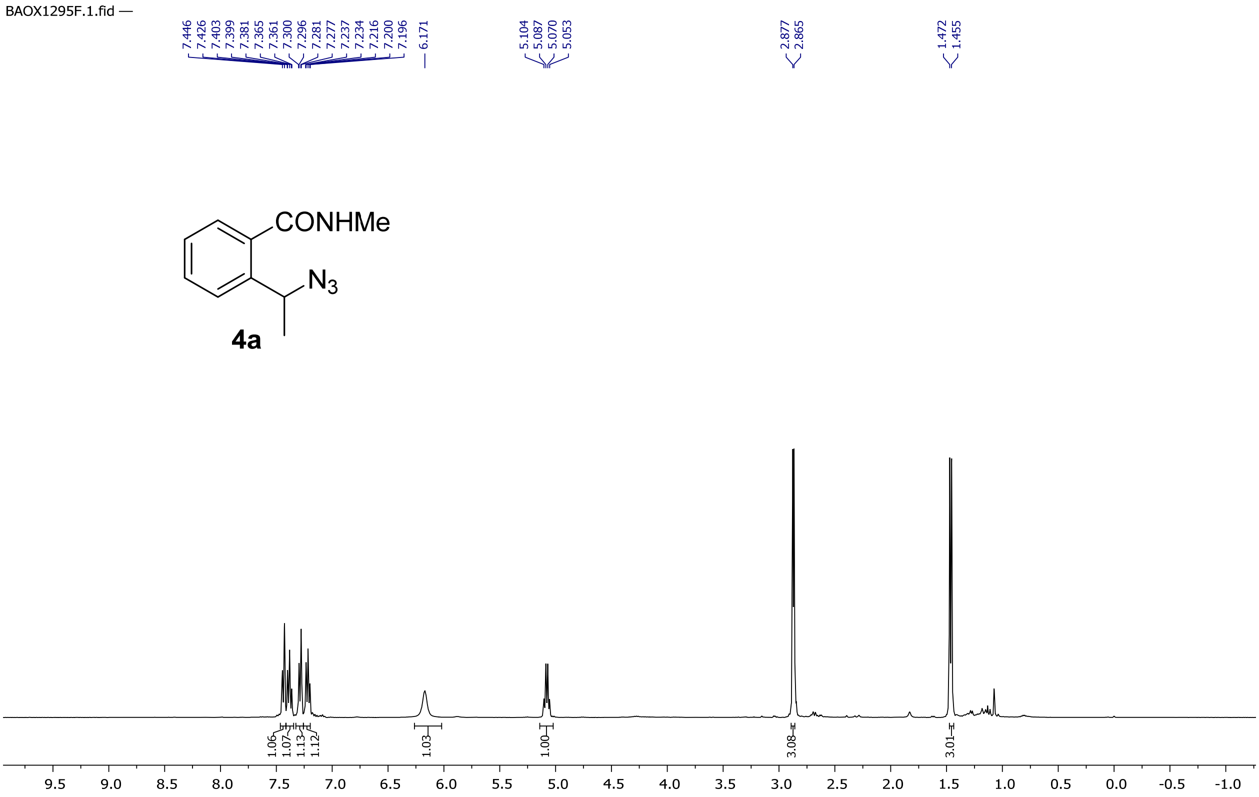

Supplementary Figure 1. ^{1}H spectra of 4a

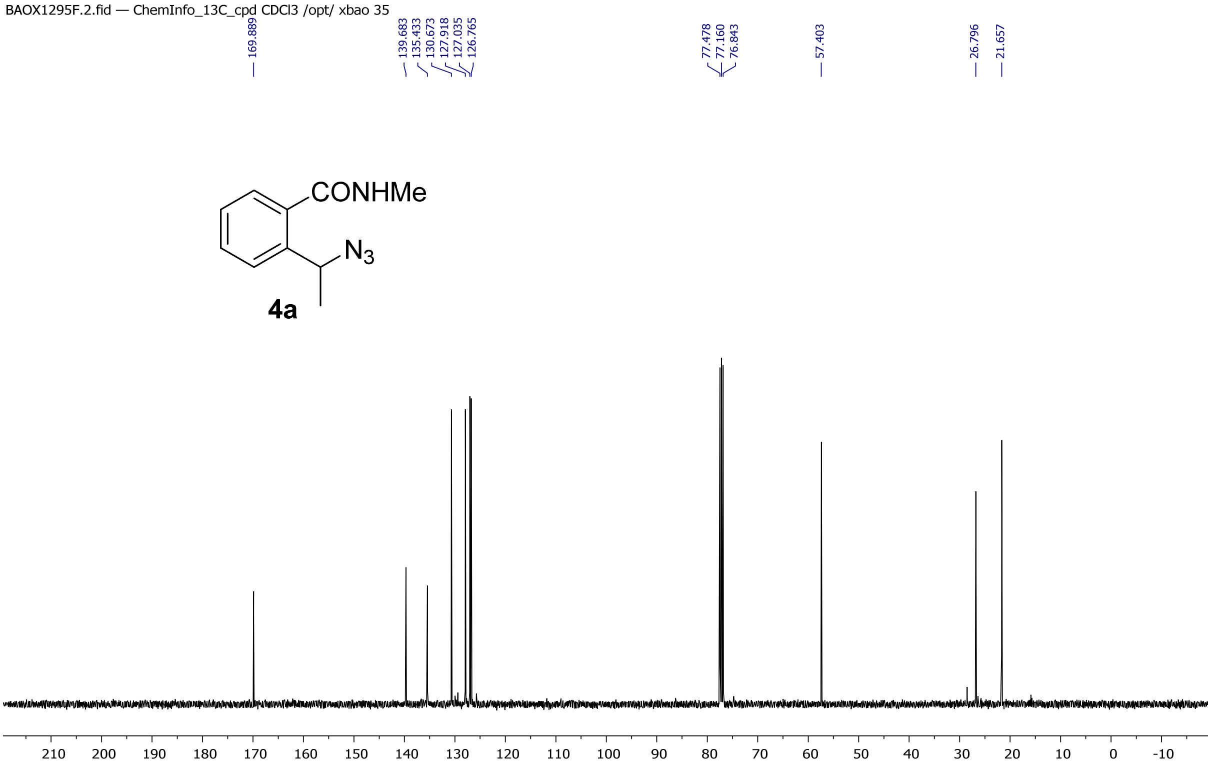

Supplementary Figure 2. ^{13}C NMR spectra of 4a

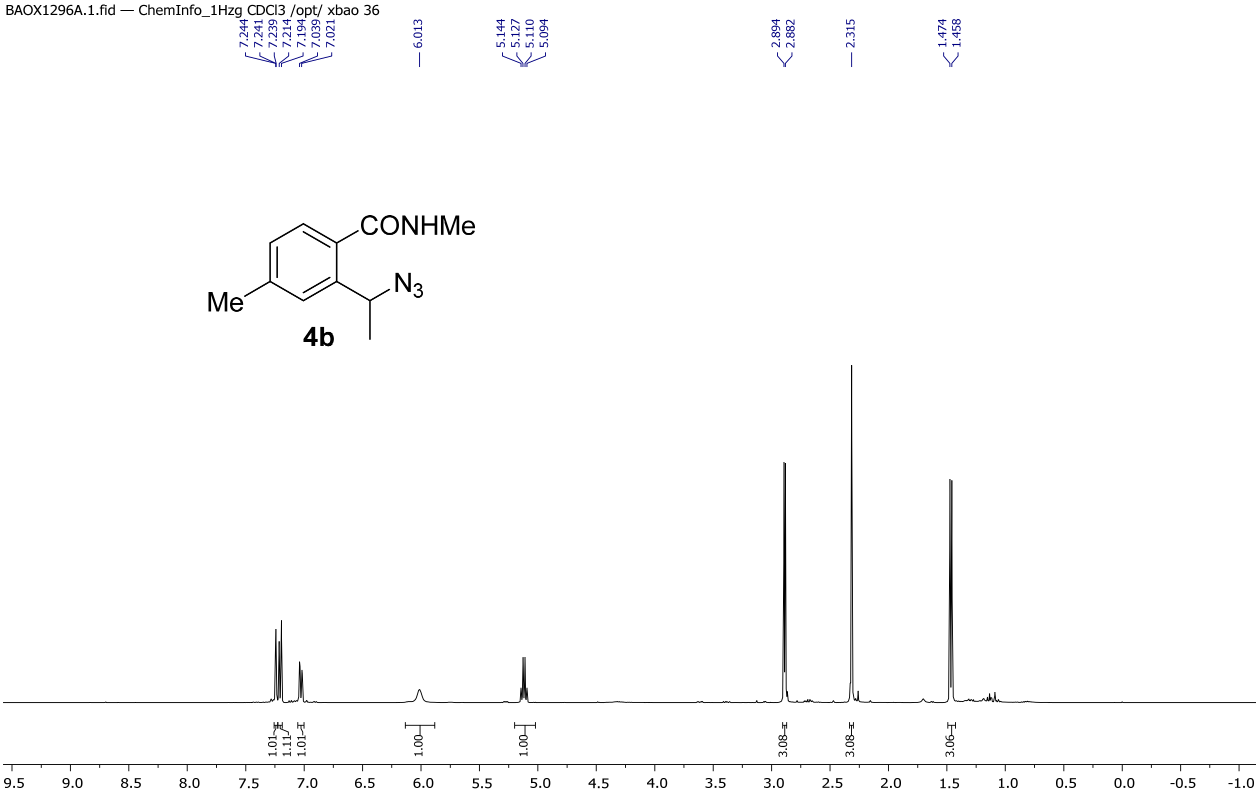

Supplementary Figure 3. ^{13}C NMR spectra of 4b

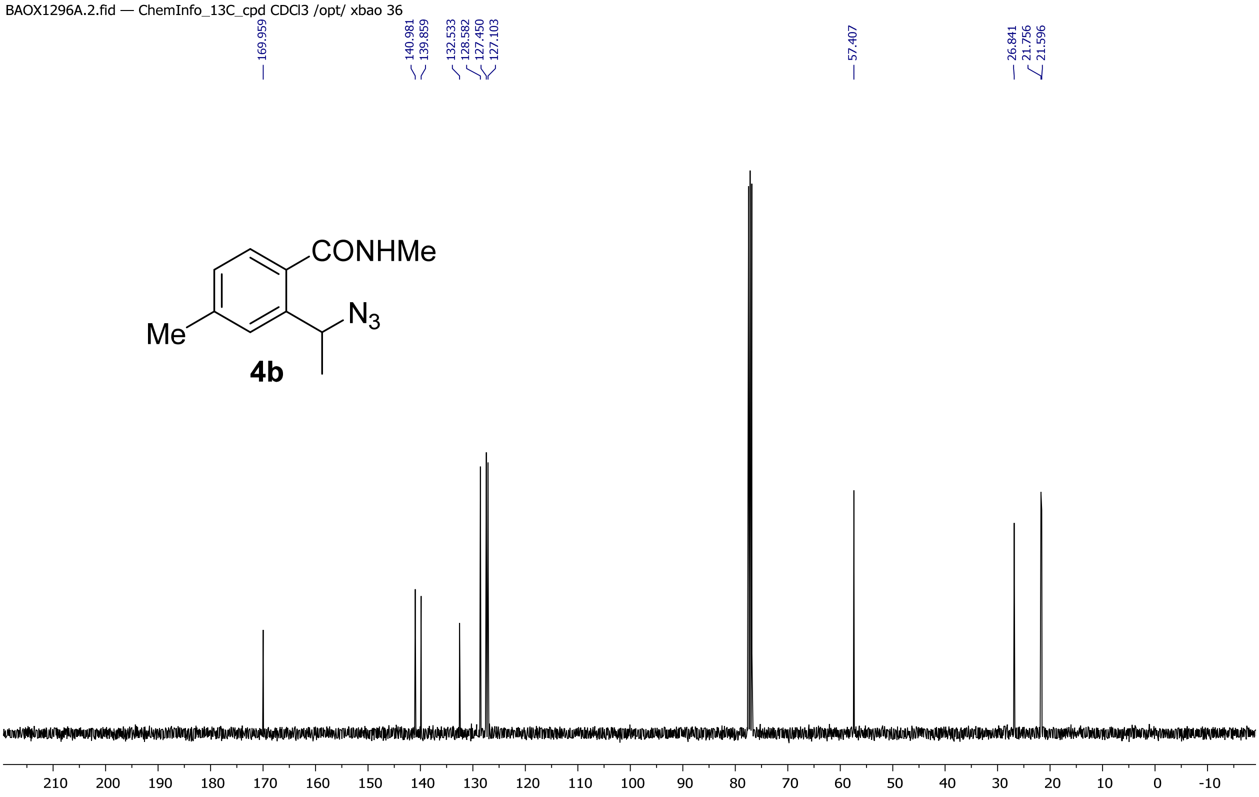

Supplementary Figure 4. ^{13}C NMR spectra of 4b

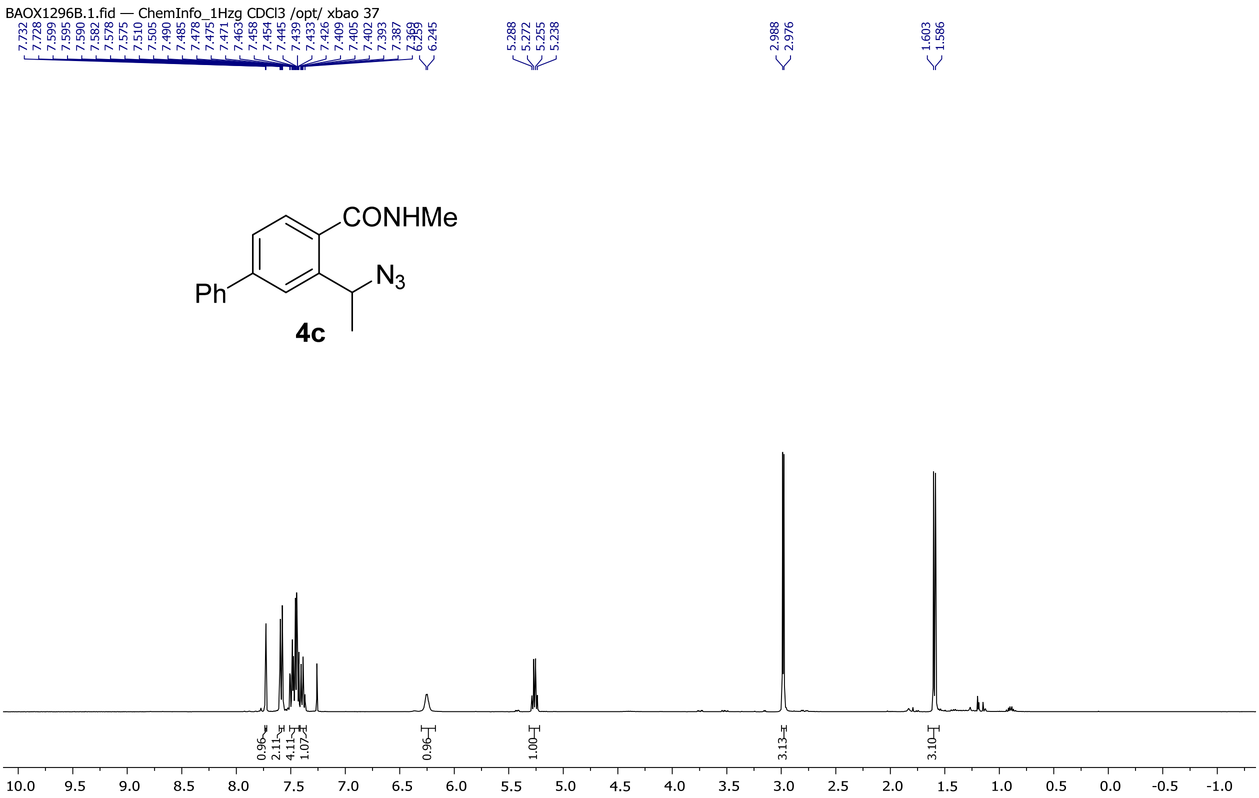

Supplementary Figure 5. ^{1}H spectra of 4c

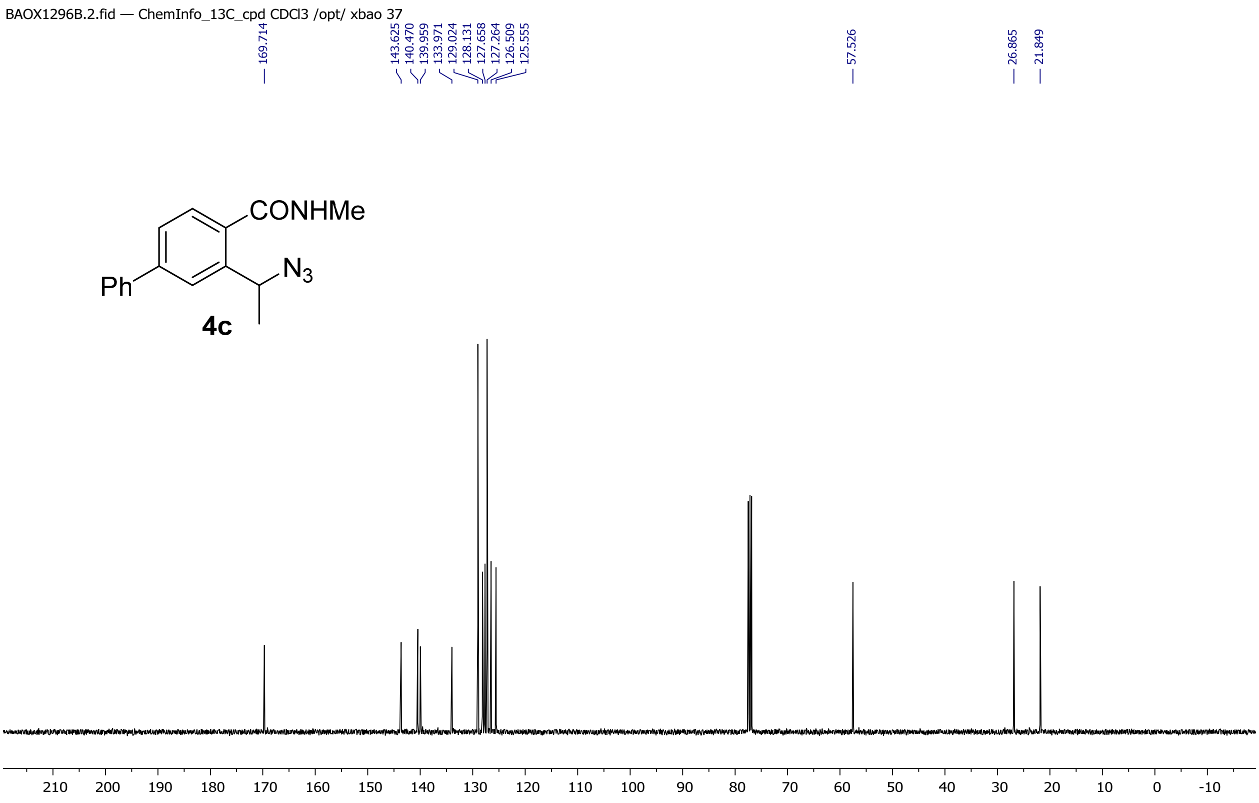

Supplementary Figure 6. ^{13}C NMR spectra of 4c

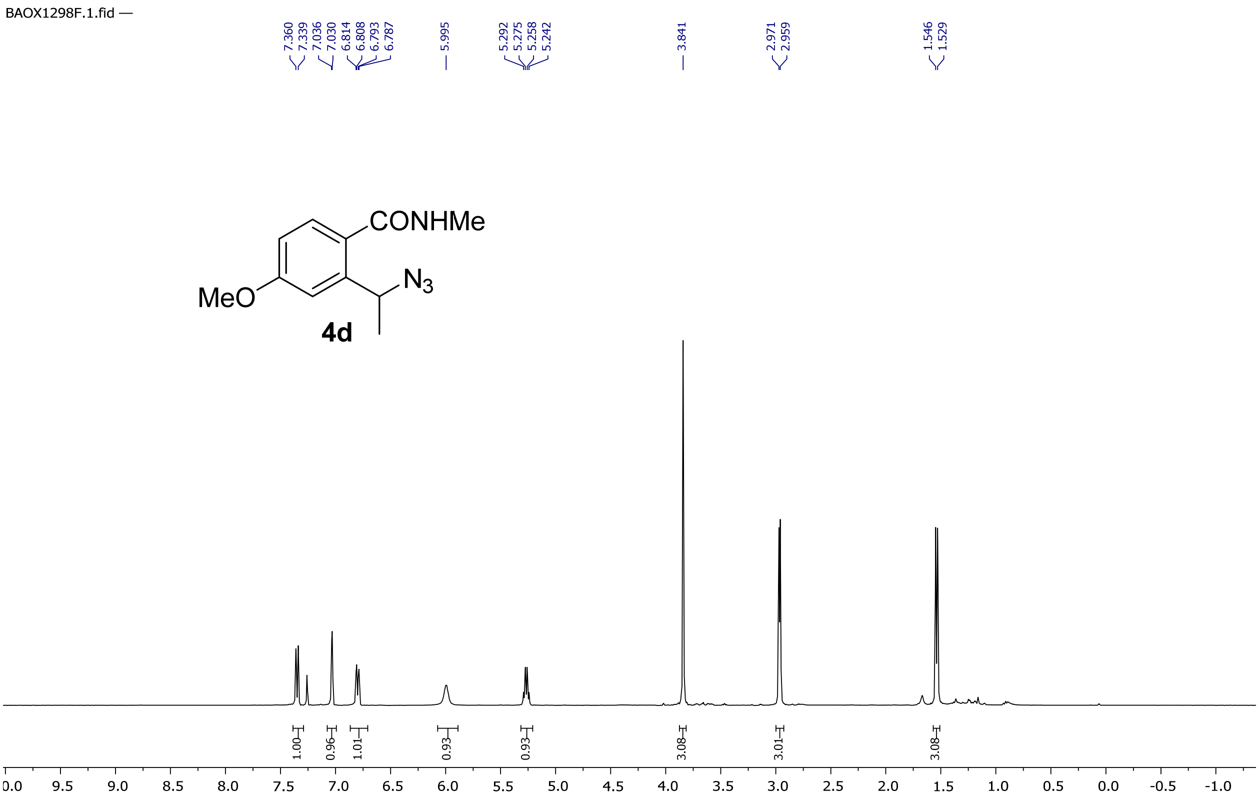

Supplementary Figure 7. ^{1}H spectra of 4d

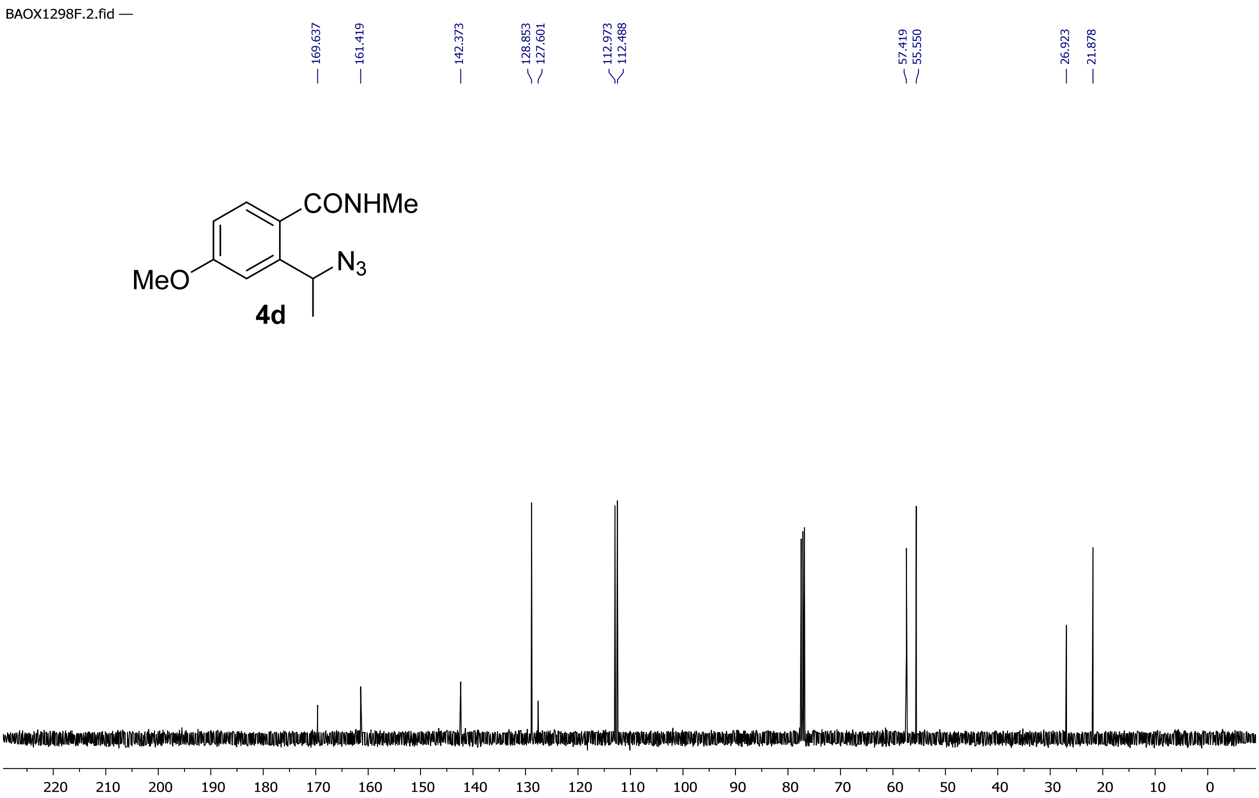

Supplementary Figure 8. ^{13}C NMR spectra of 4d

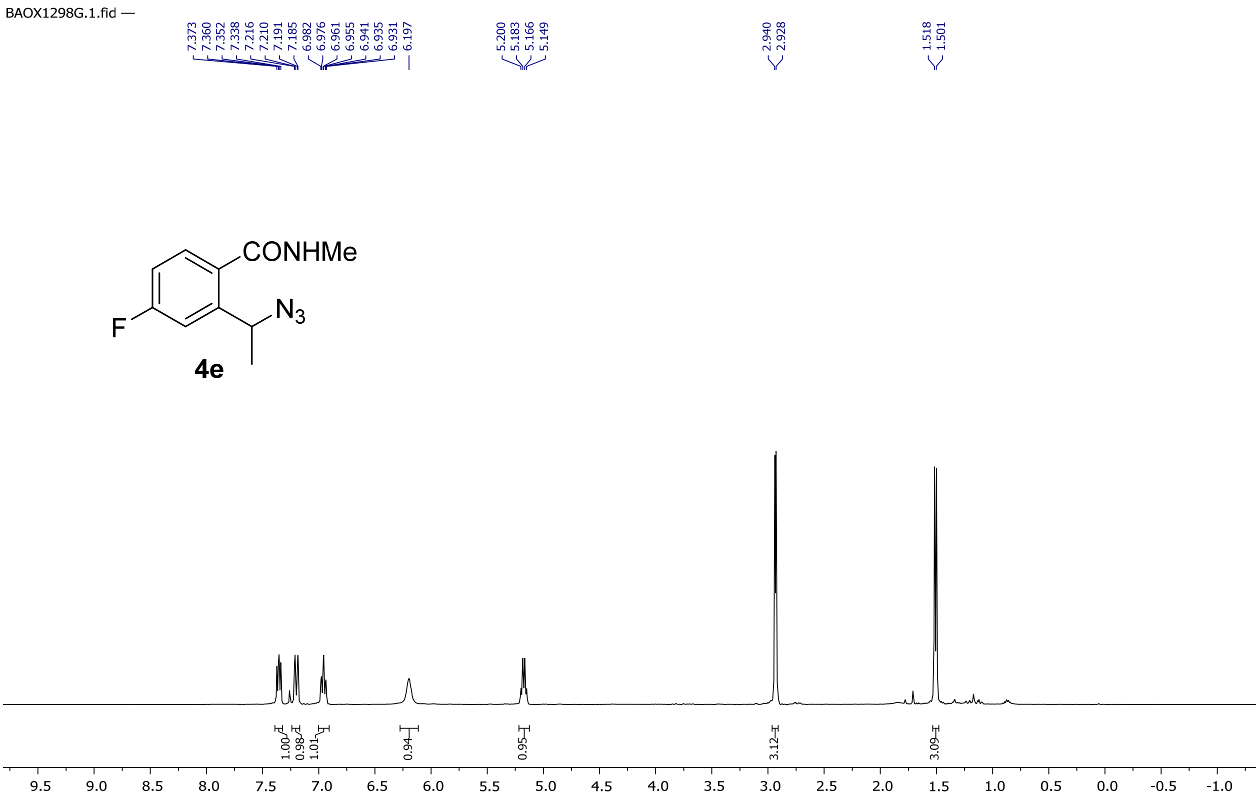

Supplementary Figure 9. ^{1}H spectra of 4e

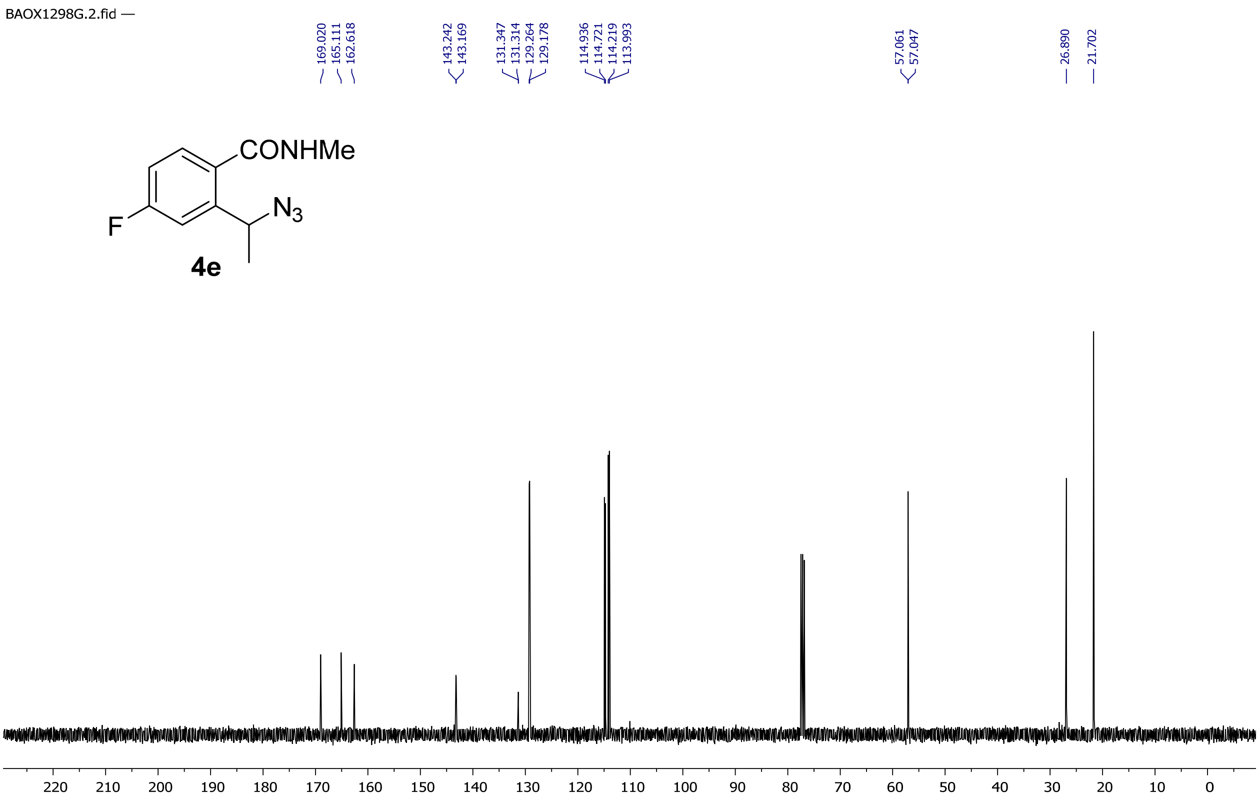

Supplementary Figure 10. ^{13}C NMR spectra of 4e

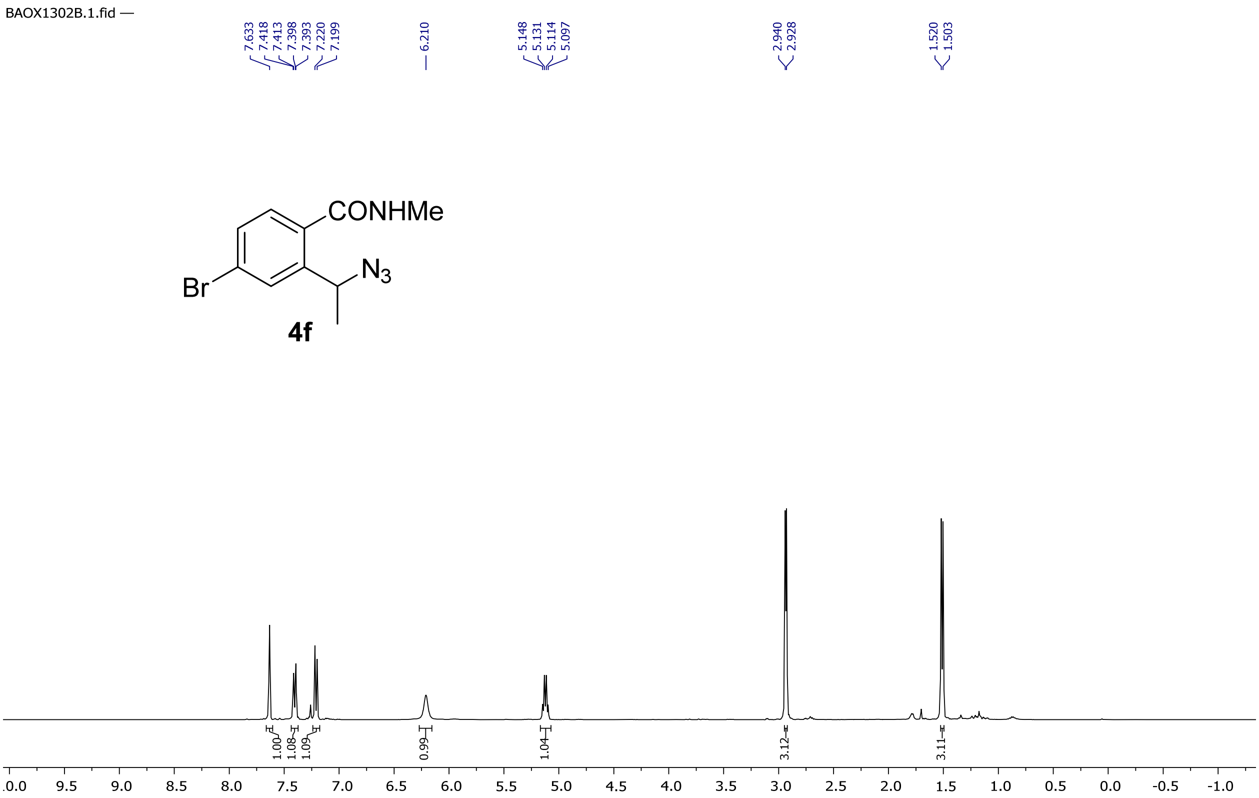

Supplementary Figure 11. ^{1}H spectra of 4f

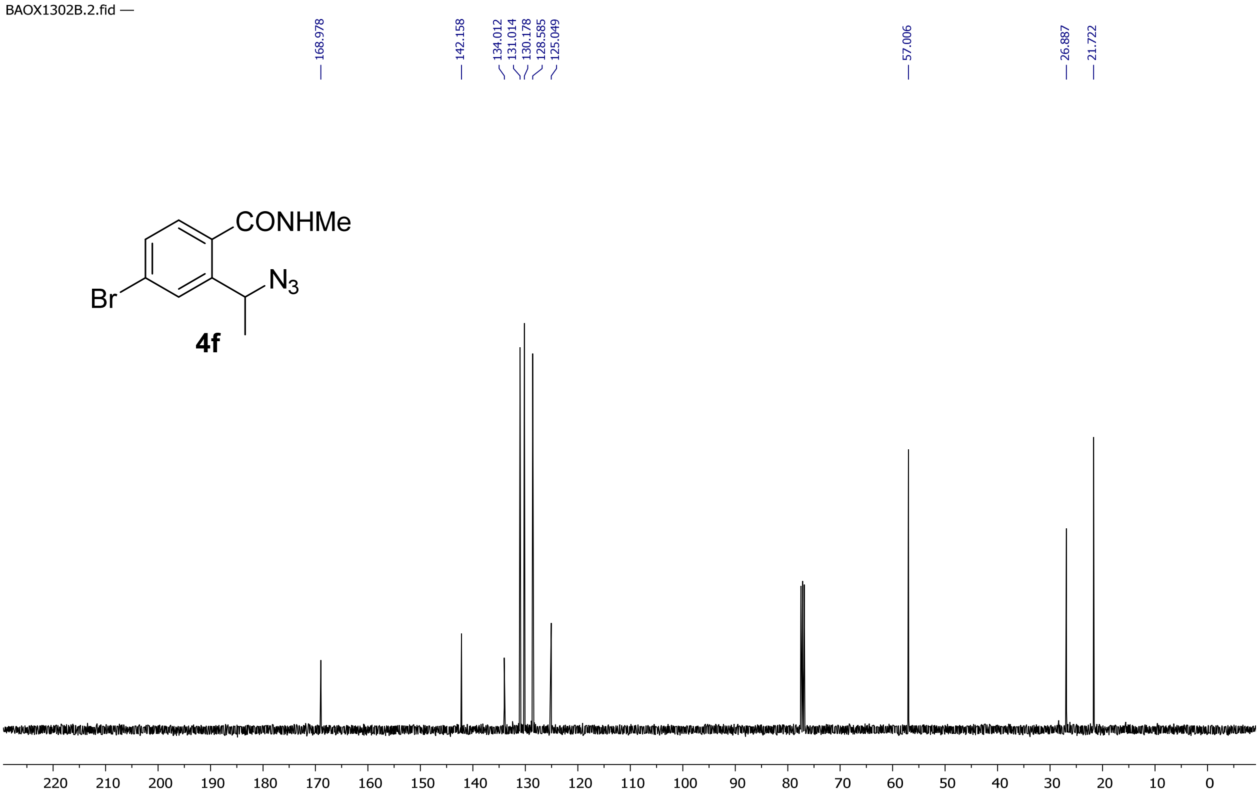

Supplementary Figure 12. ^{13}C NMR spectra of 4f

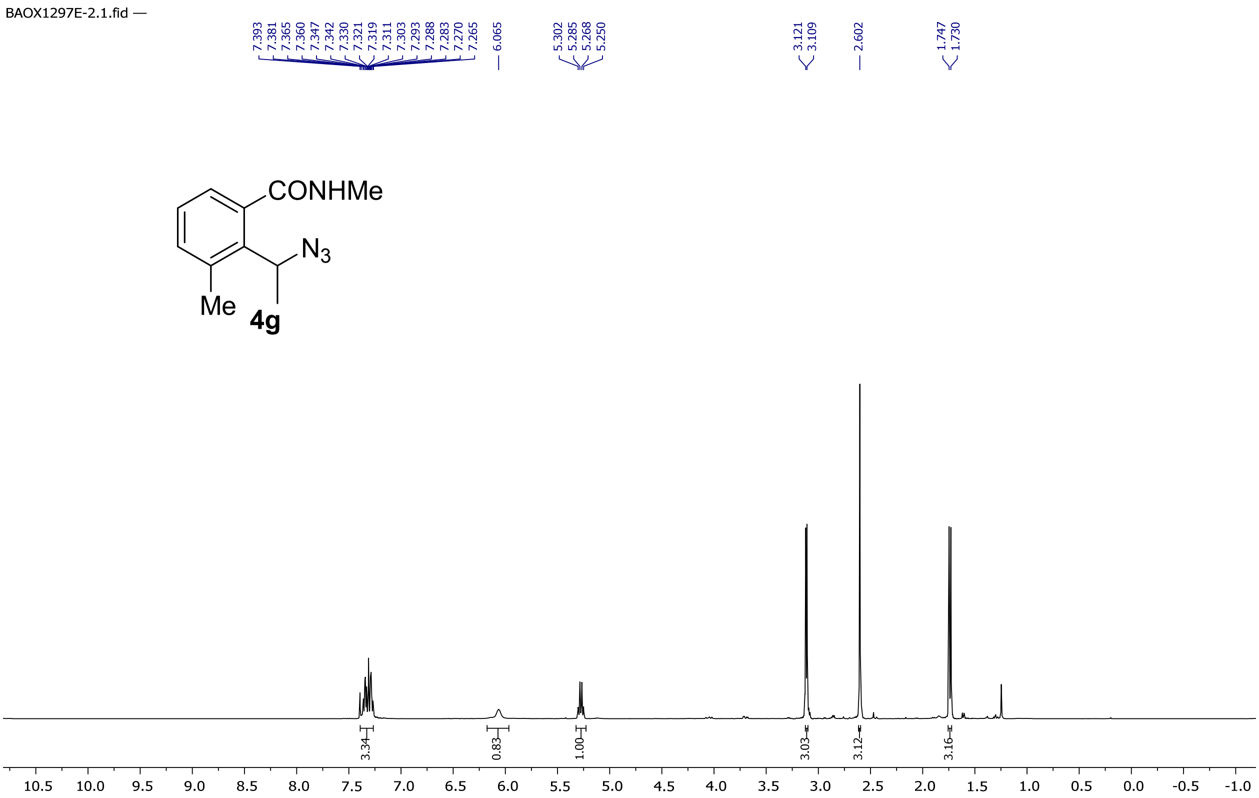

Supplementary Figure 13. ^{1}H spectra of 4g

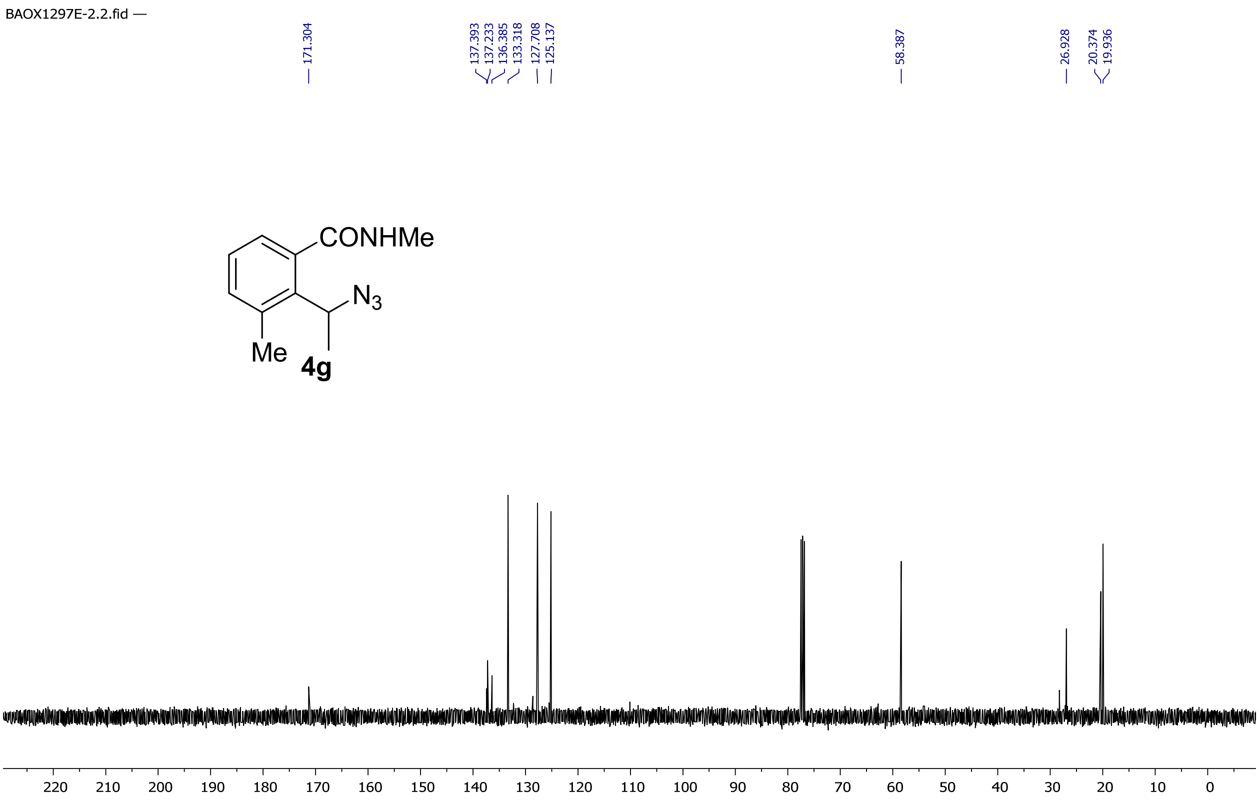

Supplementary Figure 14. ^{13}C NMR spectra of 4g

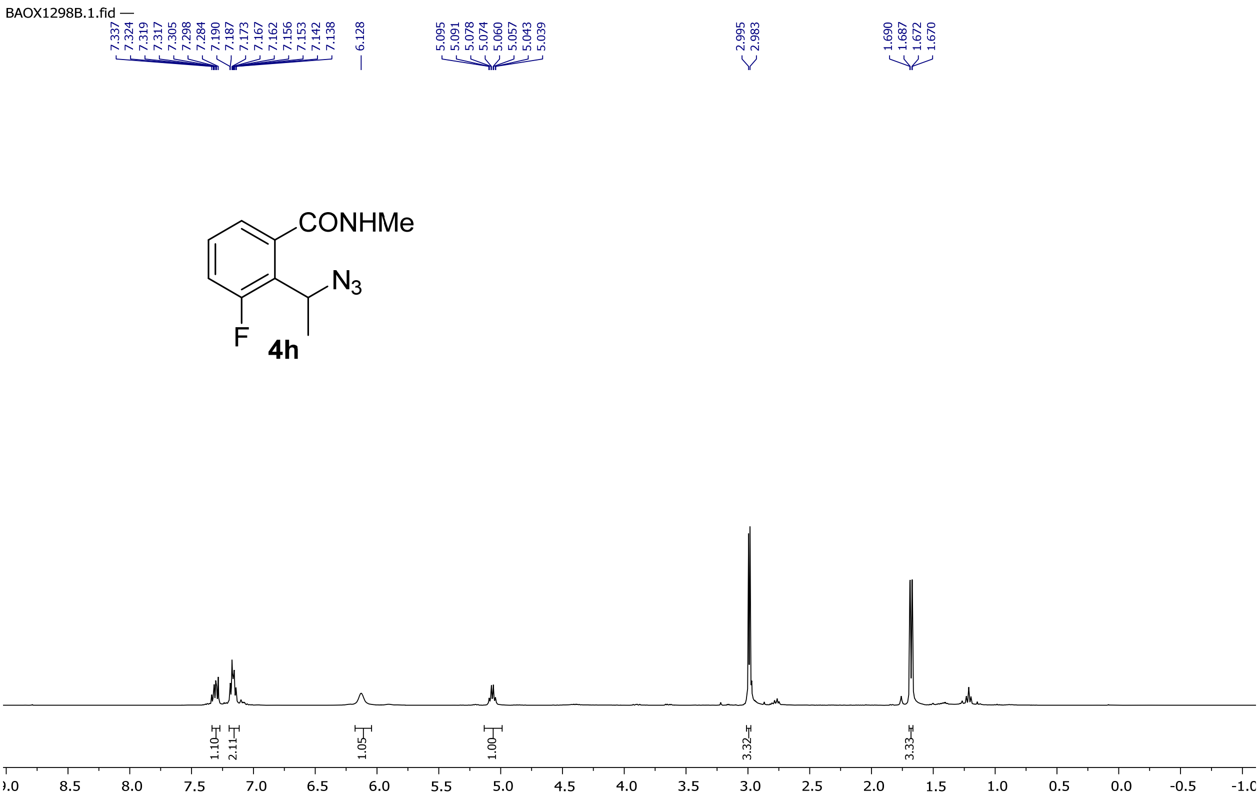

Supplementary Figure 15. ^{1}H spectra of 4h

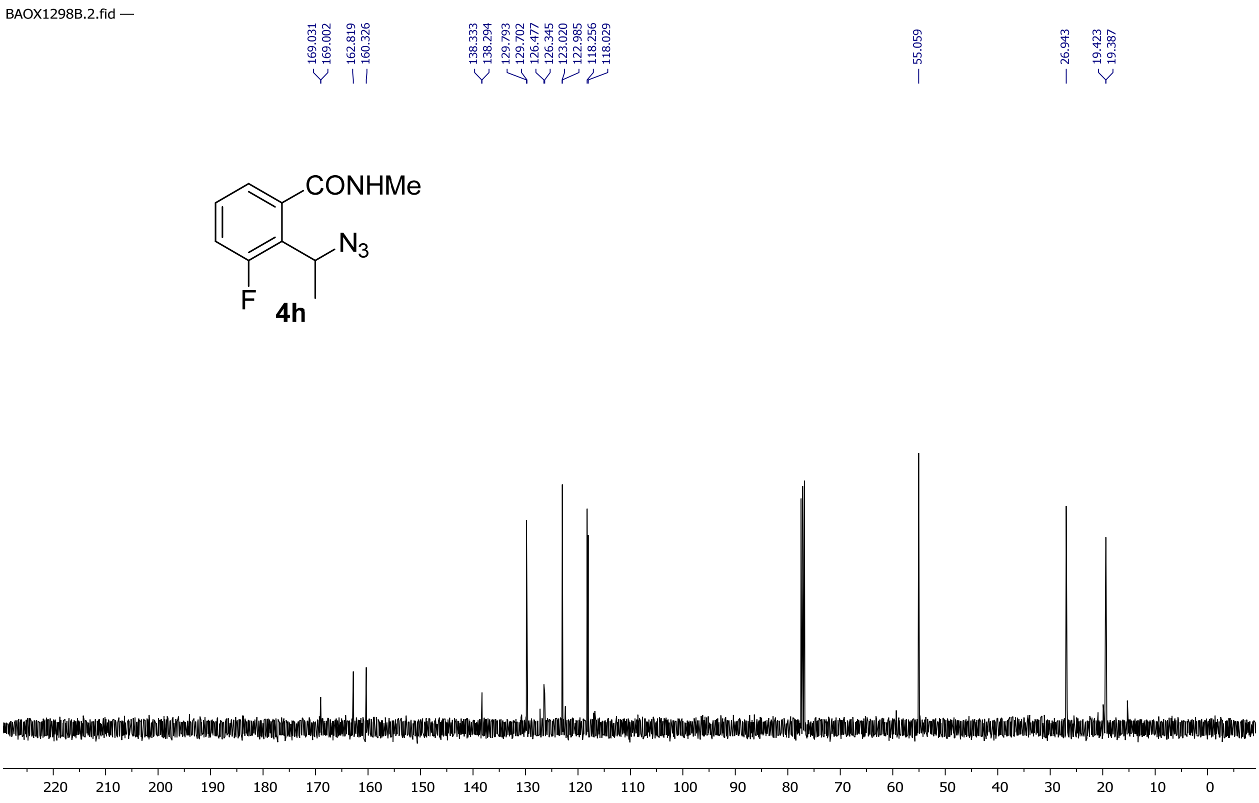

Supplementary Figure 16. ^{13}C NMR spectra of 4h

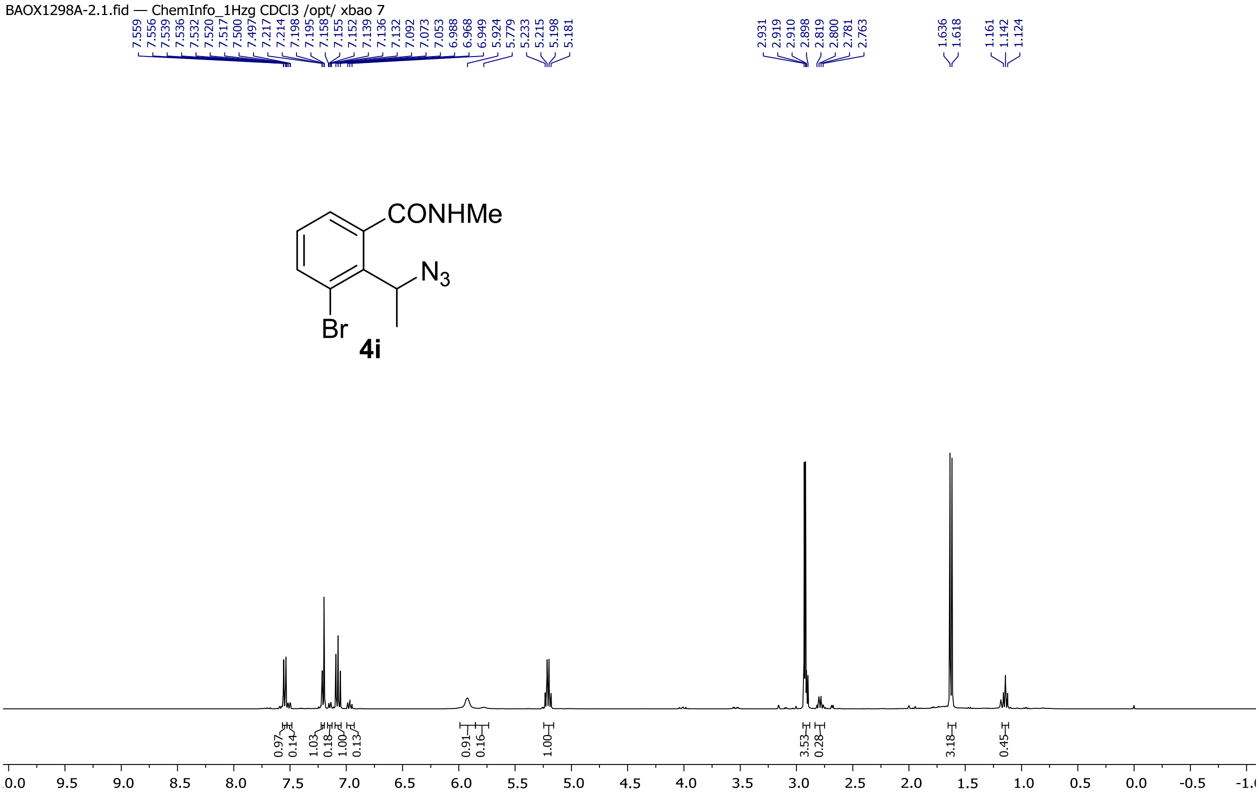

Supplementary Figure 17. ^{1}H spectra of 4i

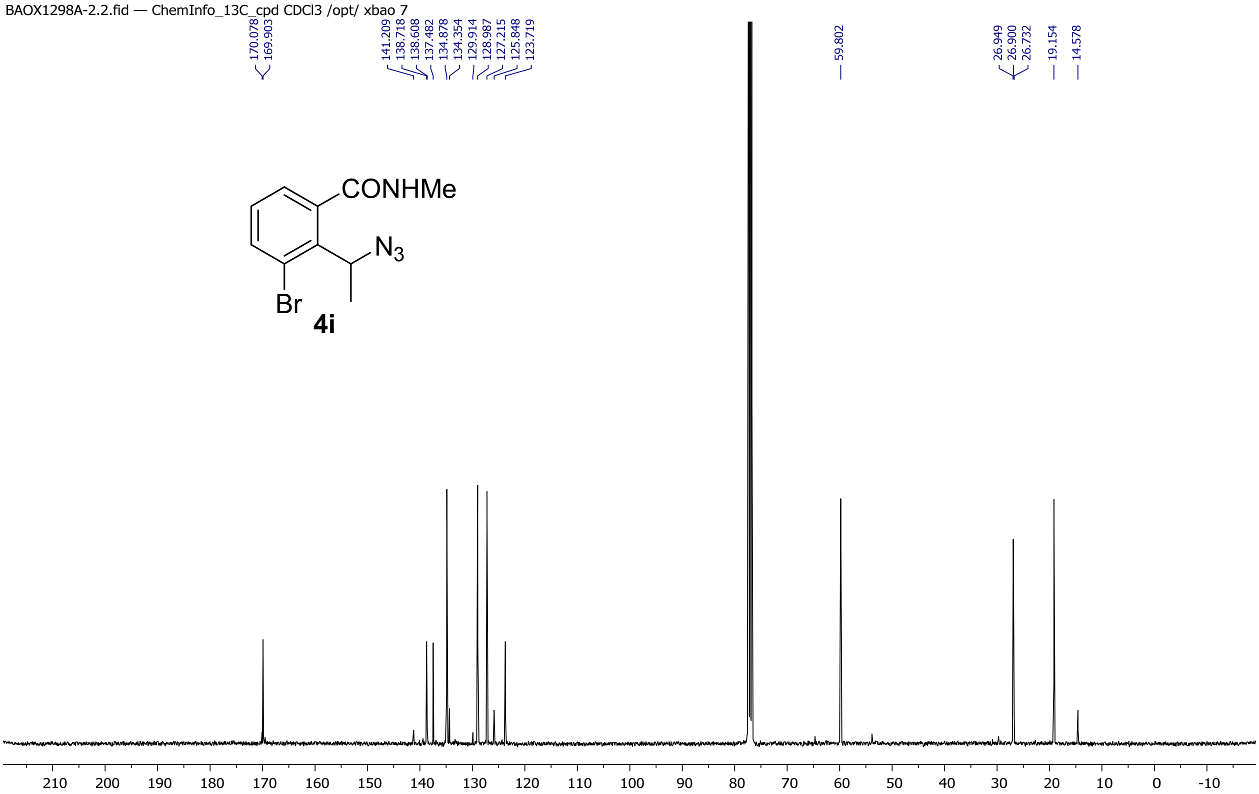

Supplementary Figure 18. ^{13}C NMR spectra of 4i

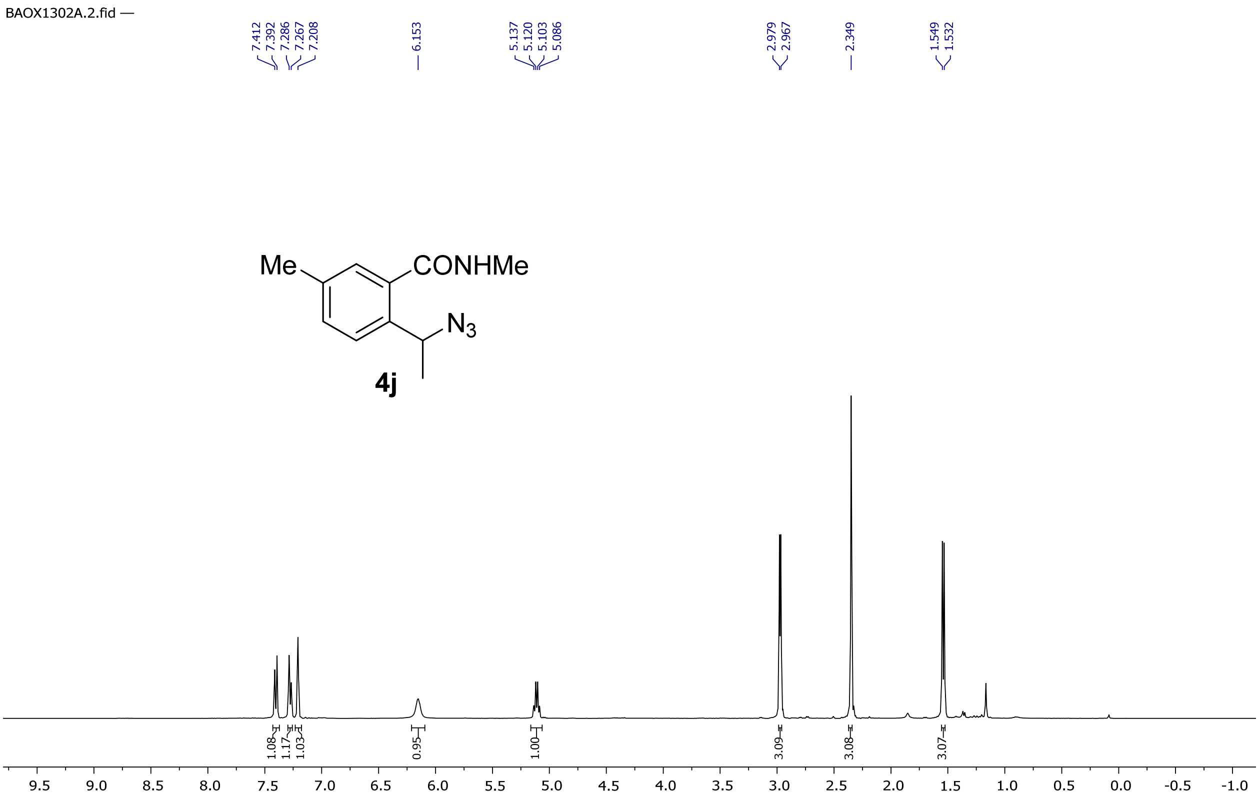

Supplementary Figure 19. ^{1}H spectra of 4j

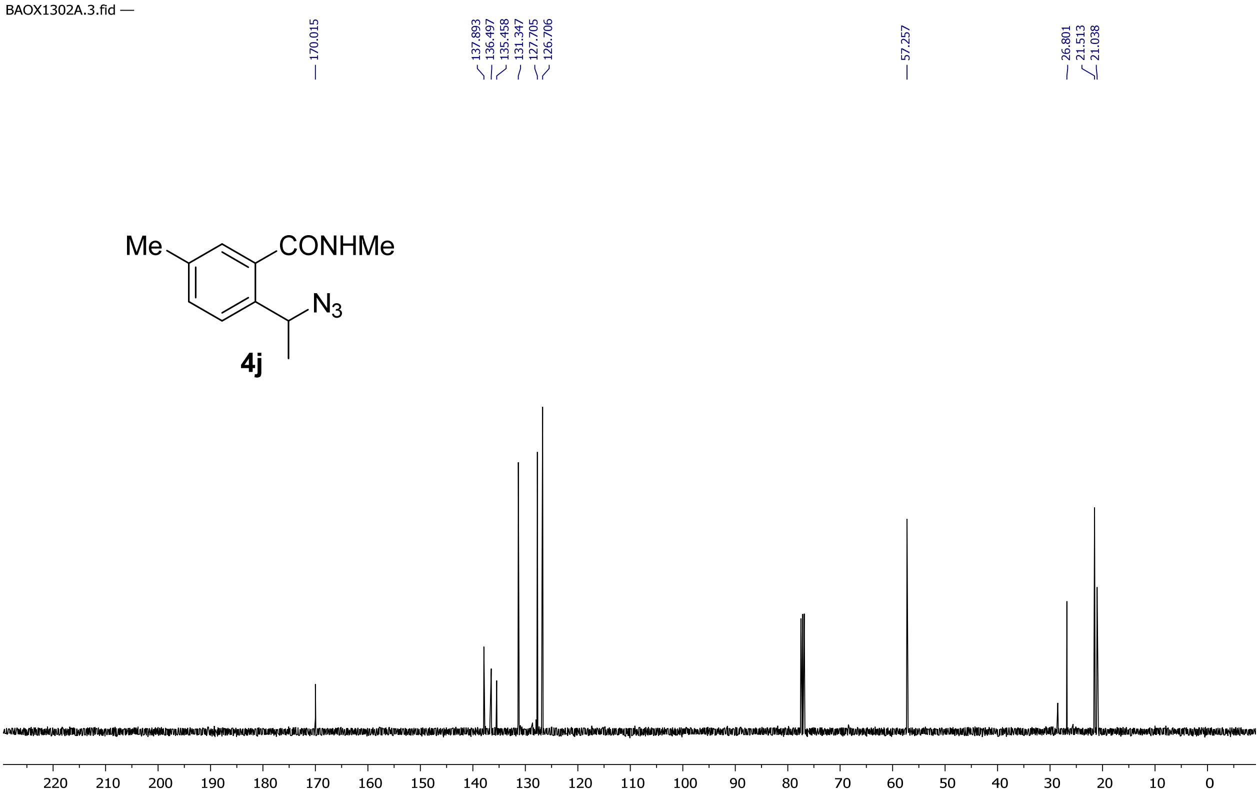

Supplementary Figure 20. ^{13}C NMR spectra of 4j

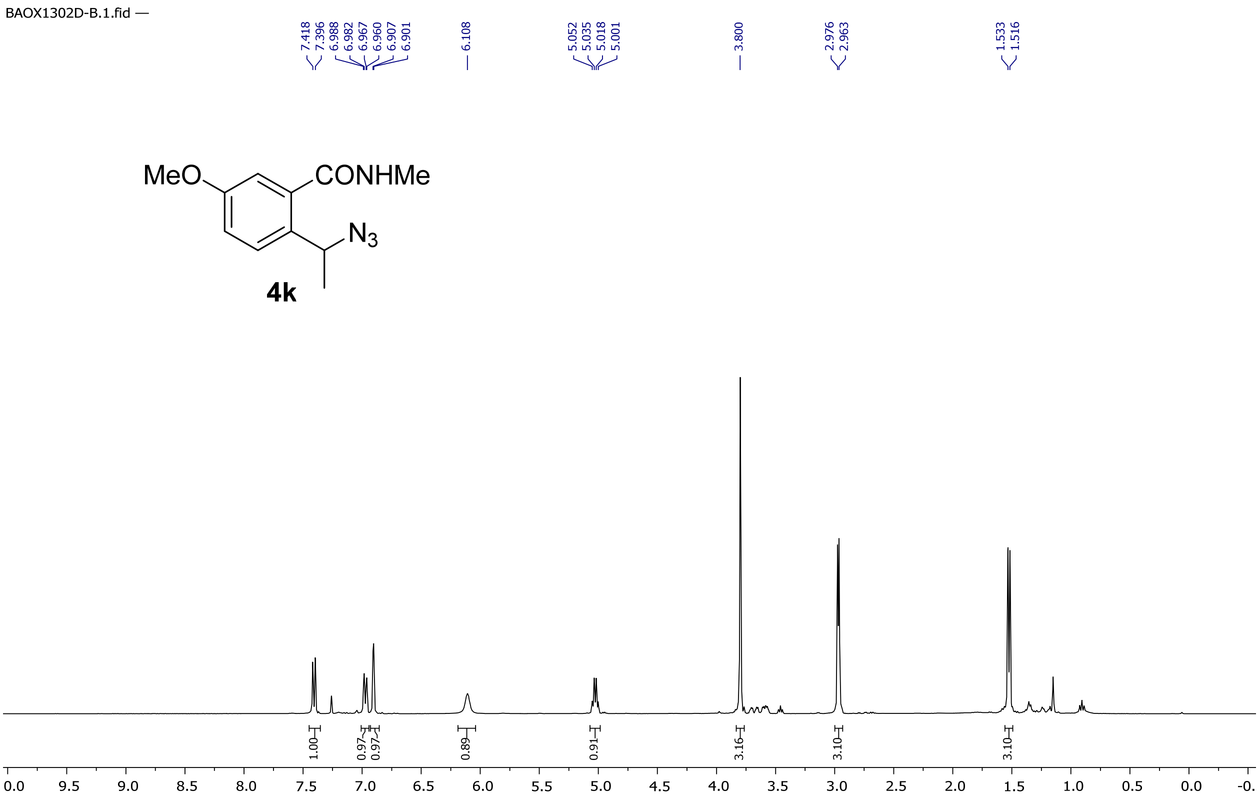

Supplementary Figure 21. ^{1}H spectra of 4k

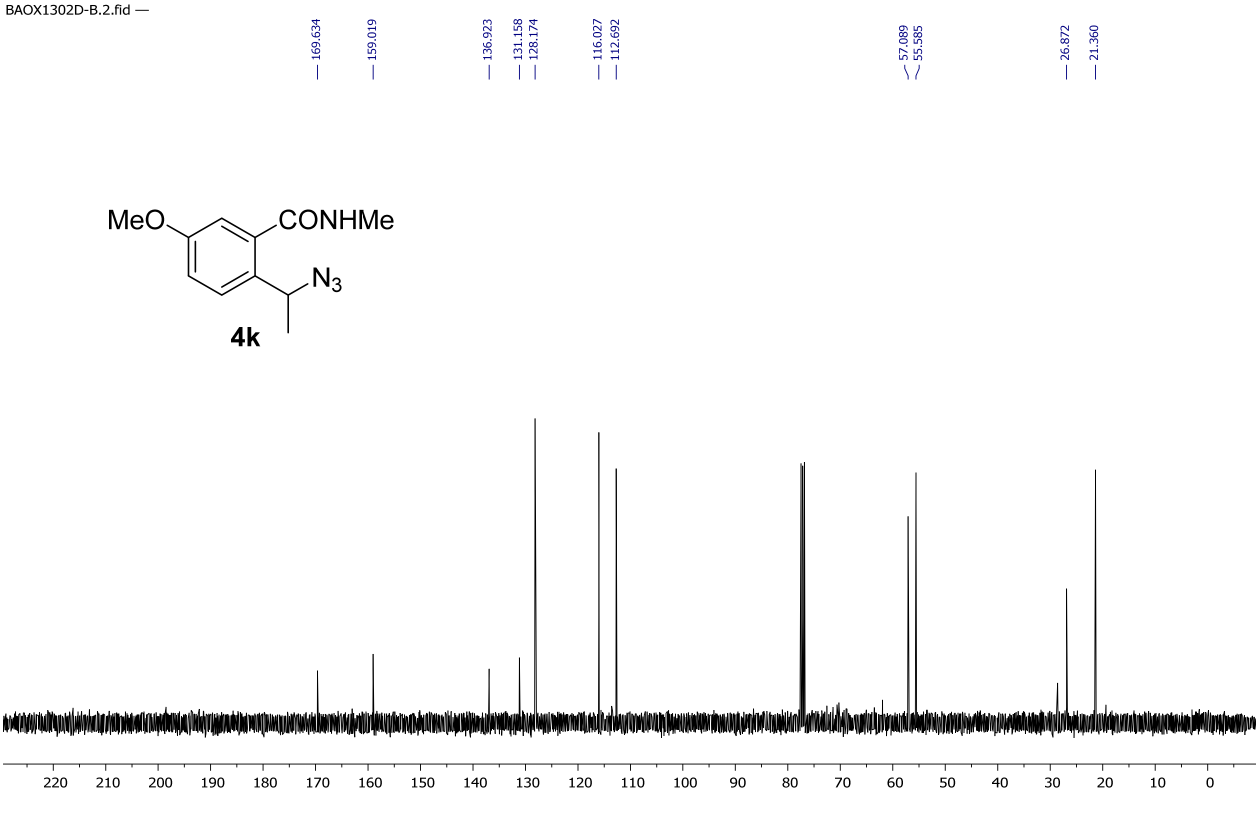

Supplementary Figure 22. ^{13}C NMR spectra of 4k

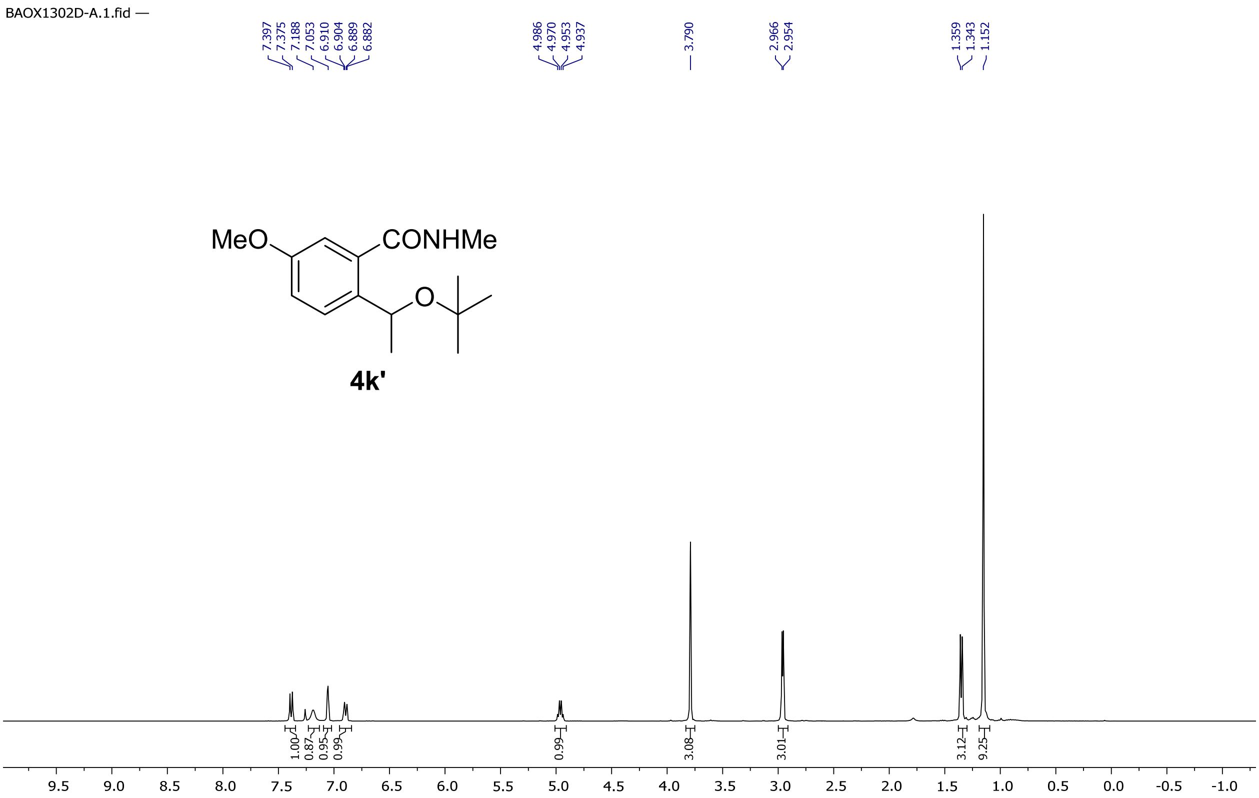

Supplementary Figure 23. ^{1}H spectra of 4k'

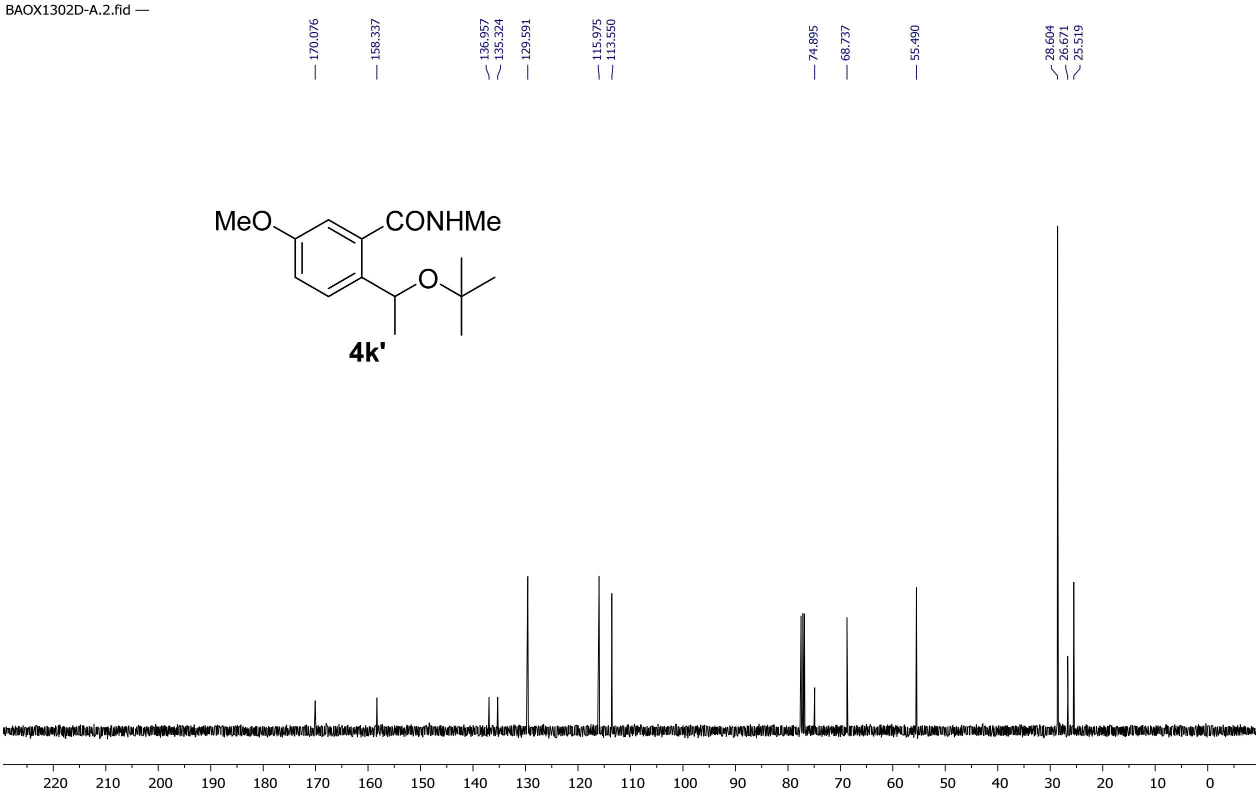

Supplementary Figure 24. ^{13}C NMR spectra of 4k'

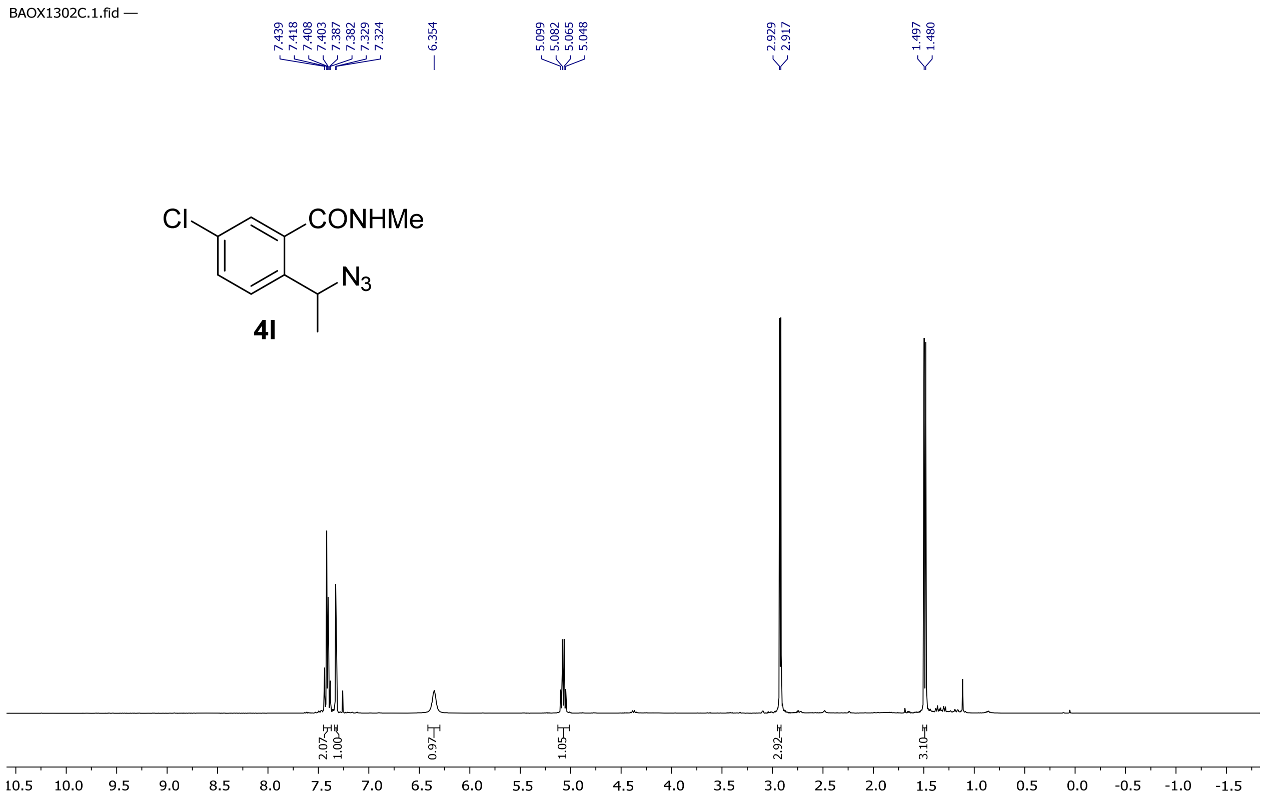

Supplementary Figure 25. ^{1}H spectra of 4l

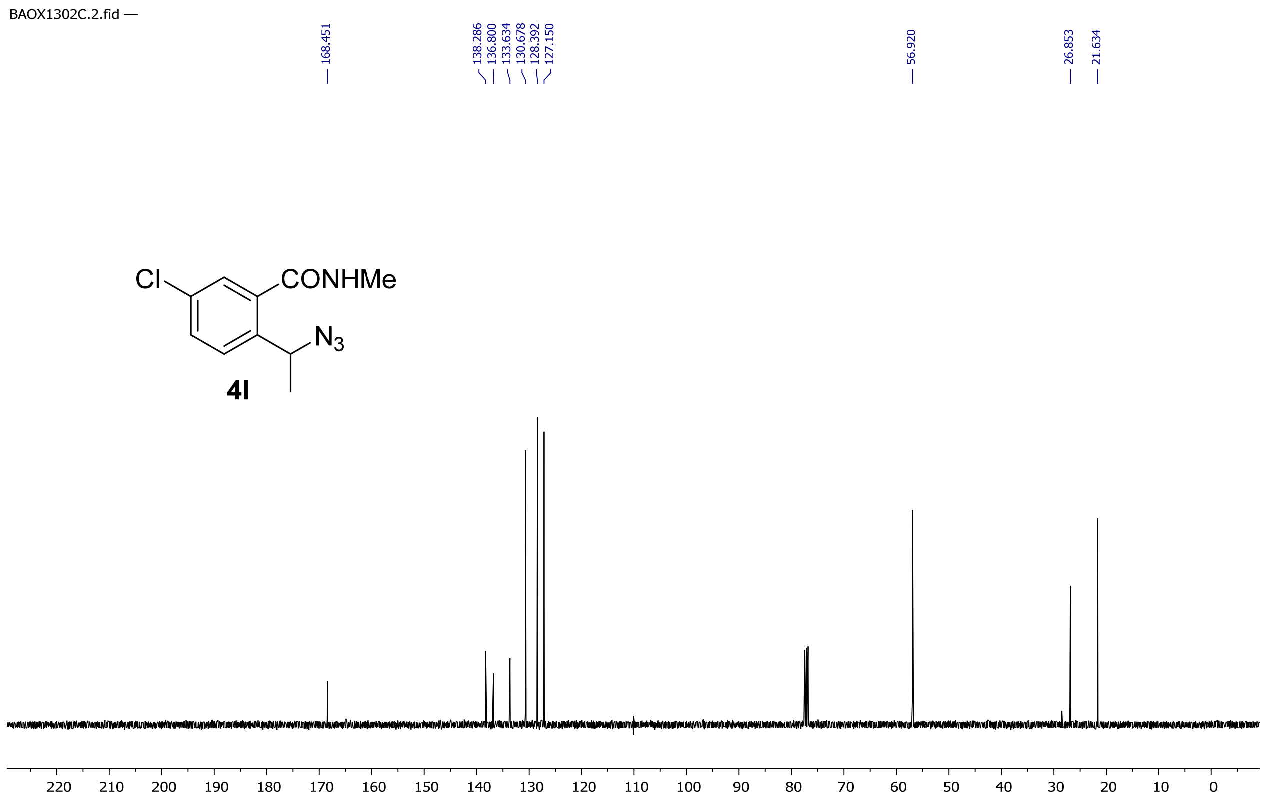

Supplementary Figure 26. ^{13}C NMR spectra of 4l

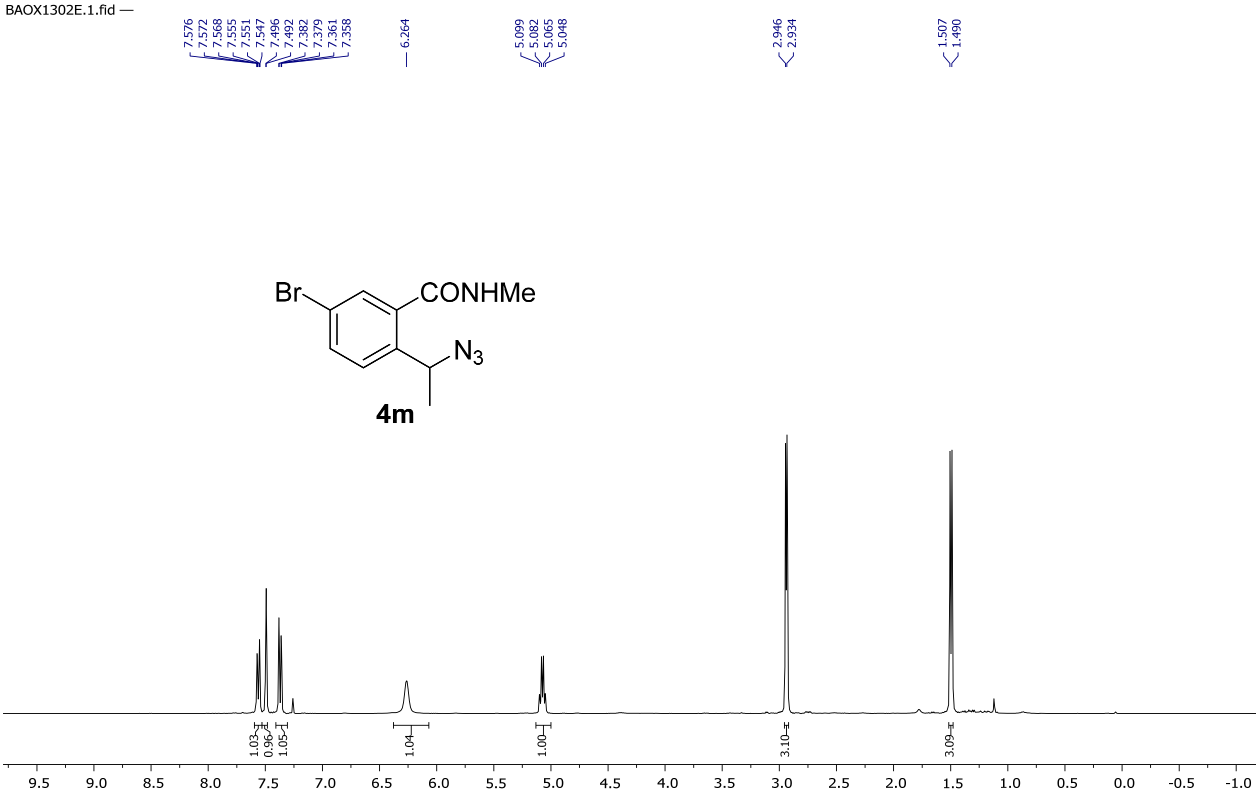

Supplementary Figure 27. ^{1}**H spectra of 4m**

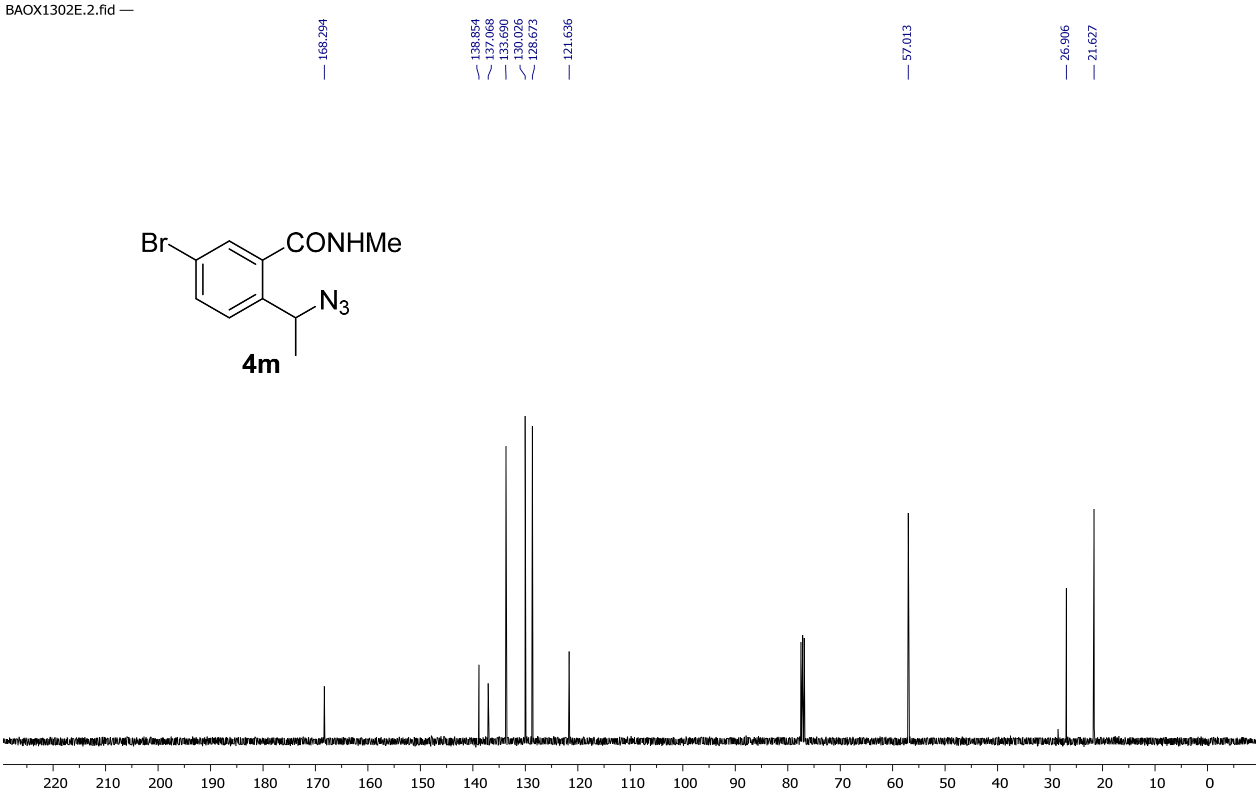

Supplementary Figure 28. ^{13}**C NMR spectra of 4m**

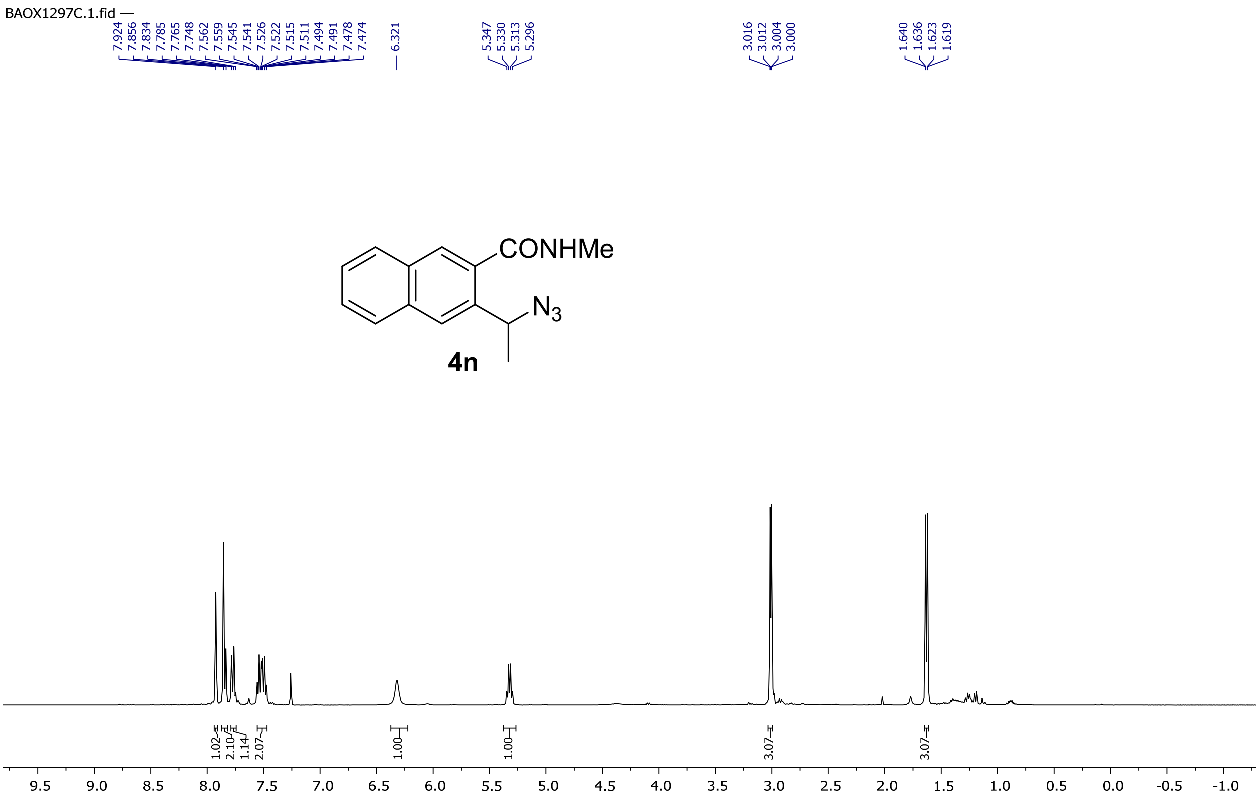

Supplementary Figure 29. ^{1}H spectra of 4n

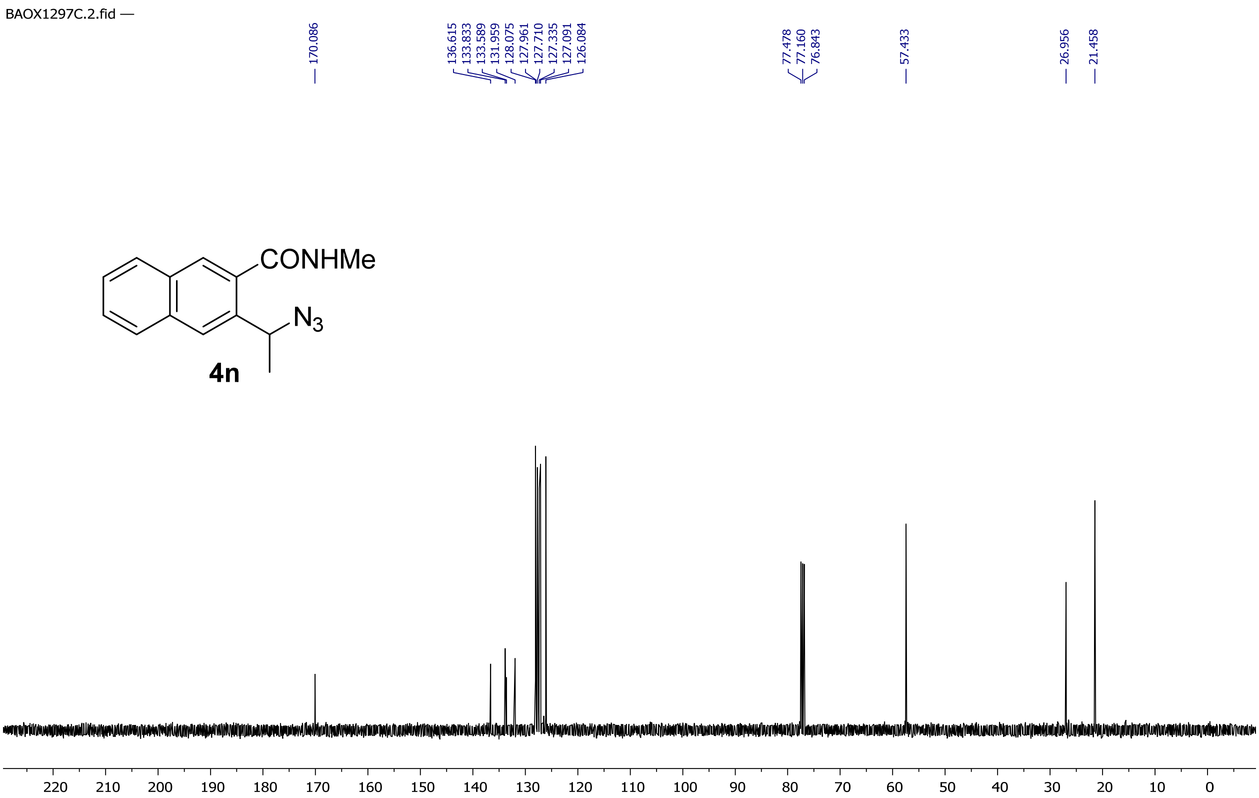

Supplementary Figure 30. ^{13}C NMR spectra of 4n

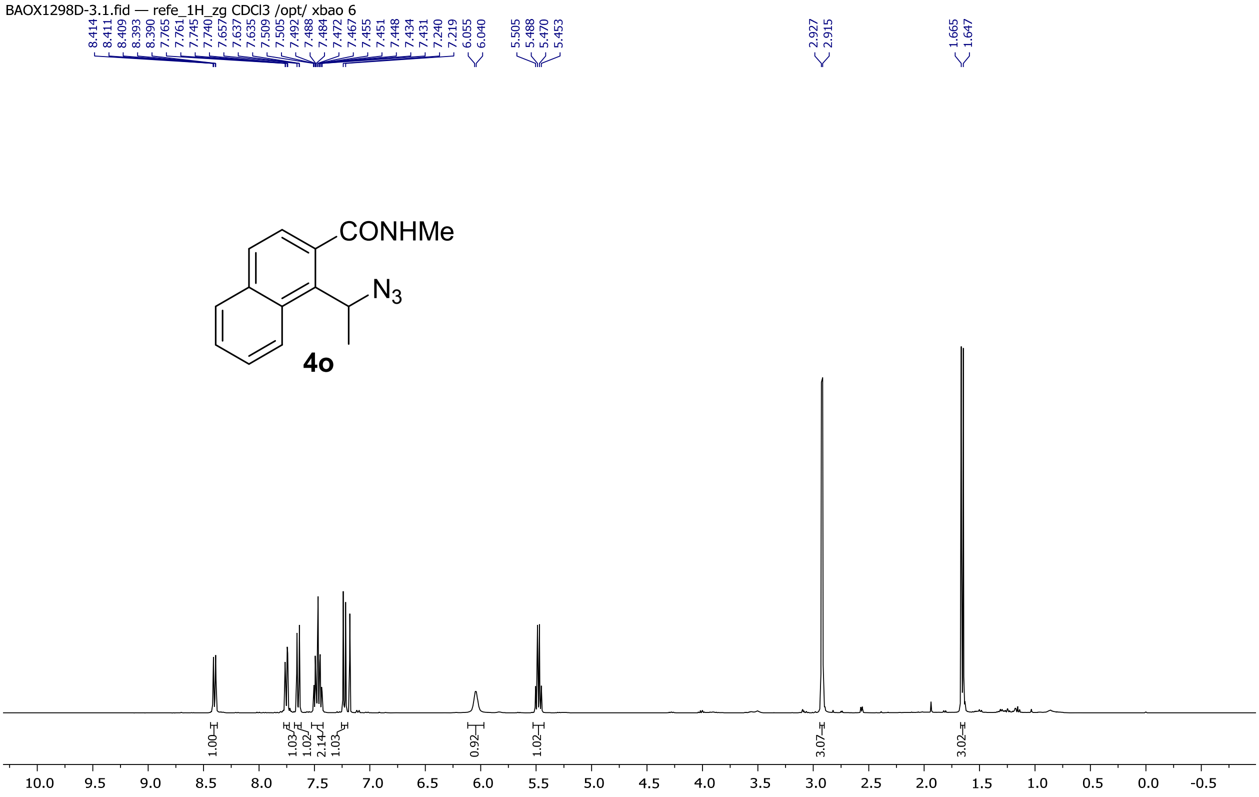

Supplementary Figure 31. ^{1}H spectra of 4o

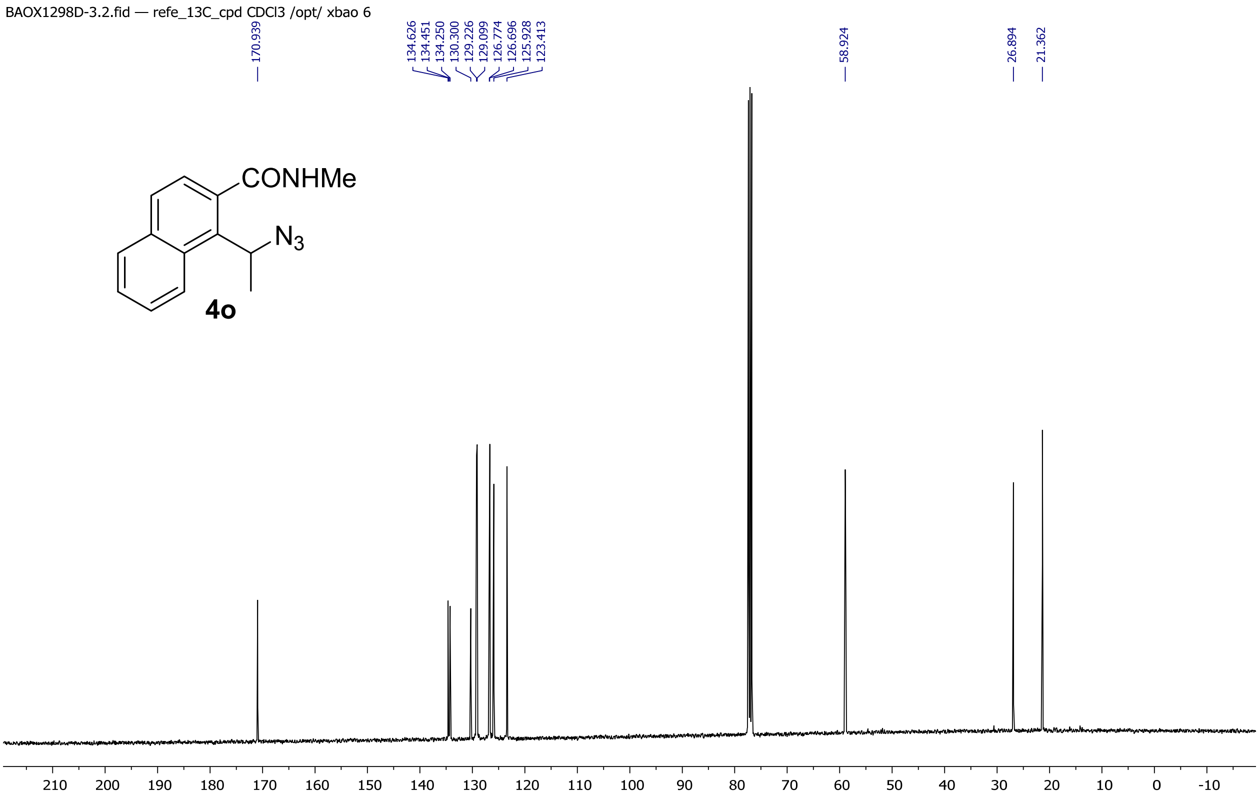

Supplementary Figure 32. ^{13}C NMR spectra of 4o

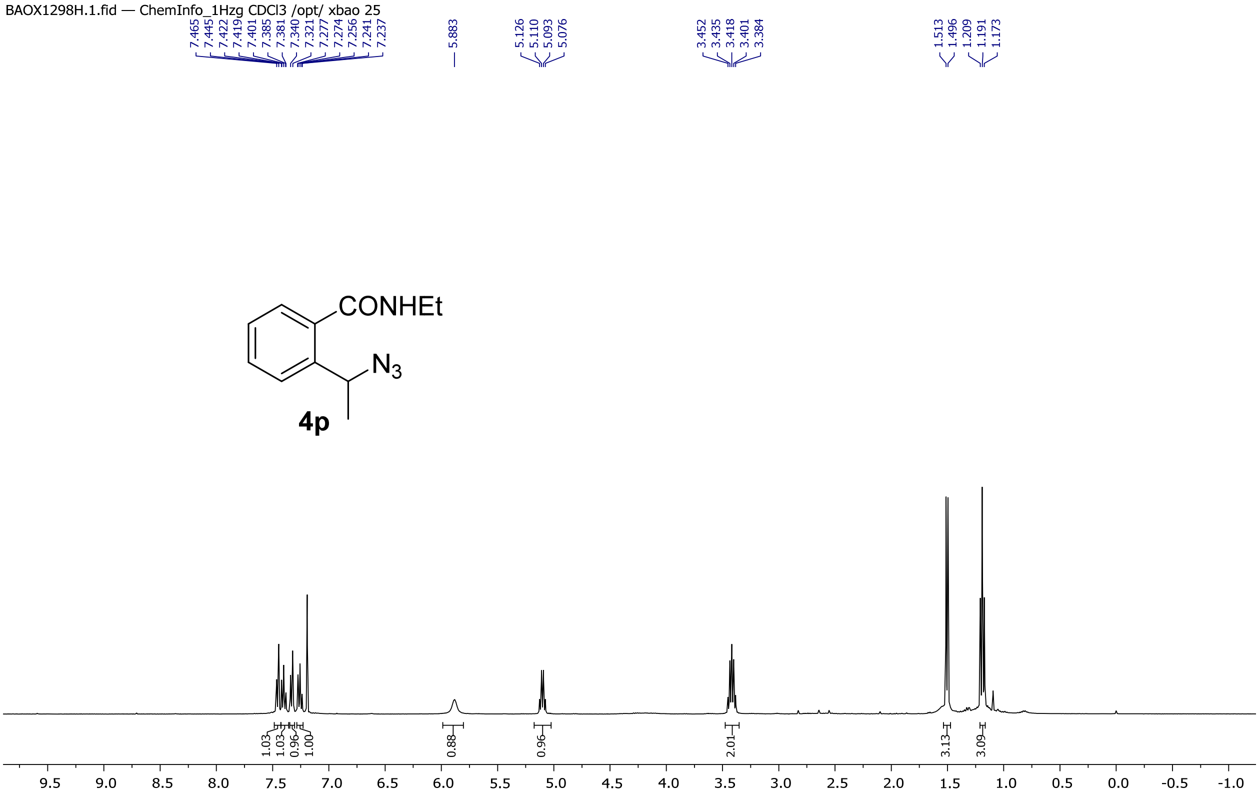

Supplementary Figure 33. ^{1}H spectra of 4p

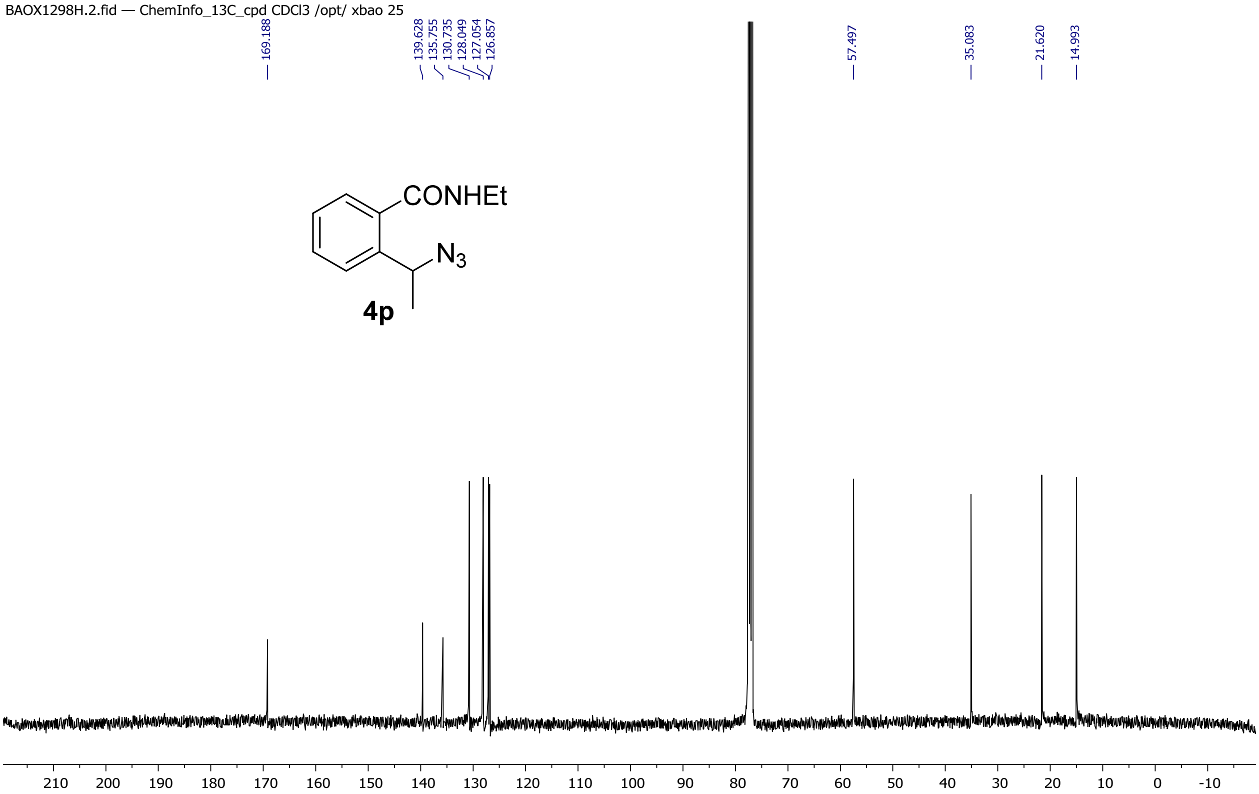

Supplementary Figure 34. ^{13}C NMR spectra of 4p

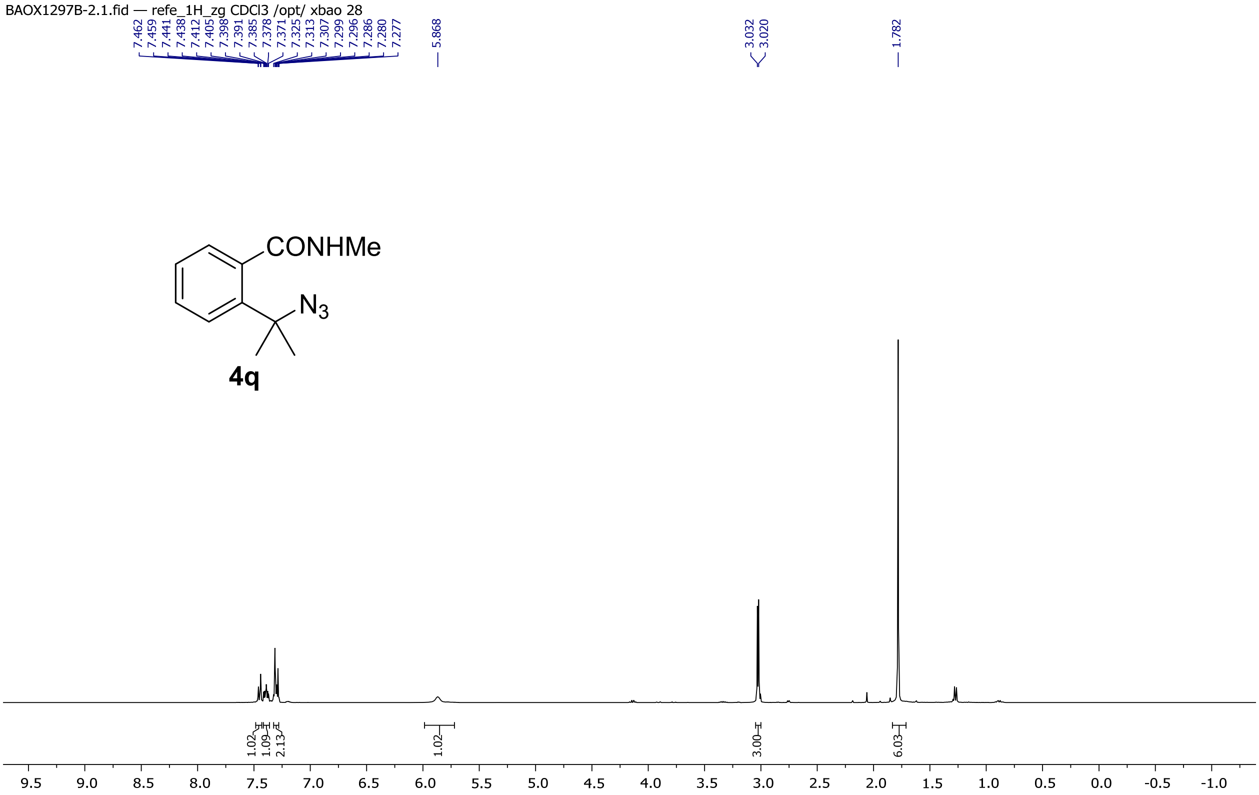

Supplementary Figure 35. ^{1}H spectra of 4q

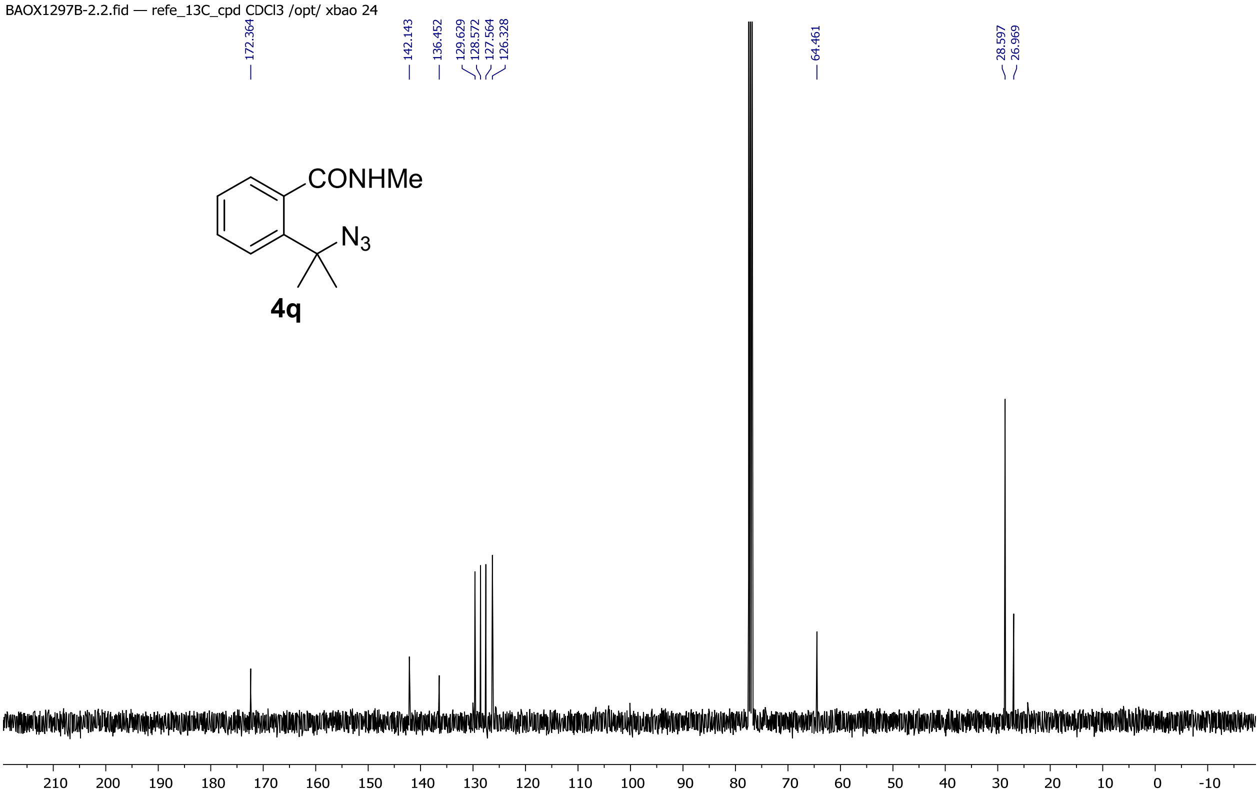

Supplementary Figure 36. ^{13}C NMR spectra of 4q

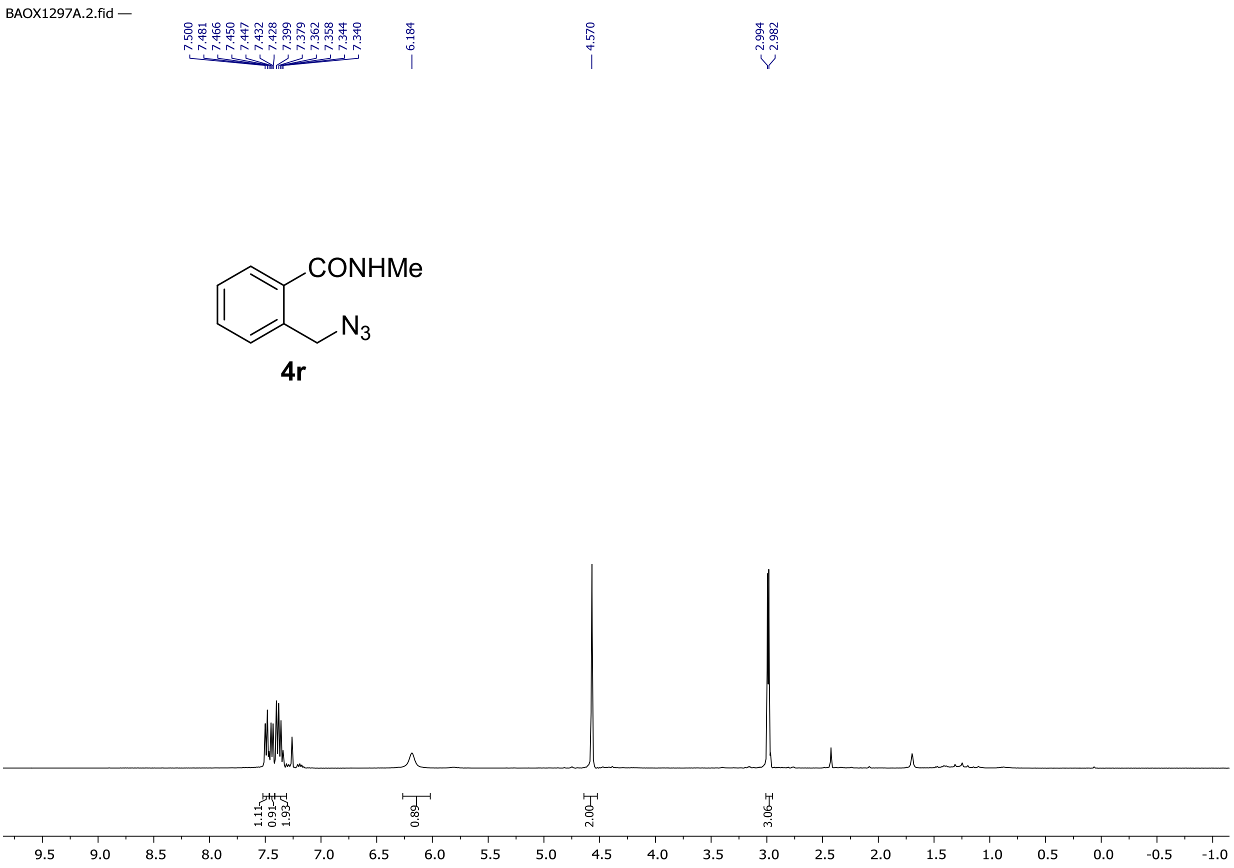

Supplementary Figure 37. ^{1}H spectra of 4r

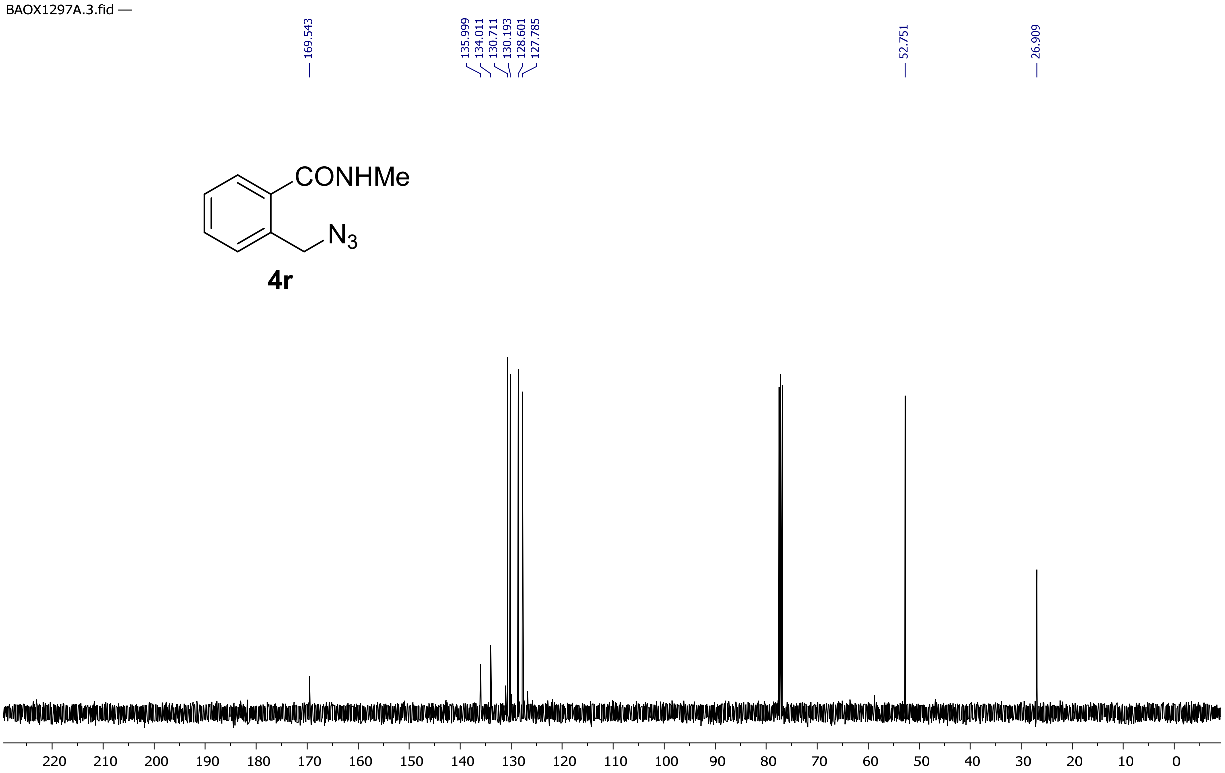

Supplementary Figure 38. ^{13}C NMR spectra of 4r

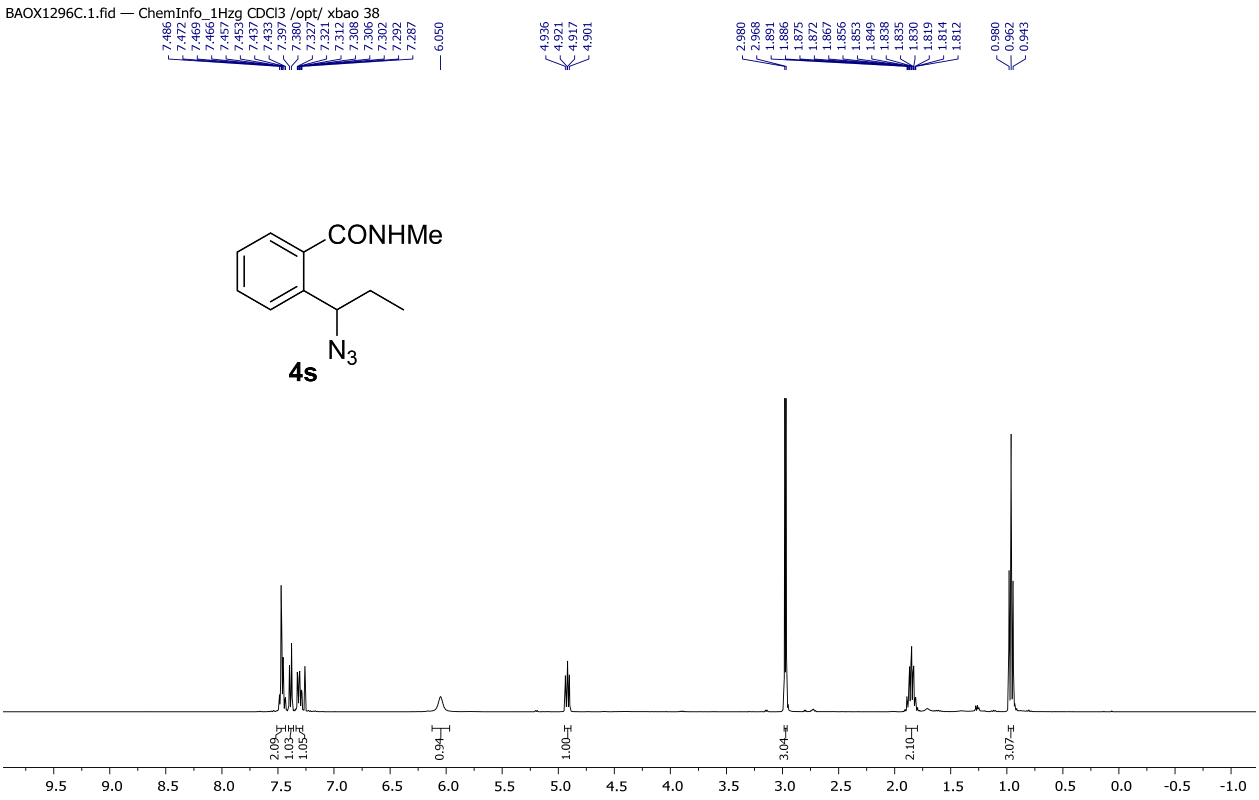

Supplementary Figure 39. ^{1}H spectra of 4s

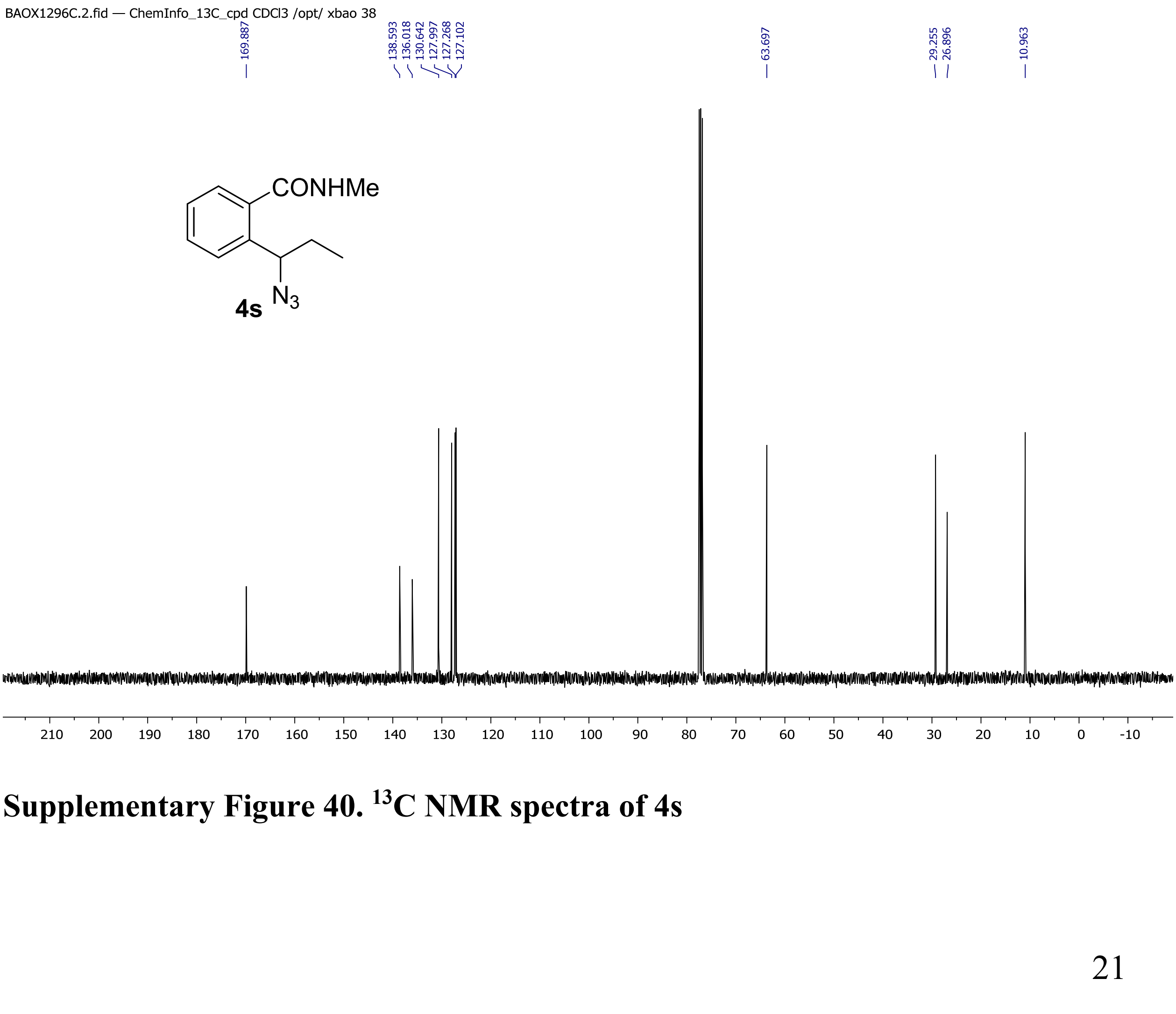

Supplementary Figure 40. ^{13}C NMR spectra of 4s

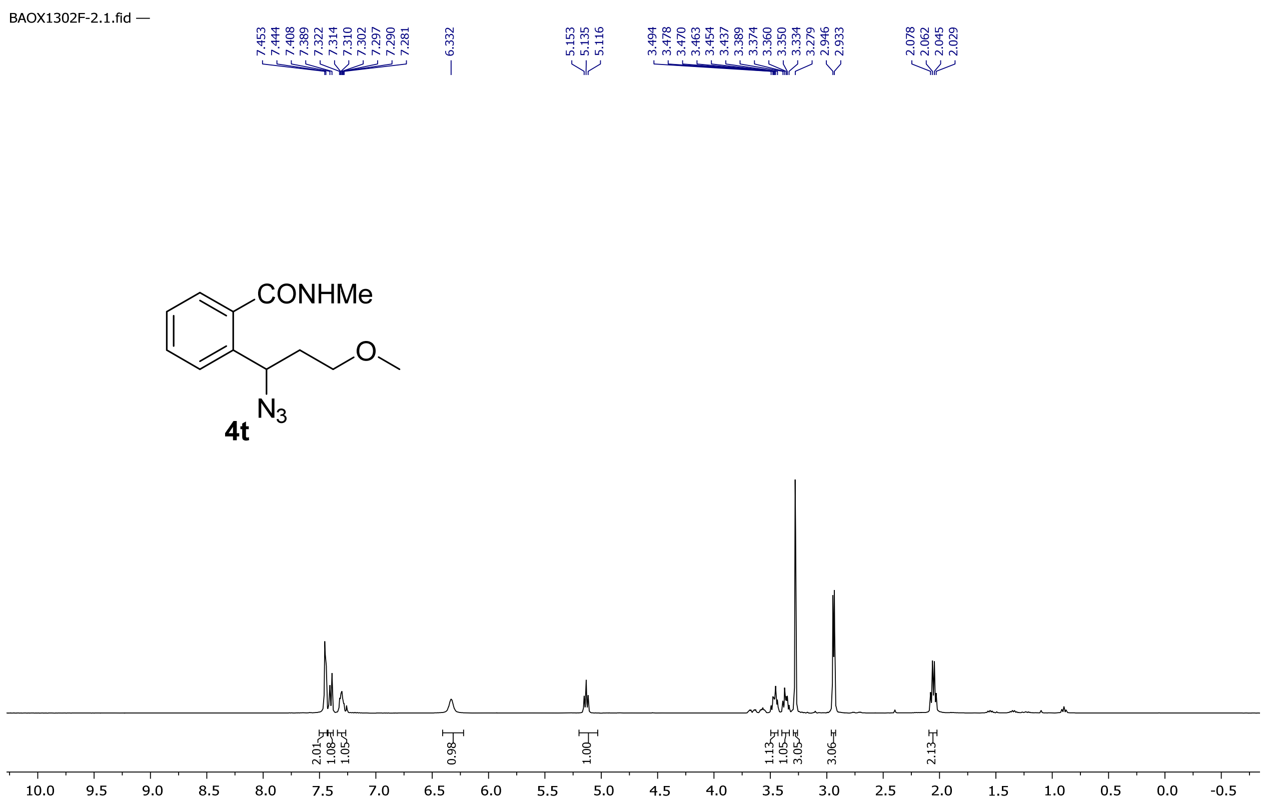

Supplementary Figure 41. ^{1}H spectra of 4t

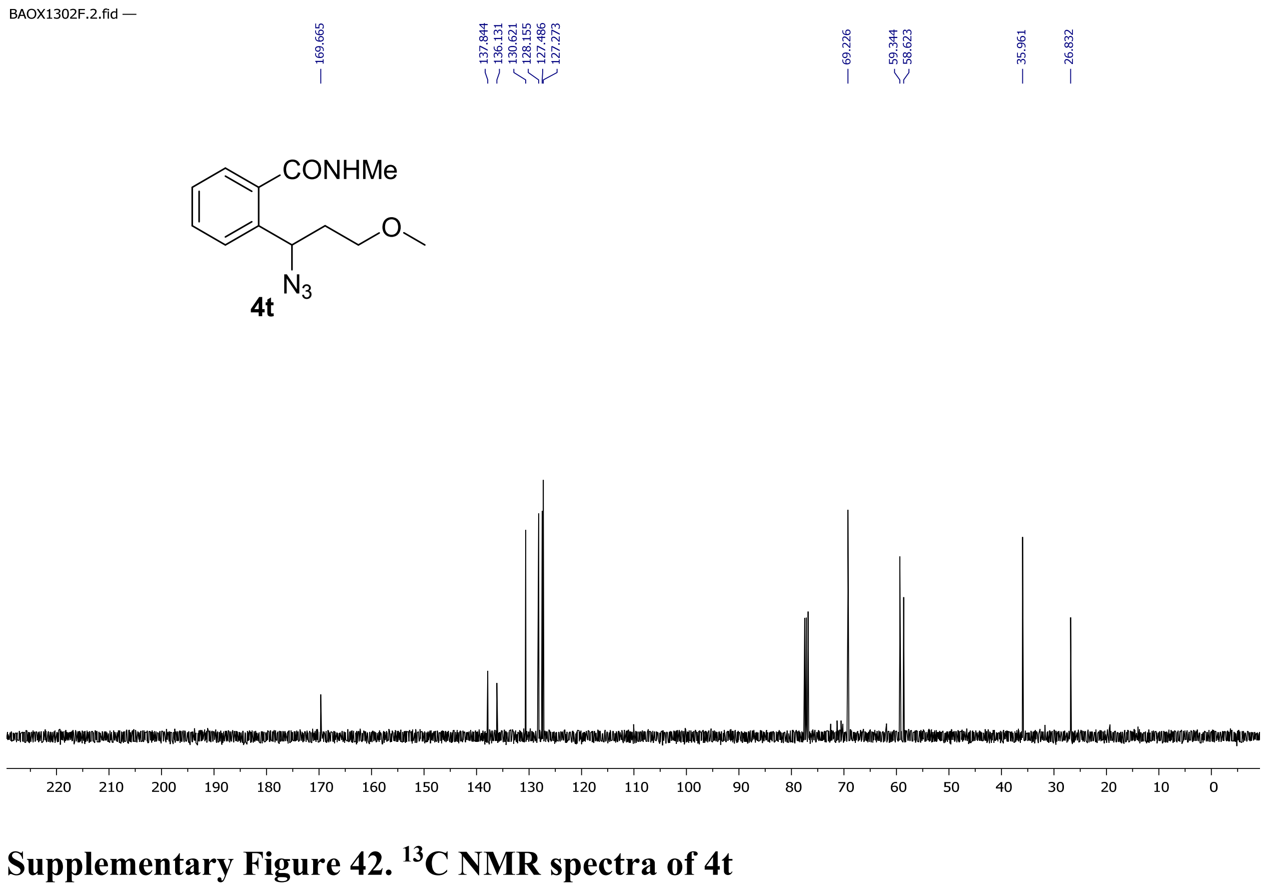

Supplementary Figure 42. ^{13}C NMR spectra of 4t

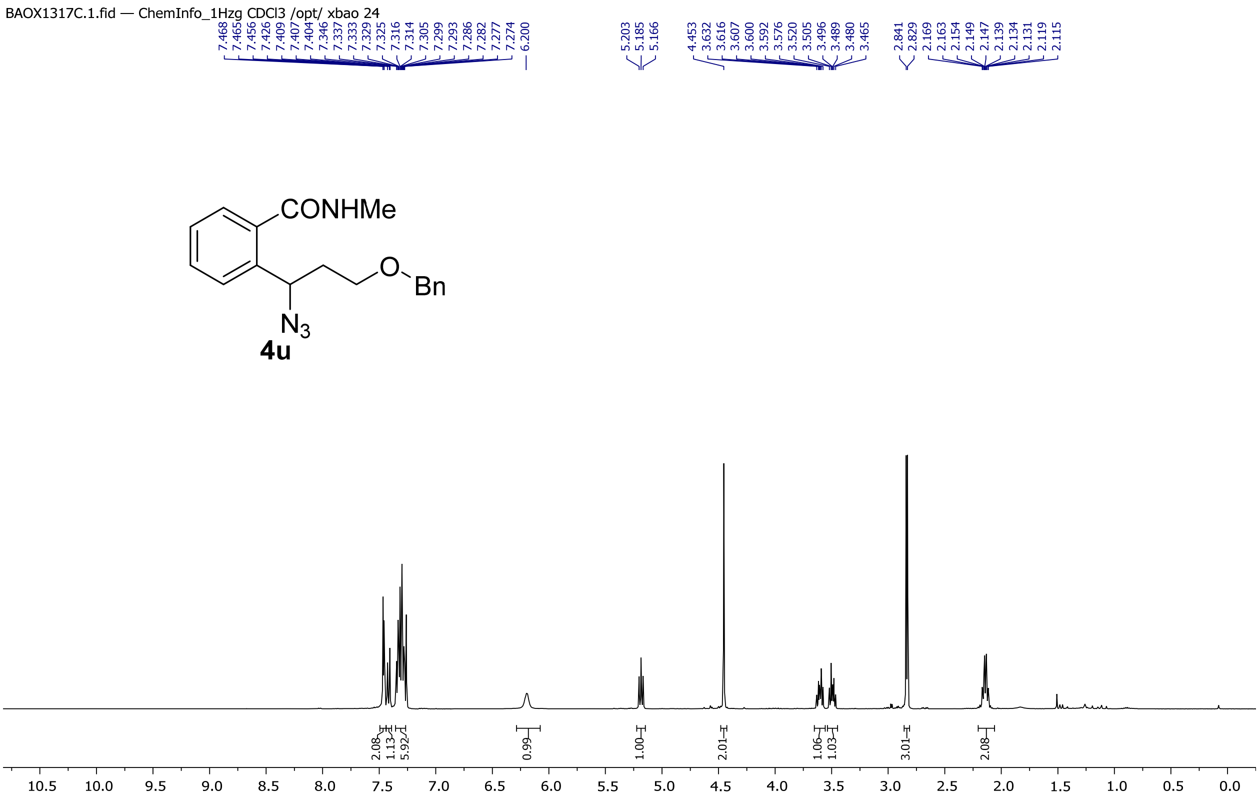

Supplementary Figure 43. ^{1}**H spectra of 4u**

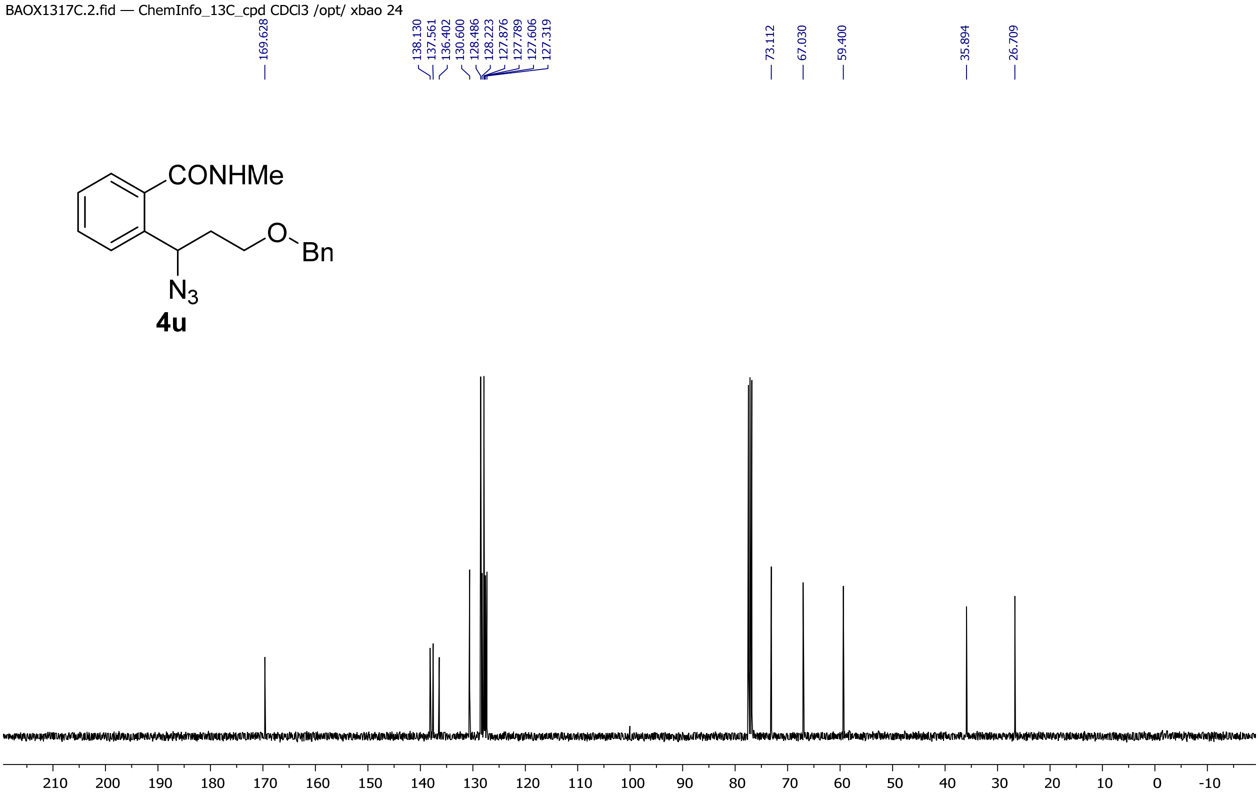

Supplementary Figure 44. ^{13}**C NMR spectra of 4u**

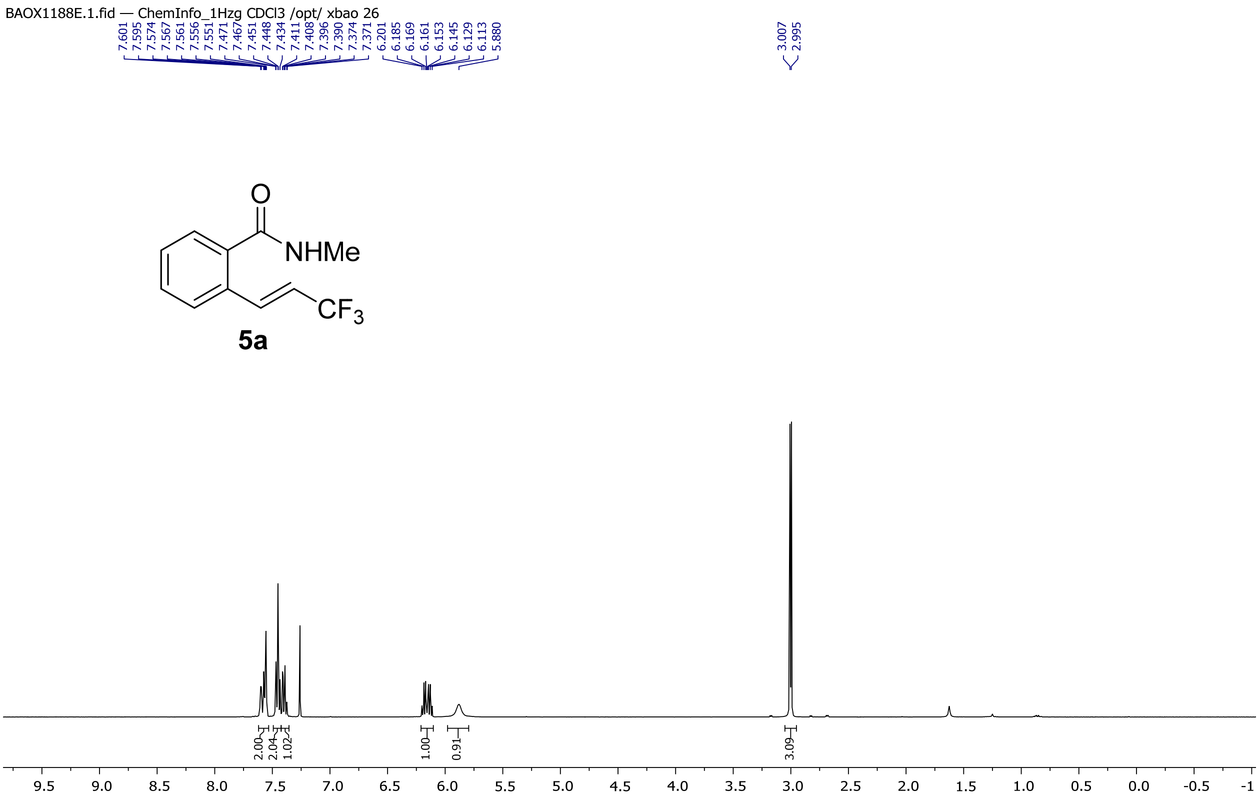

Supplementary Figure 45. ^{1}H spectra of 5a

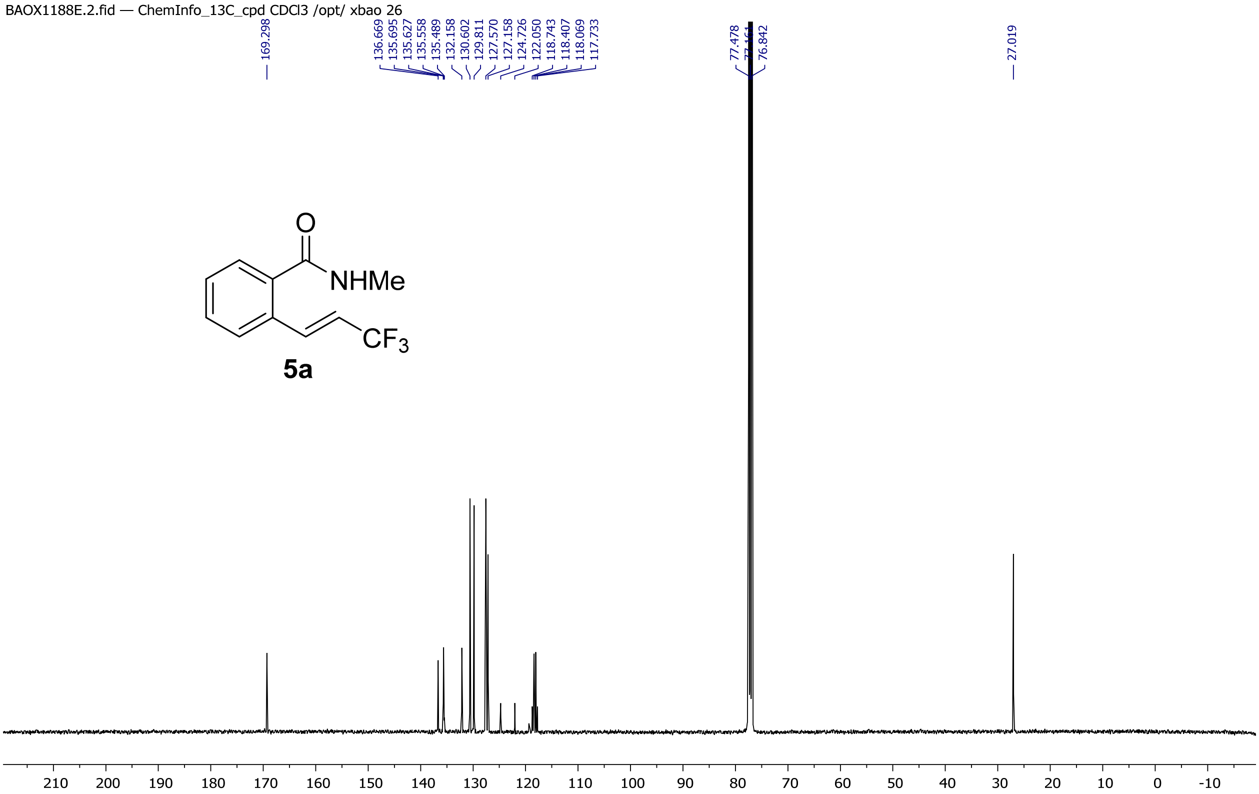

Supplementary Figure 46. ^{13}C NMR spectra of 5a

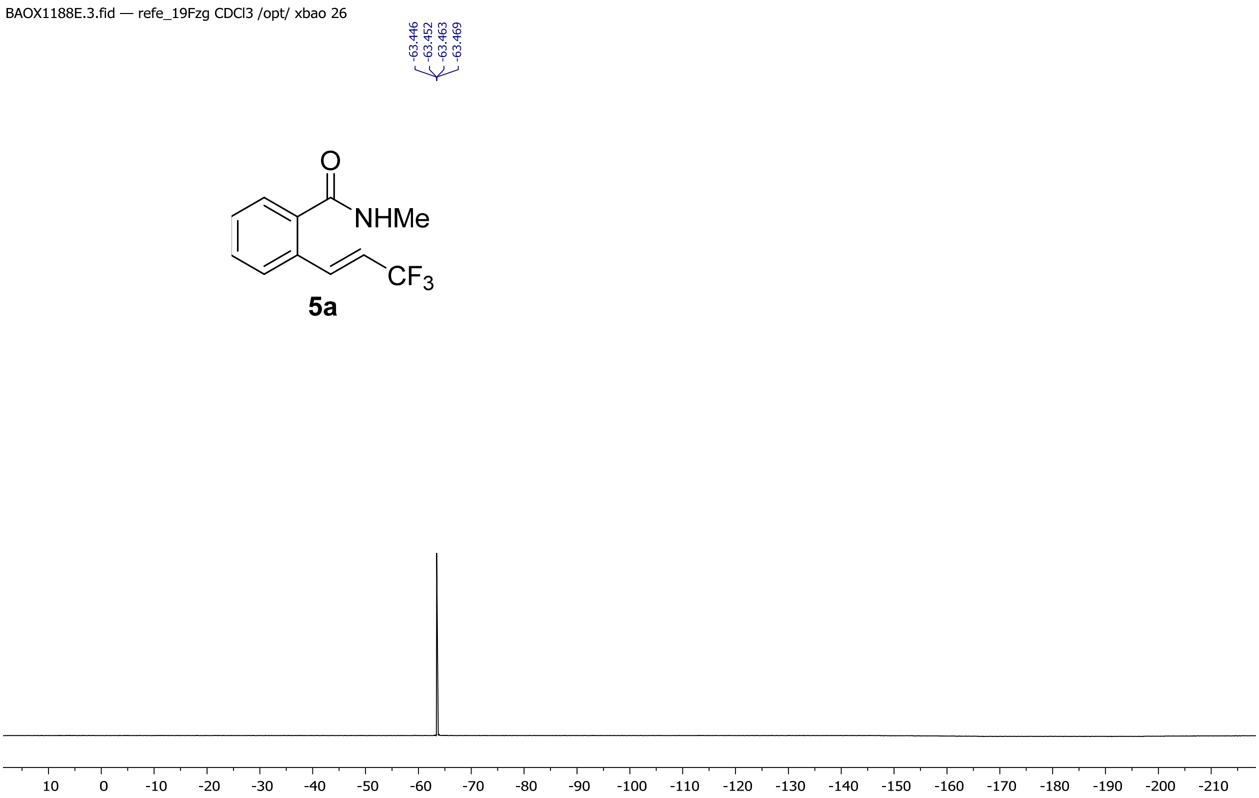

Supplementary Figure 47. ^{19}F spectra of 5a

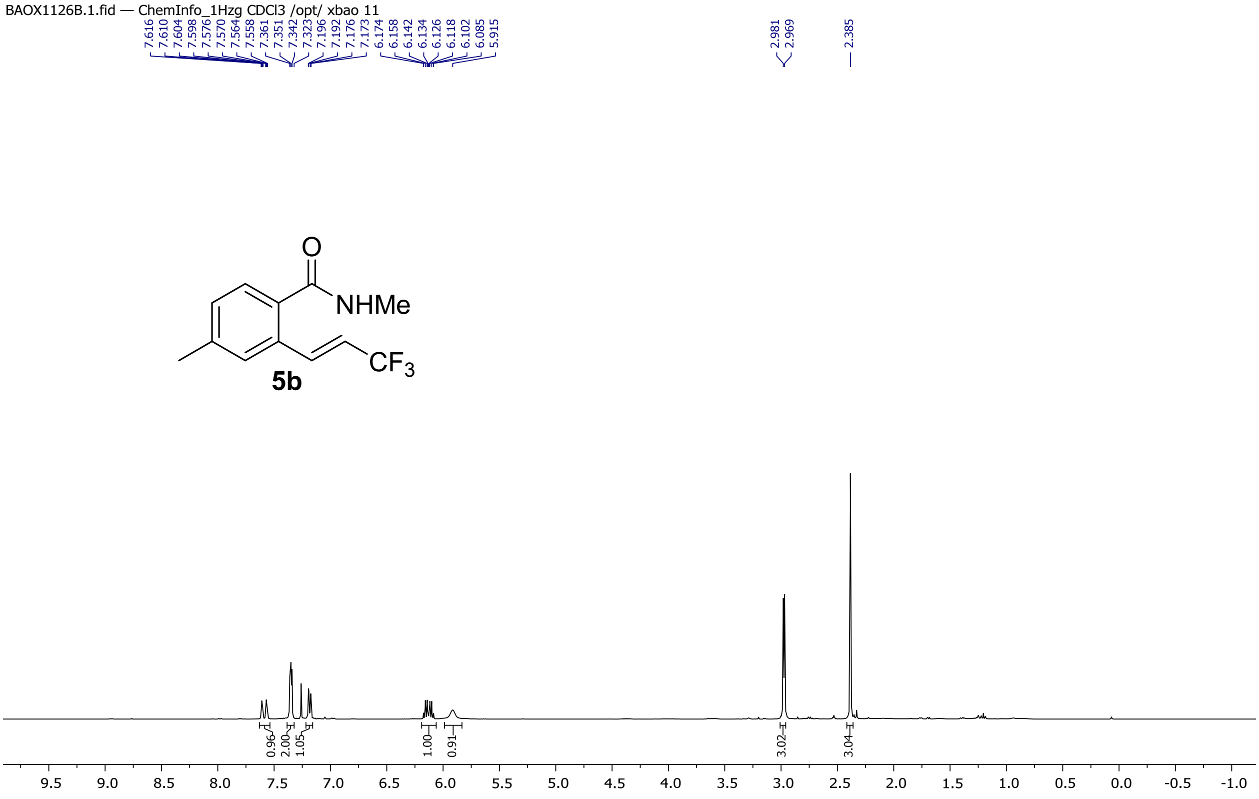

Supplementary Figure 48. ^{1}H spectra of 5b

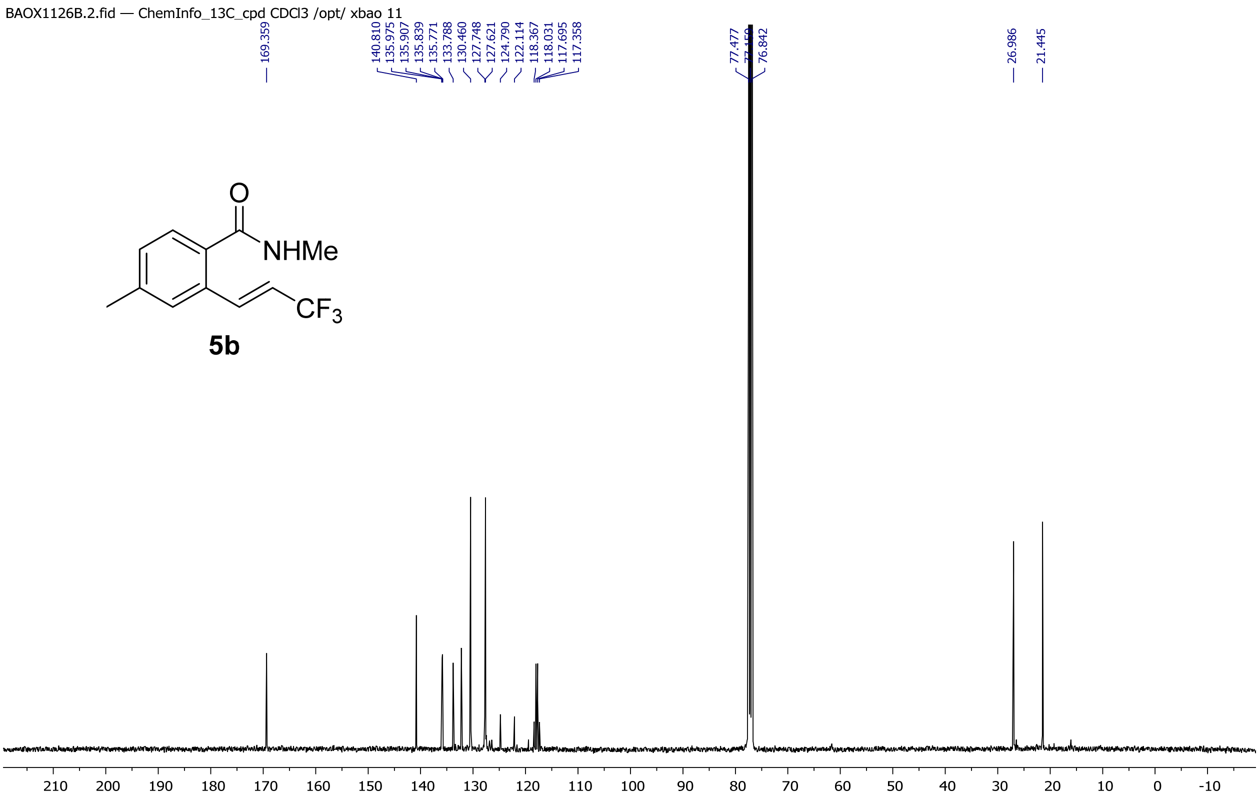

Supplementary Figure 49. ^{13}C NMR spectra of 5b

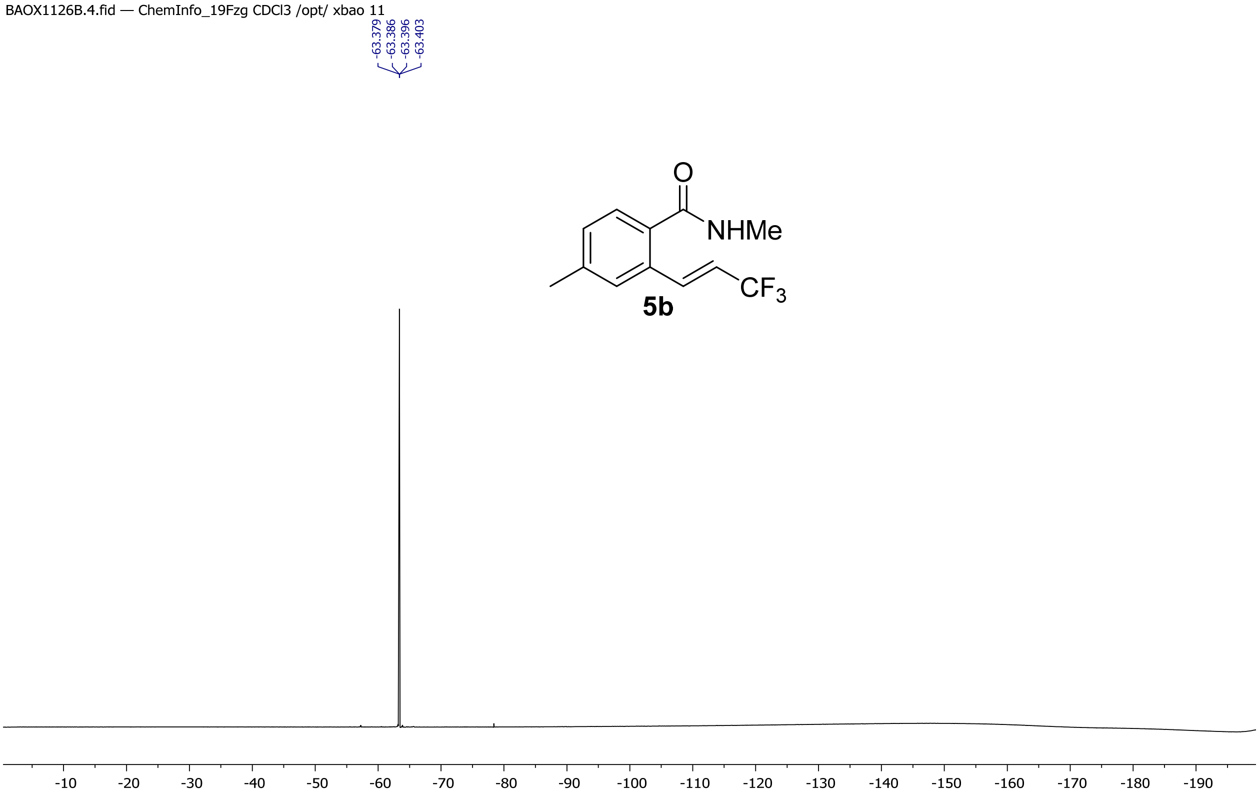

Supplementary Figure 50. ^{19}F spectra of 5b

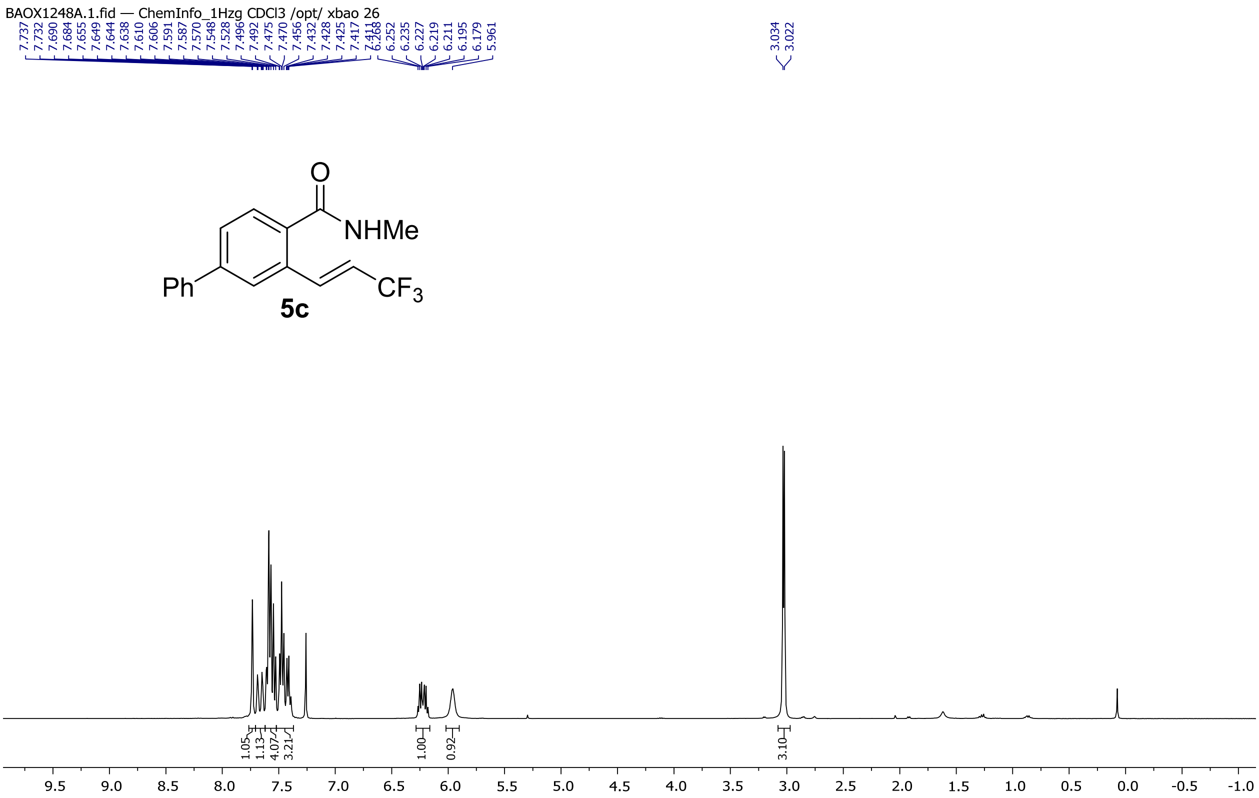

Supplementary Figure 51. ^{1}H spectra of 5c

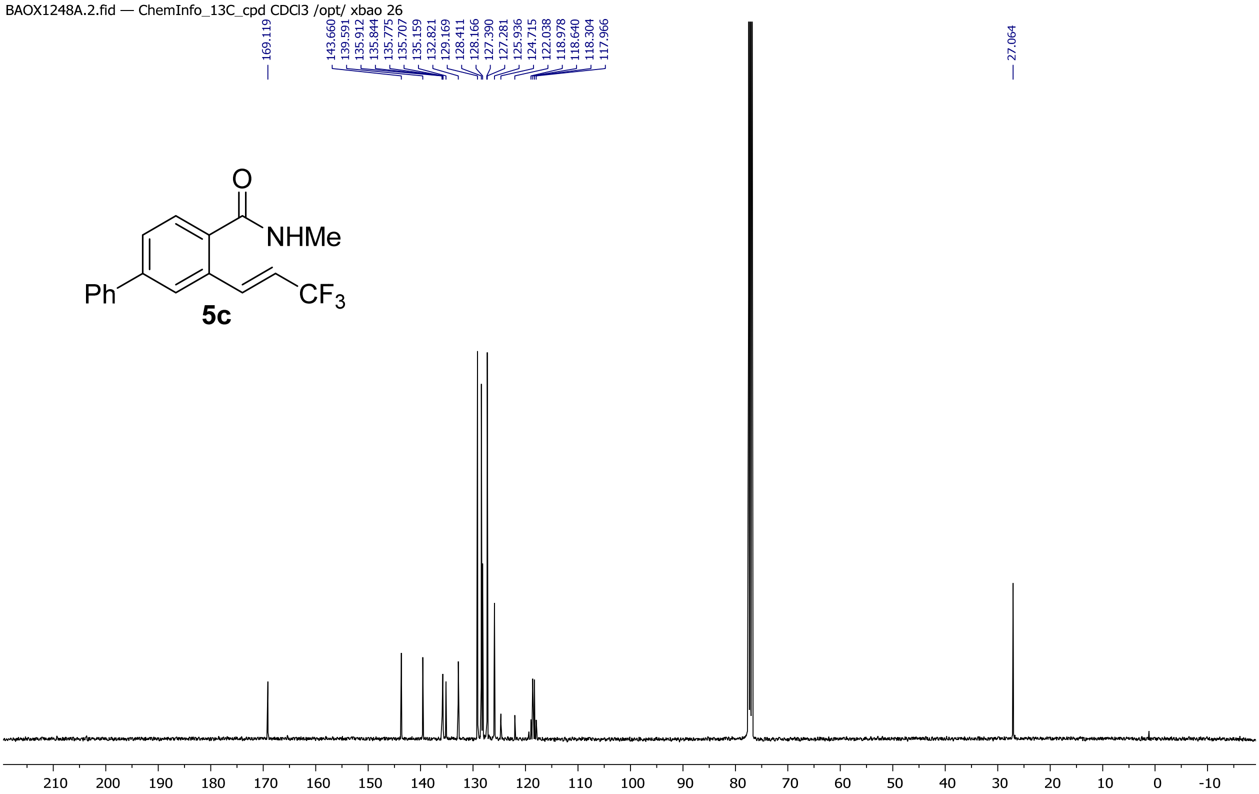

Supplementary Figure 52. ^{13}C NMR spectra of 5c

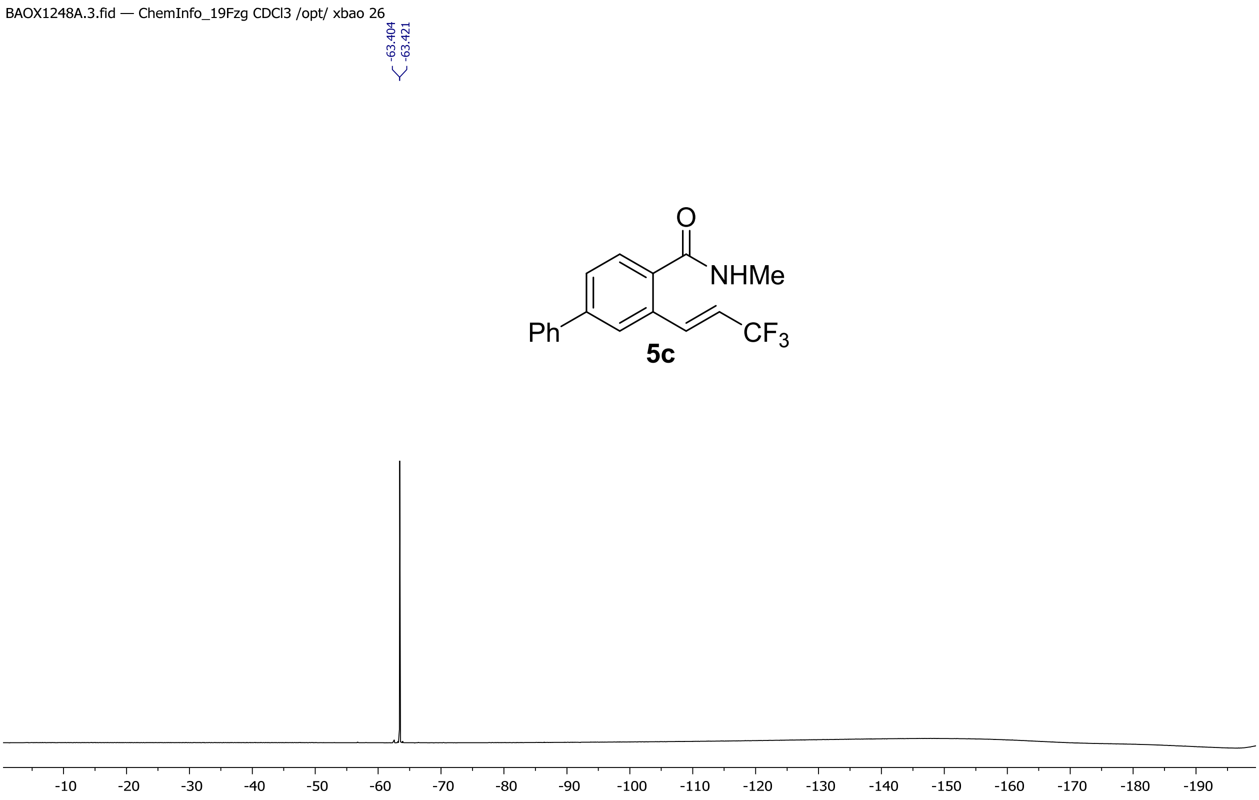

Supplementary Figure 53. ^{19}F spectra of 5c

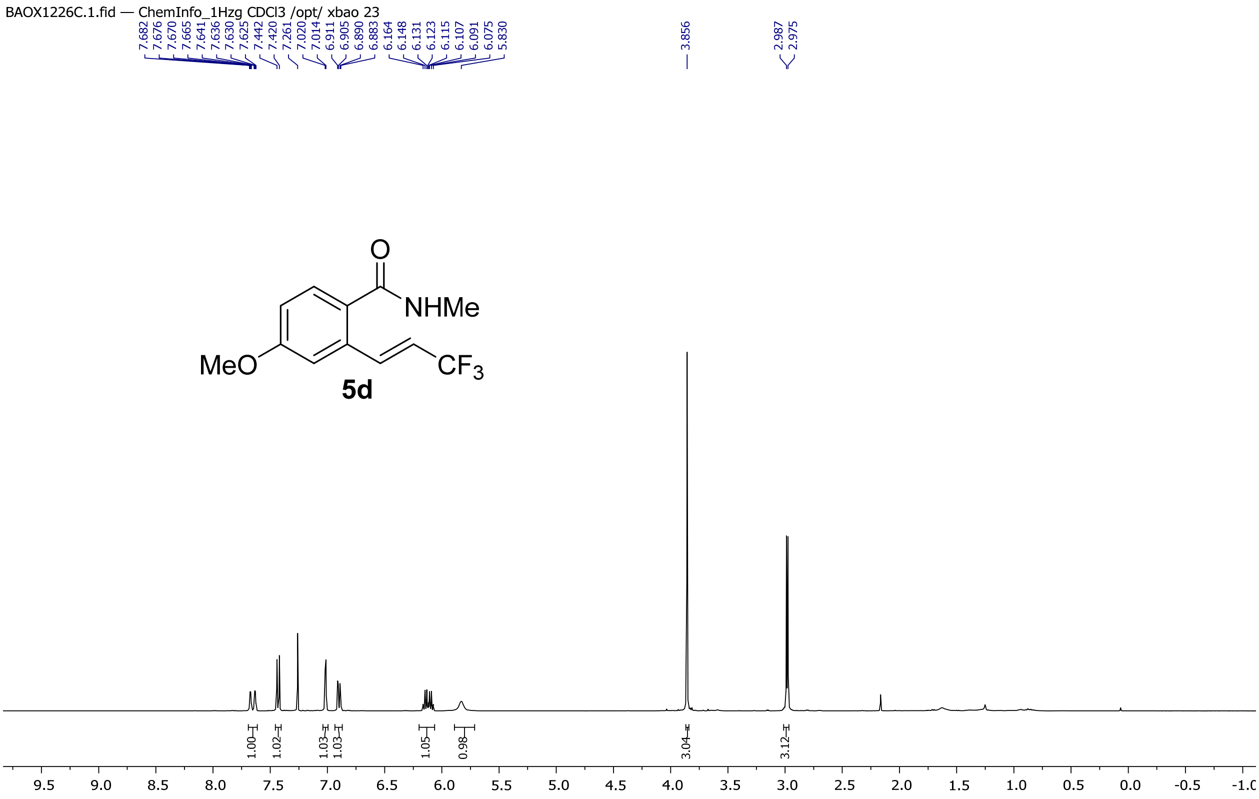

Supplementary Figure 54. ^{1}H spectra of 5d

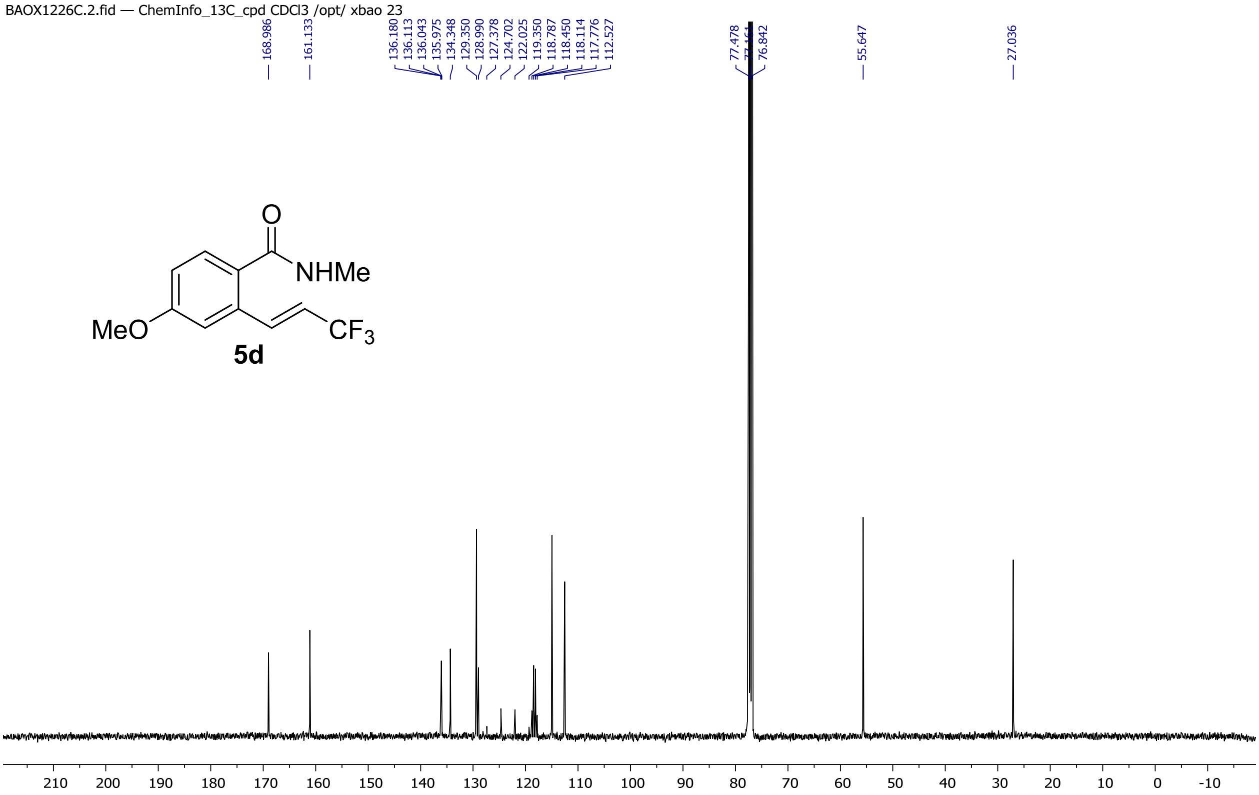

Supplementary Figure 55. ^{13}C NMR spectra of 5d

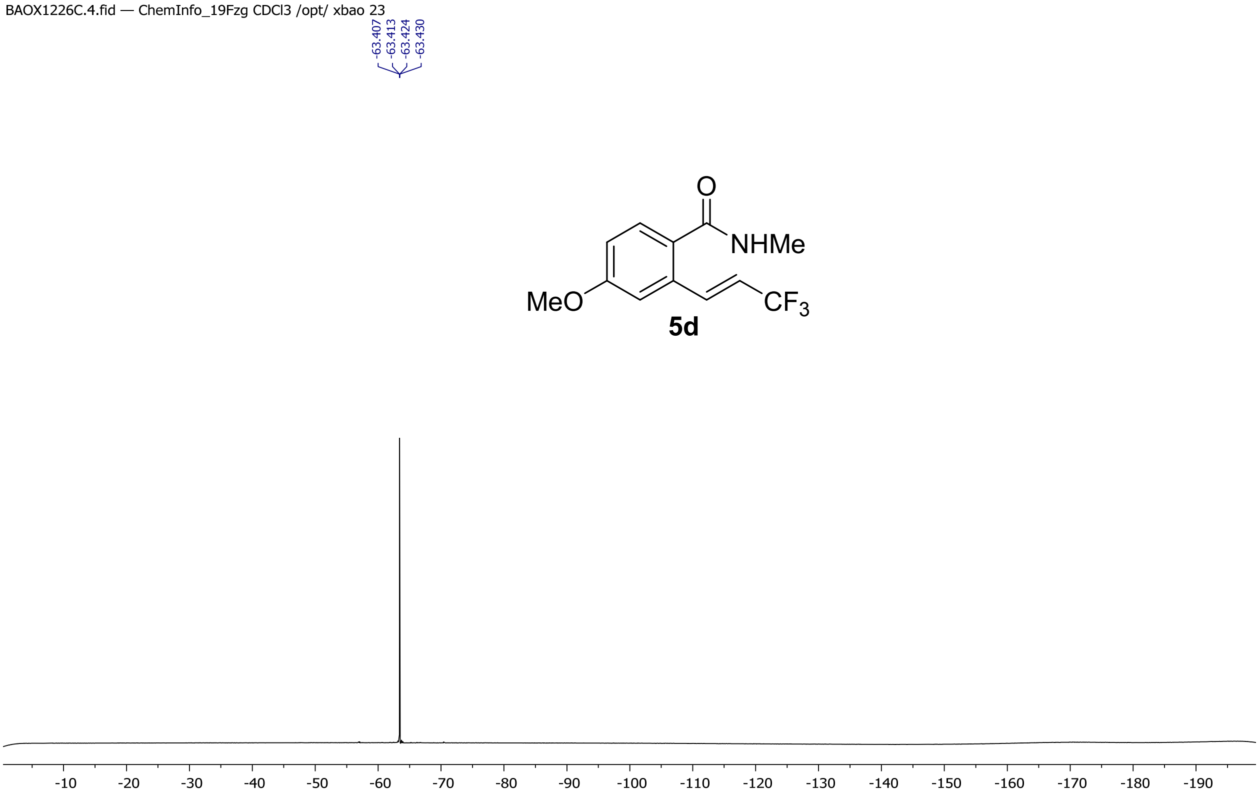

Supplementary Figure 56. ^{19}F spectra of 5d

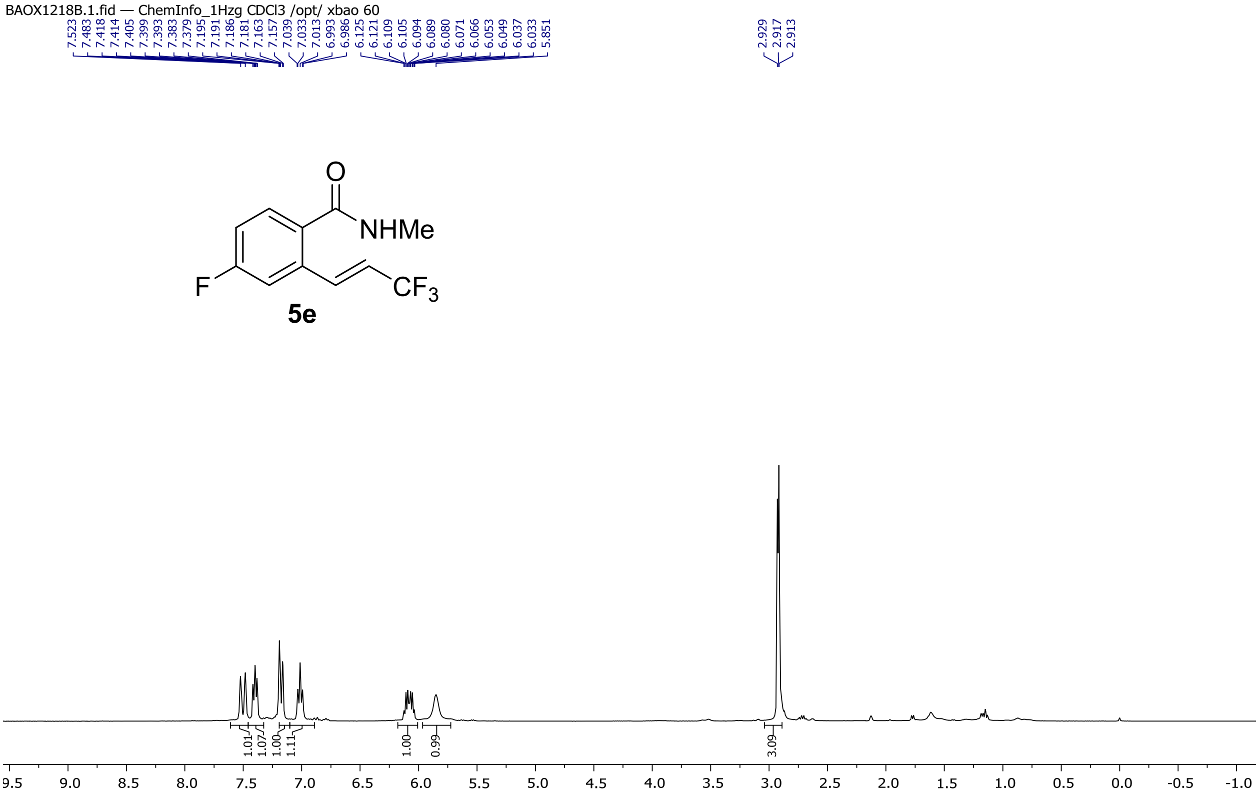

Supplementary Figure 57. ^{1}H spectra of 5e

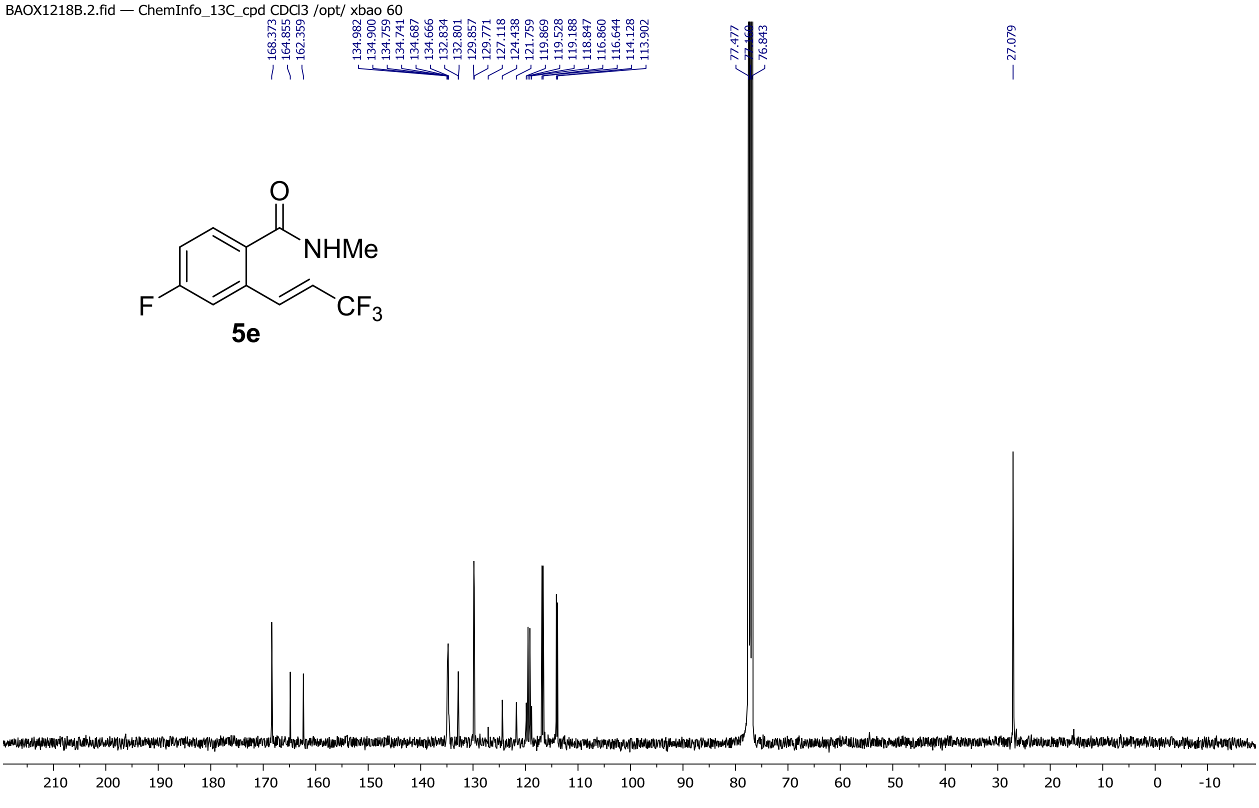

Supplementary Figure 58. ^{13}C NMR spectra of 5e

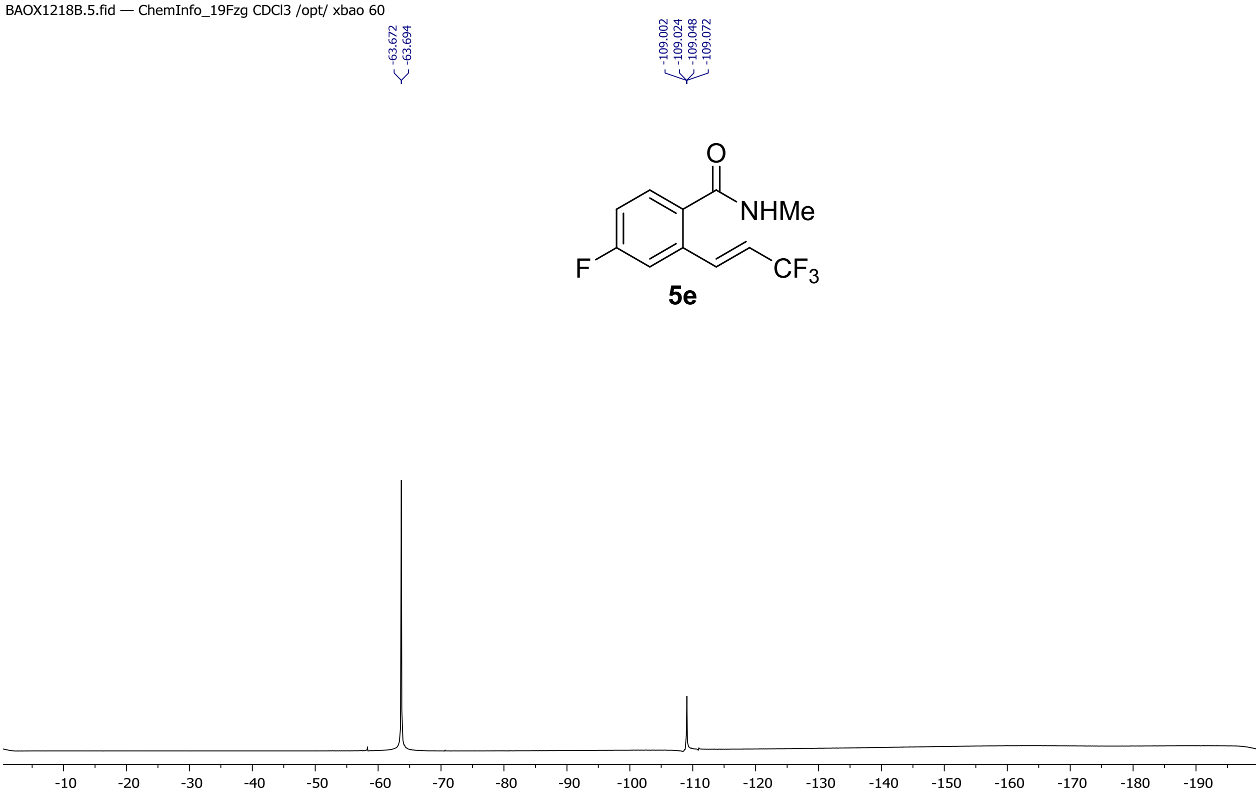

Supplementary Figure 59. ^{19}F spectra of 5e

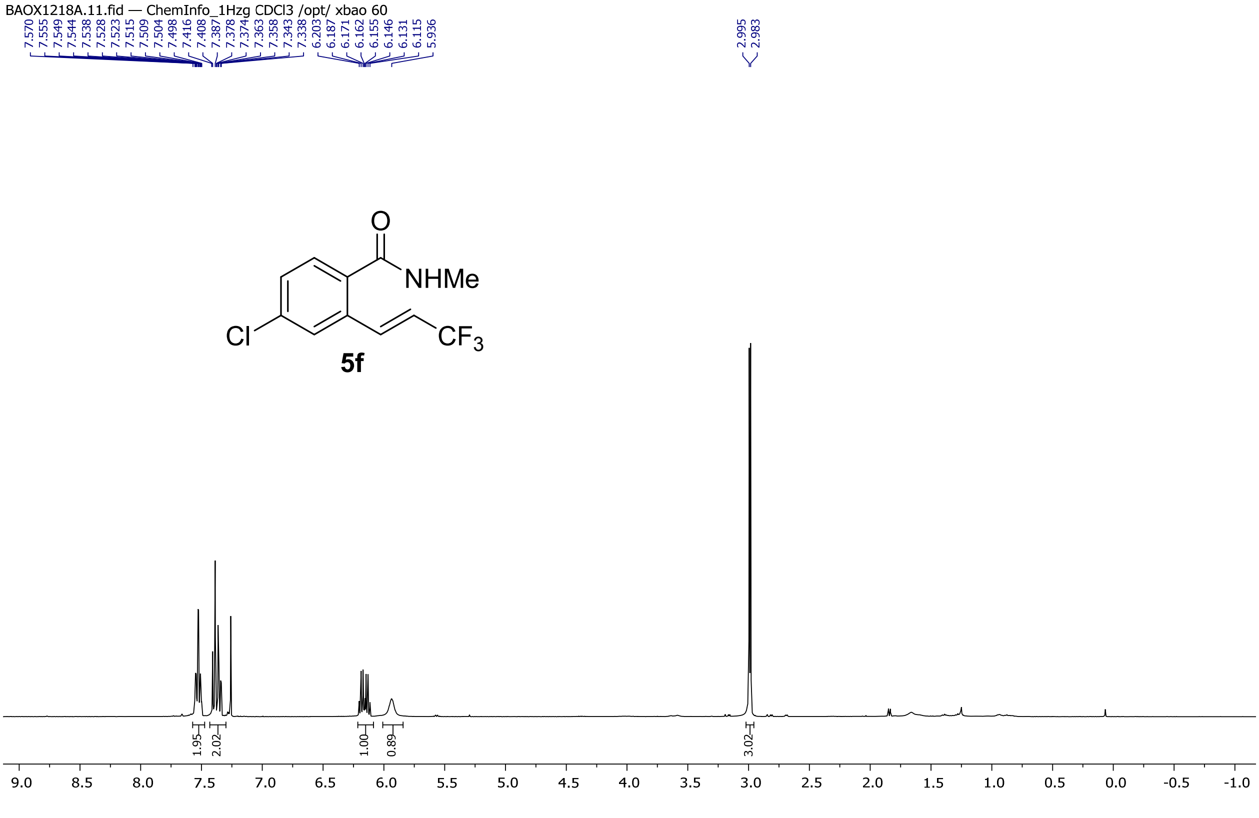

Supplementary Figure 60. ^{1}H spectra of 5f

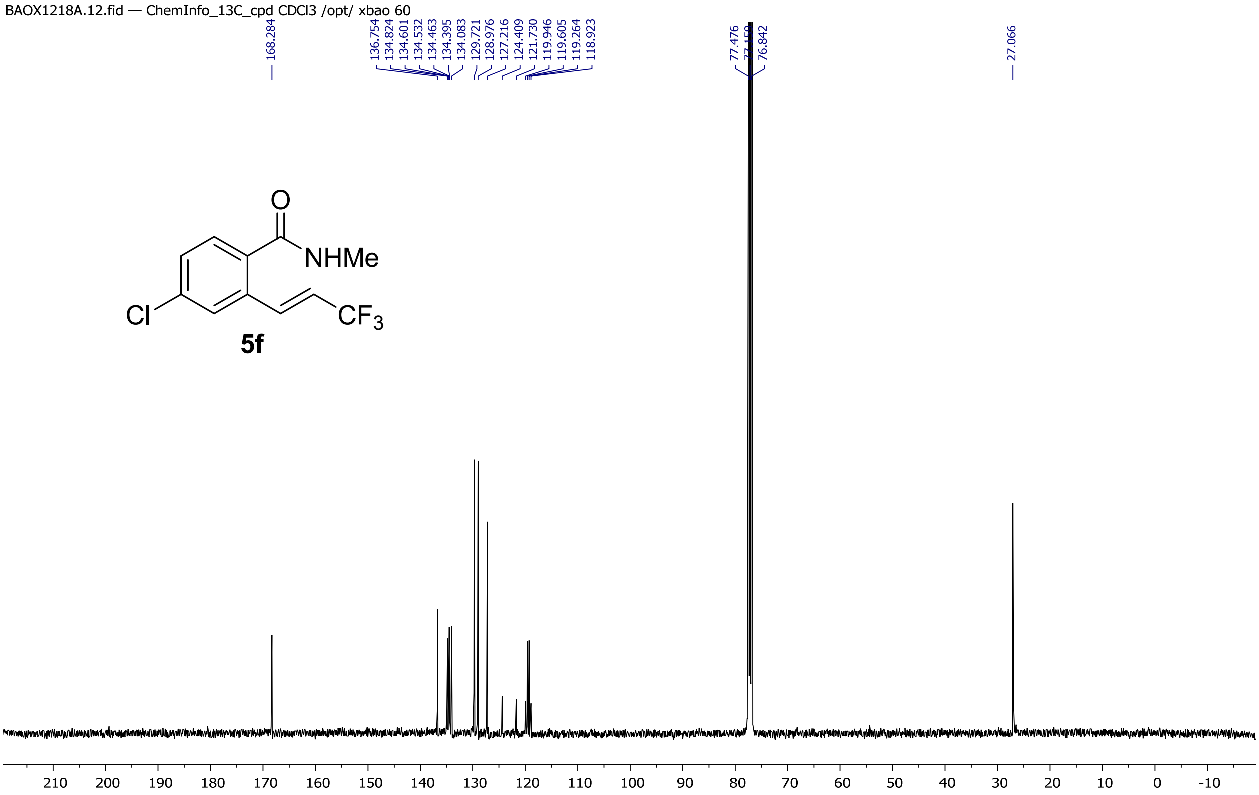

Supplementary Figure 61. ^{13}C NMR spectra of 5f

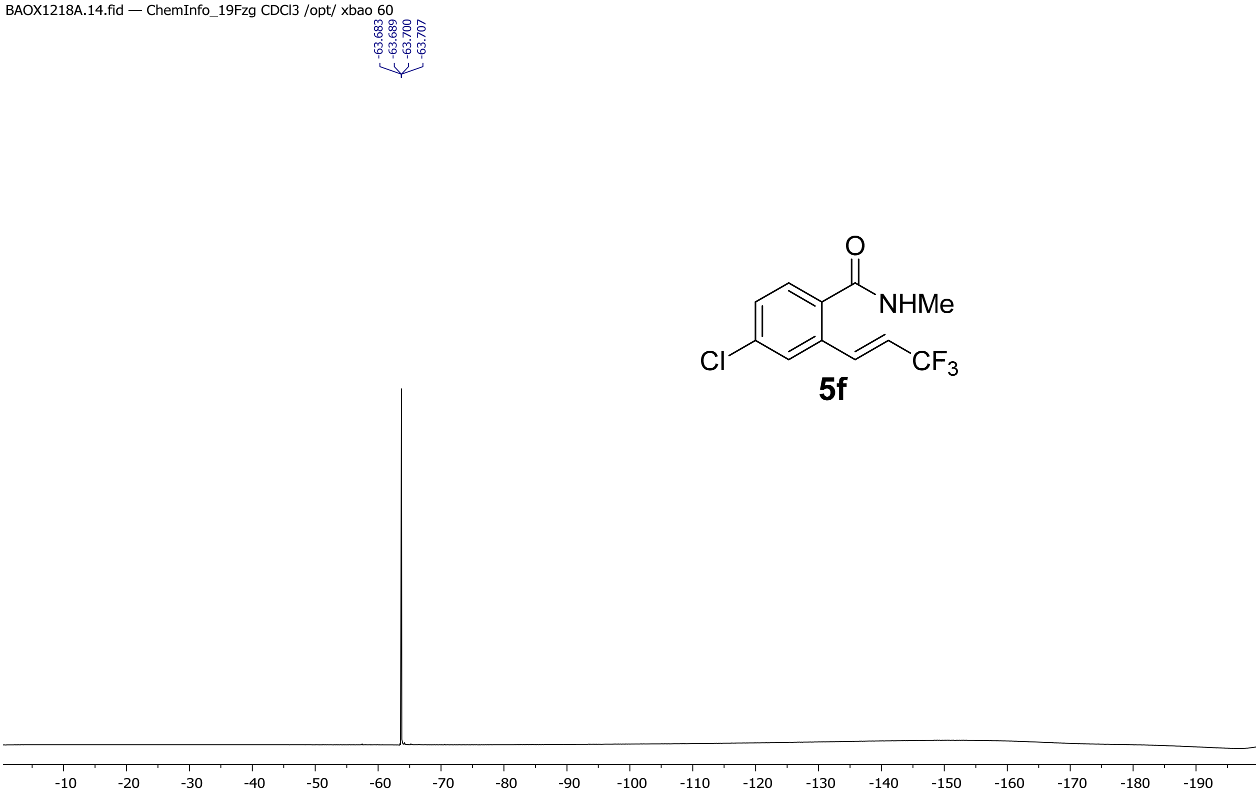

Supplementary Figure 62. ^{19}F spectra of 5f

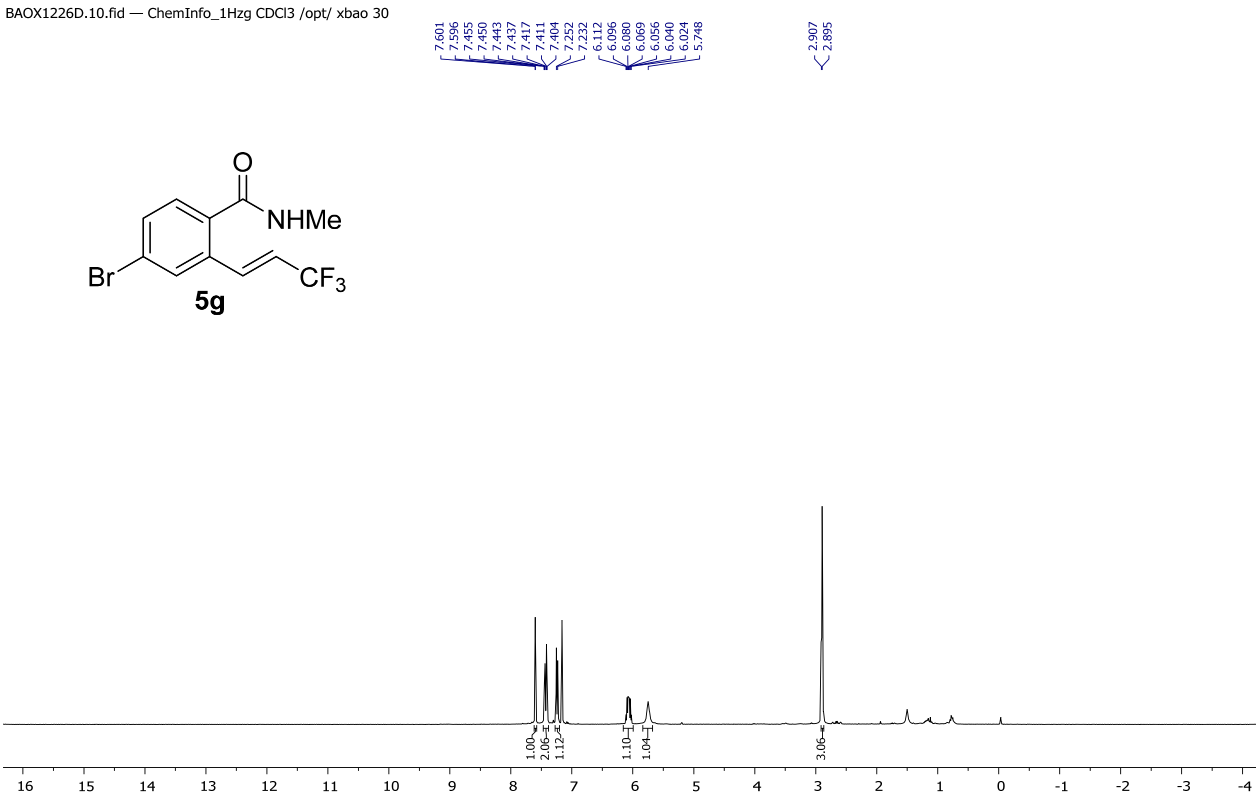

Supplementary Figure 63. ^{1}H spectra of 5g

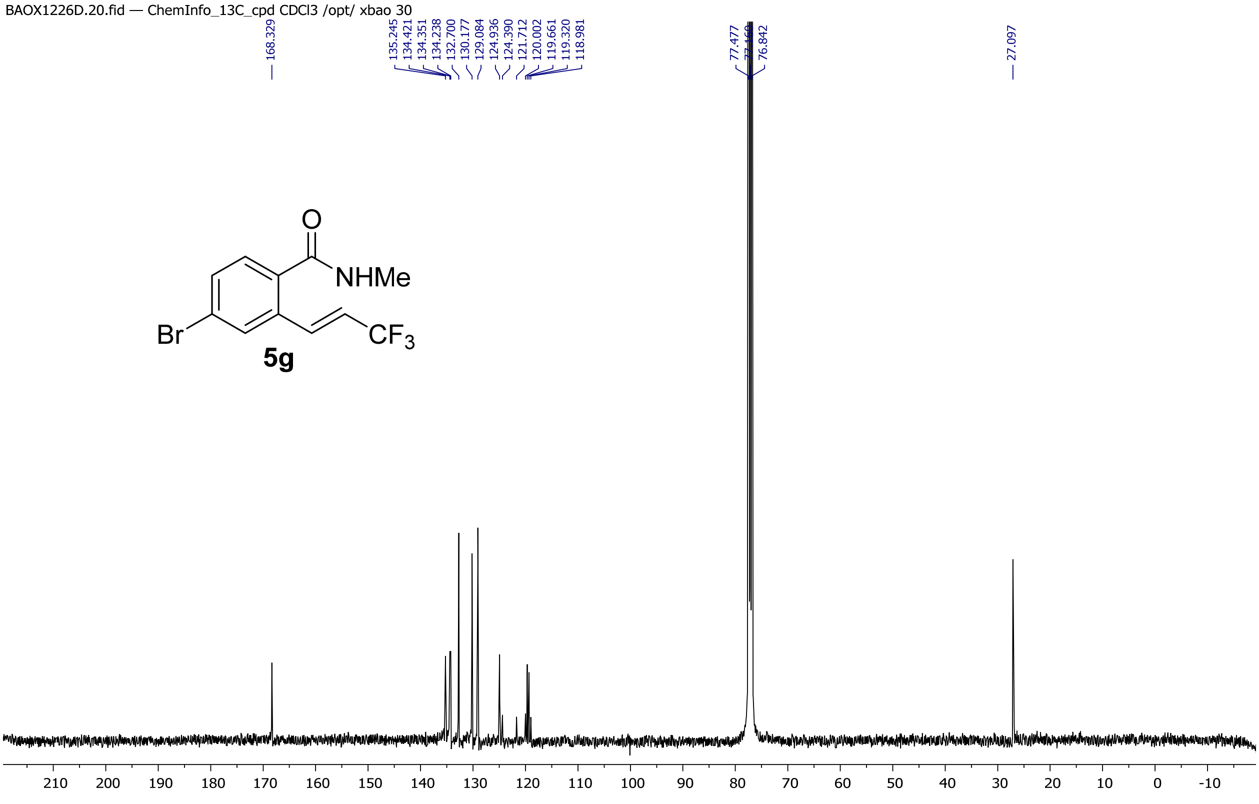

Supplementary Figure 64. ^{13}C NMR spectra of 5g

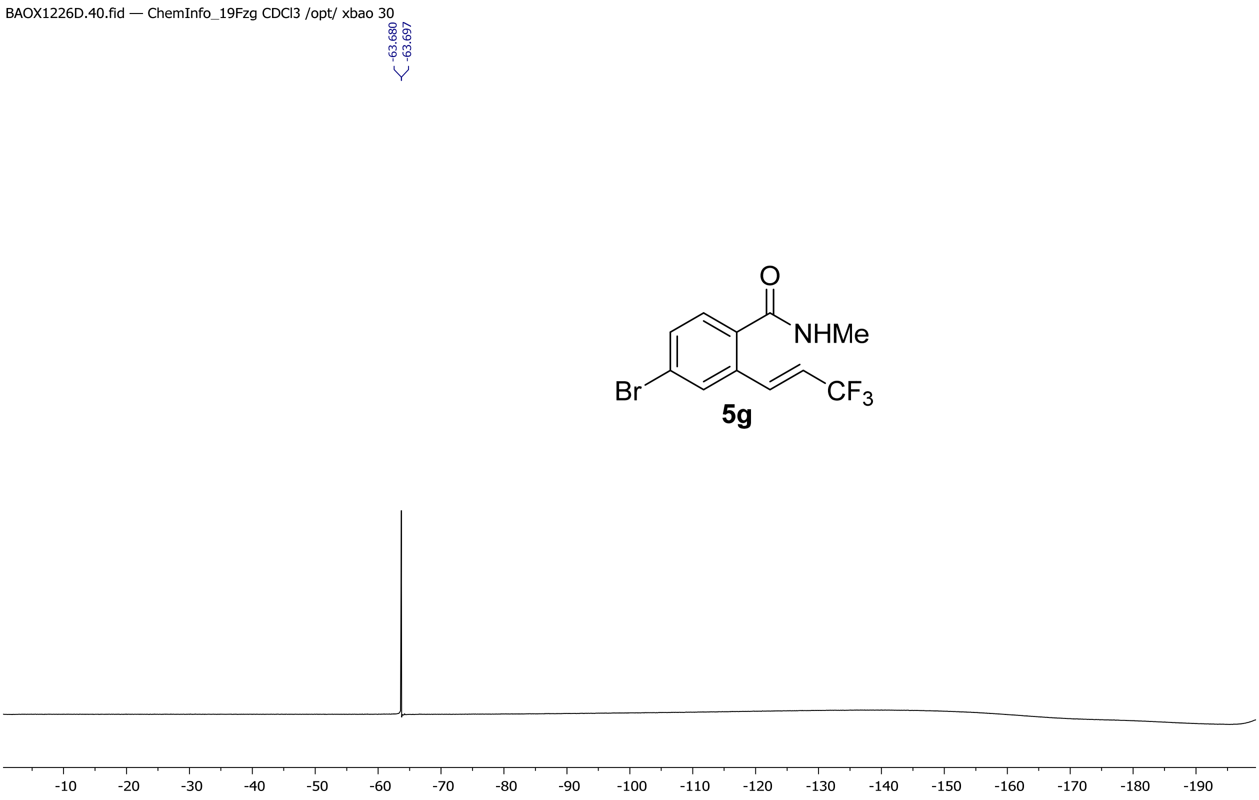

Supplementary Figure 65. ^{19}F spectra of 5g

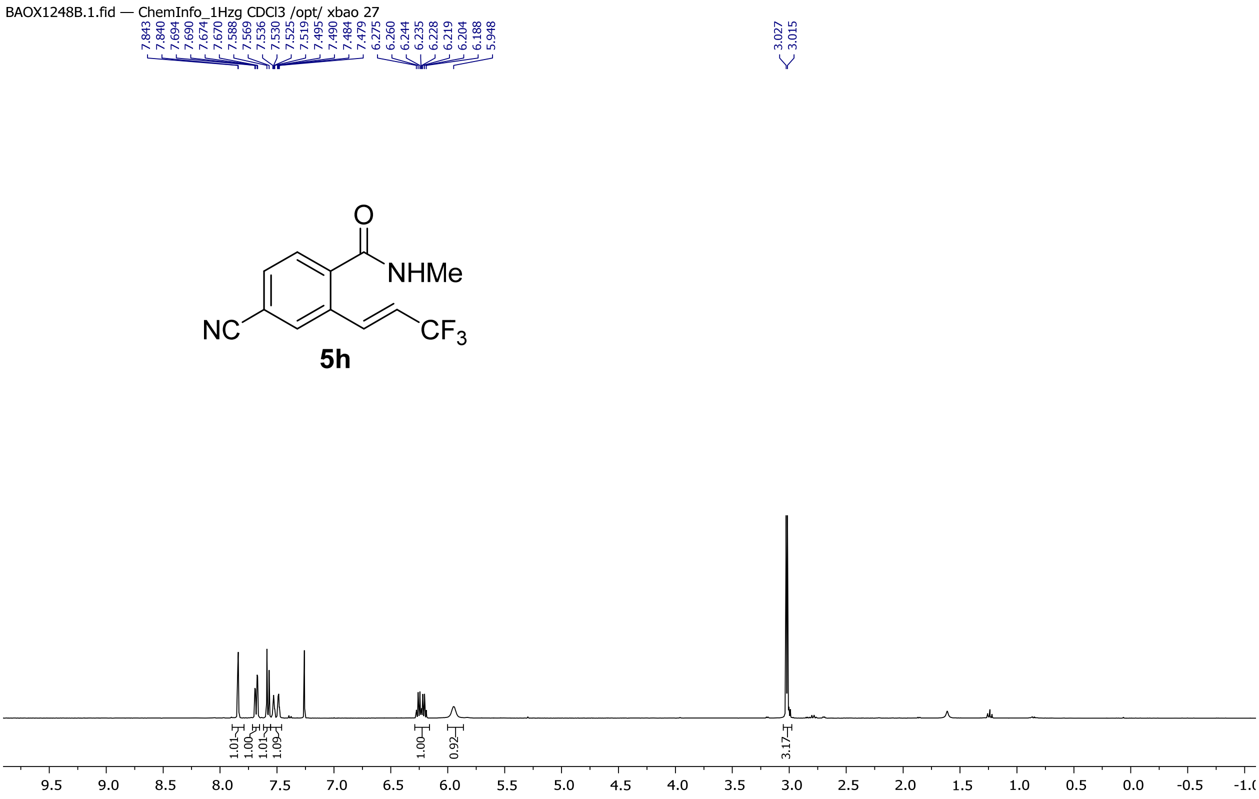

Supplementary Figure 66. ^{1}H spectra of 5h

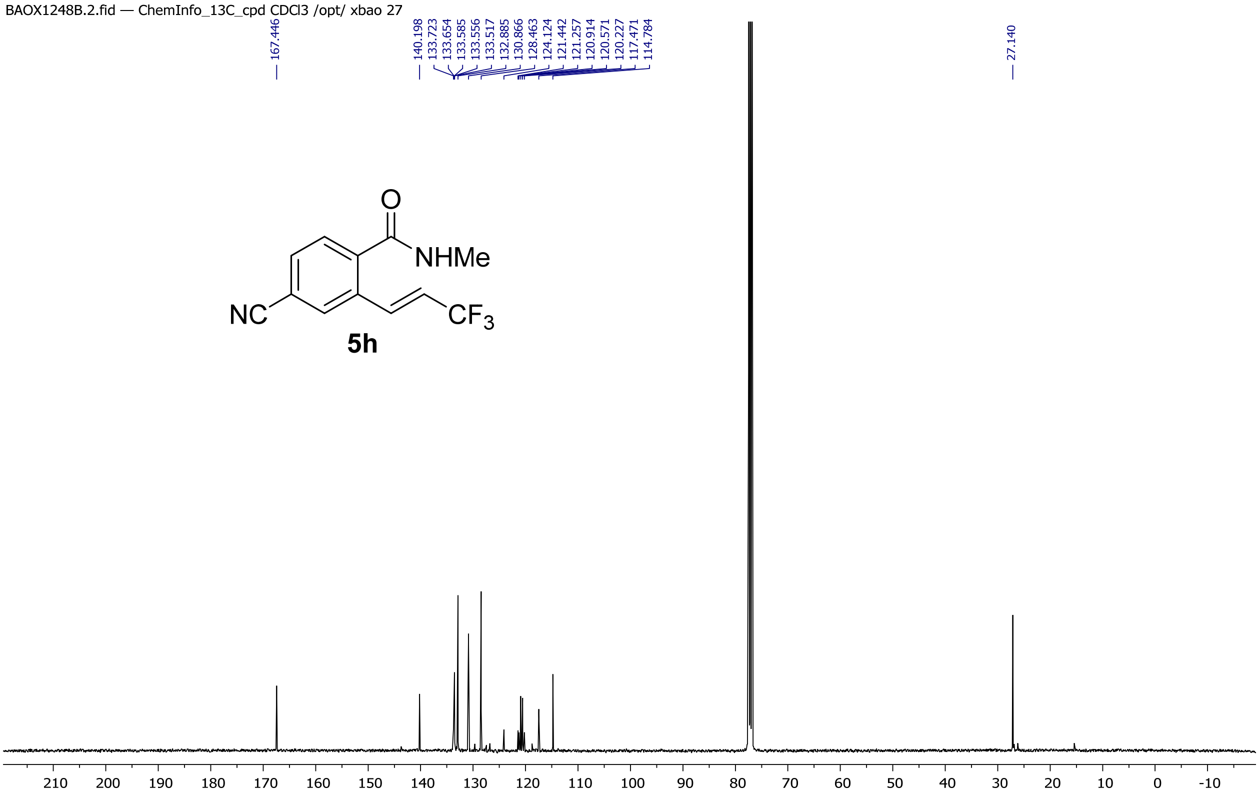

Supplementary Figure 67. ^{13}C NMR spectra of 5h

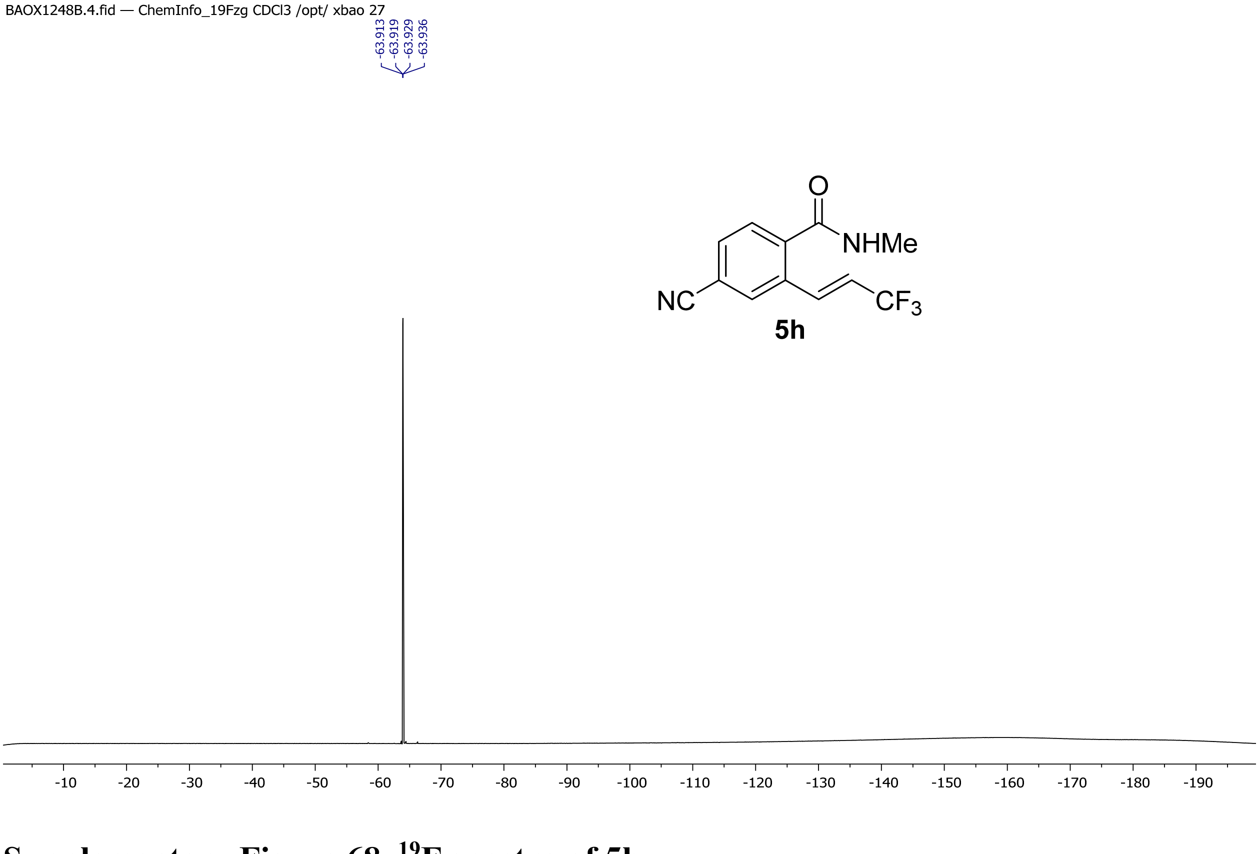

Supplementary Figure 68. ^{19}F spectra of 5h

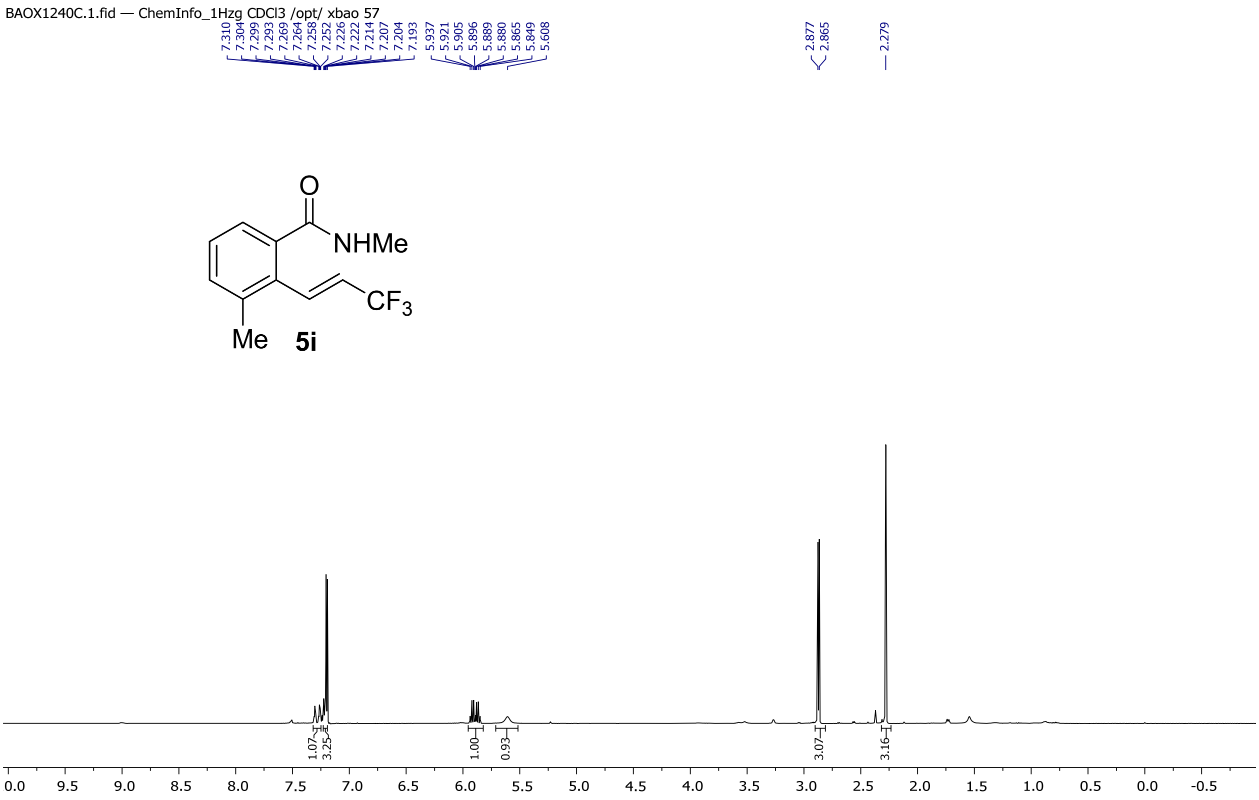

Supplementary Figure 69. ^{1}H spectra of 5i

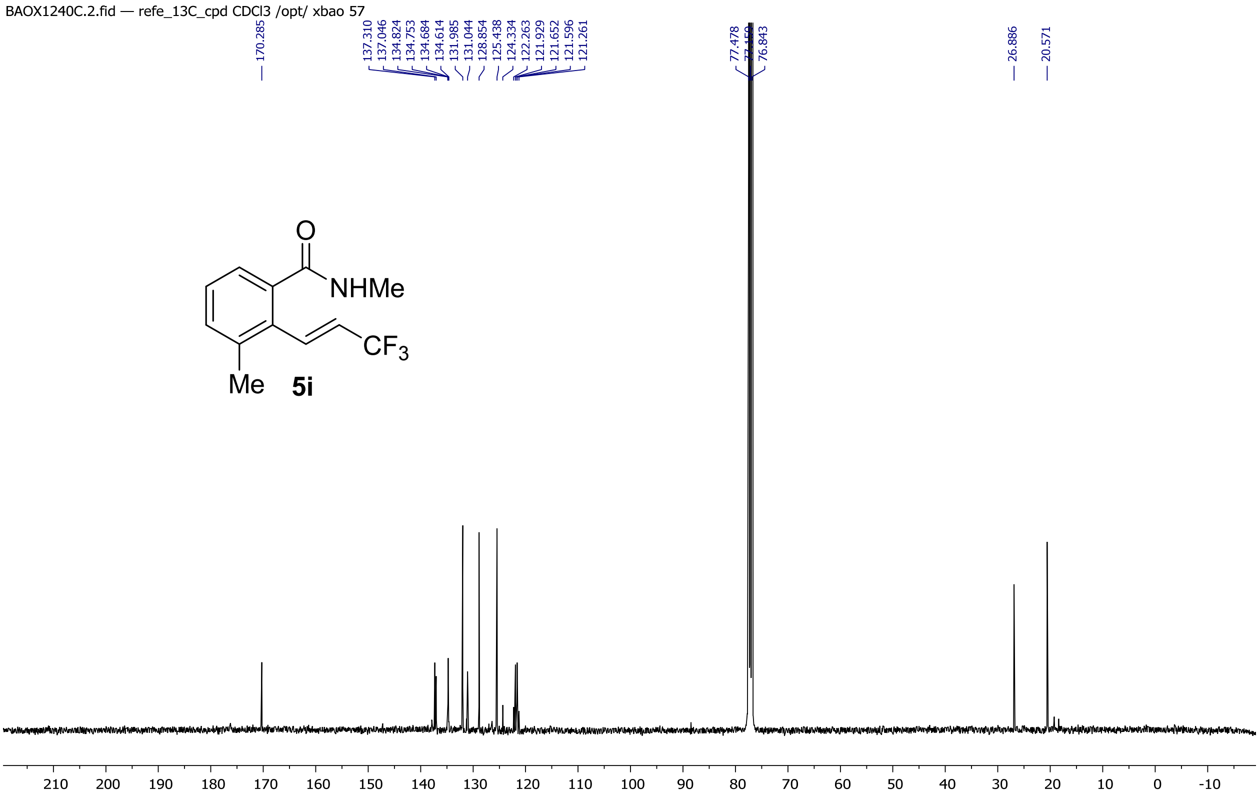

Supplementary Figure 70. ^{13}C NMR spectra of 5i

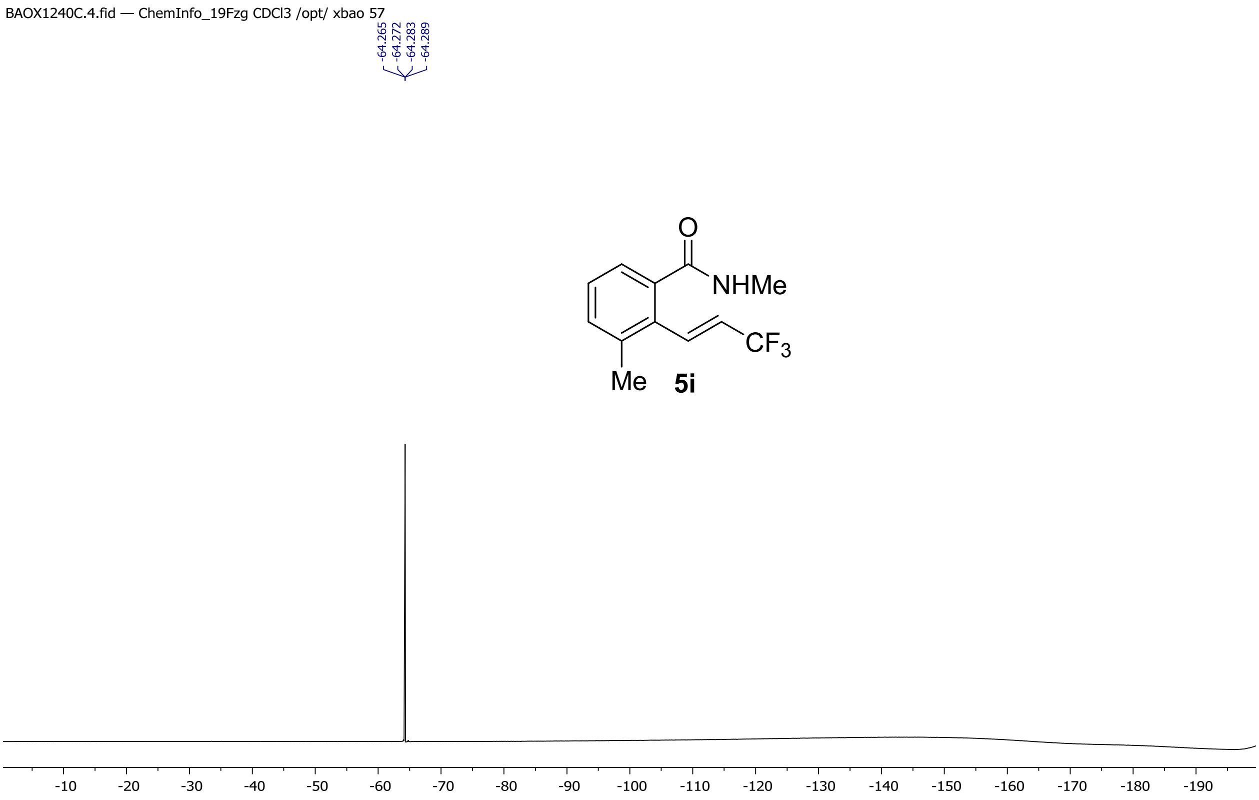

Supplementary Figure 71. ^{19}F spectra of 5i

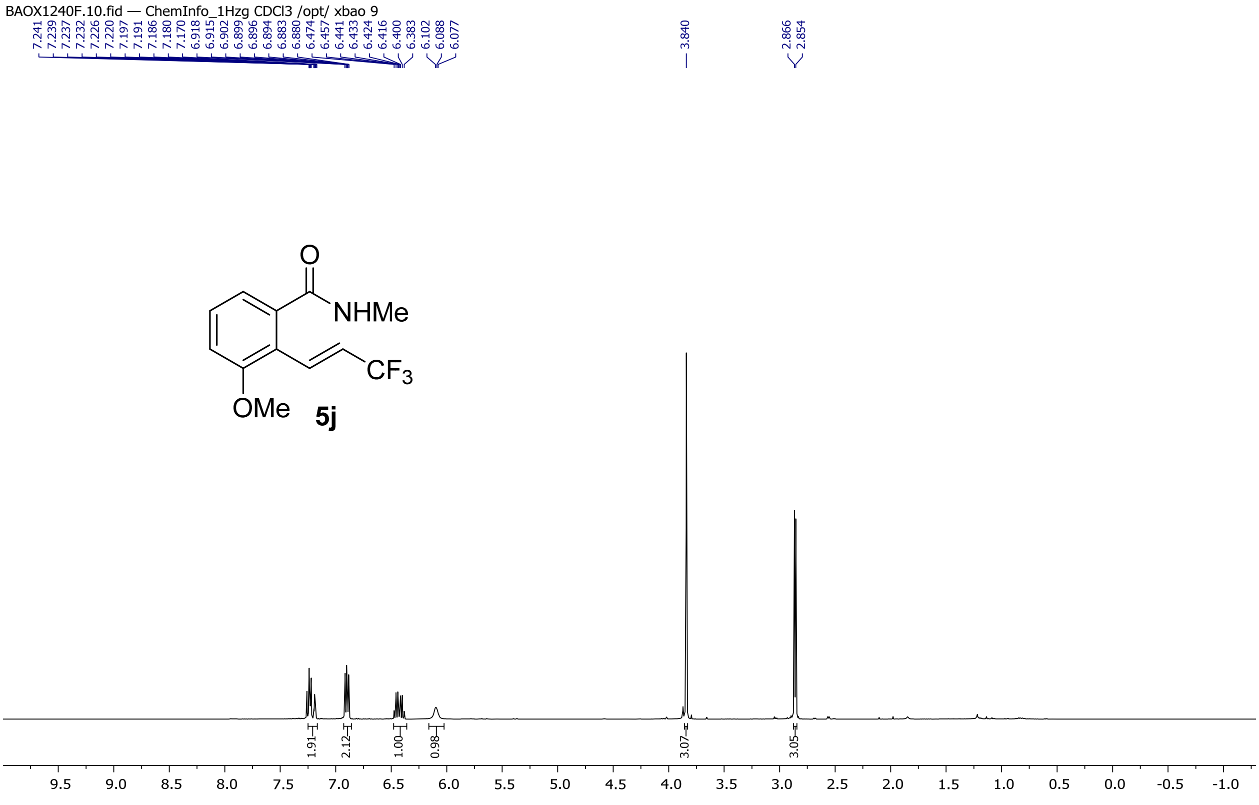

Supplementary Figure 72. ^{1}H spectra of 5j

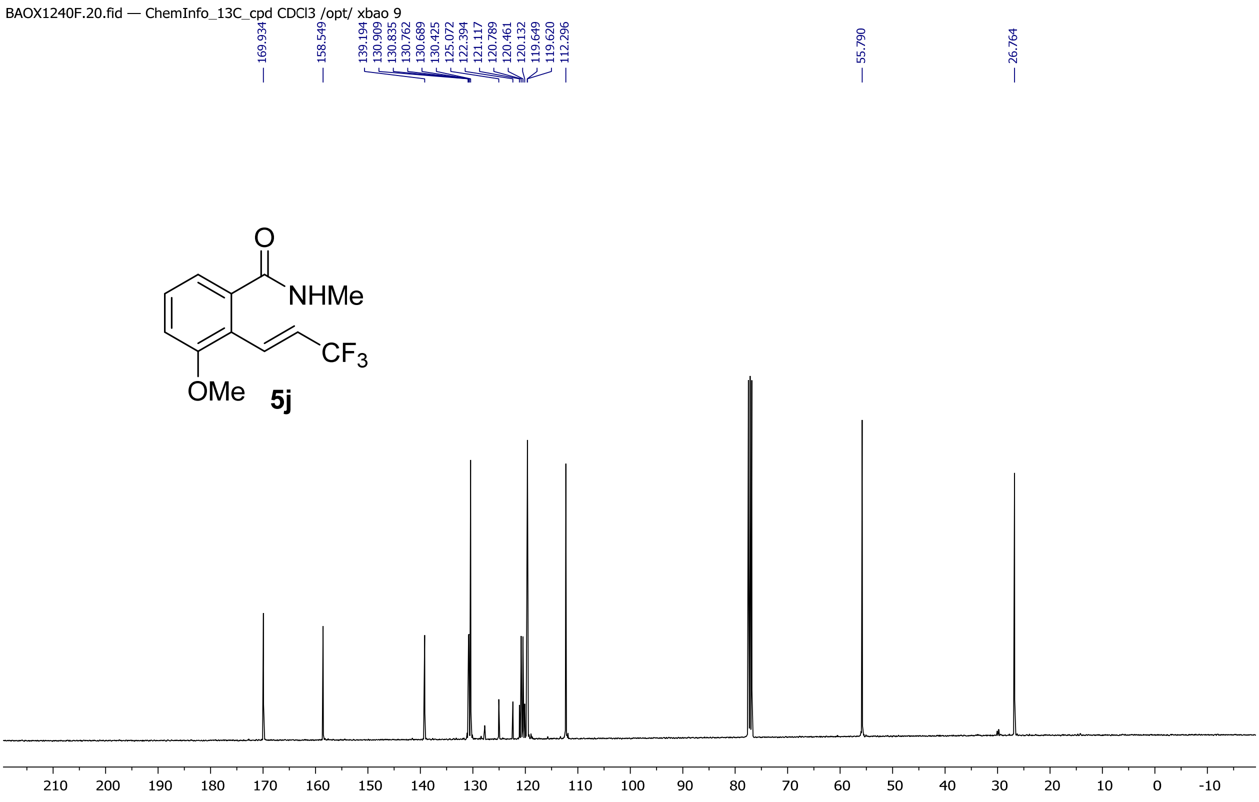

Supplementary Figure 73. ^{13}C NMR spectra of 5j

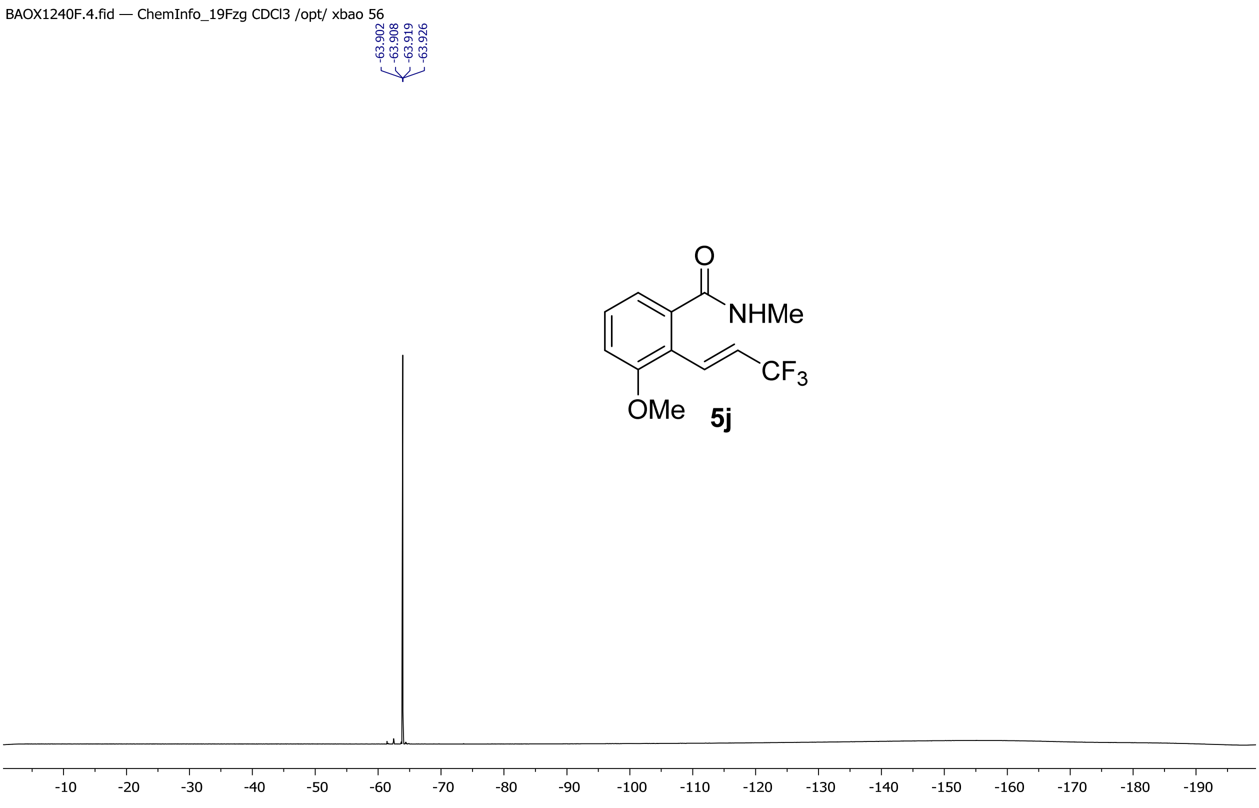

Supplementary Figure 74. ^{19}F spectra of 5j

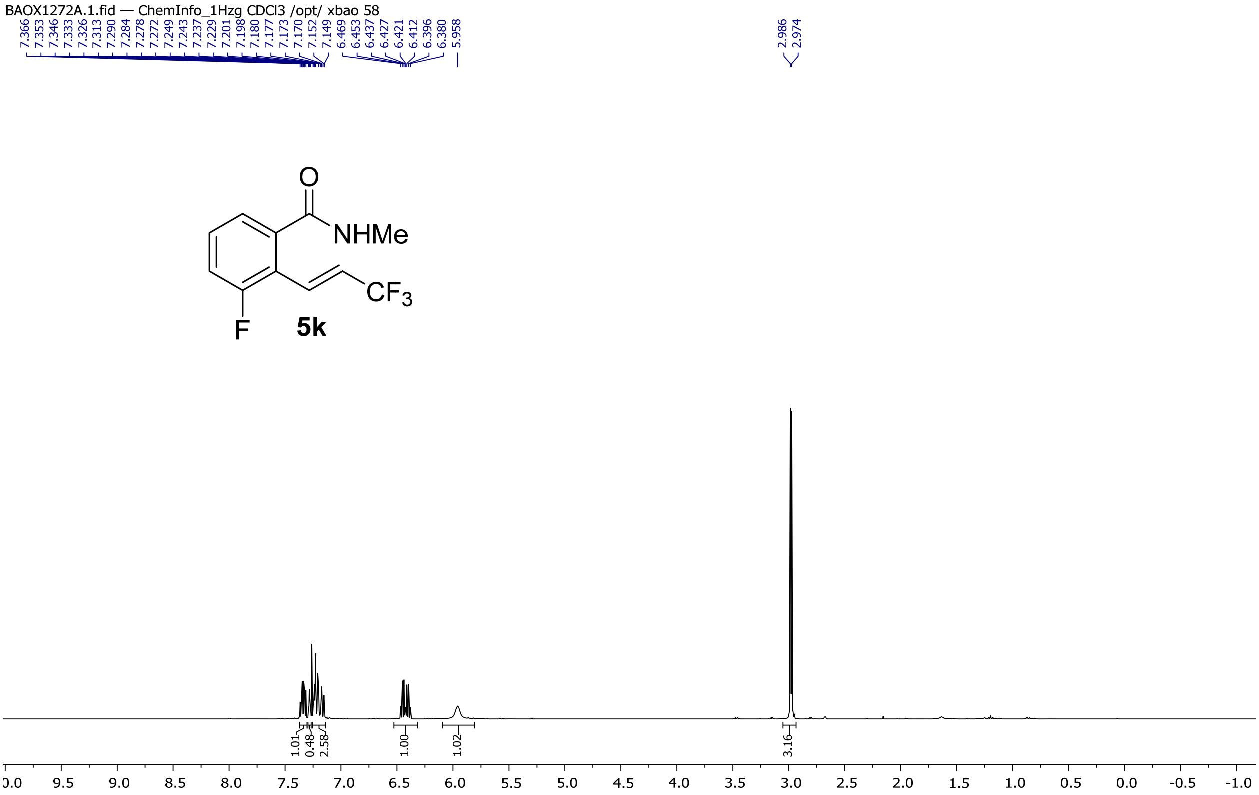

Supplementary Figure 75. ^{1}H spectra of 5k

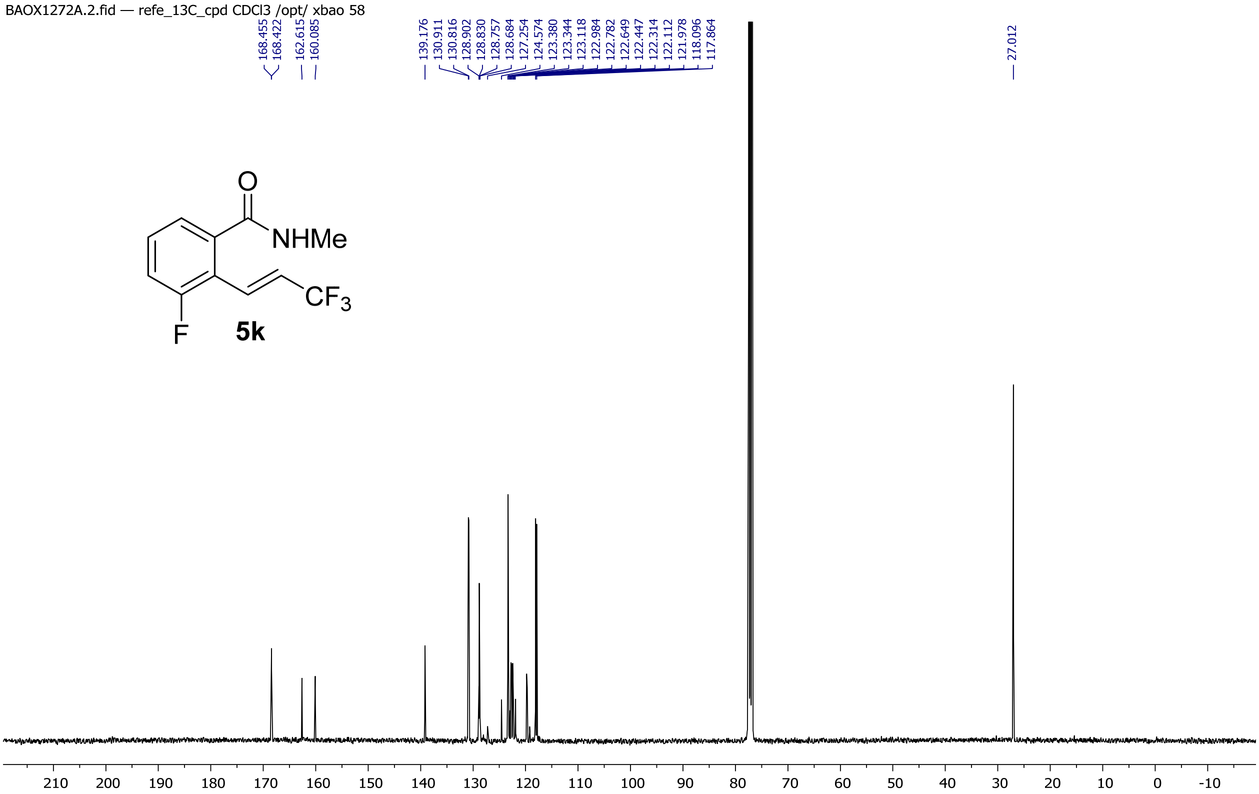

Supplementary Figure 76. ^{13}C NMR spectra of 5k

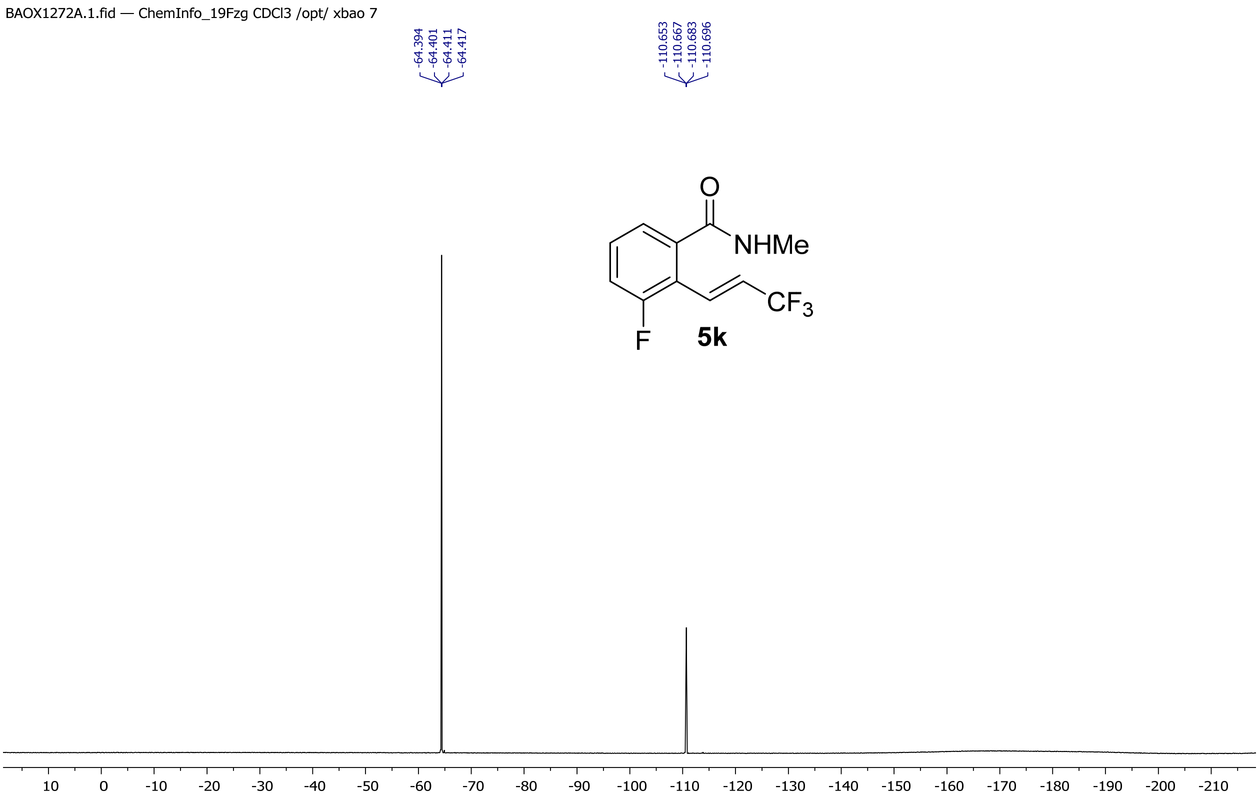

Supplementary Figure 77. ^{19}F spectra of 5k

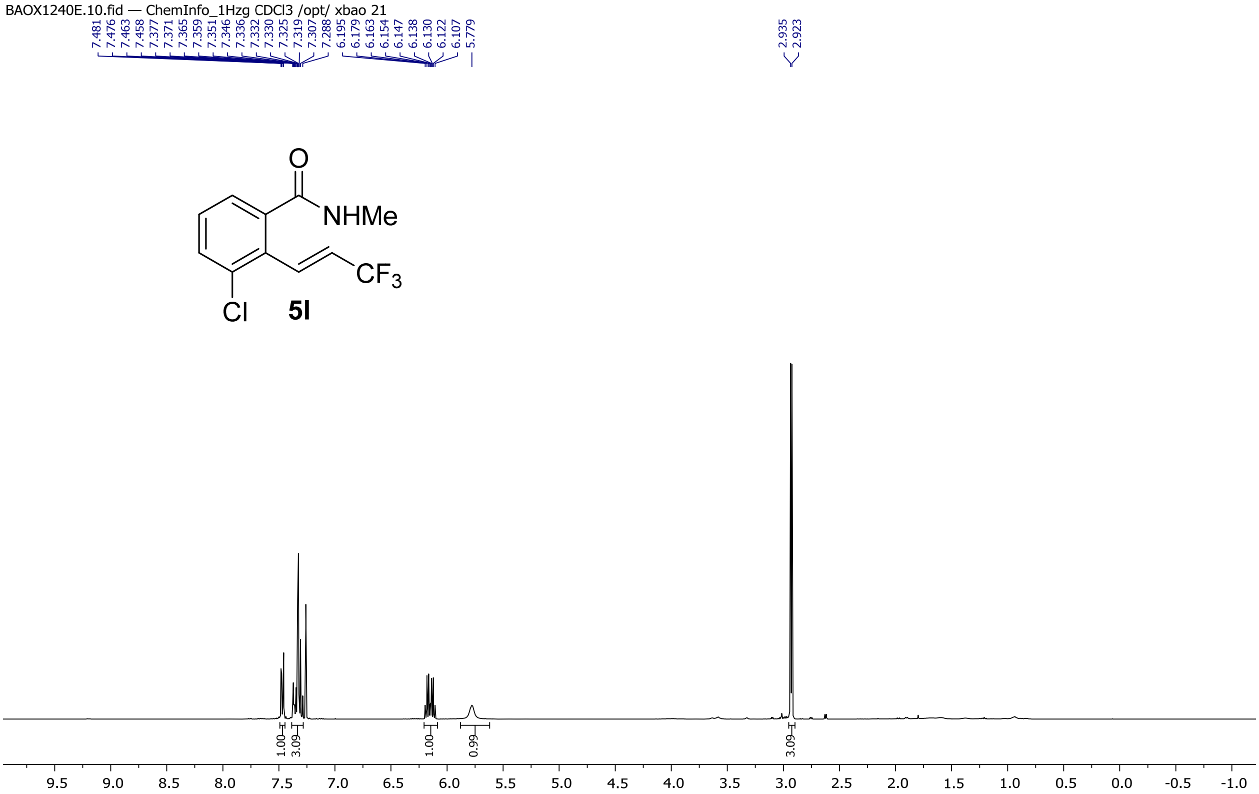

Supplementary Figure 78. ^{1}H spectra of 5l

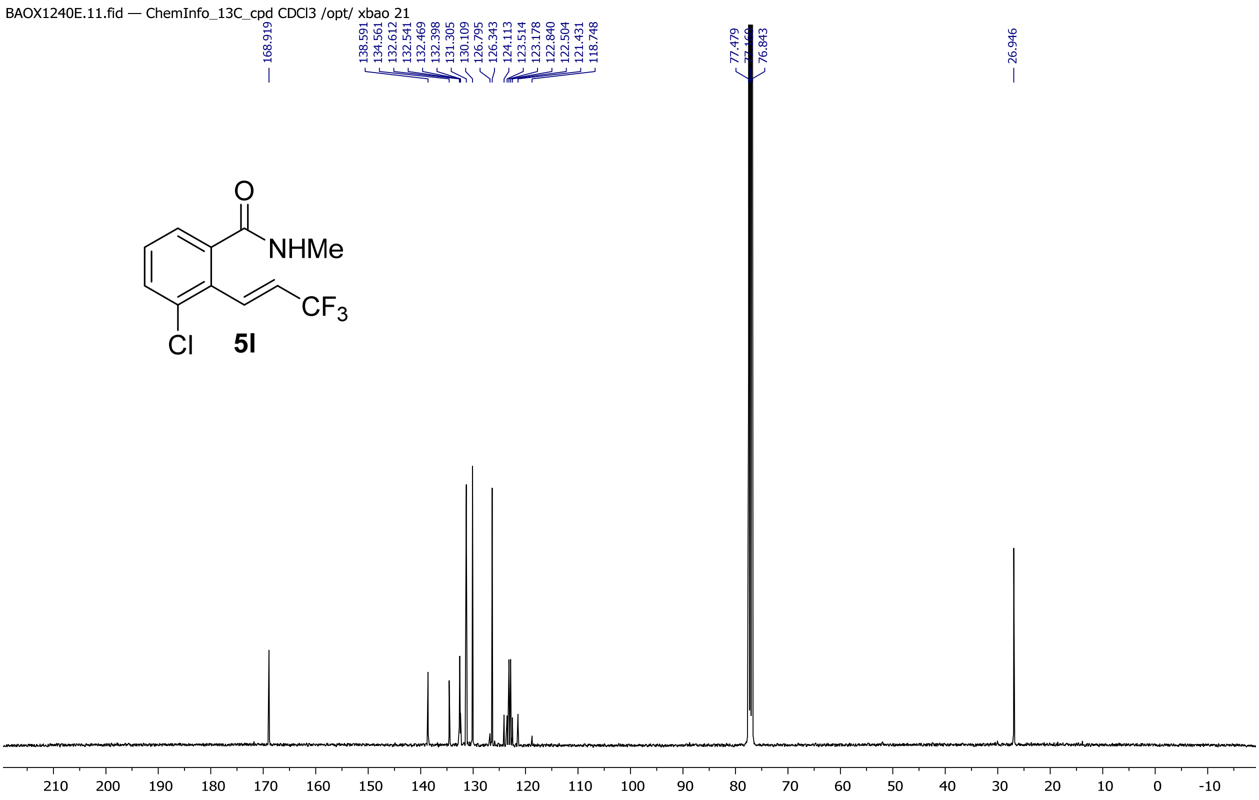

Supplementary Figure 79. ^{13}C NMR spectra of 5l

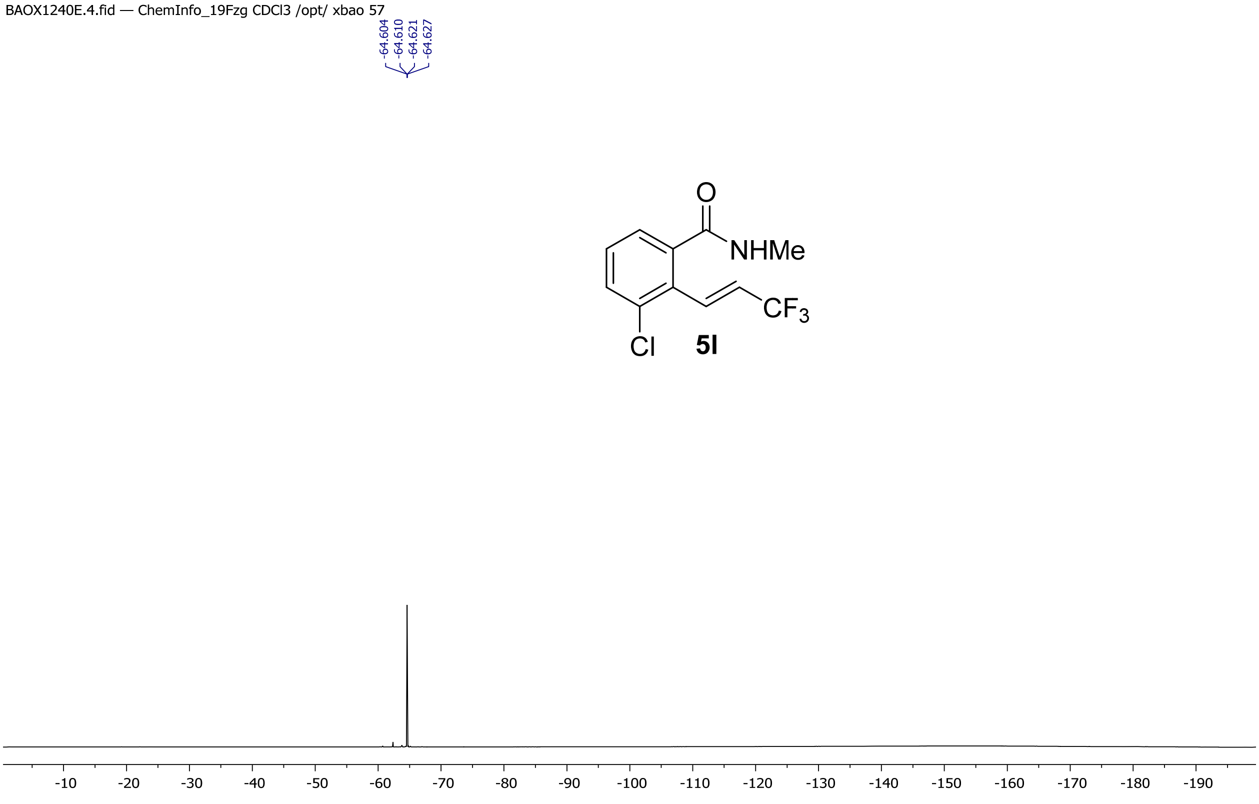

Supplementary Figure 80. ^{19}F spectra of 5l

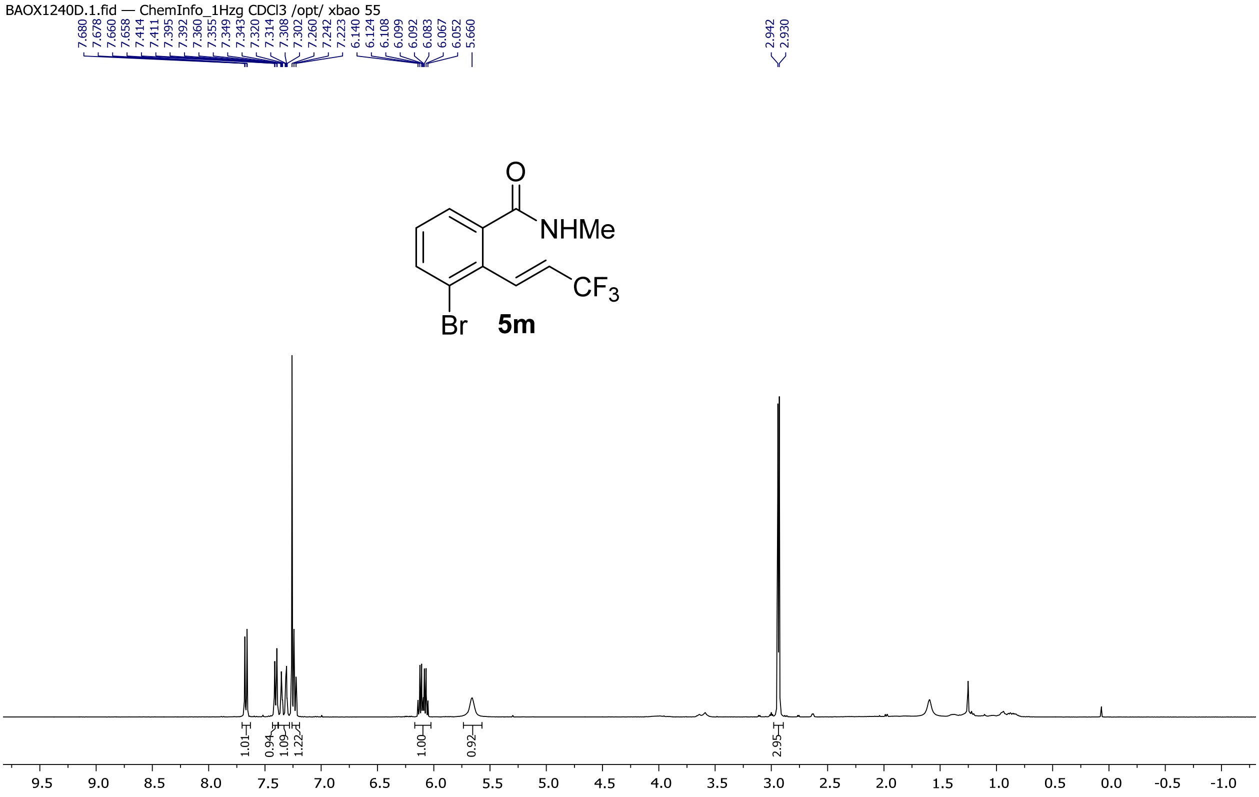

Supplementary Figure 81. **^{1}H spectra of 5m**

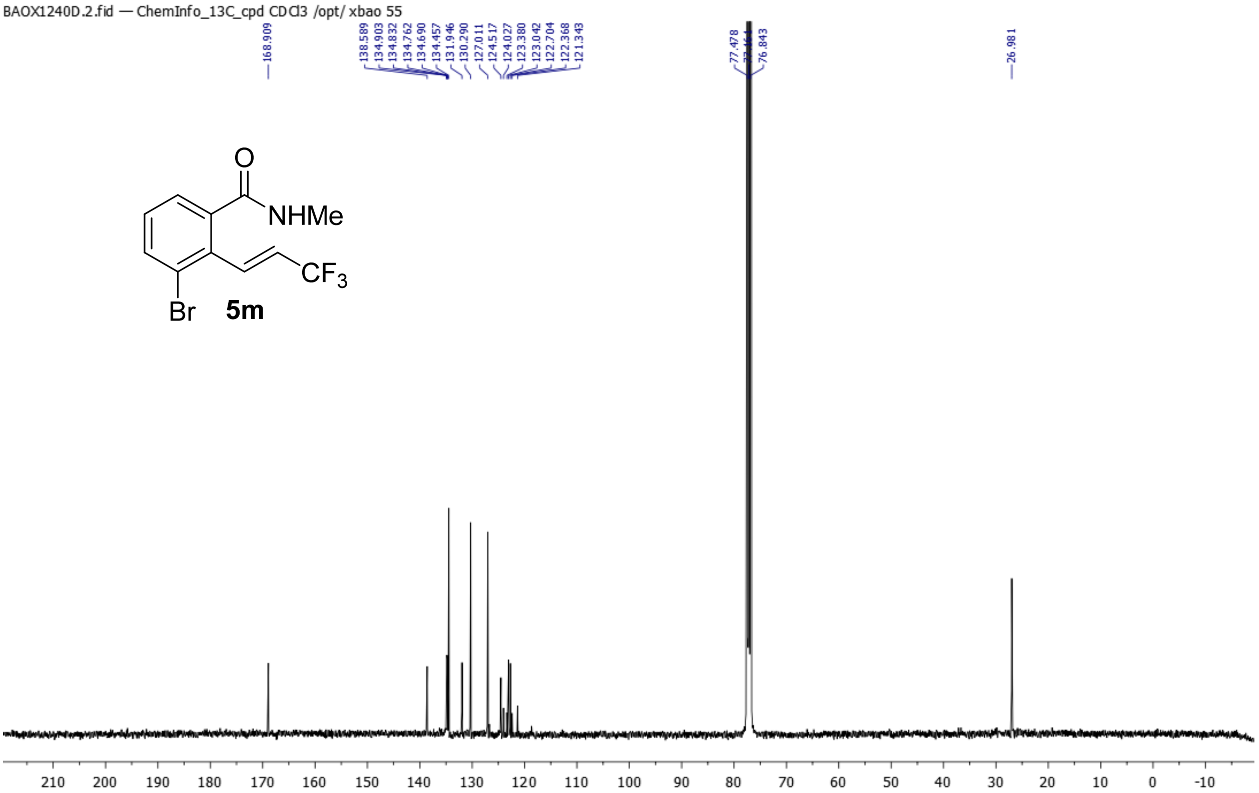

Supplementary Figure 82. **^{13}C NMR spectra of 5m**

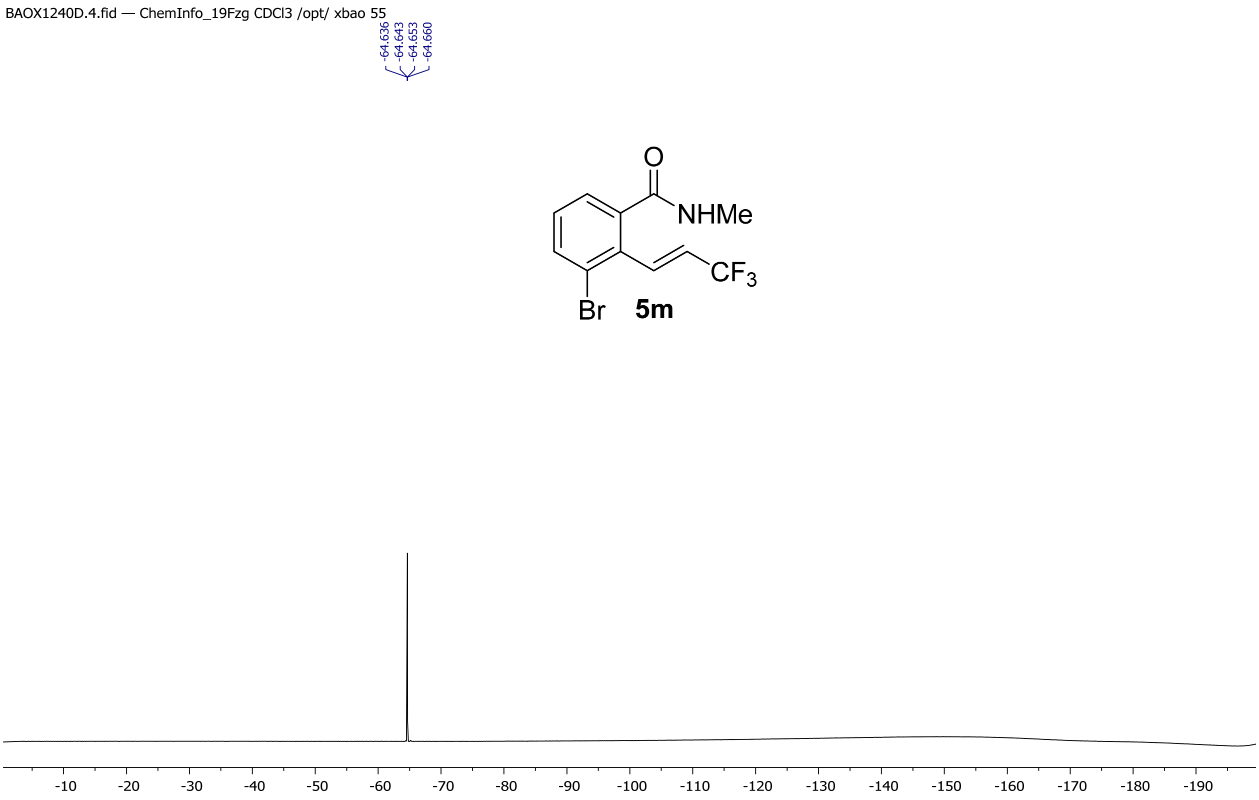

Supplementary Figure 83. ^{19}F spectra of 5m

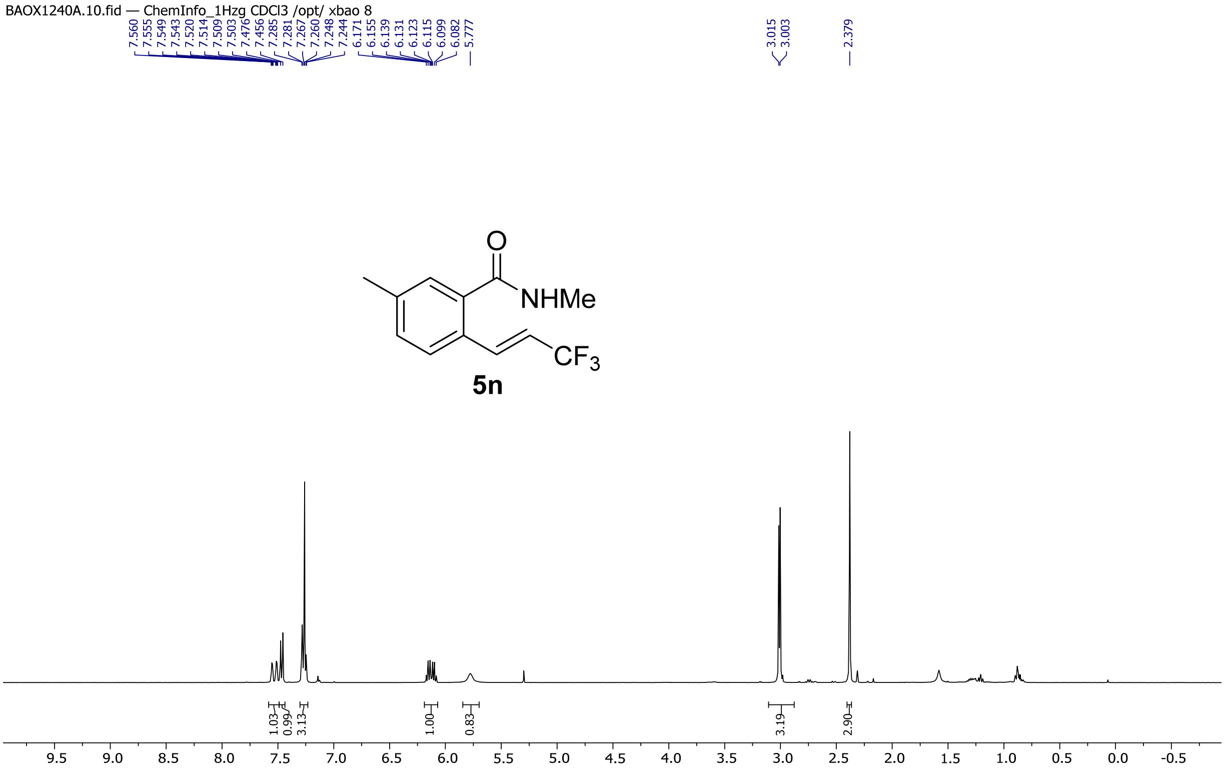

Supplementary Figure 84. ^{1}H spectra of 5n

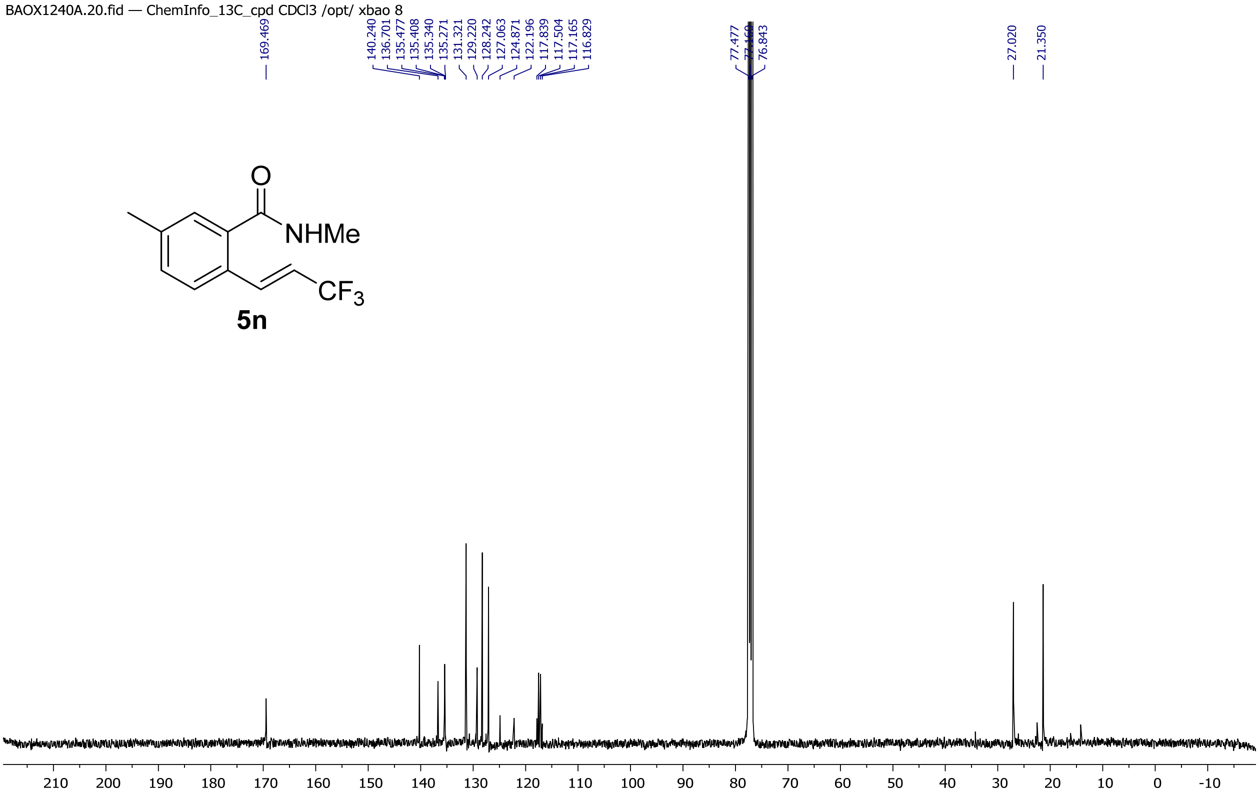

Supplementary Figure 85. ^{13}C NMR spectra of 5n

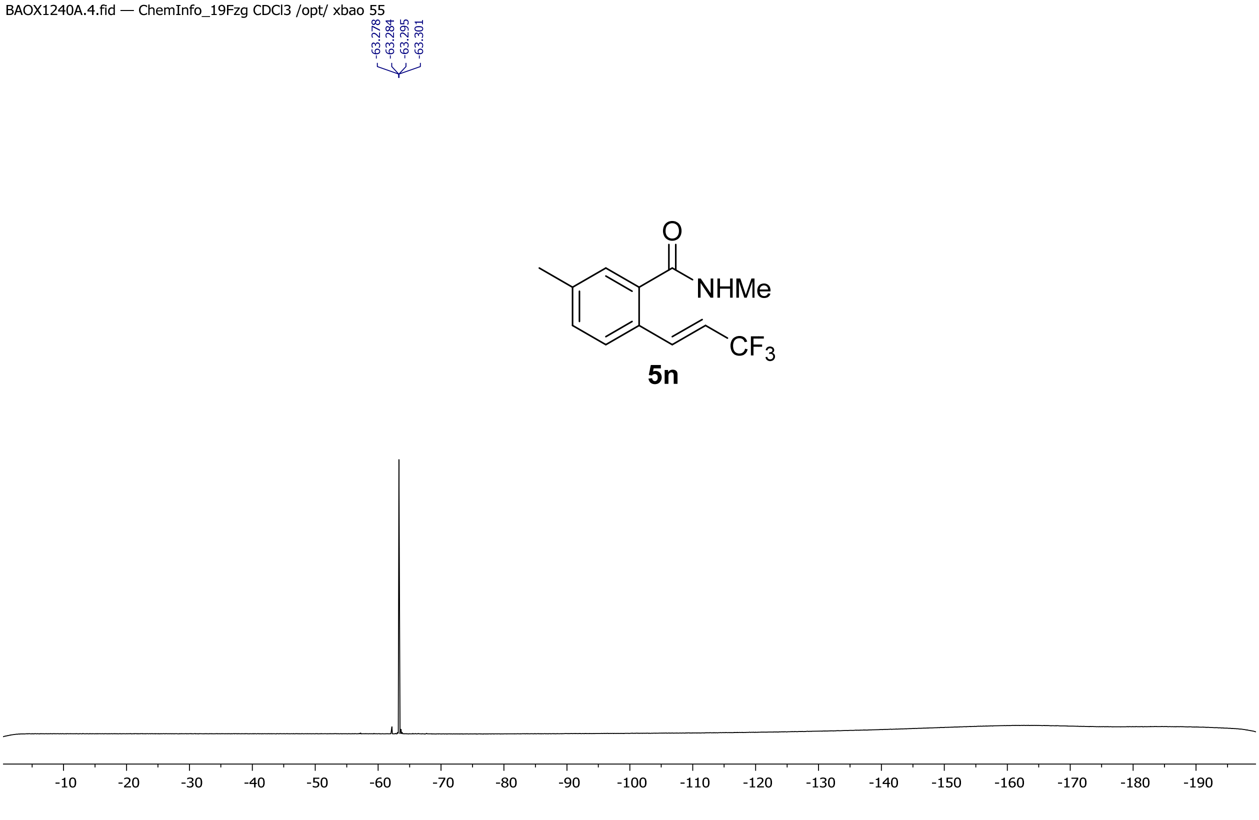

Supplementary Figure 86. ^{19}F spectra of 5n

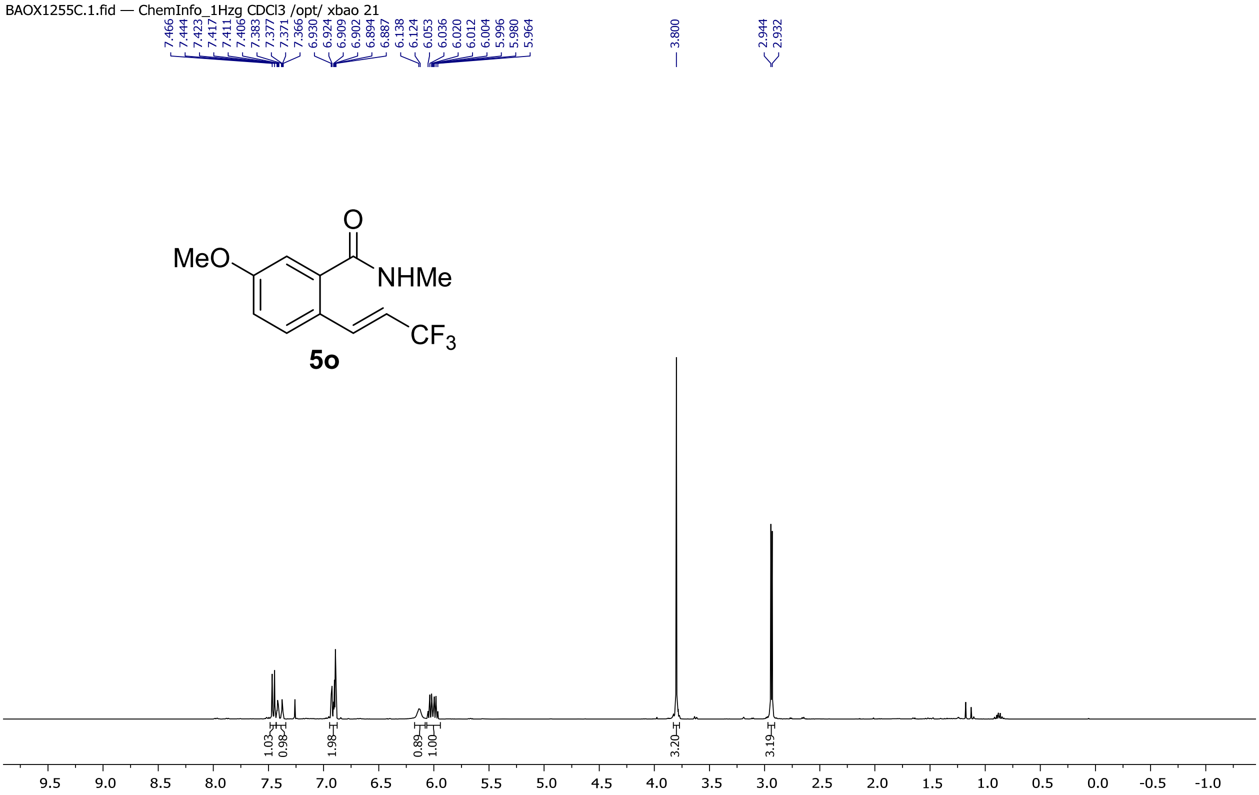

Supplementary Figure 87. ^{1}H spectra of 5o

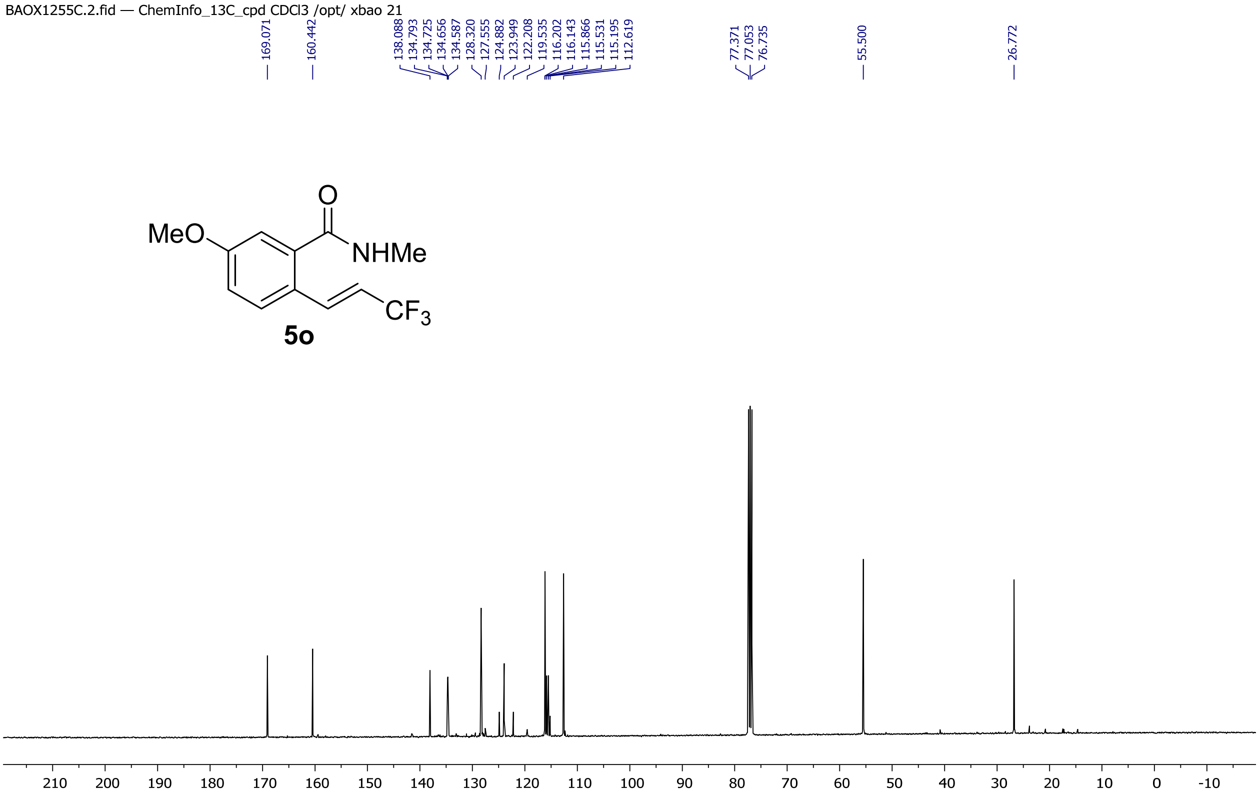

Supplementary Figure 88. ^{13}C NMR spectra of 5o

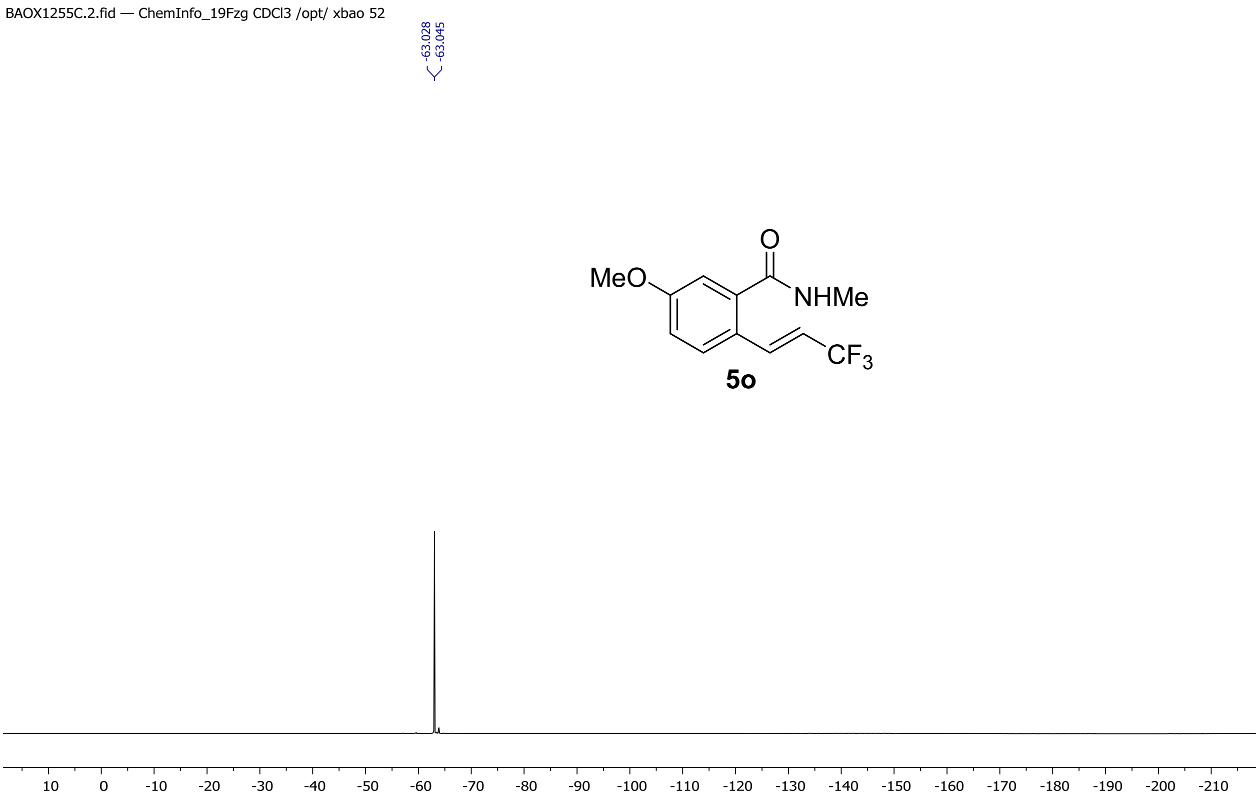

Supplementary Figure 89. ^{19}**F spectra of 5o**

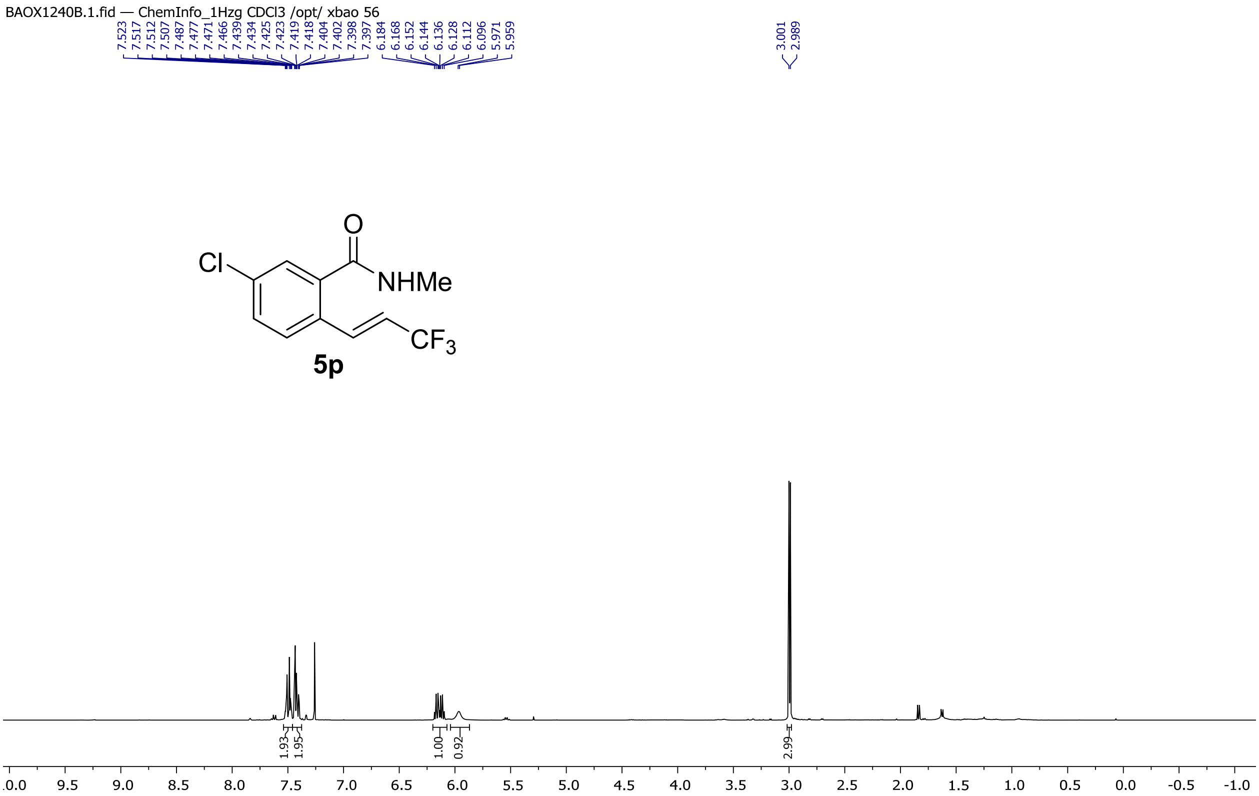

Supplementary Figure 90. ^{1}**H spectra of 5p**

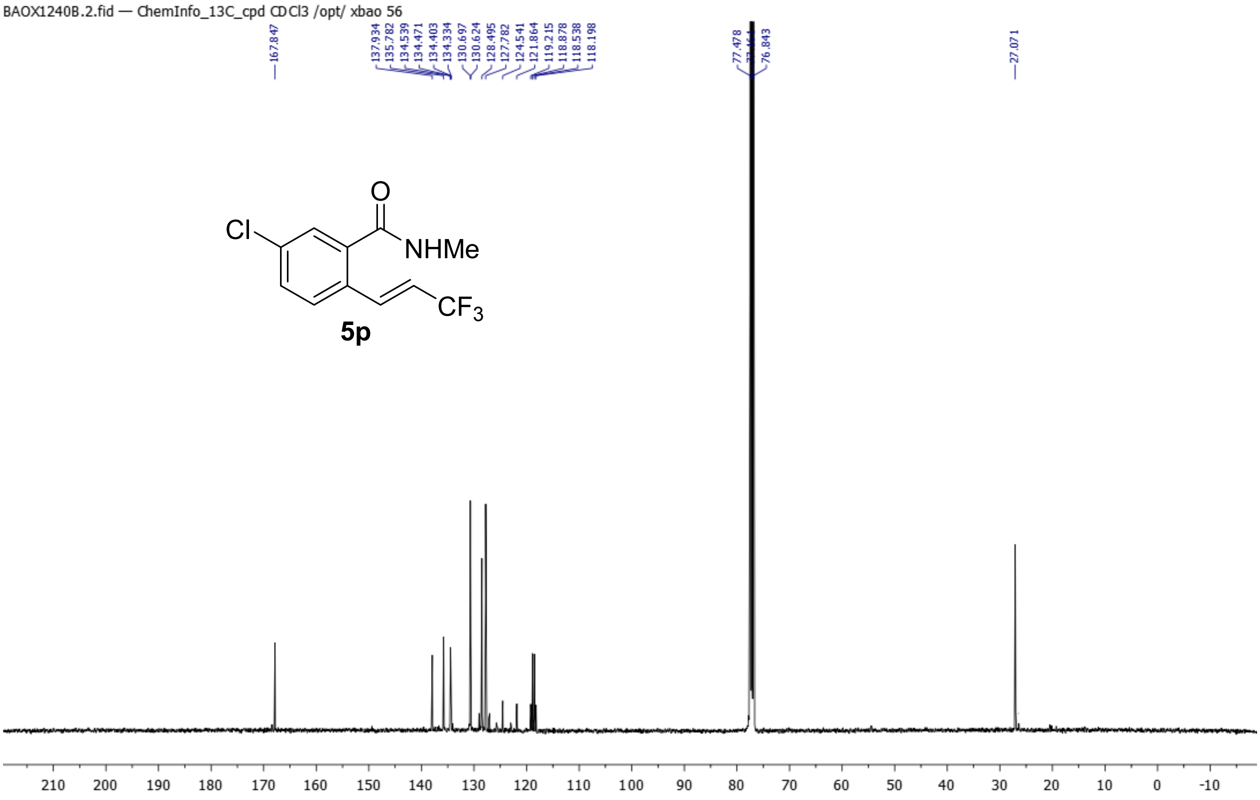

Supplementary Figure 91. ^{13}C NMR spectra of 5p

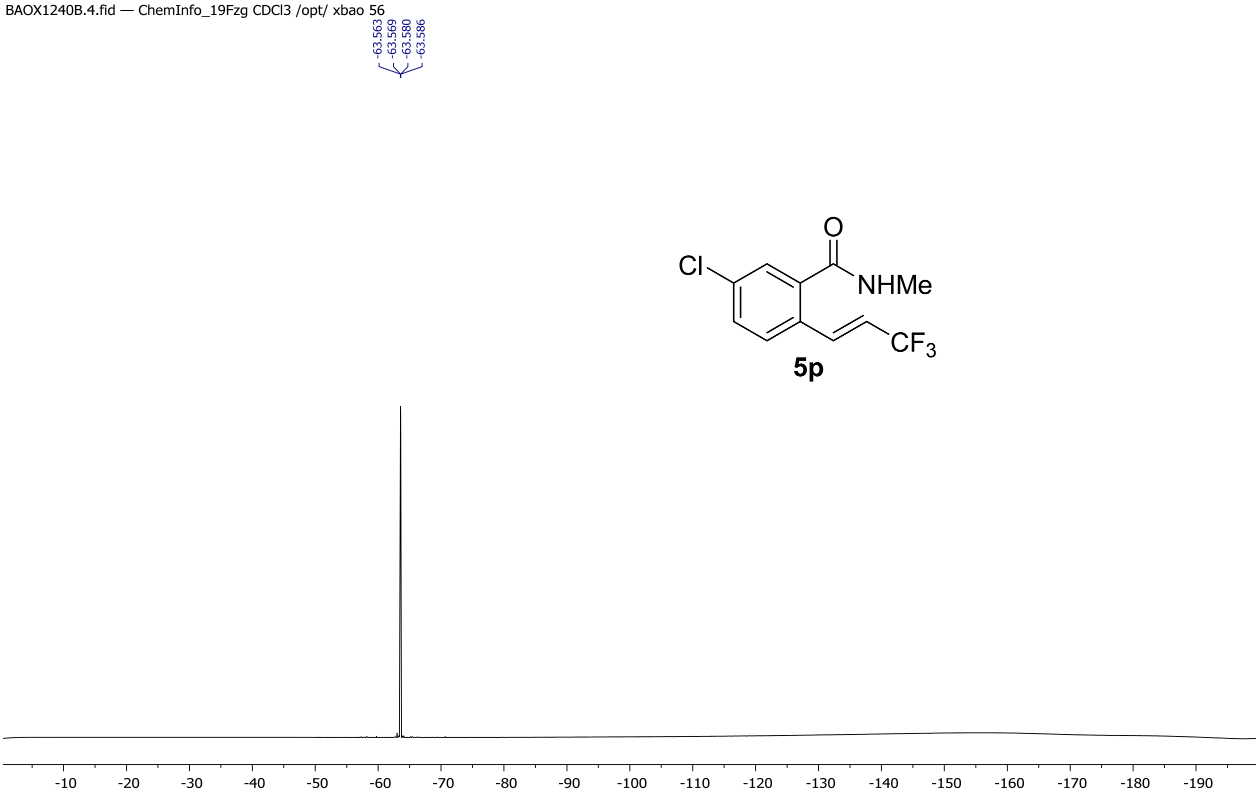

Supplementary Figure 92. ^{19}F spectra of 5p

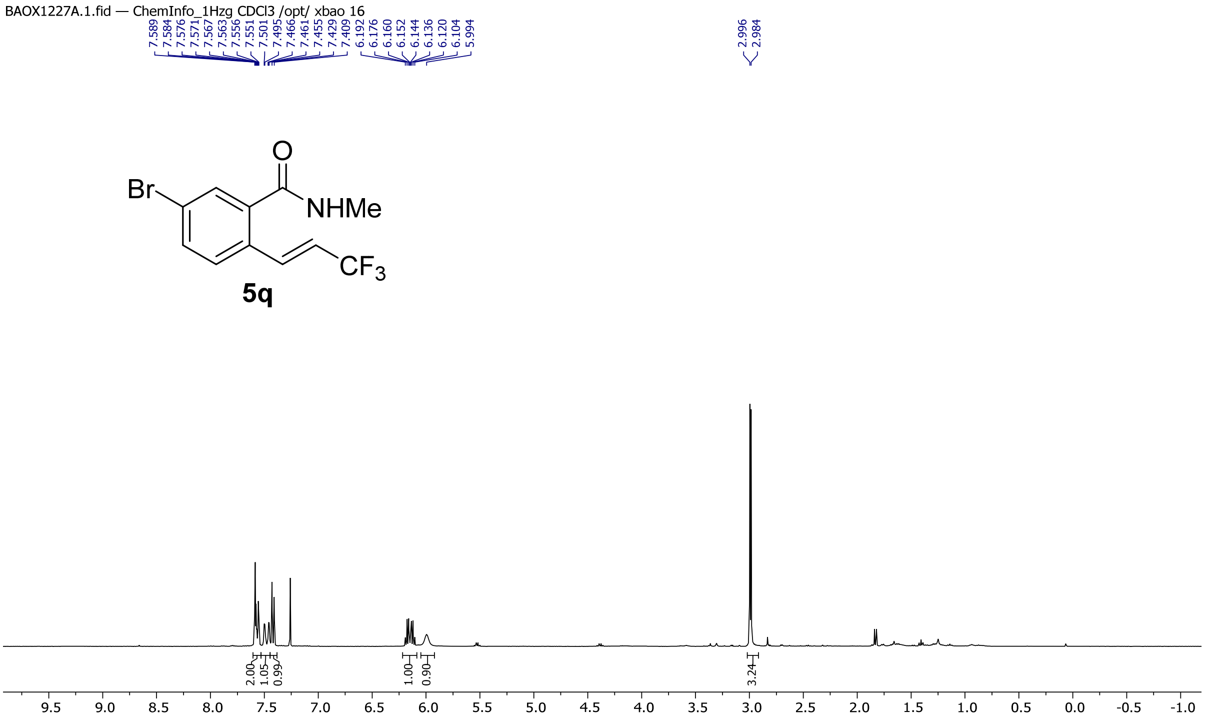

Supplementary Figure 93. ^{1}H spectra of 5q

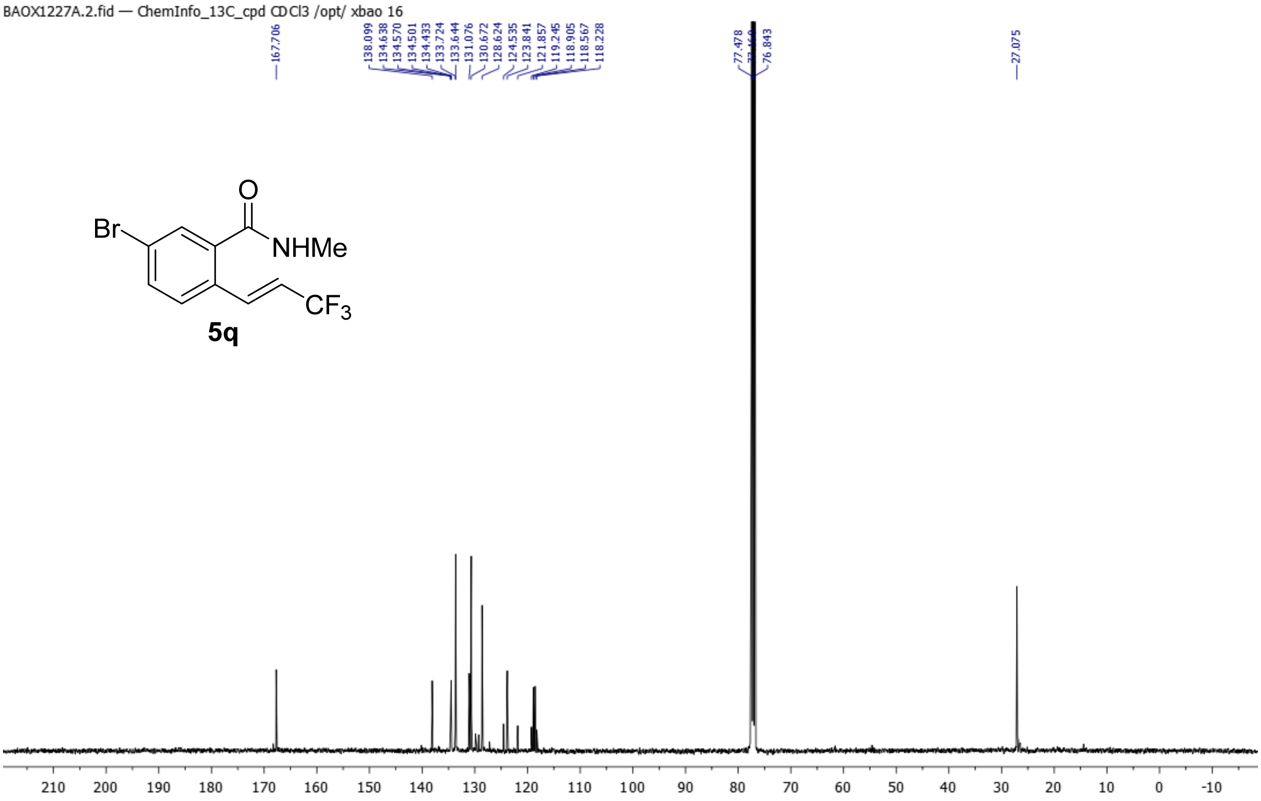

Supplementary Figure 94. ^{13}C NMR spectra of 5q

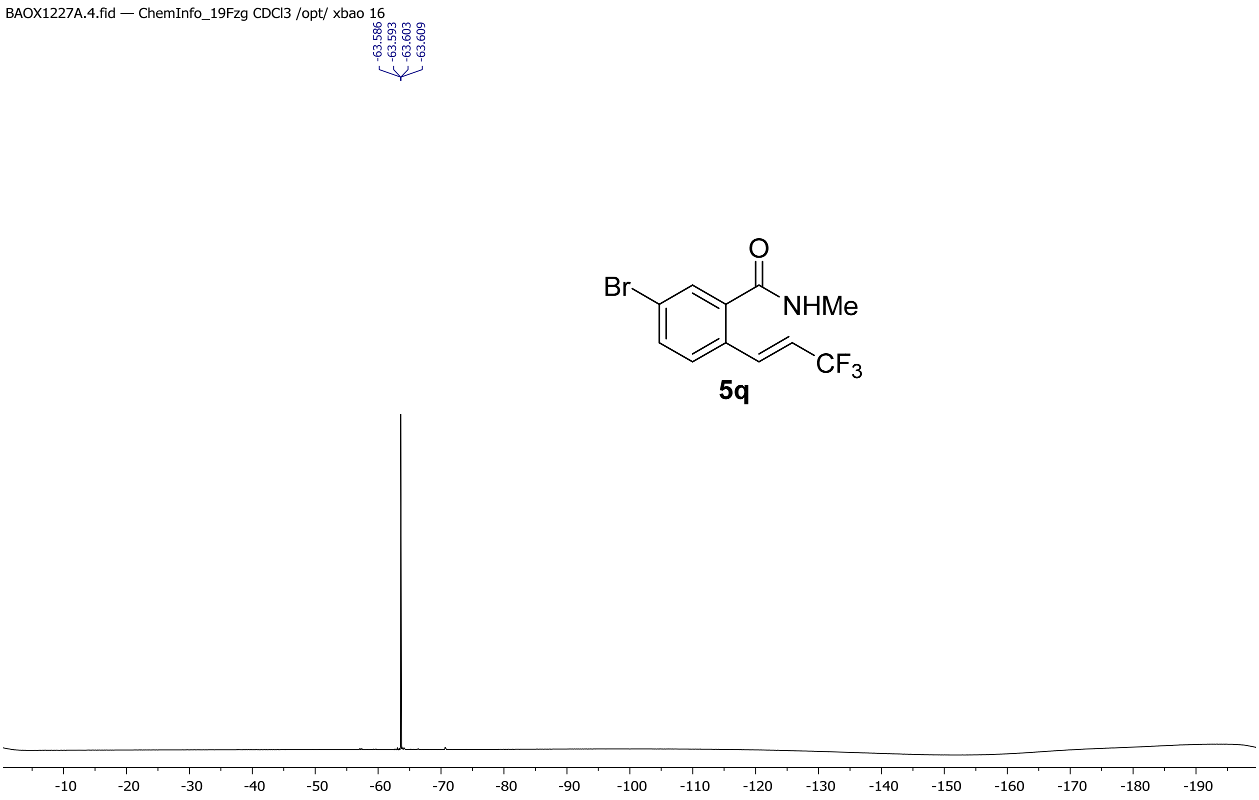

Supplementary Figure 95. ^{19}F spectra of 5q

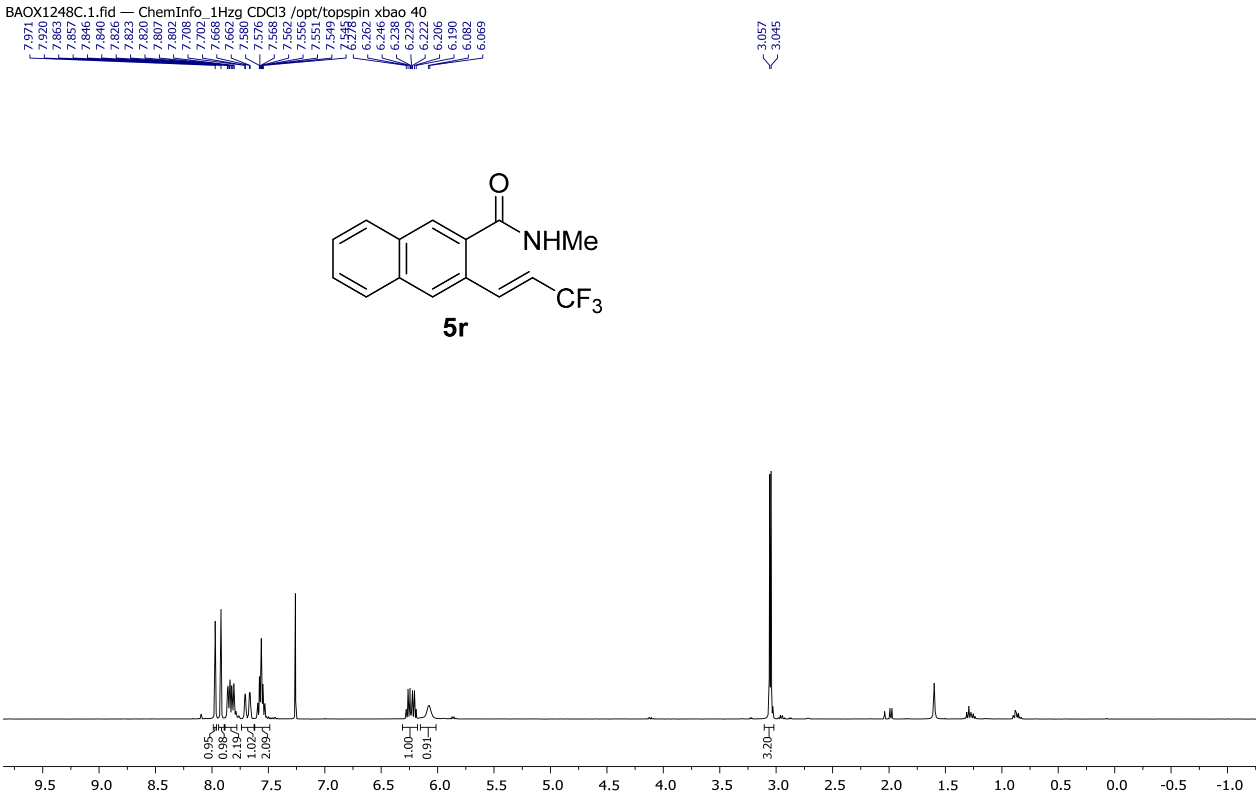

Supplementary Figure 96. ^{1}H spectra of 5r

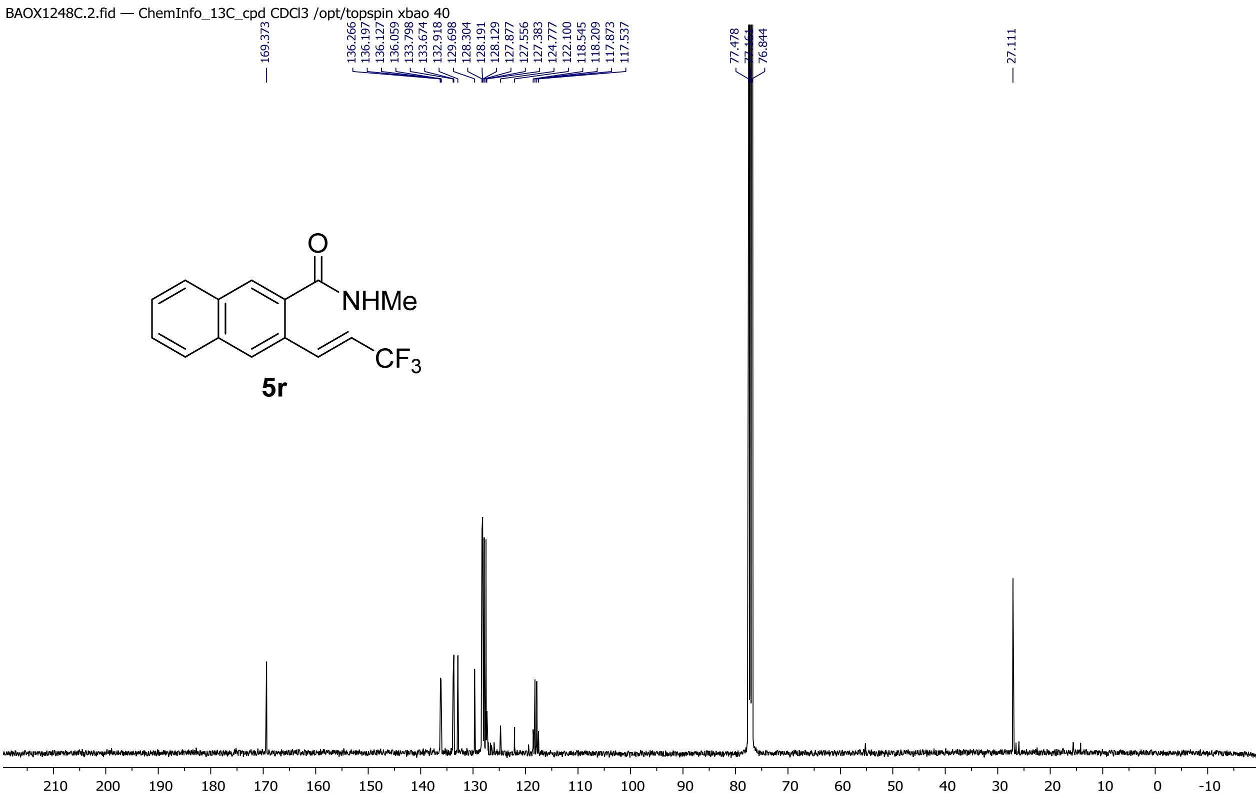

Supplementary Figure 97. ^{13}C NMR spectra of 5r

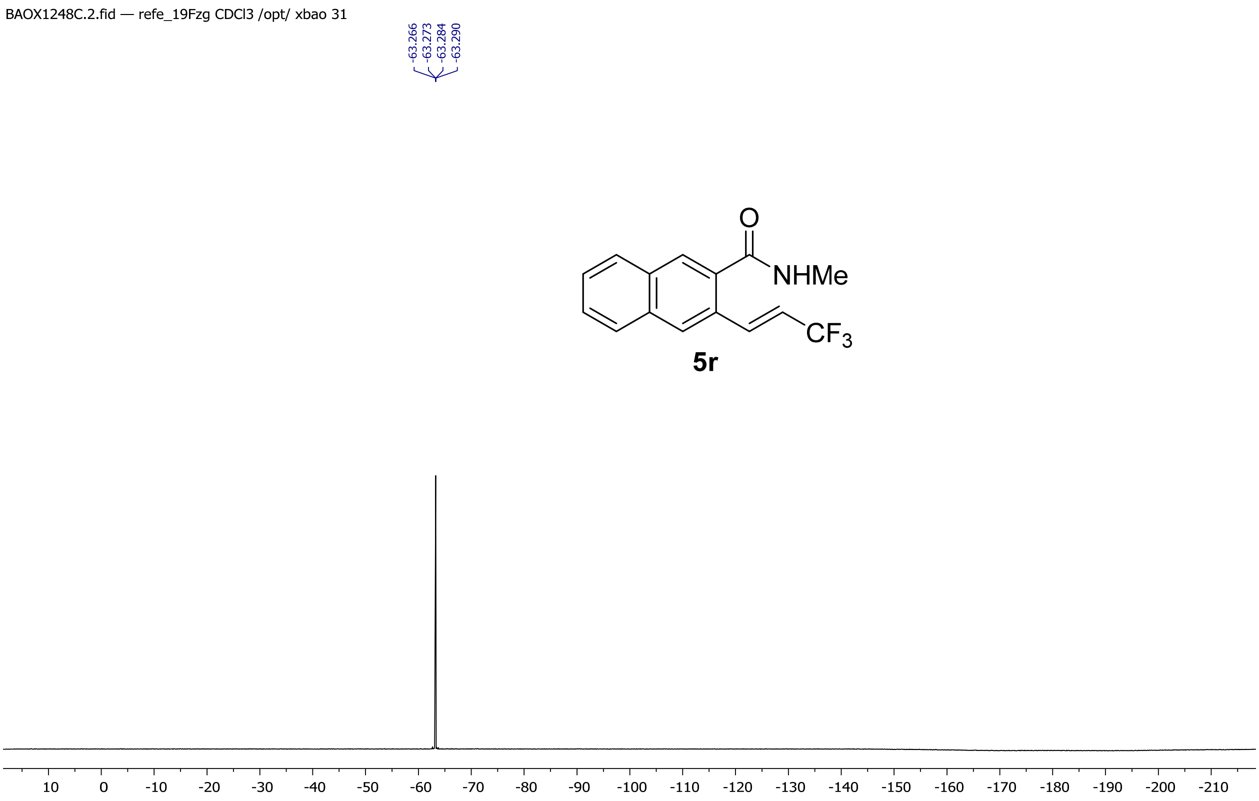

Supplementary Figure 98. ^{19}F spectra of 5r

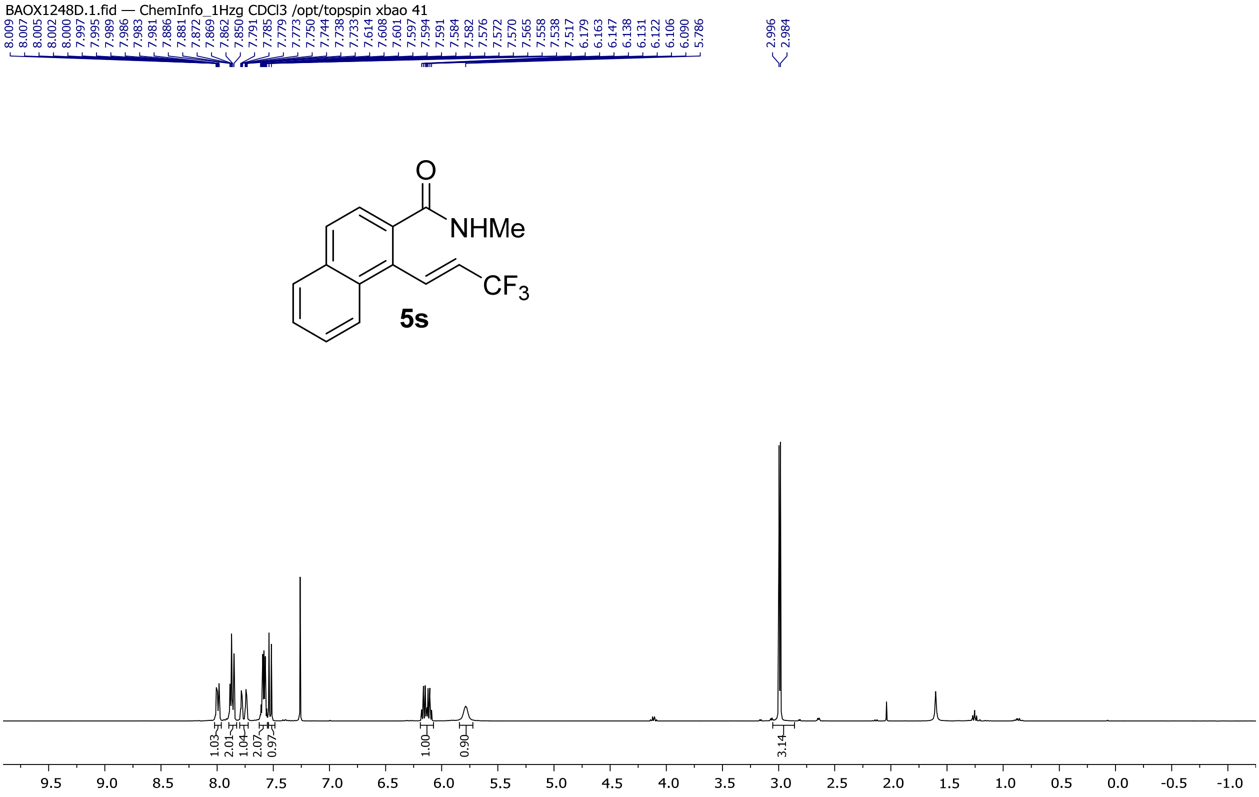

Supplementary Figure 99. ^{1}H spectra of 5s

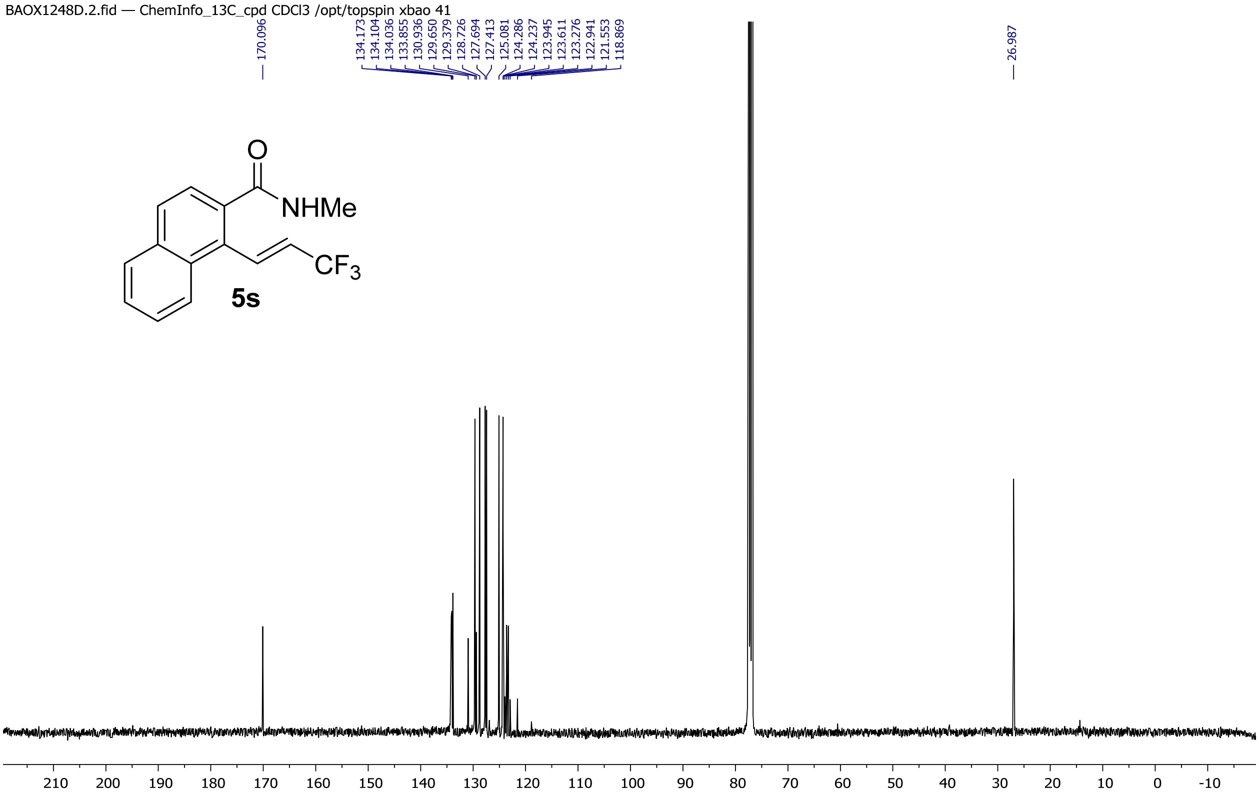

Supplementary Figure 100. ^{13}C NMR spectra of 5s

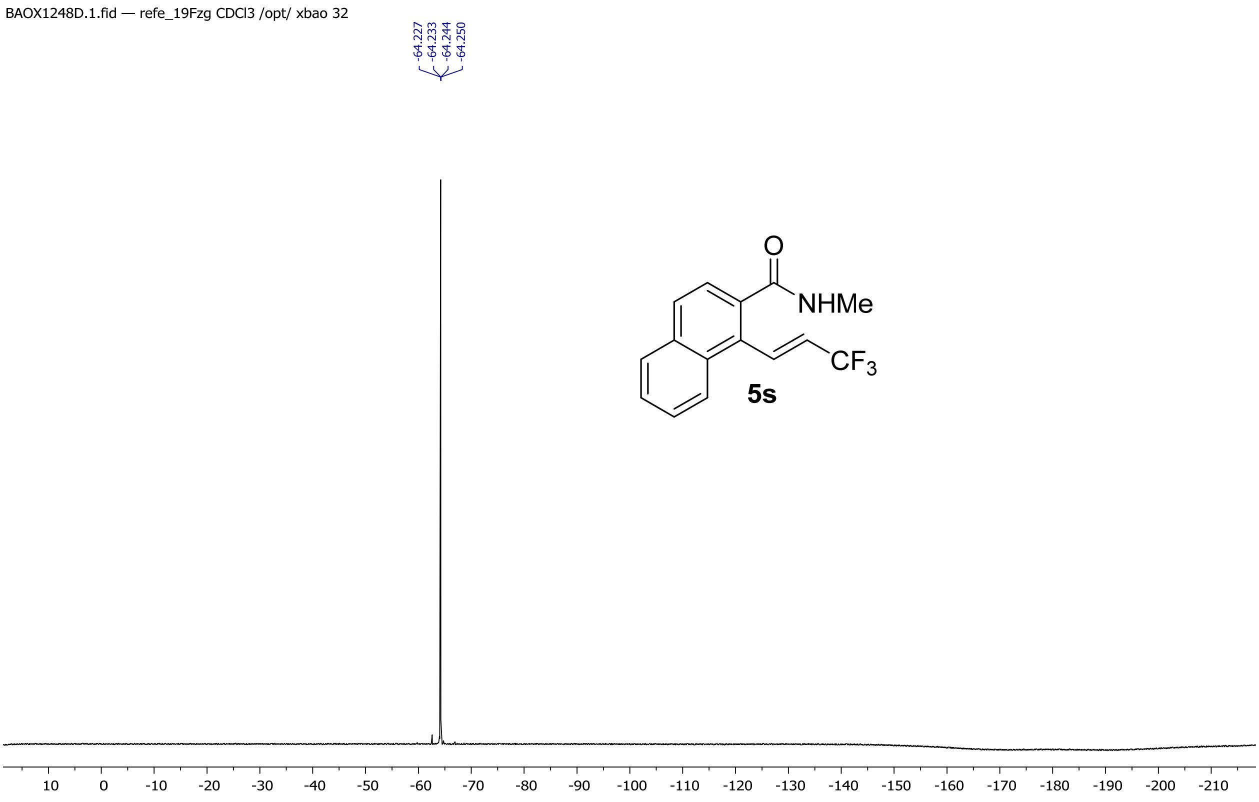

Supplementary Figure 101. ^{19}F spectra of 5s

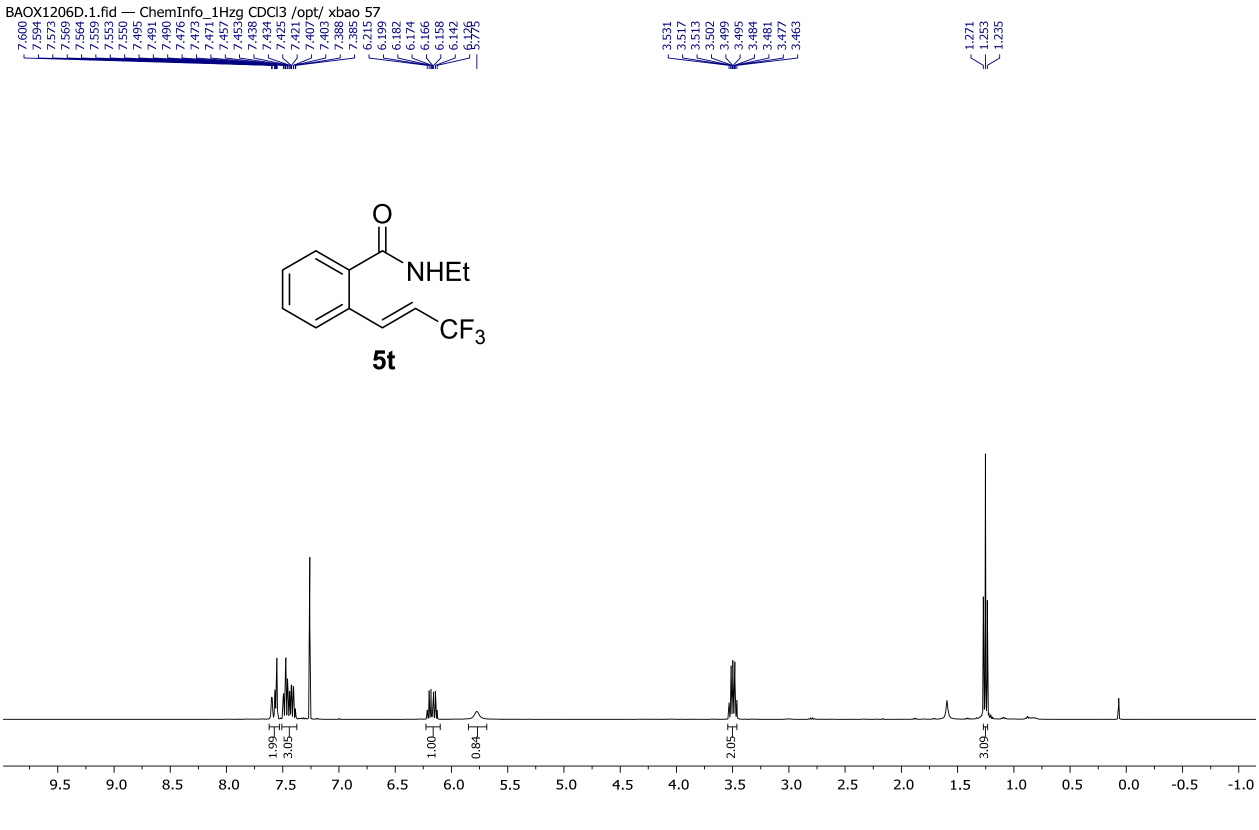

Supplementary Figure 102. ^{1}H spectra of 5t

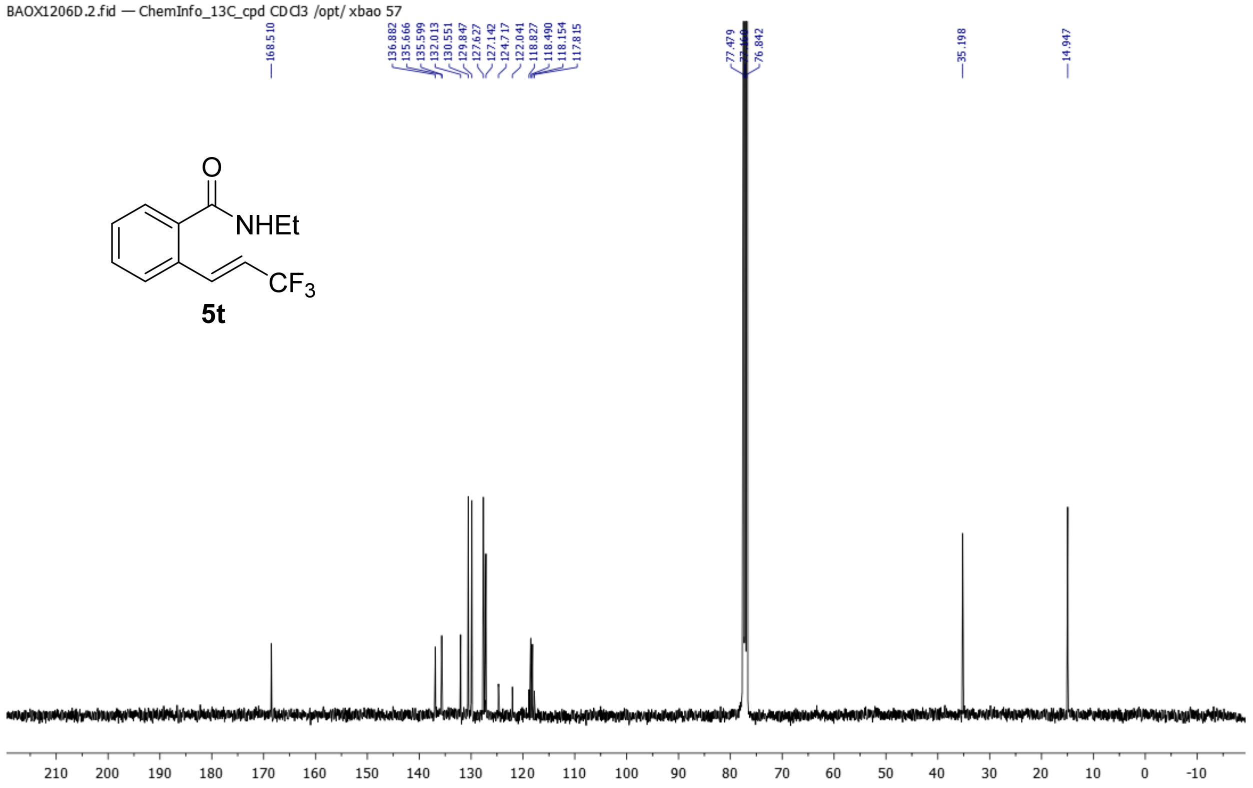

Supplementary Figure 103. ^{13}C NMR spectra of 5t

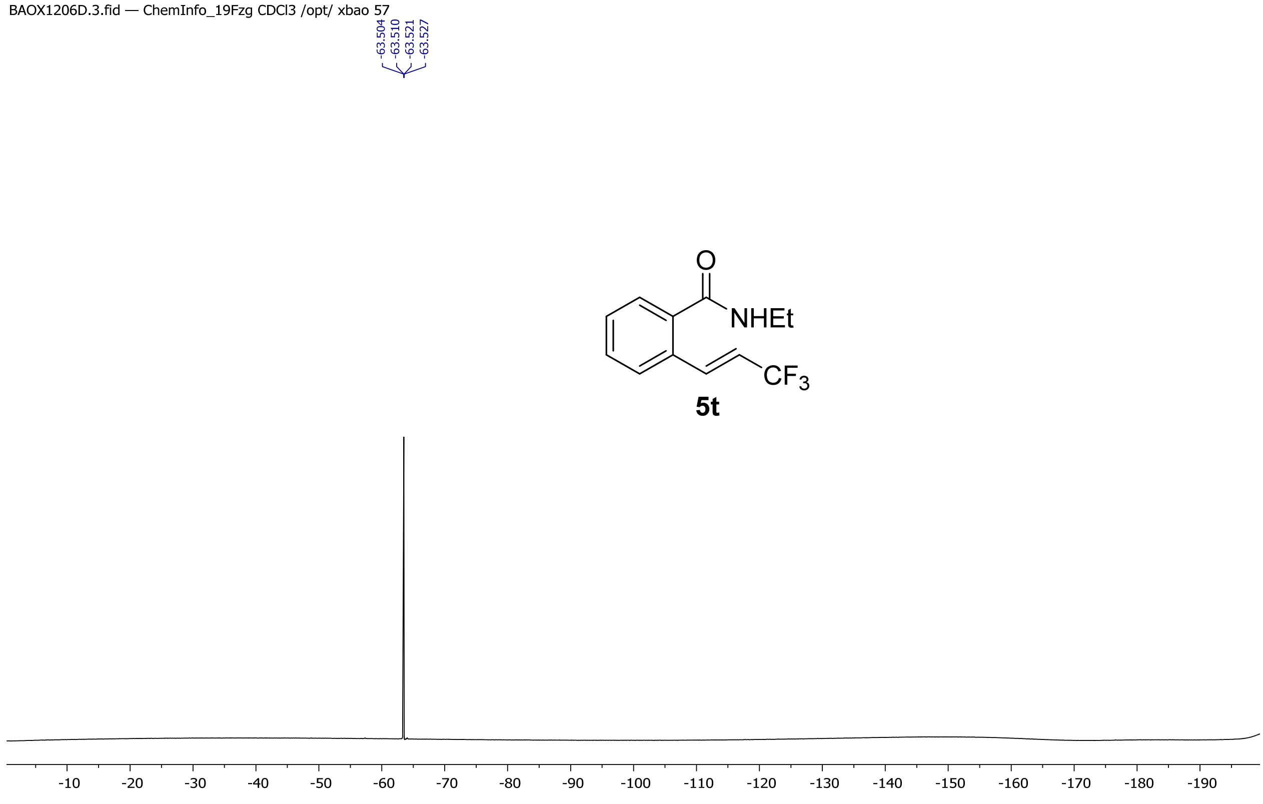

Supplementary Figure 104. ^{19}F spectra of 5t

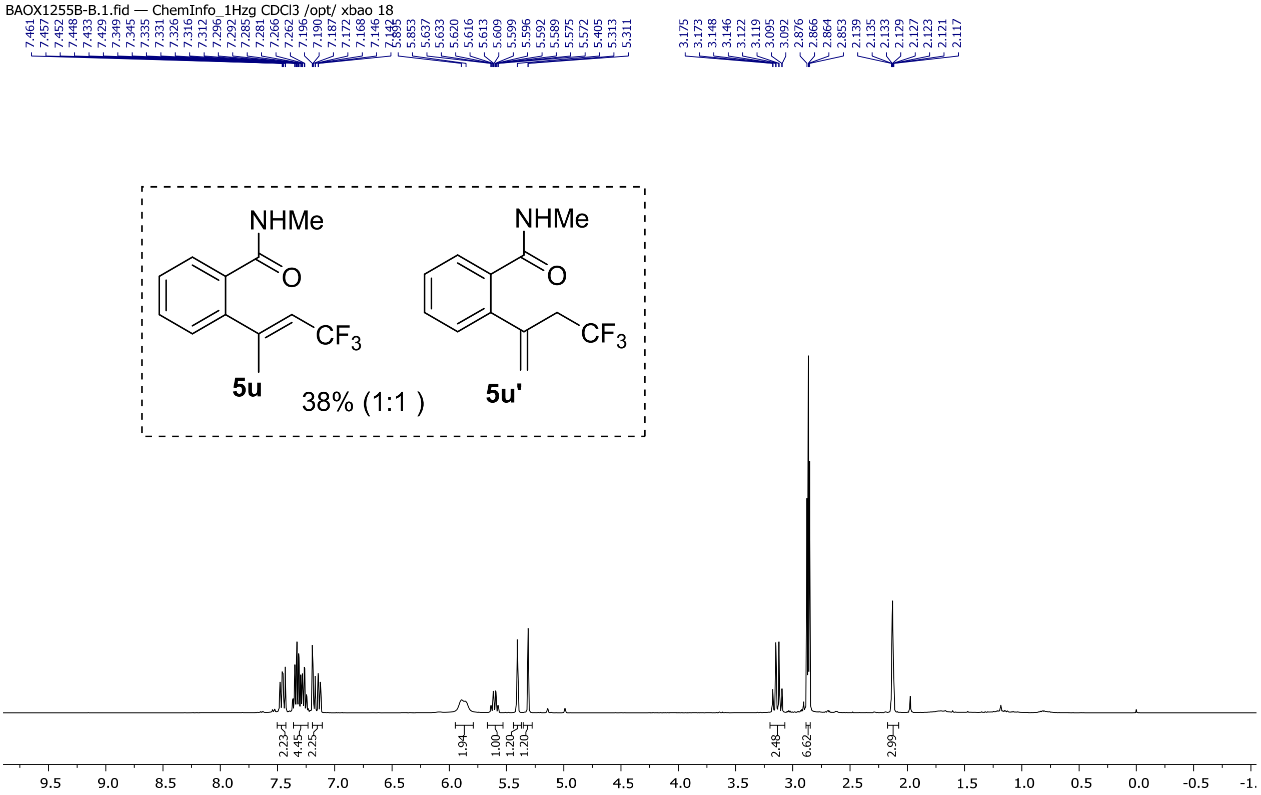

Supplementary Figure 105. **^{1}H spectra of 5u & 5u'**

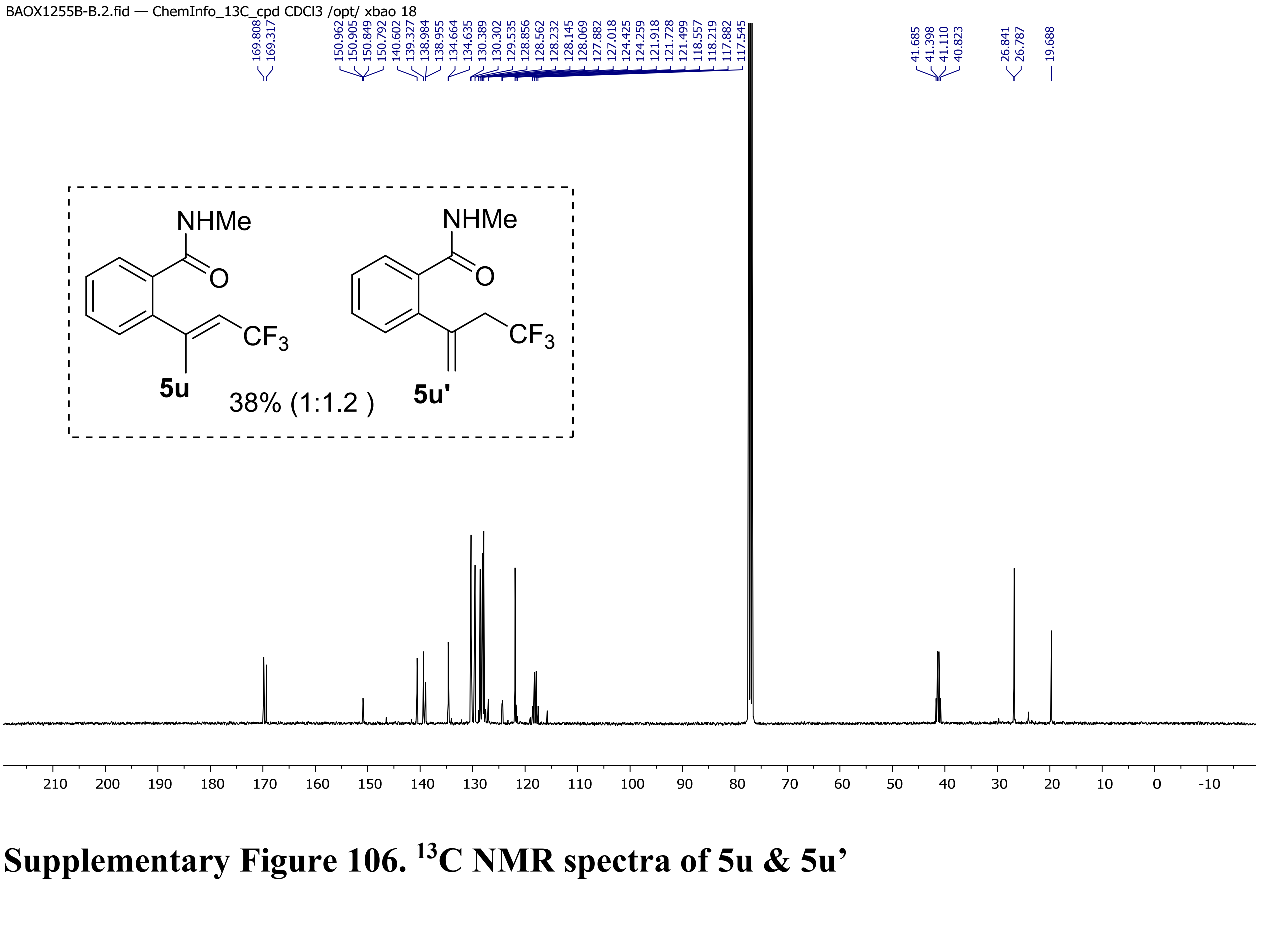

Supplementary Figure 106. **^{13}C NMR spectra of 5u & 5u'**

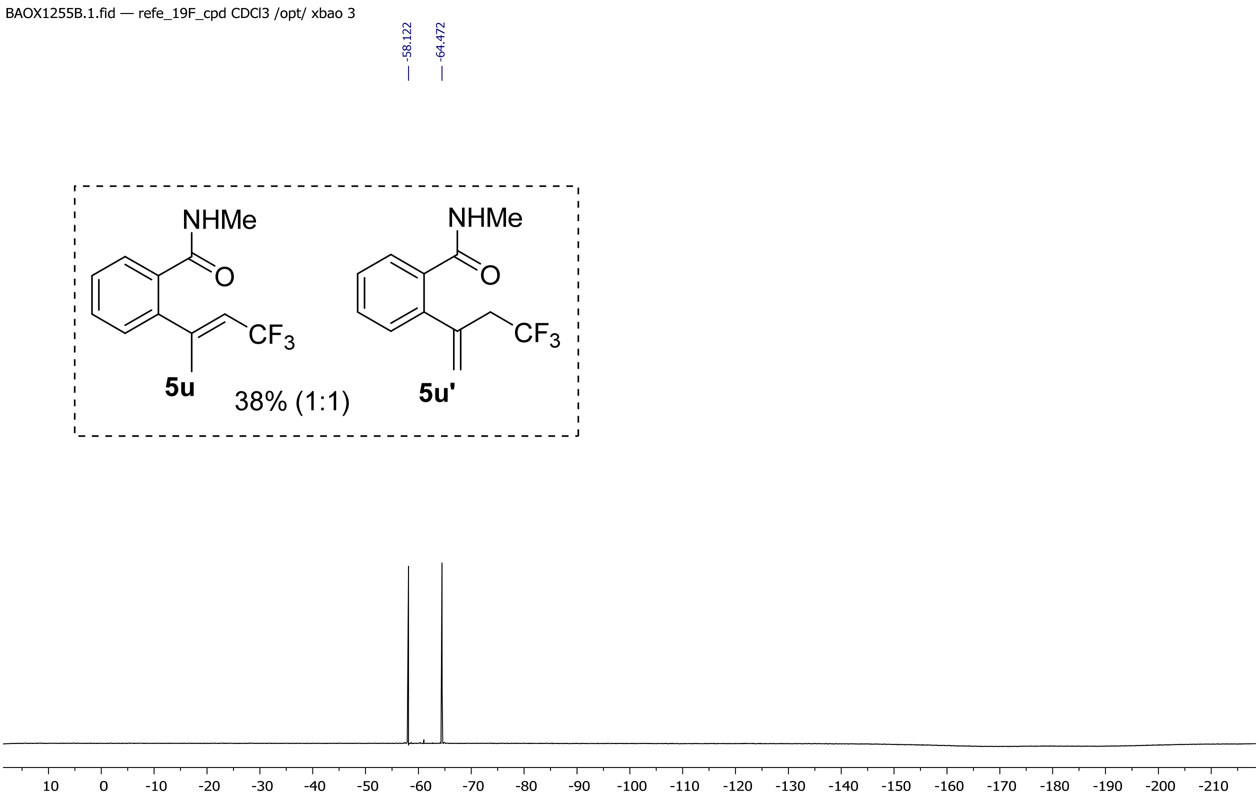

Supplementary Figure 107. ^{19}F spectra of 5u & 5u'

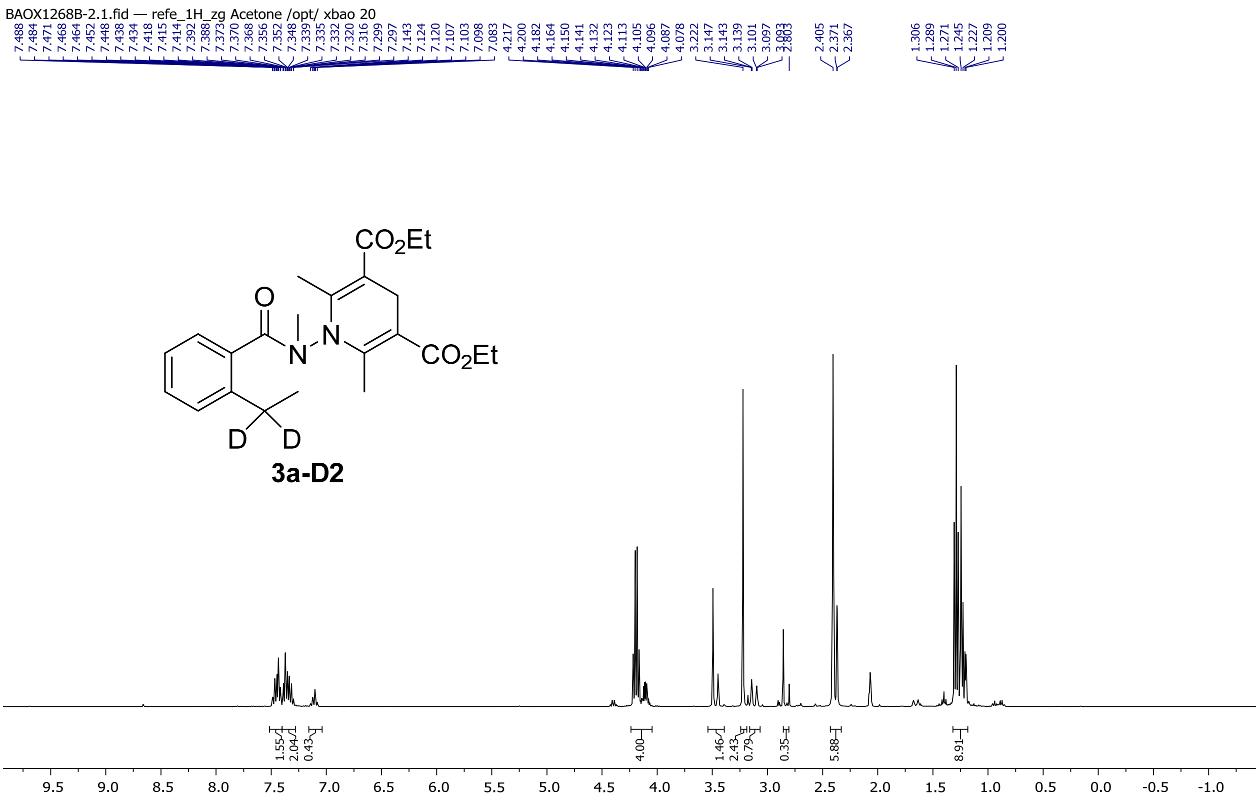

Supplementary Figure 108. ^{1}H spectra of 3a-D2

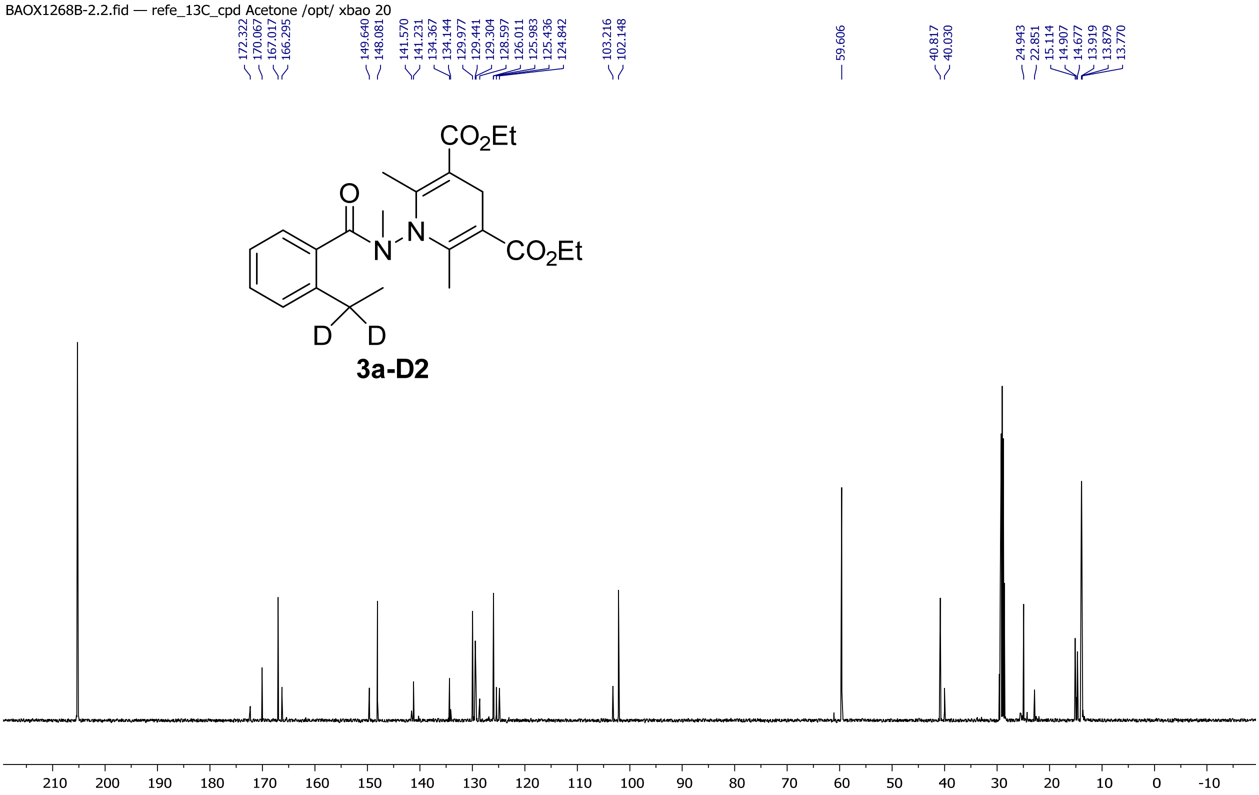

Supplementary Figure 109. ^{13}C NMR spectra of 3a-D2

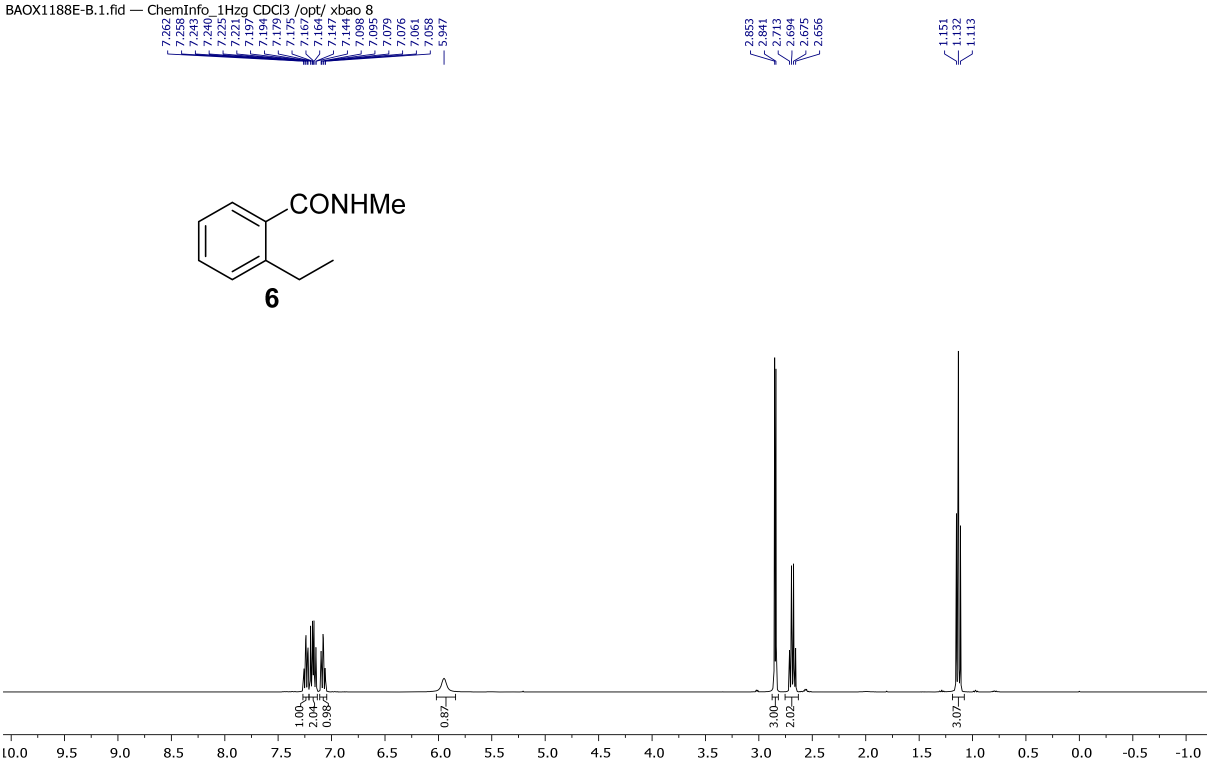

Supplementary Figure 110. ^{1}H NMR spectra of 6

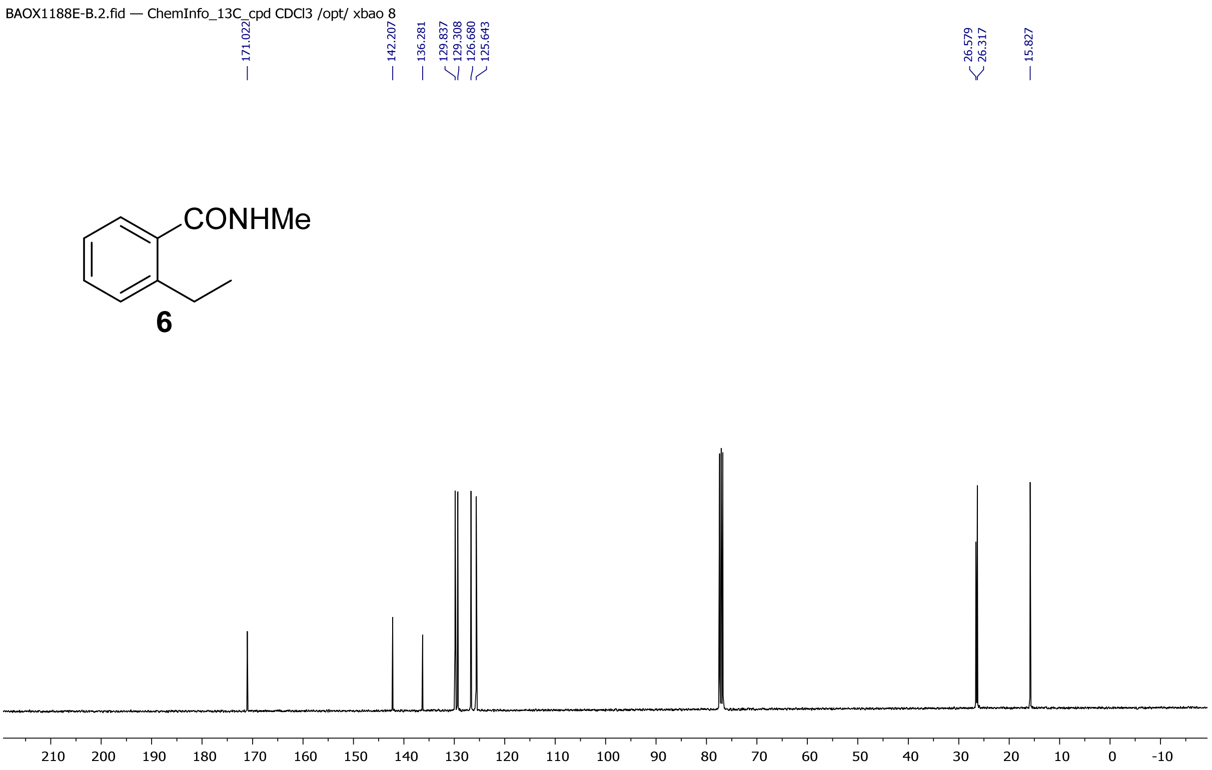

Supplementary Figure 111. ^{13}C NMR spectra of 6

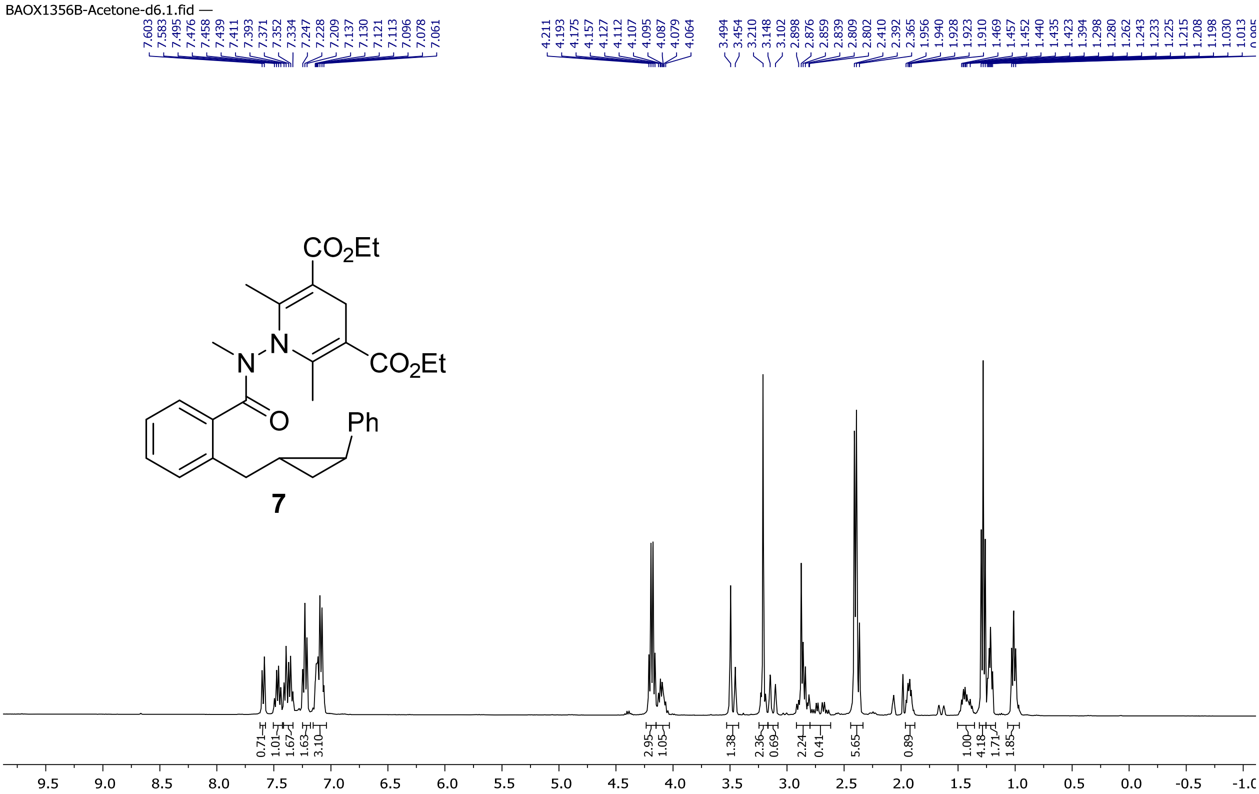

Supplementary Figure 112. ^{1}H spectra of 7

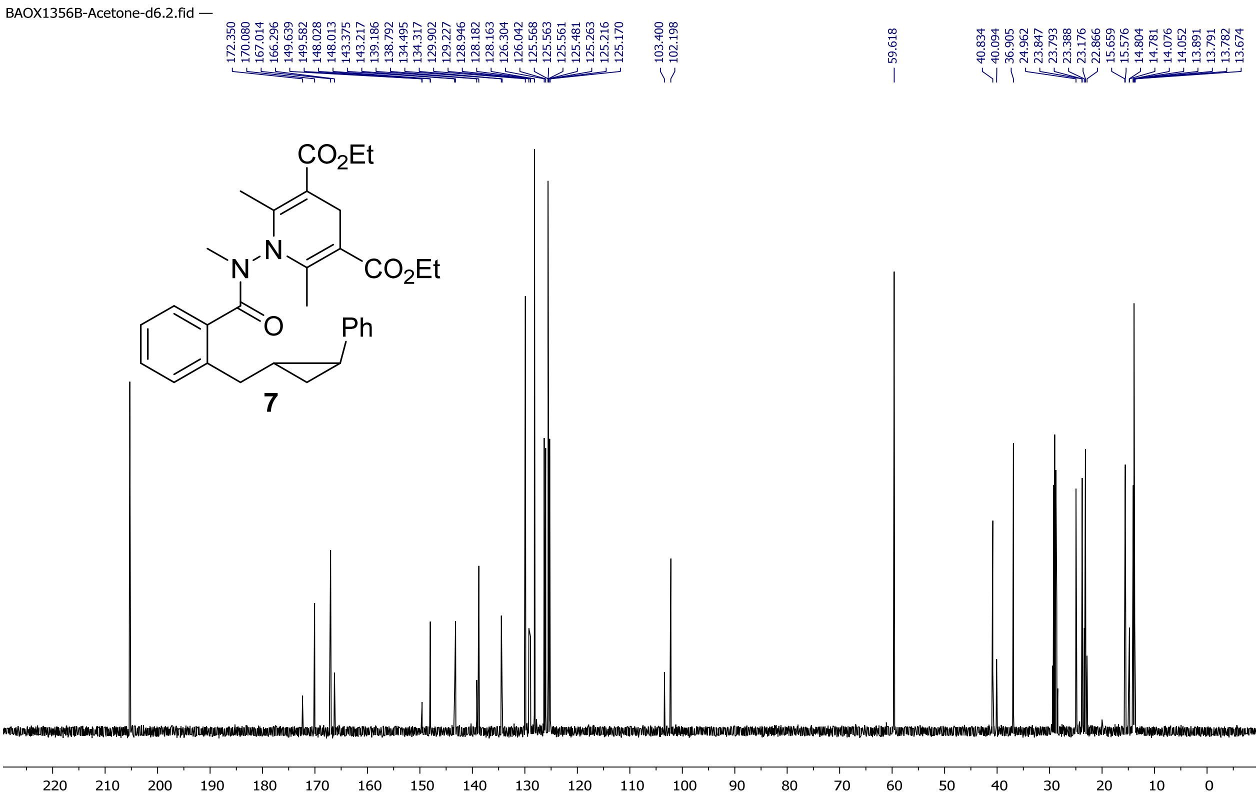

Supplementary Figure 113. ^{13}C NMR spectra of 7

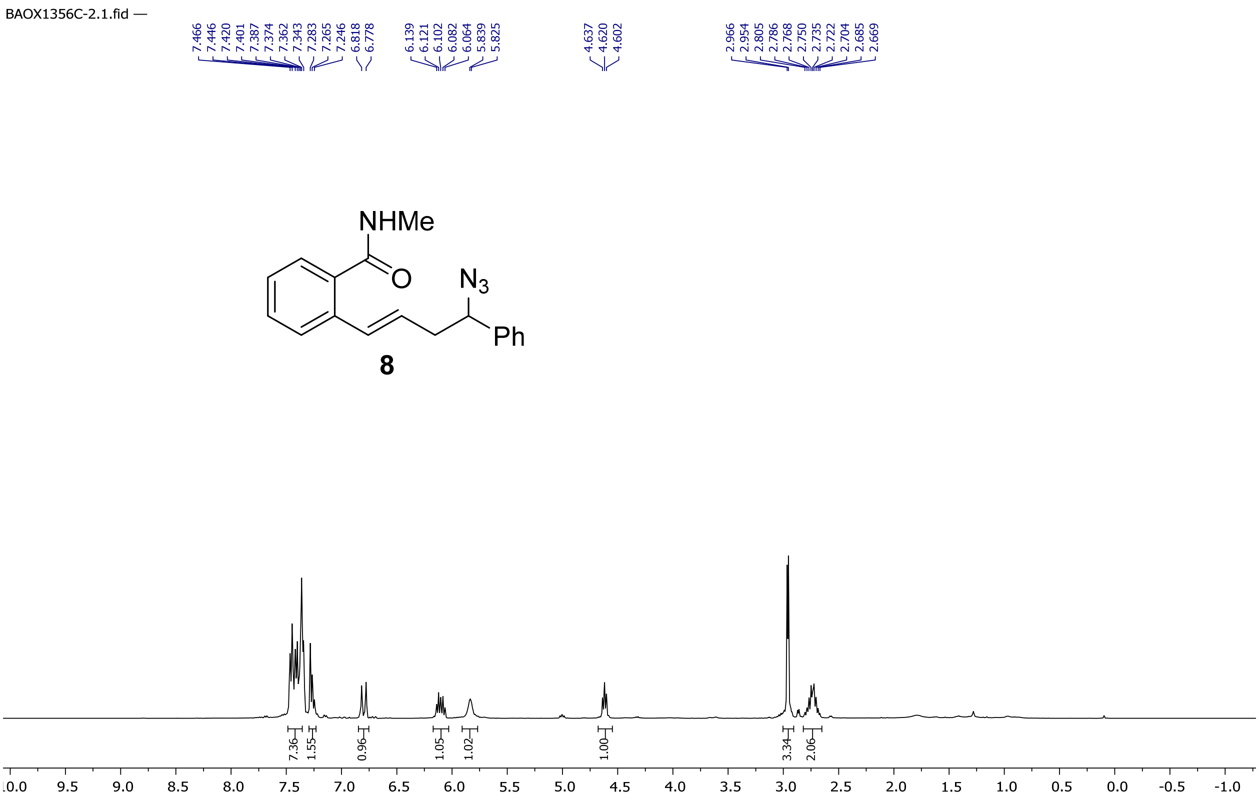

Supplementary Figure 114. ^{1}H spectra of 8

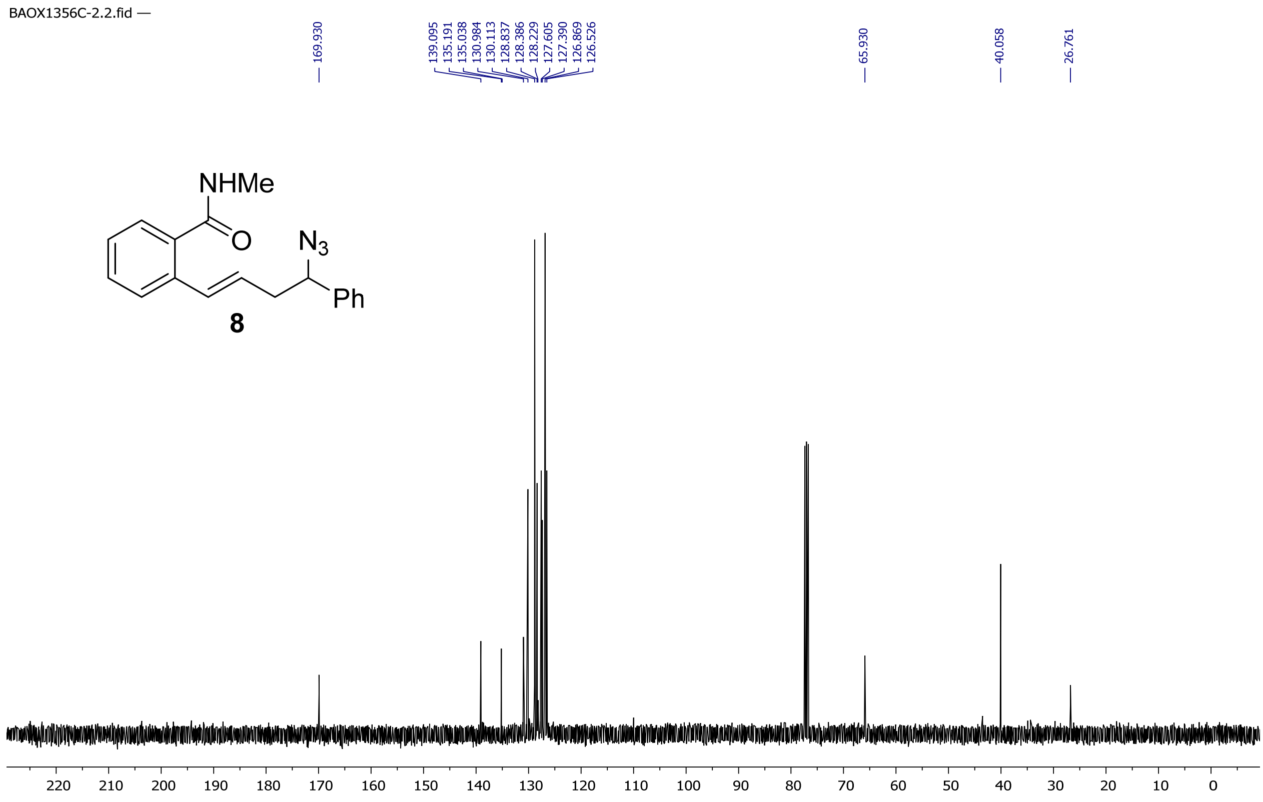

Supplementary Figure 115. ^{13}C NMR spectra of 8

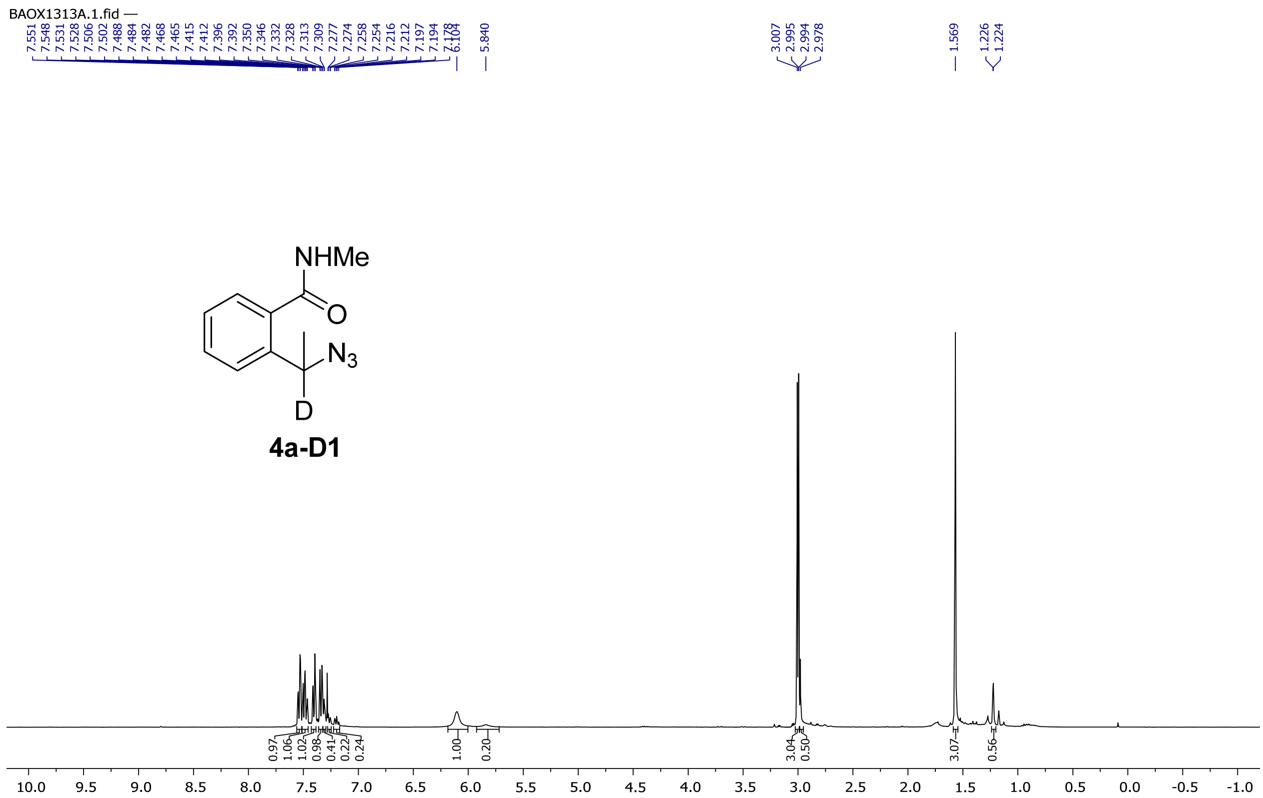

Supplementary Figure 116. ^{1}H spectra of 4a-D1

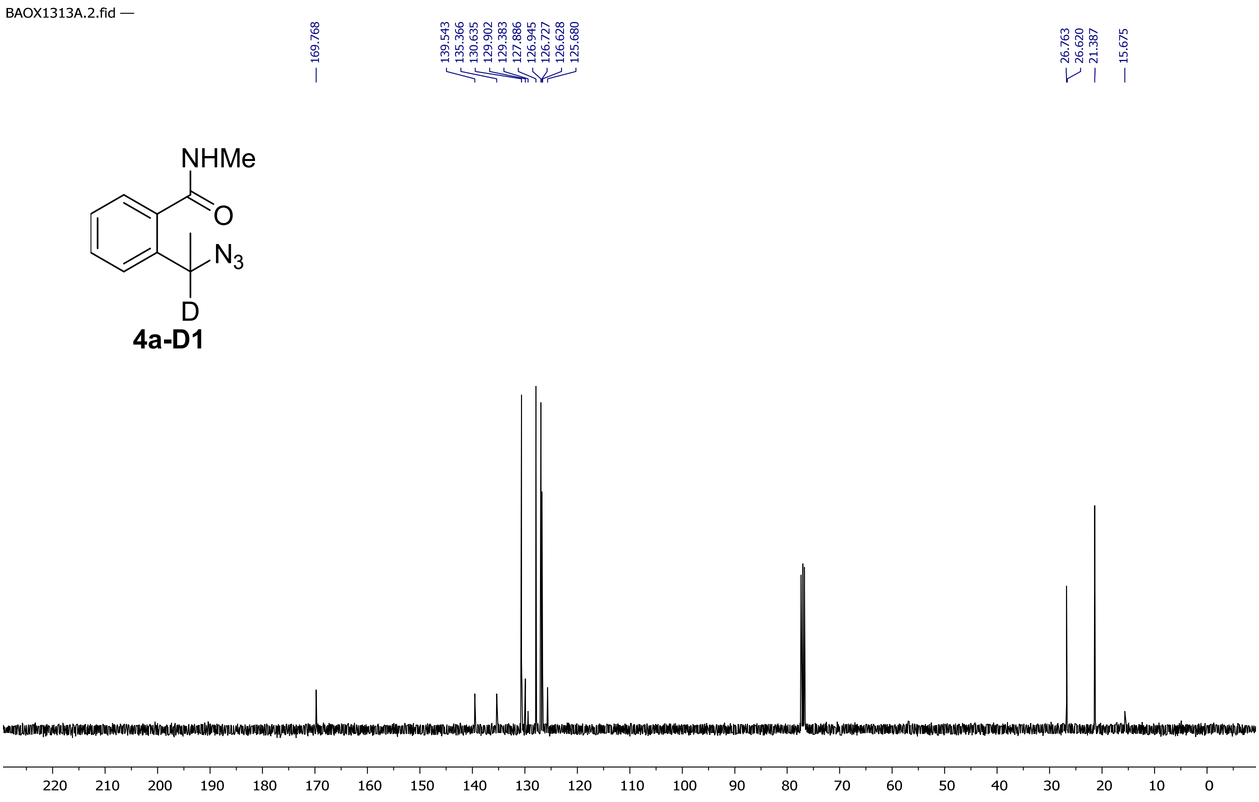

Supplementary Figure 117. ^{13}C NMR spectra of 4a-D1

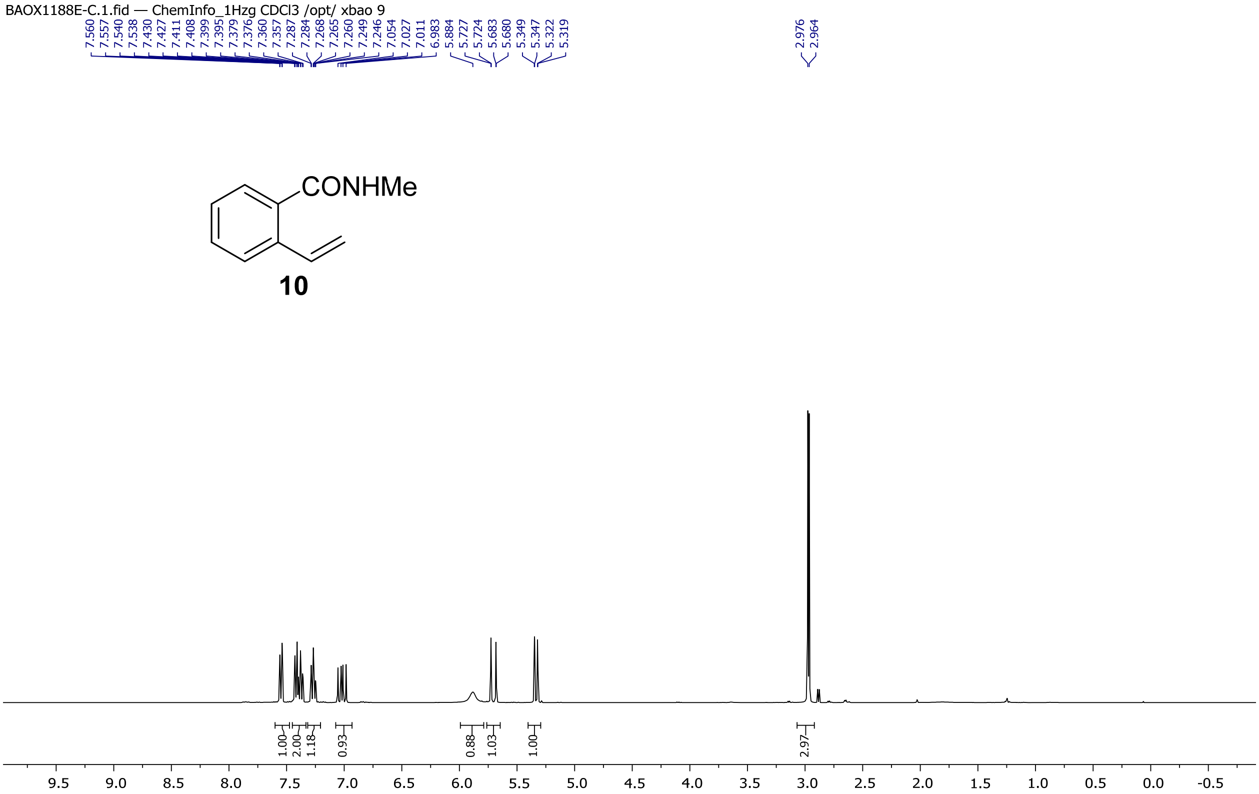

Supplementary Figure 118. ^{1}H spectra of 10

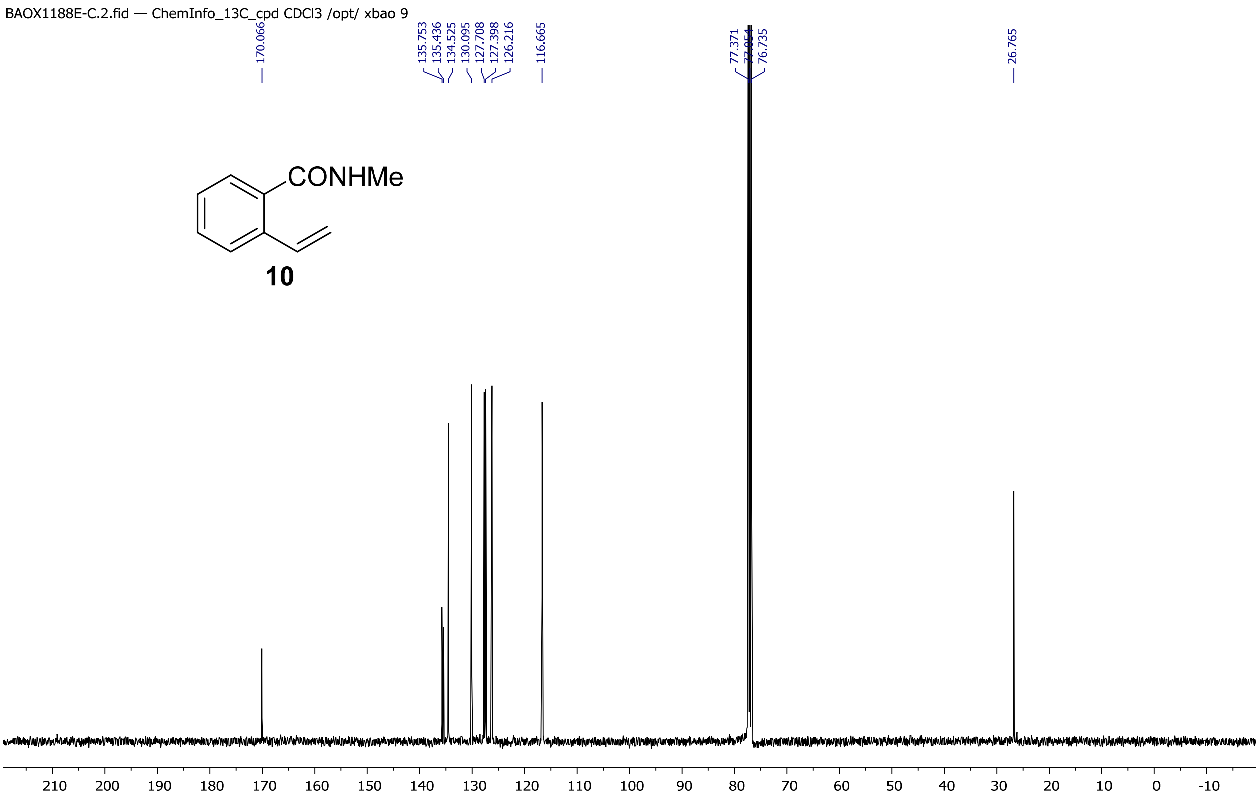

Supplementary Figure 119. ^{13}C NMR spectra of 10

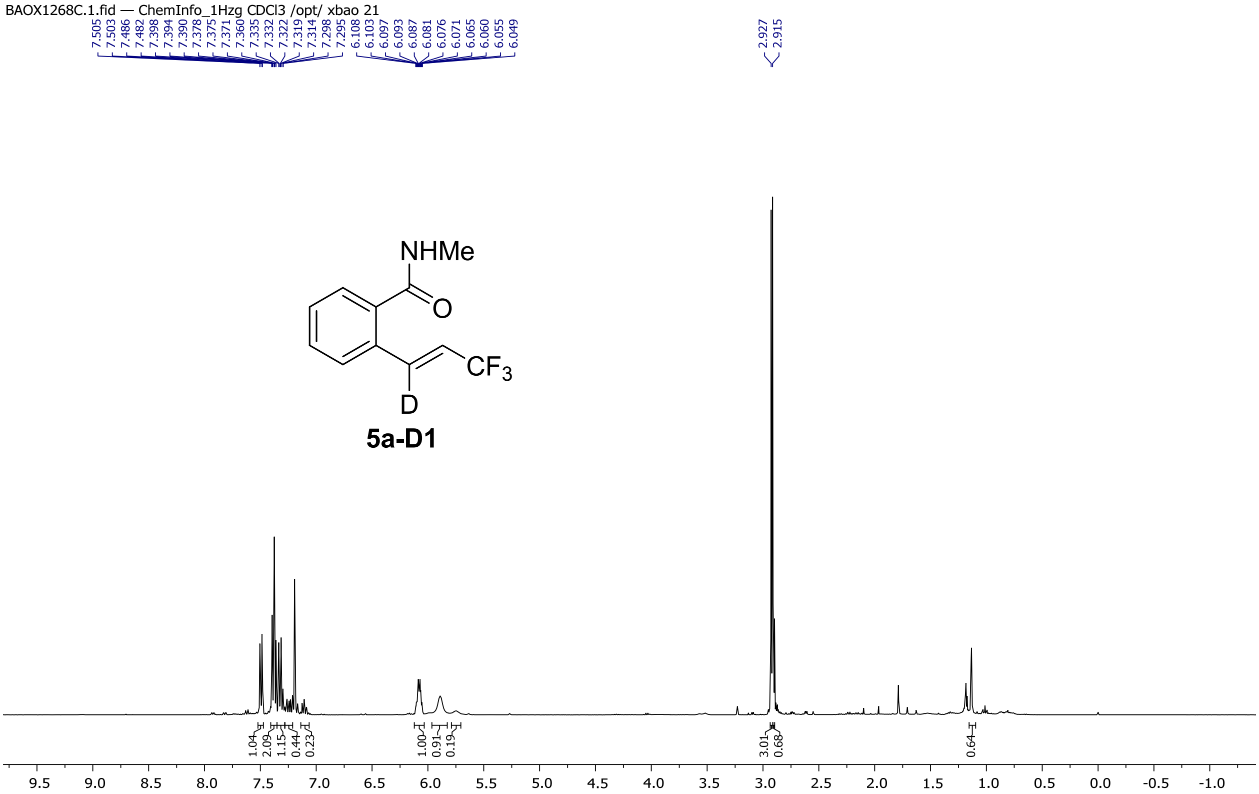

Supplementary Figure 120. ^{1}H spectra of 5a-D1

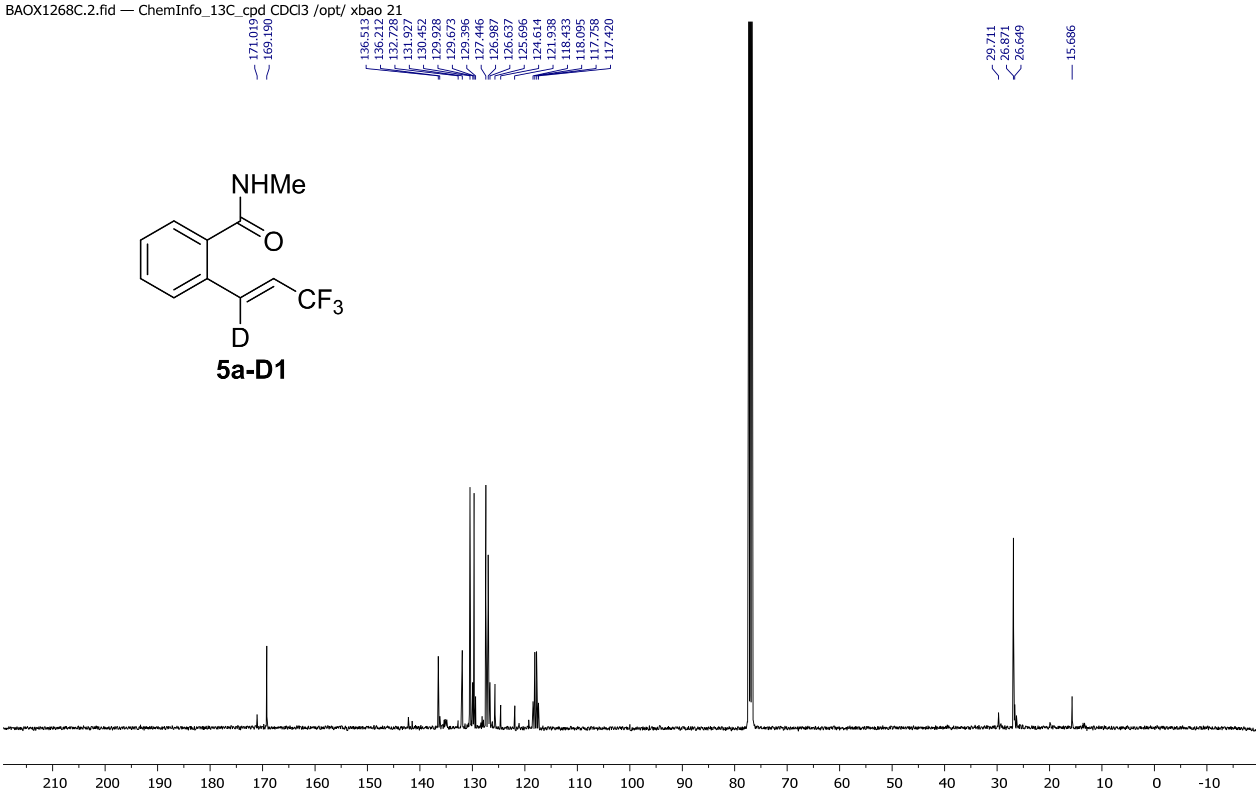

Supplementary Figure 121. ^{13}C NMR spectra of 5a-D1

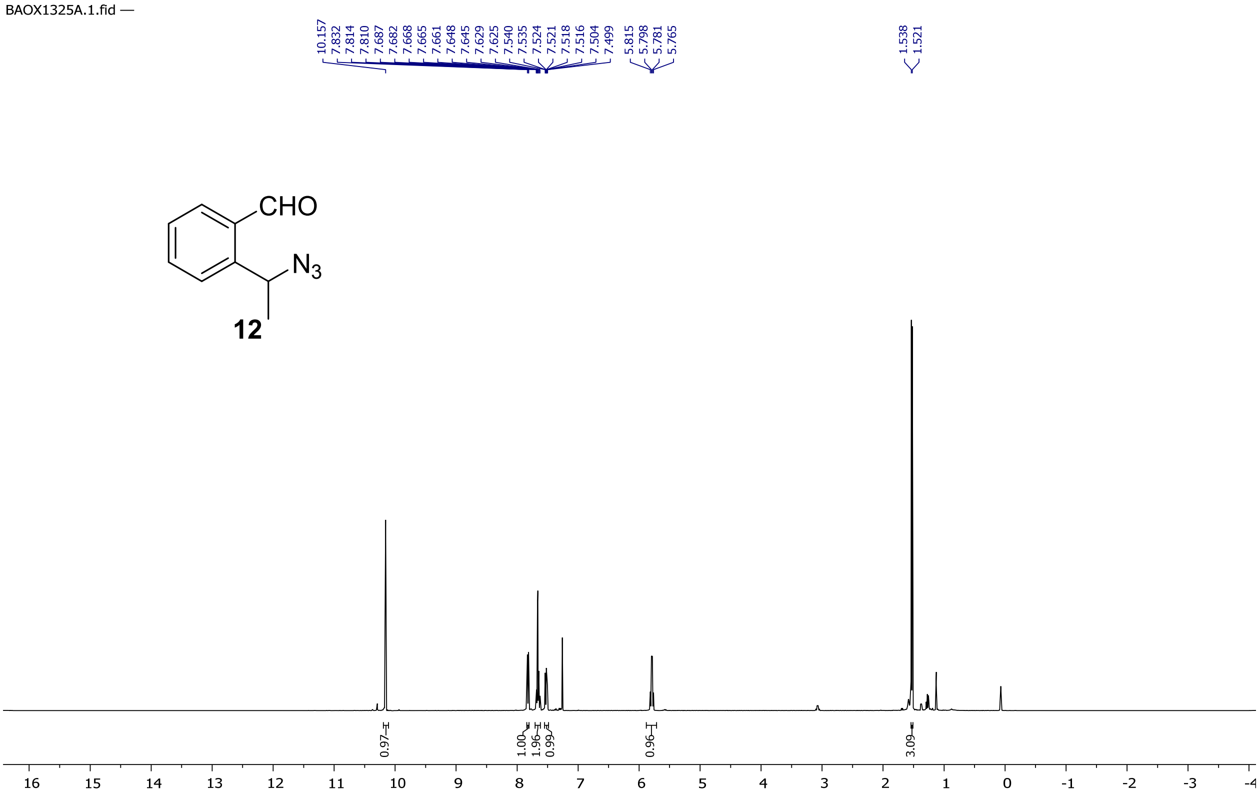

Supplementary Figure 122. ^{1}H spectra of 12

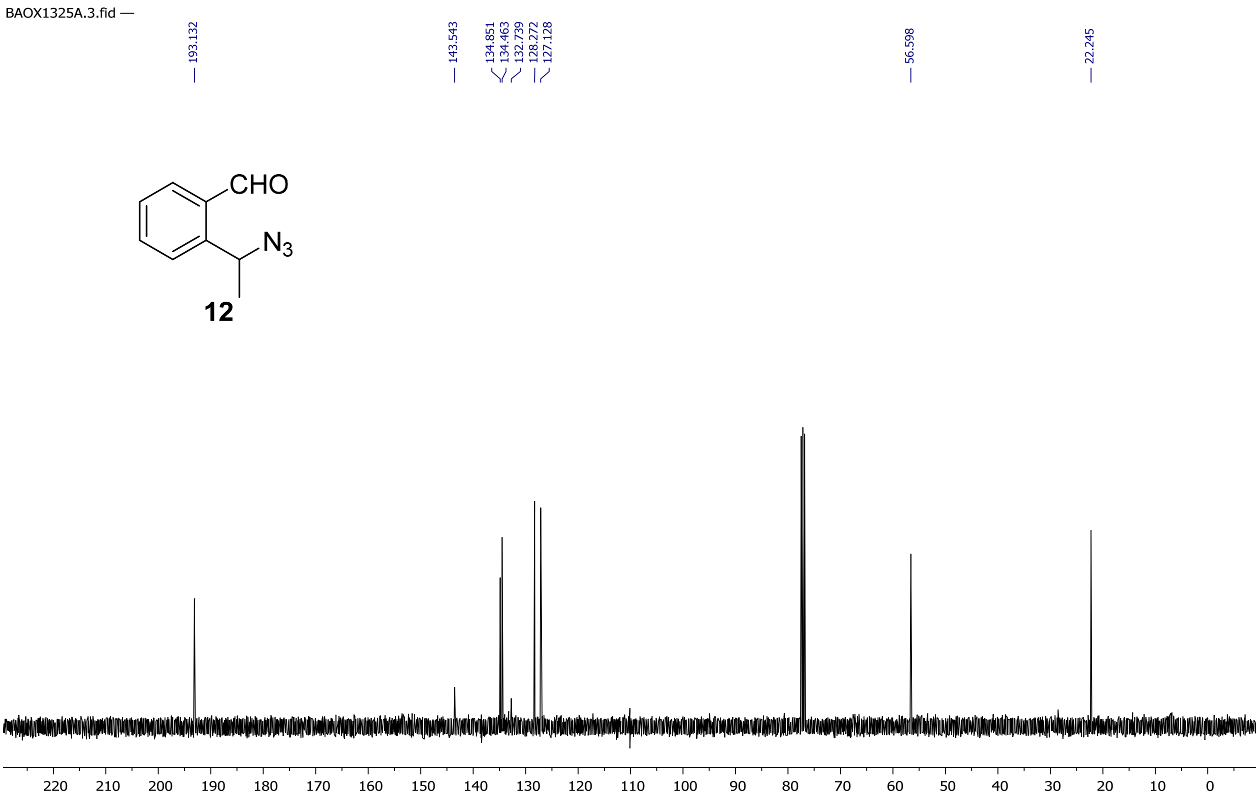

Supplementary Figure 123. ^{13}C NMR spectra of 12

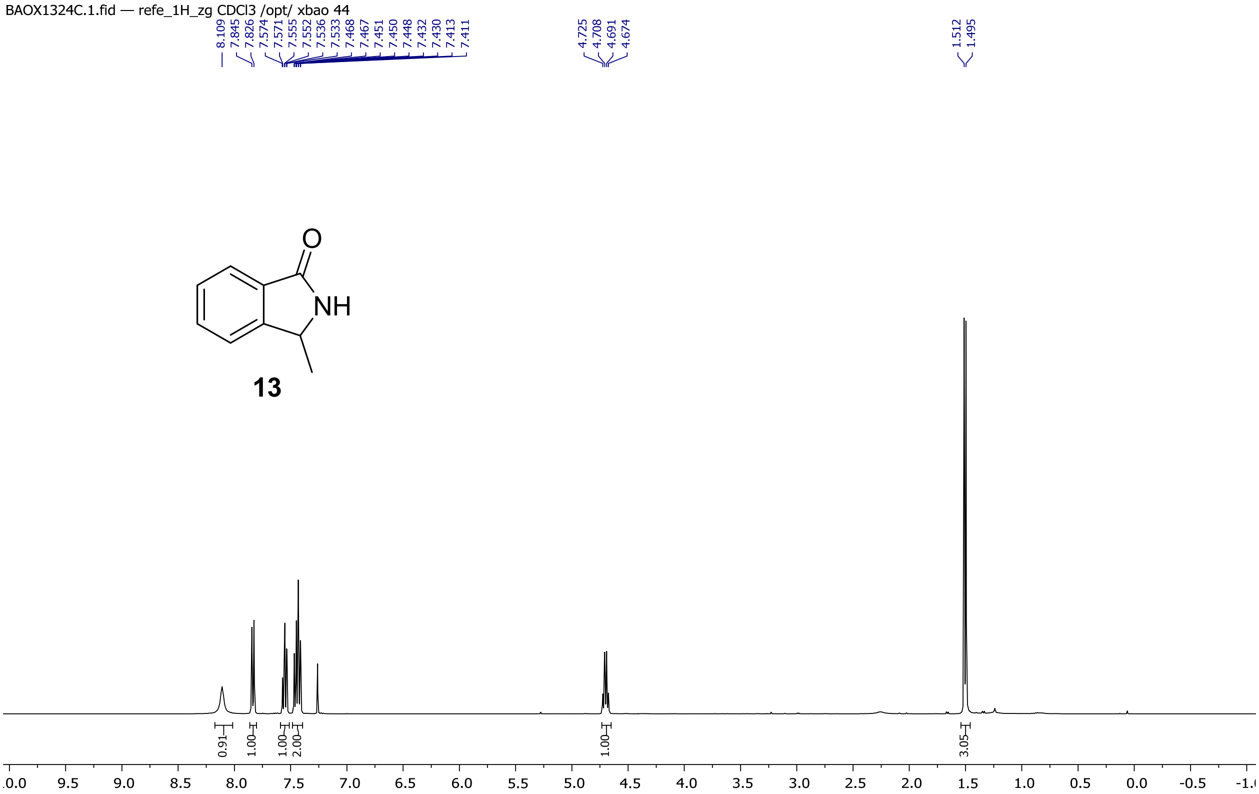

Supplementary Figure 124. ^{1}H spectra of 13

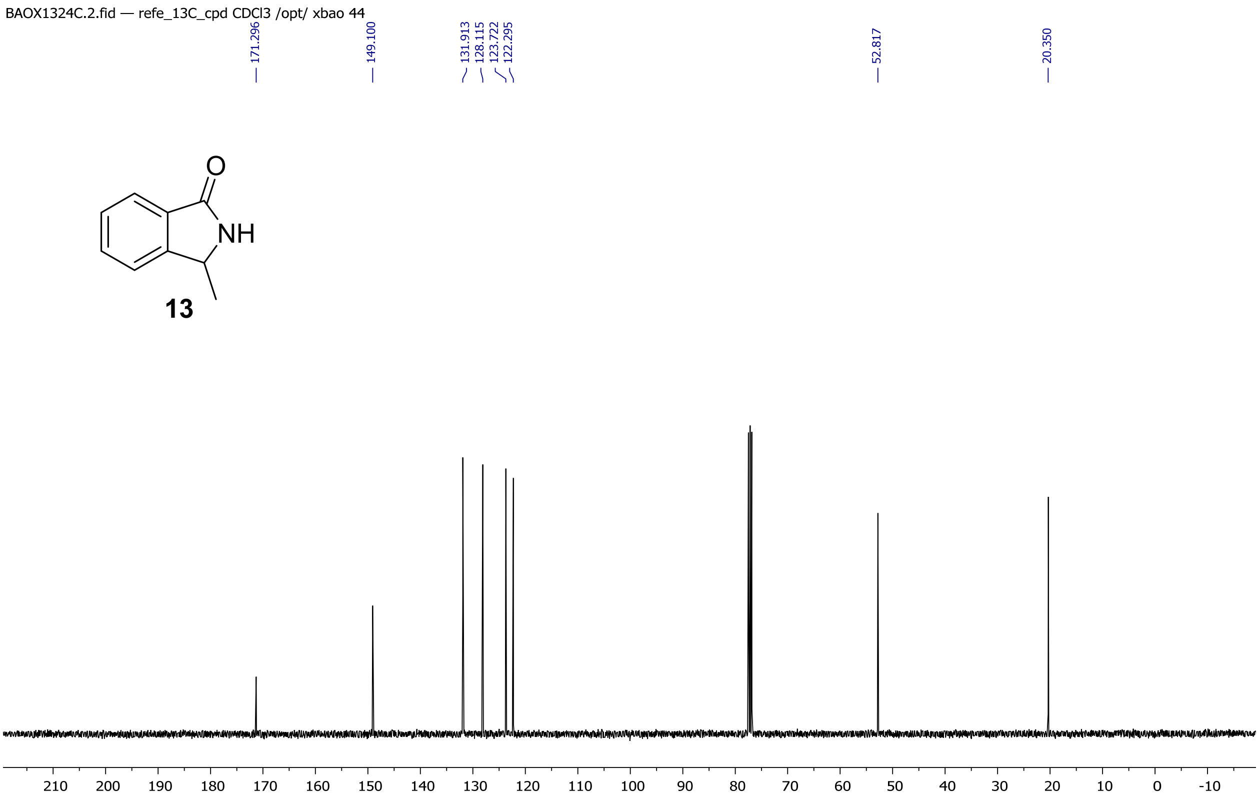

Supplementary Figure 125. ^{13}C NMR spectra of 13

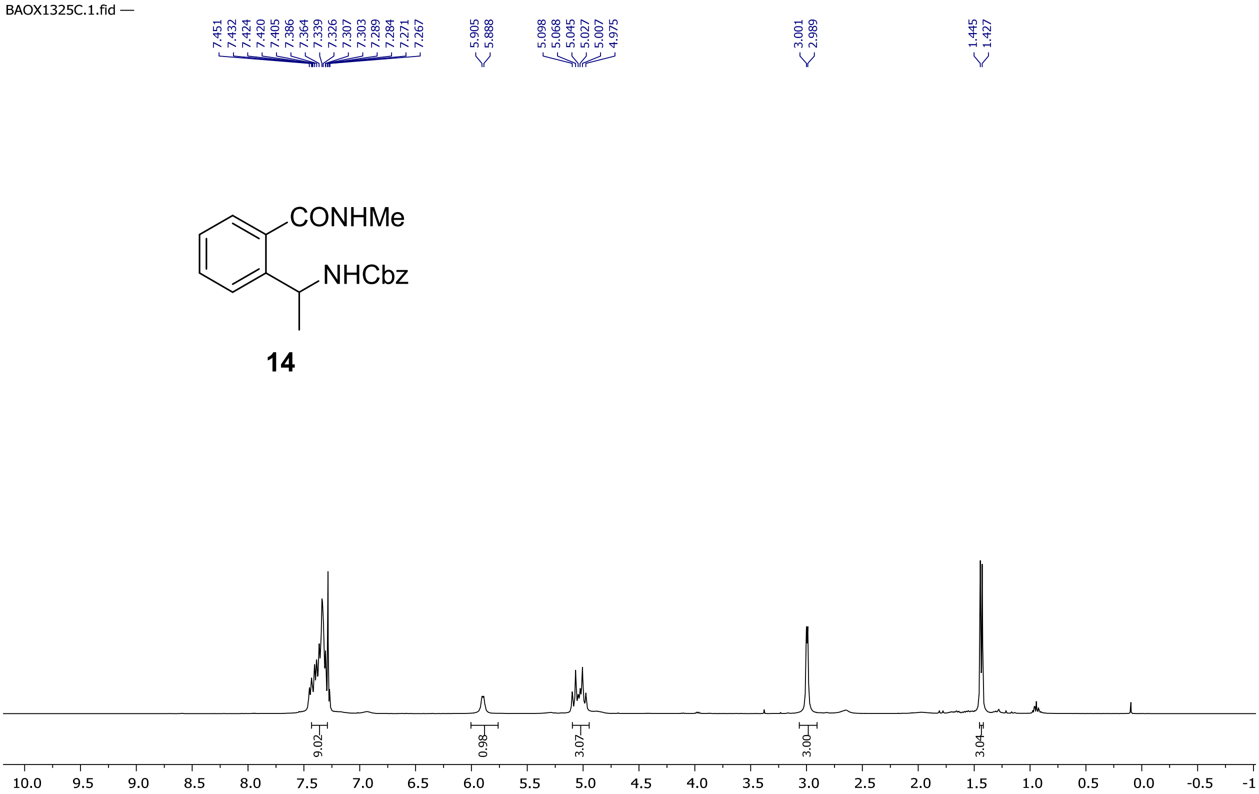

Supplementary Figure 126. ^{1}H spectra of 14

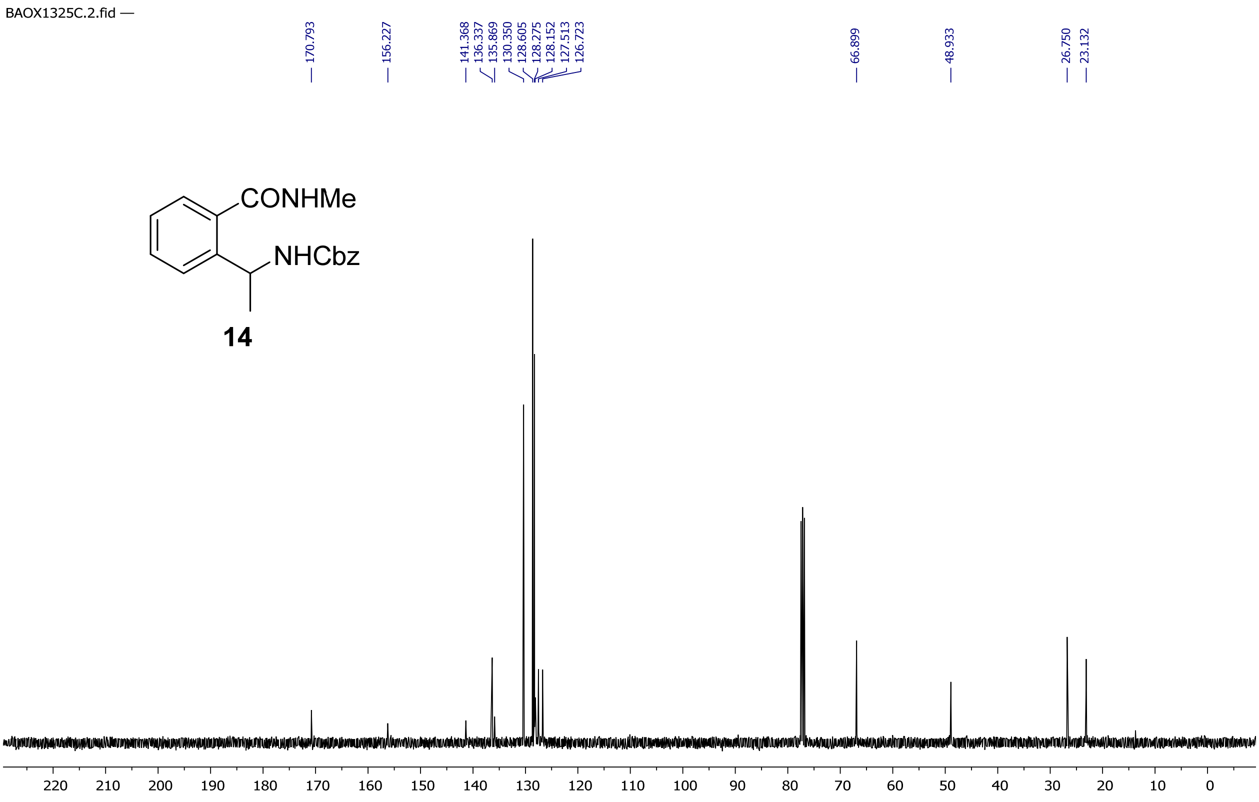

Supplementary Figure 127. ^{13}C NMR spectra of 14

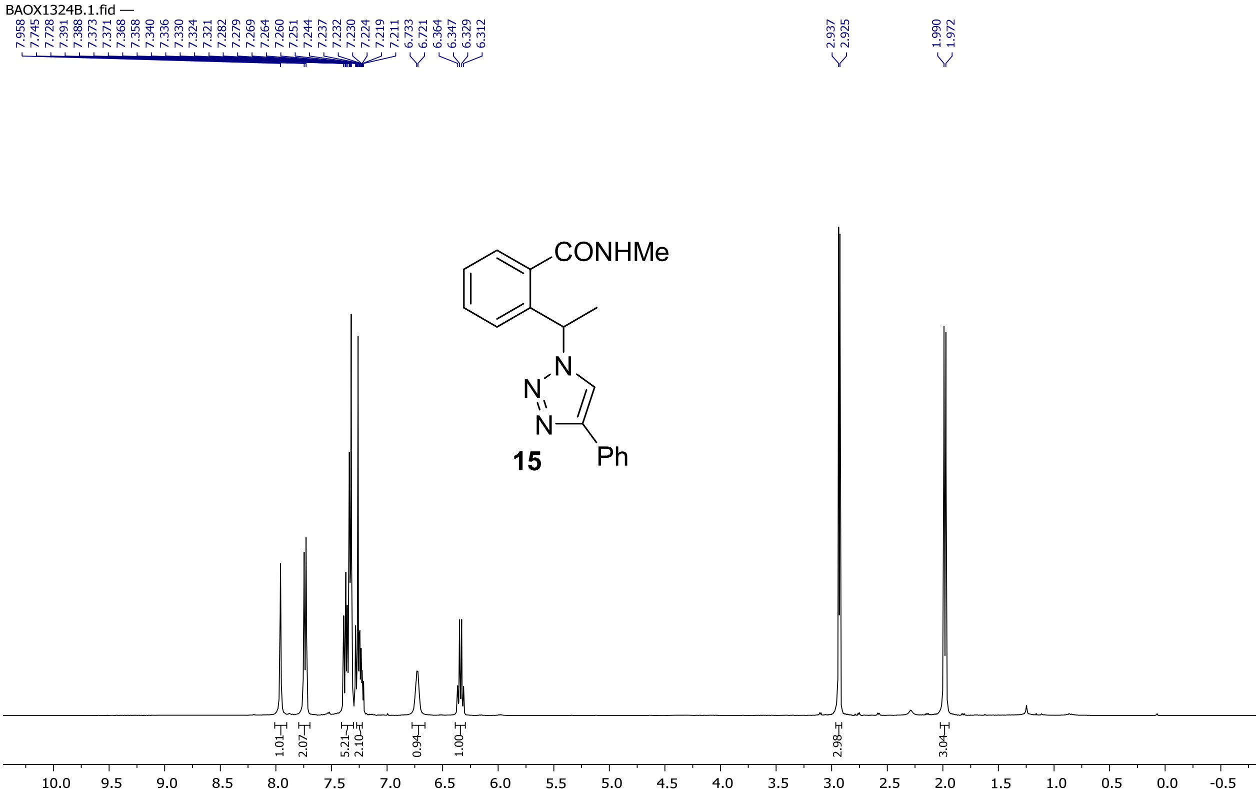

Supplementary Figure 128. ^{1}H spectra of 15

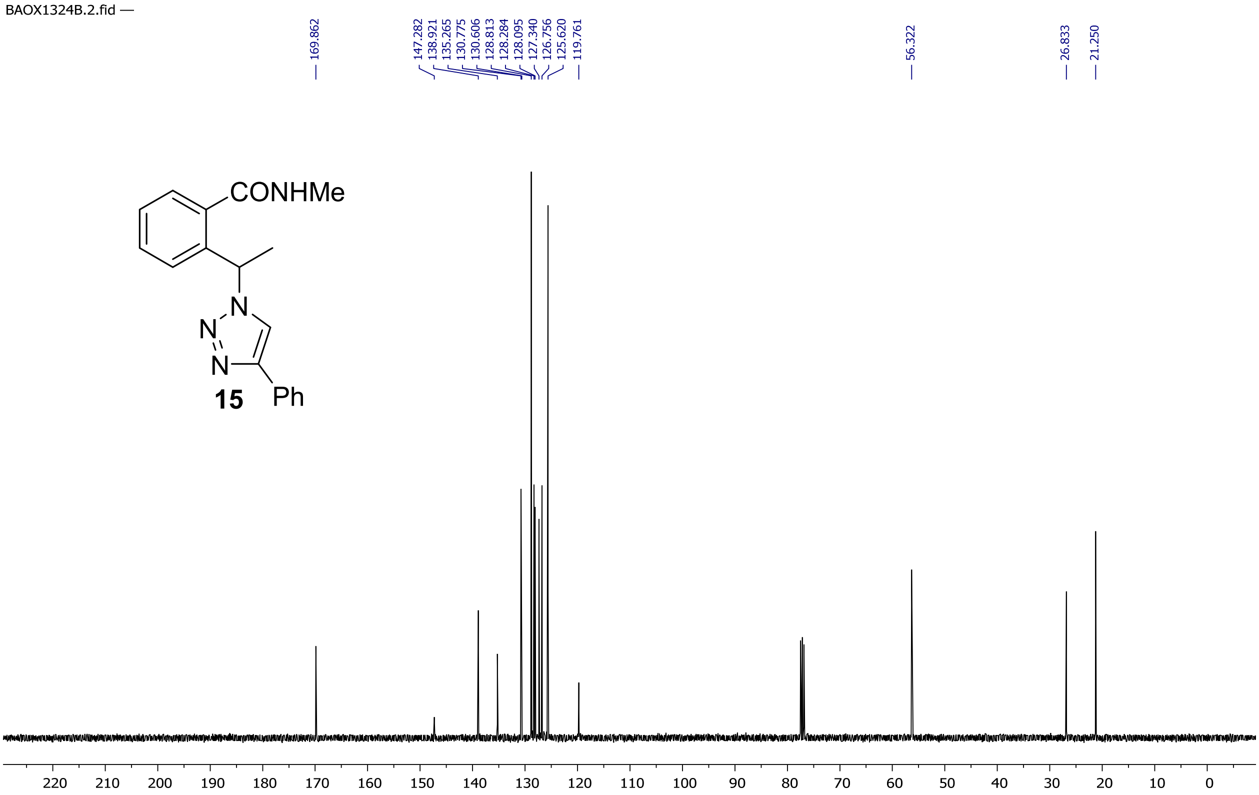

Supplementary Figure 129. ^{13}C NMR spectra of 14

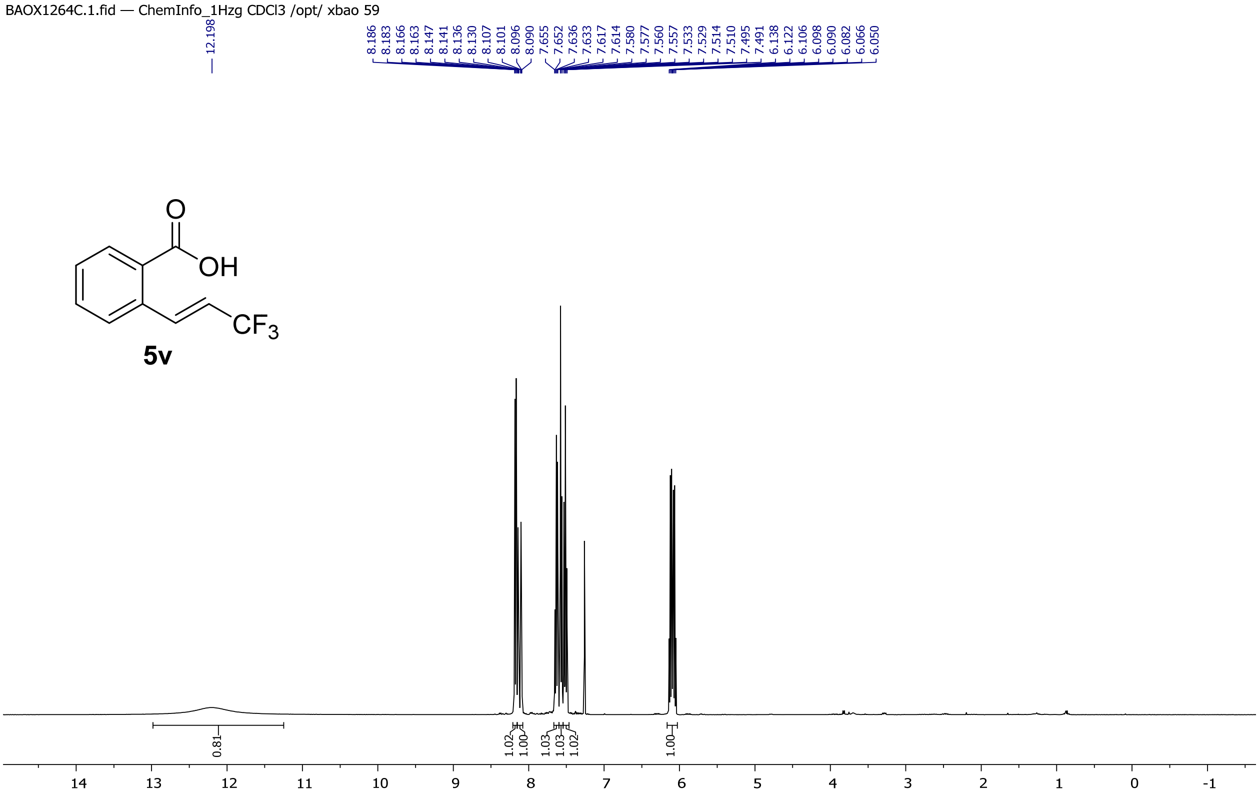

Supplementary Figure 130. ^{1}H spectra of 5v

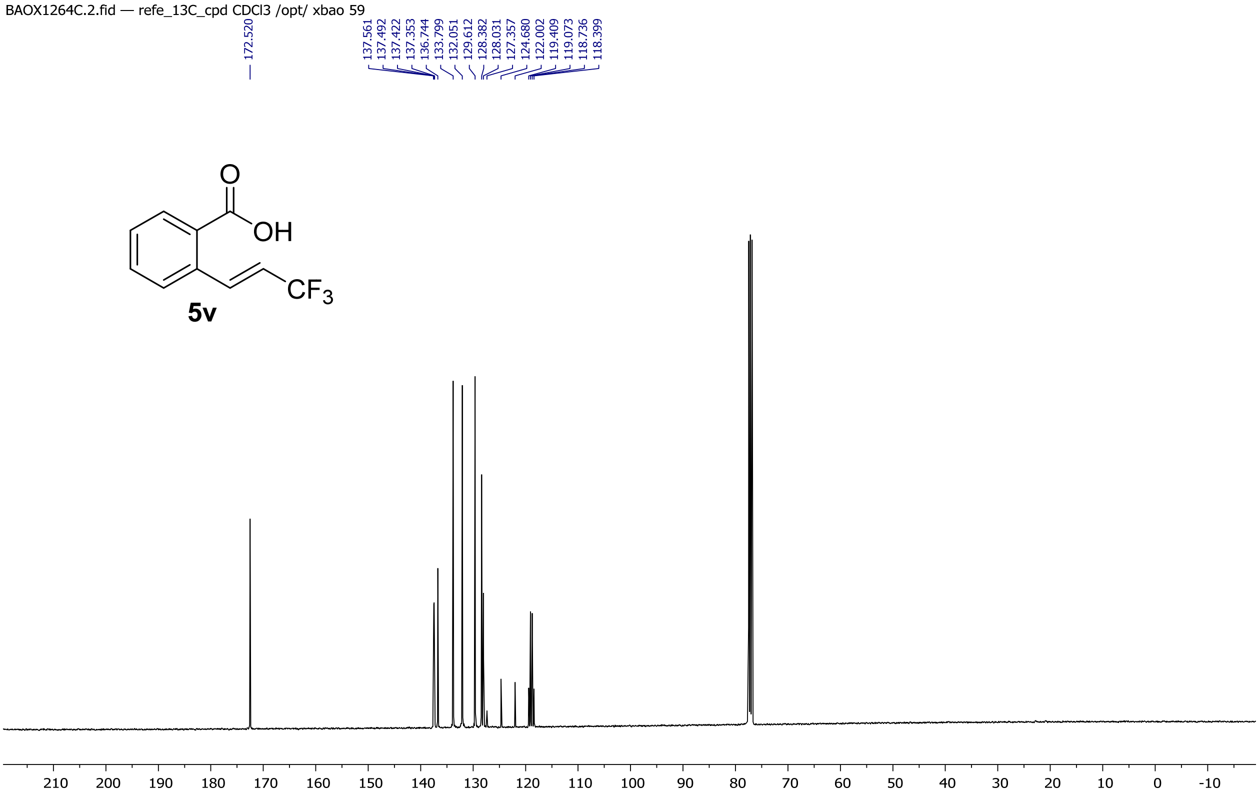

Supplementary Figure 131. ^{13}C NMR spectra of 5v

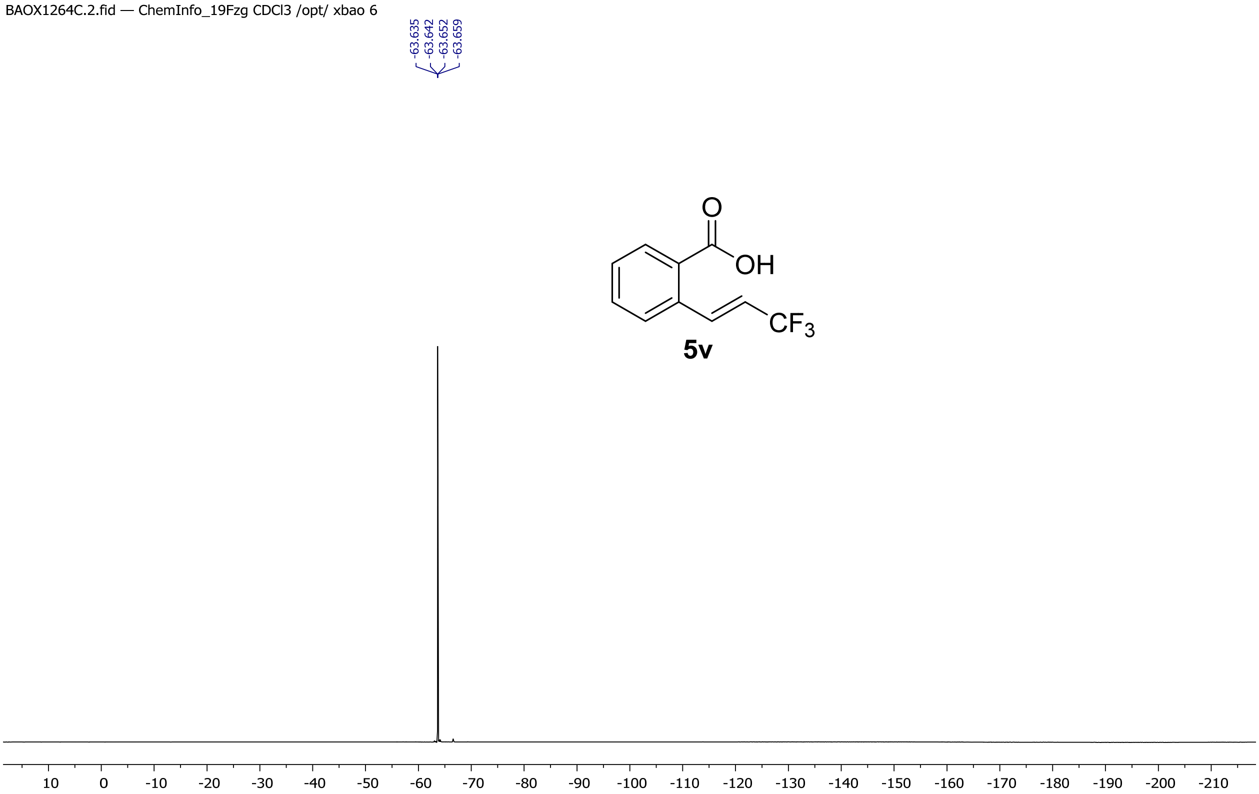

Supplementary Figure 132. ^{19}F spectra of 5v

Supplementary Tables

***Supplementary Table 1*: Copper-catalyzed γ–azidation of 2-ethyl benzohydrazide 3a: Cu(II) salts and organic peroxides screening.[a]**

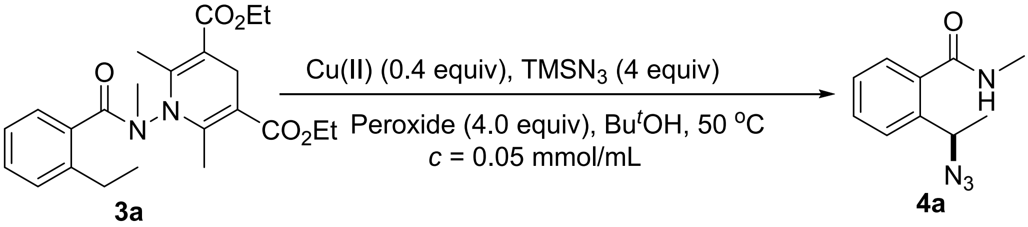

Entry	Cu(II)	Peroxide	Yield/%[b]
1	$Cu(OAc)_2$	TBHP	58
2	$Cu(OTf)_2$	TBHP	40
3	$CuSO_4$	TBHP	56
4	CuF_2	TBHP	56
5	$Cu(acac)_2$	TBHP	62
6	$Cu(ClO_4)_2•6H_2O$	TBHP	72
7	$Cu(BF_4)_2•6H_2O$	TBHP	60
8	$Cu(NO_3)_2•2H_2O$	TBHP	64
9	$CuBr_2$	TBHP	44
10	$Cu(ClO_4)_2•6H_2O$	DTBP	trace
11	$Cu(ClO_4)_2•6H_2O$	H_2O_2	trace
12	$Cu(ClO_4)_2•6H_2O$	CHP	64
13	$Cu(ClO_4)_2•6H_2O$	TBPB	60
14	$Cu(ClO_4)_2•6H_2O$	*t*BuOOAc	82
15	$Cu(ClO_4)_2•6H_2O$	DCP	trace

[a] **3a** (0.1 mmol, 1.0 equiv), $TMSN_3$ (4.0 equiv), Cu(II) (0.4 equiv), peroxide (4.0 equiv), and *t*BuOH (2.0 mL) at 50 °C. [b] Yield was determined by ^{1}H NMR spectroscopy with CH_2Br_2 as an internal standard.

***Supplementary Table 2*: Copper-catalyzed γ–azidation of 2-ethyl benzohydrazide 3a: solvents screening.**[a]

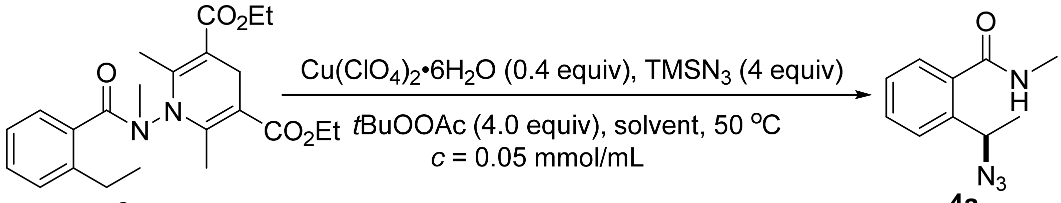

Entry	Solvent	Yield/%[b]
1	*t*BuOH	82
2	DCE	52
3	1,4-dioxane	52
4	EtOAc	36
5	CF_3CH_2OH	24
6	CH_3CN	48

[a] **3a** (0.1 mmol, 1.0 equiv), $TMSN_3$ (4.0 equiv), $Cu(ClO_4)_2$•$6H_2O$ (0.4 equiv), *t*BuOOAc (4.0 equiv), and solvent (2.0 mL) at 50 °C. [b] Yield was determined by ^1H NMR spectroscopy with CH_2Br_2 as an internal standard.

***Supplementary Table 3*: Copper-catalyzed γ–azidation of 2-ethyl benzohydrazide 3a: Cu(I) salts screening.**[a]

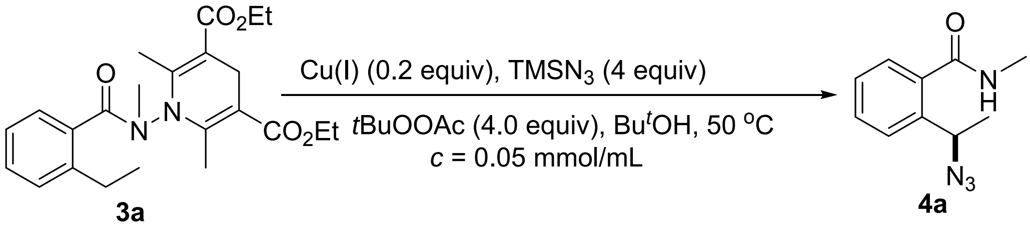

Entry	Cu(I)	Yield/%[b]
1	CuOAc	56
2	CuOTf•benzene	52
3	CuCl	44
4	CuI	53
5	CuBr	48
6	$CuPF_6$•$4CH_3CN$	68
7[c]	$CuPF_6$•$4CH_3CN$	88/(85)[d]

[a] **3a** (0.1 mmol, 1.0 equiv), $TMSN_3$ (4.0 equiv), Cu(I) (0.4 equiv), *t*BuOOAc (4.0 equiv) and Bu^tOH (2.0 mL) at 50 °C. [b] Yield was determined by ^1H NMR spectroscopy with CH_2Br_2 as an internal standard. [c] $TMSN_3$ (2.0 equiv), *t*BuOOAc (2.0 equiv). [d] Yield of isolated product in parenthesis.

***Supplementary Table 4*: Copper-catalyzed oxidative δ-trifluoromethylation of 2-ethylbenzohydrazide 3a: metal salts screening.**[a]

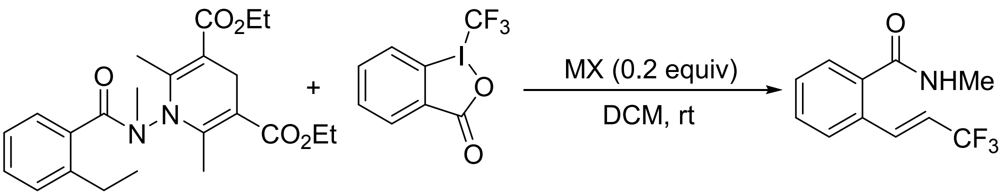

3a **9** **5a**

Entry	Metal salts	Yield/%[b]
1	CuF_2	trace
2	$Cu(OTf)_2$	trace
3	$Cu(OAc)_2$	trace
4	CuI	61
5	CuBr	54
6	CuCl	64
7	CuOAc	22
8	CuOTf•benzene	18
9	$FeCl_2$	trace
10	$FeBr_2$	trace
11	$Fe(OTf)_2$	trace
12	$FeCl_3$	trace
13	$Fe(OTf)_3$	trace

[a] **3a** (0.1 mmol, 1.0 equiv), Togni's reagent **9** (3.0 equiv), MX (0.2 equiv), and DCM (1.0 mL) at rt. [b] Yield was determined by ^{1}H NMR spectroscopy with DMAP as an internal standard.

***Supplementary Table 5*: Copper-catalyzed oxidative δ-trifluoromethylation of 2-ethylbenzohydrazide 3a: bases screening.**[a]

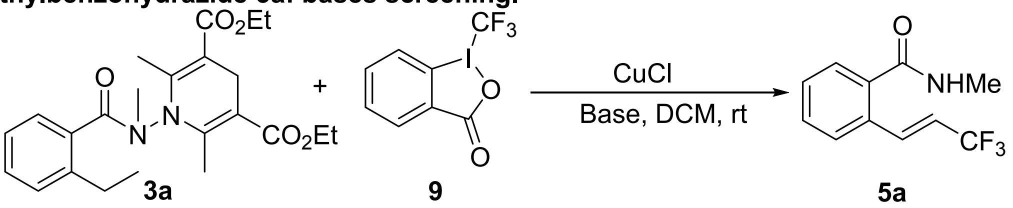

Entry	Base	Yield/%[b]
1	---	64
2	$NaHCO_3$	61
3	Na_2CO_3	68
4	K_2CO_3	24
5	Na_3PO_4	50
6	Na_2HPO_4	54
7	DABCO	36
8[c]	Na_2CO_3	74(72)[d]
9[f]	Na_2CO_3	54

[a] **3a** (0.1 mmol, 1.0 equiv), Togni's reagent **9** (3.0 equiv), CuCl (0.2 equiv mmol), base (3.0 equiv), and DCM (1.0 mL) at rt. [b] Yield was determined by ^{1}H NMR spectroscopy with DMAP as an internal standard. [c] CuCl (0.4 equiv) [d] Yield of isolated product in parenthesis. [f] CuCl (0.06 mmol).

***Supplementary Table 6*: Copper-catalyzed oxidative δ-trifluoromethylation of 2-ethylbenzohydrazide 3a: solvents screening.**[a]

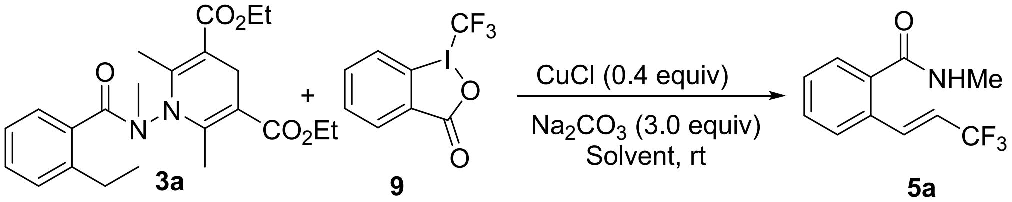

Entry	Solvent	Yield/%[b]
1	Benzene	trace
2	PhCl	trace
3	$PhCF_3$	trace
4	CH_3CN	trace
5	DCE	61
6	1,4-Dioxane	12
7	DCM	74/(72)[c]

[a] **3a** (0.1 mmol, 1.0 equiv), Togni's reagent **9** (3.0 equiv), CuCl (0.4 equiv), Na_2CO_3 (3.0 equiv), and solvent (1.0 mL) at rt. [b] Yield was determined by ^{1}H NMR spectroscopy with DMAP as an internal standard. [d] Yield of isolated product in parenthesis.

Supplementary Methods

General Information.

General Analytical Information.

NMR spectra were recorded on a Brüker AvanceIII-400, Brüker Avance-400 or Brüker DPX-400 spectrometer at room temperature, ^{1}H frequency is at 400.13 MHz, ^{13}C frequency is at 100.62 MHz. Chemical shifts (δ) were reported in parts per million (ppm) relative to residual solvent peaks rounded to the nearest 0.01 for proton and 0.1 for carbon (*ref: CHCl3 [^{1}H: 7.26, ^{13}C: 77.16]*. Coupling constants (*J)* were reported in Hz to the nearest 0.1 Hz. Peak multiplicity was indicated as follows s (singlet), d (doublet), t (triplet), q (quartet), m (multiplet) and br (broad). Attribution of peaks was done using the multiplicities and integrals of the peaks.

IR spectra were recorded in a Jasco FT/IR-4100 spectrometer outfitted with a PIKE technology MIRacleTM ATR accessory as neat films compressed onto a Zinc Selenide window. The spectra were reported in cm^{-1}.

The accurate masses were measured by the mass spectrometry service of the EPFL by ESI-TOF using a QTOF Ultima from Waters or APPI-FT-ICR using a linear ion trap Fourier transform ion cyclotron resonance mass spectrometer from Thermo Scientific.

Melting points were measured using a Stuart SMP30

Materials and Methods.

Unless otherwise stated, starting materials were purchased from Aldrich and/or Fluka. Solvents were purchased in HPLC quality, degassed by purging thoroughly with nitrogen and dried over activated molecular sieves of appropriate size. Alternatively, they were purged with argon and passed through alumina columns in a solvent purification system (Innovative Technology). Conversion was monitored by thin layer chromatography (TLC) using Merck TLC silica gel 60 F254. Compounds were visualized by UV light at 254 nm and by dipping the plates in an ethanolic vanillin/sulfuric acid solution or an

aqueous potassium permanganate solution followed by heating. Flash column chromatography was performed over silica gel (230–400 mesh).

General procedure

The benzohydrazides **3** were prepared following Studer's procedure.[1]

General procedure for the Copper-catalyzed γ-azidation of 2-alkyl benzohydrazides 3

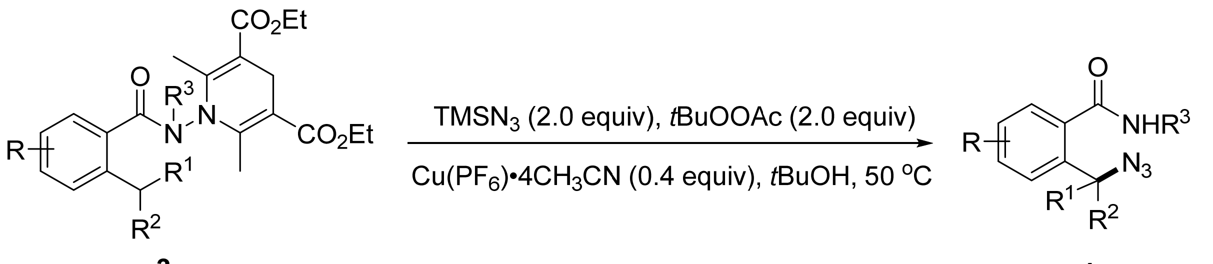

3 **4**

A screw cap tube was charged with $Cu(PF_6)$•$4CH_3CN$ (14.9 mg, 0.04 mmol, 0.4 equiv), substrate **3** (0.1 mmol, 1.0 equiv), $TMSN_3$ (26.5 µl, 0.2 mmol, 2.0 equiv) and *t*BuOH (2.0 mL). The mixture was stirred at 50 °C for 2 minutes, then *t*BuOOAc (32.2 µL, 0.2 mmol, 2.0 equiv) was added to the above mixture. After being stirred for 2 hours at 50 °C under N_2 atmosphere, the reaction mixture was quenched with water, extracted with EtOAc. The organic extracts were washed with brine, dried over Na_2SO_4. The solvent was removed under reduced pressure. The residue was purified by flash chromatography (SiO_2, eluent: PE/EtOAc = 2/1) to give **4.**

Characterization of compounds 4

2-(1-azidoethyl)-*N*-methylbenzamide (4a)

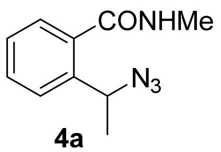

4a

The crude product was purified by flash column chromatography on silica gel to afford **4a** (17.4 mg, 85% yield) as an oil.

¹H NMR (400 MHz, $CDCl_3$) *δ* 7.44 (d, *J* = 7.8 Hz, 1H), 7.38 (t, *J* = 7.5 Hz, 1H), 7.29 (d, *J* = 7.7 Hz, 1H), 7.25 – 7.17 (m, 1H), 6.17 (s, 1H), 5.08 (q, *J* = 6.8 Hz, 1H), 2.87 (d, *J* = 4.9 Hz, 3H), 1.46 (d, *J* = 6.8 Hz, 3H).

¹³C NMR (101 MHz, $CDCl_3$) *δ* 169.9, 139.7, 135.4, 130.7, 127.9, 127.0, 126.8, 57.4, 26.8, 21.7.

IR (neat) cm^{-1} υ: 3294 (w), 2976 (w), 2104 (s), 1721 (w), 1635 (s), 1619 (m), 1538 (s), 1410 (m), 1305 (m), 1245 (s), 1065 (m), 760 (s), 697 (m).

HRMS (ESI) m/z: $[M + Na]^+$ Calcd for $C_{10}H_{12}N_4NaO^+$ 227.0903; Found 227.0906.

2-(1-azidoethyl)-*N*,4-dimethylbenzamide (4b)

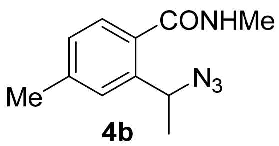

4b

The crude product was purified by flash column chromatography on silica gel to afford **4b** (18.5 mg, 85% yield) as an oil.

¹H NMR (400 MHz, $CDCl_3$) *δ* 7.24 (s, 1H), 7.20 (d, *J* = 7.9 Hz, 1H), 7.03 (d, *J* = 7.1 Hz, 1H), 6.01 (s, 1H), 5.12 (q, *J* = 6.7 Hz, 1H), 2.89 (d, *J* = 4.9 Hz, 3H), 2.31 (s, 3H), 1.47 (d, *J* = 6.7 Hz, 3H).

¹³C NMR (101 MHz, $CDCl_3$) *δ* 170.0, 141.0, 139.9, 132.5, 128.6, 127.4, 127.1, 57.4, 26.8, 21.8, 21.6.

IR (neat) cm^{-1} υ: 3262 (w), 2979 (w), 2112 (s), 2076 (m), 1625 (s), 1562 (s), 1452 (w), 1322 (m), 1249 (s), 1146 (m), 1071 (w), 836 (m), 720 (s).

HRMS (ESI) m/z: $[M + Na]^+$ Calcd for $C_{11}H_{14}N_4NaO^+$ 241.1060; Found 241.1059.

3-(1-azidoethyl)-*N*-methyl-[1,1'-biphenyl]-4-carboxamide (4c)

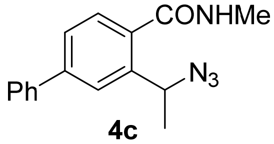

4c

The crude product was purified by flash column chromatography on silica gel to afford **4c** (25.8 mg, 92% yield) as an oil.

^{1}H NMR (400 MHz, $CDCl_3$) δ 7.73 (d, J = 1.8 Hz, 1H), 7.61 – 7.56 (m, 2H), 7.52 – 7.42 (m, 4H), 7.41 – 7.36 (m, 1H), 6.25 (q, J = 5.3 Hz, 1H), 5.26 (q, J = 6.7 Hz, 1H), 2.98 (d, J = 4.9 Hz, 3H), 1.59 (d, J = 6.8 Hz, 3H).

^{13}C NMR (101 MHz, $CDCl_3$) δ 169.7, 143.6, 140.5, 140.0, 134.0, 129.0, 128.1, 127.7, 127.3, 126.5, 125.6, 57.5, 26.9, 21.8.

IR (neat) cm^{-1} υ: 3269 (w), 2936 (w), 2100 (m), 1624 (m), 1562 (m), 1481 (w), 1450 (w), 1409 (w), 1307 (m), 1241 (m), 1069 (w), 838 (w), 761 (s), 696 (s).

HRMS (ESI) m/z: $[M + Na]^+$ Calcd for $C_{16}H_{16}N_4NaO^+$ 303.1216; Found 303.1216.

2-(1-azidoethyl)-4-methoxy-*N*-methylbenzamide (4d)

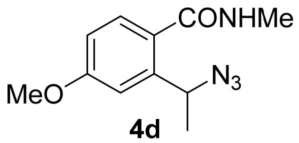

4d

The crude product was purified by flash column chromatography on silica gel to afford **4d** (19.8 mg, 85% yield) as a white solid.

MP: 74 – 75 °C

^{1}H NMR (400 MHz, $CDCl_3$) δ 7.35 (d, J = 8.5 Hz, 1H), 7.03 (d, J = 2.3 Hz, 1H), 6.80 (dd, J = 8.4, 2.4 Hz, 1H), 5.99 (s, 1H), 5.27 (q, J = 6.7 Hz, 1H), 3.84 (s, 3H), 2.96 (d, J = 4.8 Hz, 3H), 1.54 (d, J = 6.8 Hz, 3H).

^{13}C NMR (101 MHz, $CDCl_3$) δ 169.6, 161.4, 142.4, 128.8, 127.6, 113.0, 112.5, 57.4, 55.6, 26.9, 21.9.

IR (neat) cm^{-1} υ: 3259 (w), 2987 (w), 2110 (m), 2069 (w), 1631 (m), 1609 (m), 1570 (m), 1492 (w), 1409 (m), 1284 (s), 1229 (s), 1169 (w), 1066 (m), 1038 (s), 894 (w), 824 (m), 707 (m).

HRMS (ESI) m/z: $[M + Na]^+$ Calcd for $C_{11}H_{14}N_4NaO_2^+$ 257.1009; Found 257.1010.

2-(1-azidoethyl)-4-fluoro-*N*-methylbenzamide (4e)

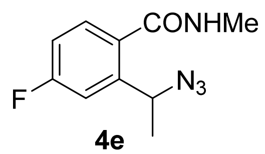

4e

The crude product was purified by flash column chromatography on silica gel to afford **4e** (20.2 mg, 90% yield) as an oil.

^{1}H NMR (400 MHz, $CDCl_3$) δ 7.36 (dd, J = 8.5, 5.6 Hz, 1H), 7.20 (dd, J = 9.9, 2.4 Hz, 1H), 6.96 (td, J = 8.1, 2.3 Hz, 1H), 6.20 (s, 1H), 5.17 (q, J = 6.8 Hz, 1H), 2.93 (d, J = 4.9 Hz, 3H), 1.51 (d, J = 6.8 Hz, 3H).

^{13}C NMR (101 MHz, $CDCl_3$) δ 169.0, 163.9 (d, J = 250.8 Hz), 143.2 (d, J = 7.4 Hz), 131.3 (d, J = 3.4 Hz), 129.2 (d, J = 8.6 Hz), 114.8 (d, J = 21.7 Hz), 114.1 (d, J = 22.8 Hz), 57.0 (d, ^4J = 1.4 Hz), 26.9, 21.7.

IR (neat) cm^{-1} υ: 3286 (w), 2935 (w), 2104 (s), 2091 (s), 1635 (s), 1542 (s), 1447 (m), 1409 (m), 1308 (m), 1259 (s), 1215 (m), 1127 (m), 1058 (w), 853 (w), 800 (m), 762 (m), 702 (s).

HRMS (ESI) m/z: $[M + Na]^+$ Calcd for $C_{10}H_{11}FN_4NaO^+$ 245.0809; Found 245.0811.

2-(1-azidoethyl)-4-bromo-*N*-methylbenzamide (4f)

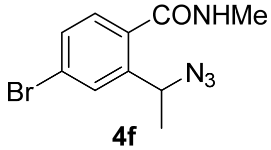

4f

The crude product was purified by flash column chromatography on silica gel to afford **4f** (23.3 mg, 83% yield) as a white solid.

MP: 107 – 108 °C

^{1}H NMR (400 MHz, $CDCl_3$) δ 7.63 (s, 1H), 7.41 (dd, J = 8.1, 1.9 Hz, 1H), 7.21 (d, J = 8.1 Hz, 1H), 6.21 (s, 1H), 5.12 (q, J = 6.8 Hz, 1H), 2.93 (d, J = 4.8 Hz, 3H), 1.51 (d, J = 6.7 Hz, 3H).

^{13}C NMR (101 MHz, $CDCl_3$) δ 169.0, 142.2, 134.0, 131.0, 130.2, 128.6, 125.0, 57.0, 26.9, 21.7.

IR (neat) cm^{-1} υ: 3249 (w), 3015 (w), 2358 (m), 2329 (m), 2141 (m), 1797 (s), 1610 (s), 1438 (s), 1225 (s), 1101 (s), 797 (s).

HRMS (ESI) m/z: $[M + H]^+$ Calcd for $C_{10}H_{12}BrN_4O^+$ 283.0189; Found 283.0188.

2-(1-azidoethyl)-*N*,3-dimethylbenzamide (4g)

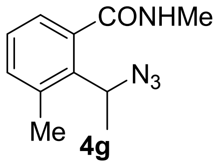

4g

The crude product was purified by flash column chromatography on silica gel to afford **4g** (17.6 mg, 86% yield) as an oil.

^{1}H NMR (400 MHz, $CDCl_3$) δ 7.60 – 7.11 (m, 3H), 6.06 (s, 1H), 5.28 (q, J = 7.0 Hz, 1H), 3.11 (d, J = 4.9 Hz, 3H), 2.60 (s, 3H), 1.74 (d, J = 7.0 Hz, 3H).

^{13}C NMR (101 MHz, $CDCl_3$) δ 171.3, 137.4, 137.2, 136.4, 133.3, 127.7, 125.1, 58.4, 26.9, 20.4, 19.9.

IR (neat) cm^{-1} υ: 3285 (w), 2972 (w), 2902 (w), 2113 (s), 1637 (s), 1538 (m), 1454 (m), 1409 (m), 1308 (m), 1244 (s), 1197 (m), 1076 (m), 1057 (s), 807 (s), 746 (m), 666 (m).

HRMS (ESI) m/z: $[M + Na]^+$ Calcd for $C_{11}H_{14}N_4NaO^+$ 241.1060; Found 241.1063.

2-(1-azidoethyl)-3-fluoro-*N*-methylbenzamide (4h)

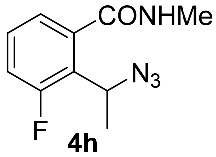

4h

The crude product was purified by flash column chromatography on silica gel to afford **4h** (17.4 mg, 78% yield) as an oil.

^{1}H NMR (400 MHz, $CDCl_3$) δ 7.40 – 7.27 (m, 1H), 7.19 – 7.01 (m, 2H), 6.13 (s, 1H), 5.07 (qd, J = 6.9, 1.4 Hz, 1H), 2.99 (d, J = 4.9 Hz, 3H), 1.68 (dd, J = 7.0, 1.2 Hz, 3H).

^{13}C NMR (101 MHz, $CDCl_3$) δ 169.0 (d, J = 2.9 Hz), 161.6 (d, J = 250.9 Hz), 138.3 (d, J = 4.0 Hz), 129.8 (d, J = 9.2 Hz), 126.4 (d, J = 13.2 Hz), 123.0 (d, J = 3.6 Hz), 118.0 (d, J = 23.3 Hz), 55.1, 26.9, 19.4 (d, ^4J = 3.6 Hz).

IR (neat) cm^{-1} υ: 3282 (w), 2091 (s), 1542 (s), 1447 (w), 1409 (m), 1259 (s), 1215 (w), 1127 (w), 1058 (w), 1008 (w), 853 (w), 762 (m), 705 (s).

HRMS (ESI) m/z: $[M + Na]^+$ Calcd for $C_{10}H_{11}FN_4NaO^+$ 245.0809; Found 245.0813.

2-(1-azidoethyl)-3-bromo-*N*-methylbenzamide (4i)

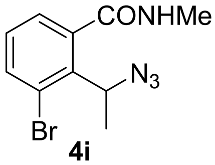

4i

The crude product was purified by flash column chromatography on silica gel to afford **4i** (21.4 mg, 76% yield) as an oil, which was contaminated by the amide **4i'**.

^{1}H NMR (400 MHz, $CDCl_3$) δ 7.55 (dd, J = 8.0, 1.2 Hz, 1H), 7.21 (d, J = 8.0, 1.2 Hz, 1H), 7.07 (t, J = 7.8 Hz, 1H), 5.92 (s, 1H), 5.21 (q, J = 6.9 Hz, 1H), 2.93 (d, J = 4.9 Hz, 3H), 1.63 (d, J = 6.9 Hz, 3H).

^{13}C NMR (101 MHz, $CDCl_3$) δ 169.9, 138.7, 137.5, 134.9, 129.0, 127.2, 123.7, 59.8, 26.9, 19.2.

IR (neat) cm^{-1} υ: 3289 (w), 2970 (w), 2159 (w), 2114 (s), 1629 (s), 1559 (m), 1553 (s), 1470 (m), 1407 (m), 1316 (s), 1255 (s), 1064 (s), 1011 (m), 806 (s), 716 (s).

HRMS (ESI) m/z: $[M + Na]^+$ Calcd for $C_{10}H_{11}BrN_4NaO^+$ 305.0008; Found 305.0010.

3-bromo-2-ethyl-*N*-methylbenzamide (4i')

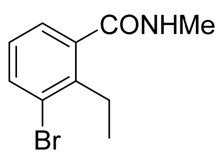

4i'

^{1}H NMR (400 MHz, $CDCl_3$) δ 7.5 (dd, J = 8.0, 1.3 Hz, 1H), 7.14 (dt, J = 7.8, 1.4 Hz, 1H), 6.97 (t, J = 7.8 Hz, 1H), 5.78 (s, 1H), 2.90 (d, J = 7.6 Hz, 3H), 2.79 (q, J = 7.5 Hz, 2H), 1.14 (t, J = 7.5 Hz, 3H).

^{13}C NMR (101 MHz, $CDCl_3$) δ 170.1, 141.2, 138.6, 134.4, 129.9, 125.8, 27.0, 26.7, 14.6.

2-(1-azidoethyl)-*N*,5-dimethylbenzamide (4j)

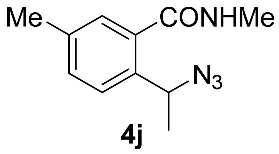

4j

The crude product was purified by flash column chromatography on silica gel to afford **4j** (18.9 mg, 86% yield) as an oil.

^{1}H NMR (400 MHz, $CDCl_3$) δ 7.40 (d, J = 7.9 Hz, 1H), 7.28 (d, J = 7.6 Hz, 1H), 7.21 (s, 1H), 6.15 (s, 1H), 5.11 (q, J = 6.8 Hz, 1H), 2.97 (d, J = 4.8 Hz, 3H), 2.35 (s, 3H), 1.54 (d, J = 6.7 Hz, 3H).

^{13}C NMR (101 MHz, $CDCl_3$) δ 170.0, 137.9, 136.5, 135.5, 131.4, 127.7, 126.7, 57.3, 26.8, 21.5, 21.0.

IR (neat) cm^{-1} υ: 3265 (w), 2978 (w), 2111 (s), 2073 (m), 1625 (s), 1561 (s), 1452 (m), 1409 (m), 1319 (s), 1249 (s), 1146 (m), 1071 (m), 836 (s), 719 (s).

HRMS (ESI) m/z: $[M + H]^+$ Calcd for $C_{11}H_{15}N_4O^+$ 219.1240; Found 219.1236.

2-(1-azidoethyl)-5-methoxy-N-methylbenzamide (4k)

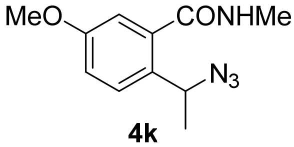

4k

The crude product was purified by flash column chromatography on silica gel to afford **4k** (16.8 mg, 72% yield) as an oil and **4k'** (5.6 mg, 21% yield) as an oil.

^{1}H NMR (400 MHz, $CDCl_3$) δ 7.41 (d, J = 8.7 Hz, 1H), 6.97 (dd, J = 8.7, 2.6 Hz, 1H), 6.90 (d, J = 2.5 Hz, 1H), 6.11 (s, 1H), 5.03 (q, J = 6.8 Hz, 1H), 3.80 (s, 3H), 2.97 (d, J = 5.1 Hz, 3H), 1.52 (d, J = 6.8 Hz, 3H).

^{13}C NMR (101 MHz, $CDCl_3$) δ 169.6, 159.0, 136.9, 131.2, 128.2, 116.0, 112.7, 57.1, 55.6, 26.9, 21.4.

IR (neat) cm^{-1} υ: 3288 (w), 2936 (w), 2099 (m), 1639 (m), 1605 (m), 1541 (m), 1493 (w), 1410 (w), 1293 (m), 1237 (s), 1116 (w), 1036 (m), 826 (m).

HRMS (ESI) m/z: $[M + Na]^+$ Calcd for $C_{11}H_{14}N_4NaO_2^+$ 257.1009; Found 257.1009.

2-(1-(*tert*-butoxy)ethyl)-5-methoxy-*N*-methylbenzamide (4k')

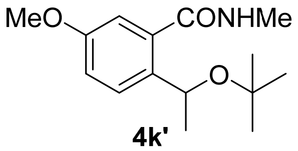

4k'

^{1}H NMR (400 MHz, $CDCl_3$) δ 7.39 (d, J = 8.6 Hz, 1H), 7.19 (s, 1H), 7.05 (s, 1H), 6.90 (dd, J = 8.6, 2.6 Hz, 1H), 4.96 (q, J = 6.6 Hz, 1H), 3.79 (s, 3H), 2.96 (d, J = 4.8 Hz, 3H), 1.35 (d, J = 6.5 Hz, 3H), 1.15 (s, 9H).

^{13}C NMR (101 MHz, $CDCl_3$) δ 170.1, 158.3, 137.0, 135.3, 129.6, 116.0, 113.6, 74.9, 68.7, 55.5, 28.6, 26.7, 25.5.

IR (neat) cm^{-1} υ: 3308 (w), 2974 (w), 1631 (m), 1601 (m), 1556 (m), 1469 (w), 1367 (w), 1327 (w), 1275 (w), 1234 (s), 1195 (w), 1076 (s), 1039 (m), 1015 (m), 958 (w), 850 (m), 703 (m).

HRMS (ESI) m/z: $[M + H]^+$ Calcd for $C_{15}H_{24}NO_3^+$ 266.1751; Found 266.1747.

2-(1-azidoethyl)-5-chloro-*N*-methylbenzamide (4l)

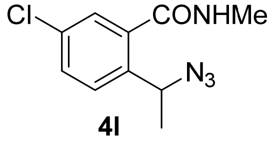

4l

The crude product was purified by flash column chromatography on silica gel to afford **4l** (22.3 mg, 93% yield) as an oil.

^{1}H NMR (400 MHz, $CDCl_3$) δ 7.43 (d, J = 8.4 Hz, 1H), 7.40 (dd, J = 8.4, 2.0 Hz, 1H), 7.33 (d, J = 2.0 Hz, 1H), 6.35 (s, 1H), 5.07 (q, J = 6.7 Hz, 1H), 2.92 (d, J = 4.9 Hz, 3H), 1.49 (d, J = 6.8 Hz, 3H).

^{13}C NMR (101 MHz, $CDCl_3$) δ 168.5, 138.3, 136.8, 133.6, 130.7, 128.4, 127.2, 56.9, 26.8, 21.6.

IR (neat) cm^{-1} υ: 3238 (w), 3088 (w), 2976 (w), 2113 (s), 2079 (s), 1624 (s), 1619 (m), 1559 (s), 1412 (m), 1311 (s), 1242 (s), 1154 (m), 1099 (m), 1064 (m), 1007 (w), 881 (m), 827 (s), 720 (s), 687 (m).

HRMS (ESI) m/z: $[M + H]^+$ Calcd for $C_{10}H_{12}ClN_4O^+$ 239.0772; Found 239.0768.

2-(1-azidoethyl)-5-bromo-*N*-methylbenzamide (4m)

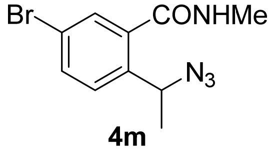

4m

The crude product was purified by flash column chromatography on silica gel to afford **4m** (23.4 mg, 93% yield) as a white solid.

MP: 89 – 90 ºC

^{1}H NMR (400 MHz, $CDCl_3$) δ 7.56 (dd, J = 8.4, 1.9 Hz, 1H), 7.49 (d, J = 1.9 Hz, 1H), 7.37 (d, J = 8.4 Hz, 1H), 6.26 (s, 1H), 5.07 (q, J = 6.8 Hz, 1H), 2.94 (d, J = 4.8 Hz, 3H), 1.50 (d, J = 6.7 Hz, 3H).

^{13}C NMR (101 MHz, $CDCl_3$) δ 168.3, 138.9, 137.1, 133.7, 130.0, 128.7, 121.6, 57.0, 26.9, 21.6.

IR (neat) cm^{-1} υ: 3238 (w), 2981 (w), 2113 (s), 2080 (s), 1625 (s), 1568 (m), 1557 (s), 1409 (m), 1311 (s), 1243 (s), 1153 (m), 1088 (m), 1062 (s), 1005 (m), 861 (m), 824 (s), 718 (s), 680 (s).

HRMS (ESI) m/z: $[M + H]^+$ Calcd for $C_{10}H_{12}BrN_4O^+$ 283.0189; Found 283.0187.

3-(1-azidoethyl)-*N*-methyl-2-naphthamide (4n)

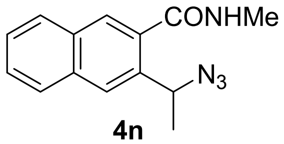

4n

The crude product was purified by flash column chromatography on silica gel to afford **4n** (20.7 mg, 82% yield) as a white solid.

MP: 106-107 °C

^{1}H NMR (400 MHz, $CDCl_3$) δ 7.92 (s, 1H), 7.86 (s, 1H), 7.84 (d, J = 8.0 Hz, 1H), 7.78 (d, J = 8.0 Hz, 1H), 7.58 – 7.45 (m, 2H), 6.32 (s, 1H), 5.32 (q, J = 6.7 Hz, 1H), 3.01 (d, J = 4.9 Hz, 3H), 1.63 (d, J = 6.8 Hz, 3H).

^{13}C NMR (101 MHz, $CDCl_3$) δ 170.1, 136.6, 133.8, 133.6, 132.0, 128.1, 128.0, 127.7, 127.3, 127.1, 126.1, 57.4, 27.0, 21.5.

IR (neat) cm^{-1} υ: 3269 (w), 2972 (w), 2093 (s), 1627 (s), 1552 (m), 1457 (w), 1409 (m), 1313 (m), 1245 (m), 1161 (w), 1109 (w), 1053 (w), 1012 (w), 891 (m), 845 (w), 746 (s), 721 (s).

HRMS (ESI) m/z: $[M + Na]^+$ Calcd for $C_{14}H_{14}N_4NaO^+$ 277.1060; Found 277.1067.

1-(1-azidoethyl)-*N*-methyl-2-naphthamide (4o)

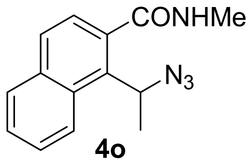

4o

The crude product was purified by flash column chromatography on silica gel to afford **4o** (21.5 mg, 84% yield) as an oil.

^{1}H NMR (400 MHz, $CDCl_3$) δ 8.40 (d, J = 8.5 Hz, 1H), 7.75 (dd, J = 8.1, 1.6 Hz, 1H), 7.65 (d, J = 8.1 Hz, 1H), 7.52 – 7.41 (m, 2H), 7.23 (d, J = 8.4 Hz, 1H), 6.05 (s, 1H), 5.48 (q, J = 7.0 Hz, 1H), 2.92 (d, J = 4.9 Hz, 3H), 1.66 (d, J = 7.0 Hz, 3H).

^{13}C NMR (101 MHz, $CDCl_3$) δ 170.9, 134.6, 134.4, 134.2, 130.3, 129.2, 129.1, 126.8, 126.7, 125.9, 123.4, 58.9, 26.9, 21.4.

IR (neat) cm^{-1} υ: 3259 (w), 2987 (w), 2110 (m), 2069 (w), 1609 (m), 1554 (w), 1492 (w), 1409 (m), 1284 (s), 1230 (s), 1066 (m), 1003 (w), 824 (m), 692 (w).

HRMS (ESI) m/z: $[M + Na]^+$ Calcd for $C_{14}H_{14}N_4NaO^+$ 277.1060; Found 277.1058.

2-(1-azidoethyl)-*N*-ethylbenzamide (4p)

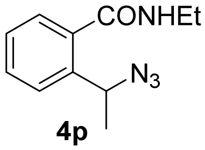

4p

The crude product was purified by flash column chromatography on silica gel to afford **4p** (16.5 mg, 75% yield) as an oil.

^{1}H NMR (400 MHz, $CDCl_3$) δ 7.46 (d, J = 7.8 Hz, 1H), 7.43 – 7.38 (m, 1H), 7.33 (d, J = 7.5 Hz, 1H), 7.26 (t, J = 7.4 Hz, 1H), 5.88 (s, 1H), 5.10 (q, J = 6.7 Hz, 1H), 3.46-3.38 (m, 2H), 1.50 (d, J = 6.7 Hz, 3H), 1.19 (t, J = 7.3 Hz, 3H).

^{13}C NMR (101 MHz, $CDCl_3$) δ 169.2, 139.6, 135.8, 130.7, 128.0, 127.0, 126.9, 57.5, 35.1, 21.6, 15.0.

IR (neat) cm^{-1} υ: 3281 (w), 2976 (w), 2090 (s), 1637 (s), 1535 (s), 1448 (m), 1305 (s), 1251 (s), 1149 (w), 1066 (m), 872 (w), 760 (s), 650 (m).

HRMS (ESI) m/z: $[M + Na]^+$ Calcd for $C_{11}H_{14}N_4NaO^+$ 241.1060; Found 241.1062.

2-(2-azidopropan-2-yl)-*N*-methylbenzamide (4q)

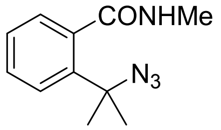

4q

The crude product was purified by flash column chromatography on silica gel to afford **4q** (16.1 mg, 74% yield) as an oil.

^{1}H NMR (400 MHz, $CDCl_3$) δ 7.46 – 7.43 (m, 1H), 7.39 (ddd, J = 8.1, 5.9, 2.9 Hz, 1H), 7.34 – 7.26 (m, 2H), 5.87 (s, 1H), 3.03 (d, J = 4.9 Hz, 3H), 1.78 (s, 6H).

^{13}C NMR (101 MHz, $CDCl_3$) δ 172.4, 142.1, 136.4, 129.6, 128.6, 127.6, 126.3, 64.5, 28.6, 27.0.

IR (neat) cm^{-1} υ: 3275 (w), 2934 (w), 2111 (s), 1628 (s), 1549 (m), 1407 (m), 1312 (m), 1249 (s), 1149 (m), 1075 (w), 766 (s), 720 (s).

HRMS (ESI) m/z: $[M + Na]^+$ Calcd for $C_{11}H_{14}N_4NaO^+$ 241.1060; Found 241.1063.

2-(azidomethyl)-*N*-methylbenzamide (4r)

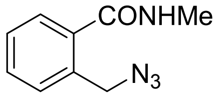

4r

The crude product was purified by flash column chromatography on silica gel to afford **4r** (15.9 mg, 83% yield) as an oil.

^{1}H NMR (400 MHz, $CDCl_3$) δ 7.49 (d, J = 7.6 Hz, 1H), 7.46 – 7.42 (m, 1H), 7.41 – 7.33 (m, 2H), 6.18 (s, 1H), 4.57 (s, 2H), 2.99 (d, J = 4.9 Hz, 3H).

^{13}C NMR (101 MHz, $CDCl_3$) δ 169.5, 136.0, 134.0, 130.7, 130.2, 128.6, 127.8, 52.8, 26.9.

IR (neat) cm^{-1} υ: 3284 (w), 2939 (w), 2074 (m), 1628 (m), 1551 (m), 1447 (w), 1407 (w), 1319 (m), 1232 (m), 1171 (m), 878 (w), 850 (w), 755 (m), 698 (s).

HRMS (ESI) m/z: $[M + Na]^+$ Calcd for $C_9H_{10}N_4NaO^+$ 213.0747; Found 213.0748.

2-(1-azidopropyl)-*N*-methylbenzamide (4s)

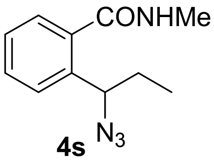

4s

The crude product was purified by flash column chromatography on silica gel to afford **4s** (18.0 mg, 82% yield) as an oil.

^{1}H NMR (400 MHz, $CDCl_3$) δ 7.49 – 7.41 (m, 2H), 7.39 (d, J = 7.0 Hz, 1H), 7.31 (ddd, J = 7.7, 6.2, 2.3 Hz, 1H), 6.05 (s, 1H), 4.92 (dd, J = 7.7, 6.3 Hz, 1H), 2.97 (d, J = 4.9 Hz, 3H), 1.93 – 1.78 (m, 2H), 0.96 (t, J = 7.3 Hz, 3H).

^{13}C NMR (101 MHz, $CDCl_3$) δ 169.9, 138.6, 136.0, 130.6, 128.0, 127.3, 127.1, 63.7, 29.3, 26.9, 11.0.

IR (neat) cm^{-1} υ: 3242 (w), 2966 (w), 2158 (w), 2089 (s), 1645 (m), 1624 (s), 1558 (m), 1447 (w), 1322 (m), 1242 (s), 1174 (w), 911 (w), 754 (m), 720 (s).

HRMS (ESI) m/z: $[M + Na]^+$ Calcd for $C_{11}H_{14}N_4NaO^+$ 241.1060; Found 241.1064.

2-(1-azido-3-methoxypropyl)-*N*-methylbenzamide (4t)

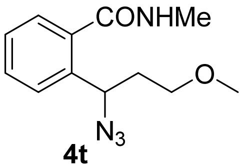

4t

The crude product was purified by flash column chromatography on silica gel to afford **4t** (20.2 mg, 81% yield) as an oil.

^{1}H NMR (400 MHz, $CDCl_3$) δ 7.47 – 7.43 (m, 2H), 7.40 (d, J = 7.7 Hz, 1H), 7.33-7.27 (m, 1H), 6.33 (s, 1H), 5.13 (t, J = 7.3 Hz, 1H), 3.47 (dt, J = 10.4, 6.5 Hz, 1H), 3.40 – 3.30 (m, 1H), 3.28 (s, 3H), 2.94 (d, J = 5.2 Hz, 3H), 2.05 (q, J = 6.6 Hz, 2H).

^{13}C **NMR** (101 MHz, $CDCl_3$) δ 169.7, 137.8, 136.1, 130.6, 128.2, 127.5, 127.3, 69.2, 59.3, 58.6, 36.0. 26.8.

IR (neat) cm^{-1} υ: 3304 (w), 2933 (w), 2878 (w), 2095 (s), 1637 (s), 1539 (m), 1410 (w), 1307 (m), 1245 (m), 1118 (s), 1012 (w), 761 (m), 670 (s).

HRMS (ESI) m/z: $[M + H]^+$ Calcd for $C_{12}H_{17}N_4O_2^+$ 249.1346; Found 249.1341.

2-(1-azido-3-(benzyloxy)propyl)-*N*-methylbenzamide (4u)

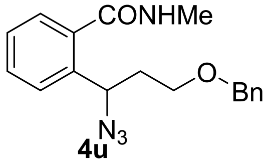

4u

The crude product was purified by flash column chromatography on silica gel to afford **4u** (30.2 mg, 93% yield) as an oil.

^{1}H **NMR** (400 MHz, $CDCl_3$) δ 7.50 – 7.43 (m, 2H), 7.44 – 7.38 (m, 1H), 7.36 – 7.27 (m, 6H), 6.20 (s, 1H), 5.18 (t, J = 7.6 Hz, 1H), 4.45 (s, 2H), 3.60 (dt, J = 9.8, 6.3 Hz, 1H), 3.49 (dt, J = 9.7, 6.1 Hz, 1H), 2.83 (d, J = 4.8 Hz, 3H), 2.17 – 2.11 (m, 2H).

^{13}C **NMR** (101 MHz, $CDCl_3$) δ 169.6, 138.1, 137.6, 136.4, 130.6, 128.5, 128.2, 127.9, 127.8, 127.6, 127.3, 73.1, 67.0, 59.4, 35.9, 26.7.

IR (neat) cm^{-1} υ: 3291 (w), 2868 (w), 2097 (s), 1717 (w), 1637 (s), 1542 (m), 1409 (w), 1309 (m), 1274 (m), 1101 (m), 1099 (m), 757 (s), 716 (s), 699 (s).

HRMS (ESI) m/z: $[M + H]^+$ Calcd for $C_{18}H_{21}N_4O_2^+$ 325.1659; Found 325.1652.

General procedure for the Copper-catalyzed oxidative δ-trifluoromethylation of 2-alkyl benzohydrazides 3

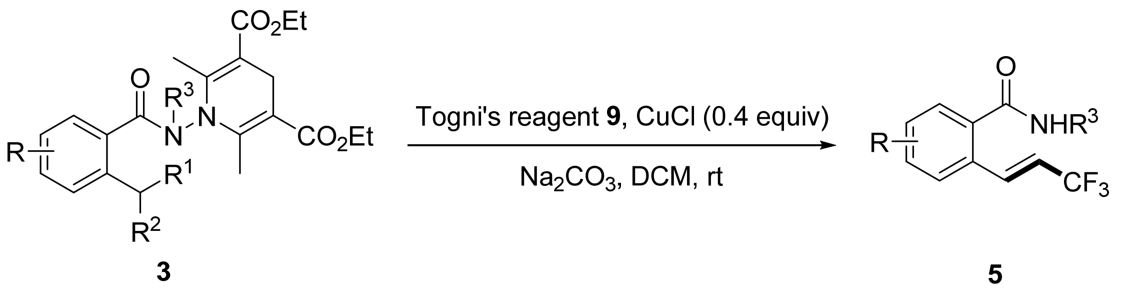

To a screw cap tube charged with CuCl (4.0 mg, 0.04 mmol) and Na_2CO_3 (31.8 mg, 0.3 mmol, 3.0 equiv) was added a solution of Togni's reagent **9** (94.8 mg, 0.3 mmol, 3.0 equiv) in DCM (0.5 mL) under argon atmosphere. After stirring for 2 min, substrate **3** (0.1 mmol, 1.0 equiv) in DCM (0.5 mL) was added to the above mixture. After being stirred for 4 hours at room temperature under argon atmosphere, the reaction mixture was quenched with water, extracted with EtOAc. The organic extracts were washed with Na_2CO_3 solution and brine, dried over Na_2SO_4. The solvent was removed under reduced pressure. The residue was purified by flash chromatography (SiO_2, eluent: PE/EtOAc = 2/1) to give **5**.

Characterization of compounds 5

(*E*)-*N*-methyl-2-(3,3,3-trifluoroprop-1-en-1-yl)benzamide (5a)

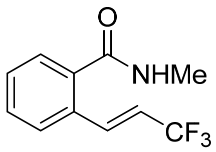

5a

The crude product was purified by flash column chromatography on silica gel to afford **5a** (16.8 mg, 74% yield) as a white solid.

MP: 104 – 105 °C

^{1}H NMR (400 MHz, $CDCl_3$) δ 7.61 – 7.54 (m, 2H), 7.49 – 7.43 (m, 2H), 7.42 – 7.37 (m, 1H), 6.16 (dq, J = 16.1, 6.5 Hz, 1H), 5.88 (s, 1H), 3.00 (d, J = 4.9 Hz, 3H).

^{13}C NMR (101 MHz, $CDCl_3$) δ 169.3, 136.7, 135.6 (q, J = 6.9 Hz), 132.2, 130.6, 129.8, 127.6, 127.2, 123.4 (q, J = 269.2 Hz), 118.2 (q, J = 33.8 Hz), 27.0.

^{19}F NMR (376 MHz, $CDCl_3$) δ –63.46 (dd, J = 6.5, 2.3 Hz).

IR (neat) cm^{-1} υ: 3285 (w), 1668 (w), 1633 (m), 1546 (m), 1417 (w), 1319 (m), 1272 (s), 1101 (s), 966 (m), 858 (w), 748 (m), 696 (m).

HRMS (ESI) m/z: $[M + H]^+$ Calcd for $C_{11}H_{11}F_3NO^+$ 230.0787; Found 230.0788.

(*E*)-*N*,4-dimethyl-2-(3,3,3-trifluoroprop-1-en-1-yl)benzamide (5b)

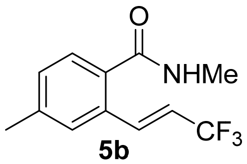

5b

The crude product was purified by flash column chromatography on silica gel to afford **5b** (18.1 mg, 74% yield) as a white solid.

MP: 143 – 144 °C

^{1}H NMR (400 MHz, $CDCl_3$) δ 7.59 (dq, J = 16.0, 2.3 Hz, 1H), 7.38 – 7.33 (m, 2H), 7.18 (d, J = 7.8 Hz, 1H), 6.13 (dq, J = 16.1, 6.5 Hz, 1H), 5.92 (s, 1H), 2.98 (d, J = 4.6 Hz, 3H), 2.38 (s, 3H).

^{13}C NMR (101 MHz, $CDCl_3$) δ 169.4, 140.8, 135.9 (q, J = 6.8 Hz), 133.8, 132.2, 130.5, 127.8, 127.6, 123.4 (q, J = 269.2 Hz), 117.9 (q, J = 33.8 Hz), 27.0, 21.4.

^{19}F NMR (376 MHz, $CDCl_3$) δ –63.39 (dd, J = 6.5, 2.4 Hz).

IR (neat) cm^{-1} υ: 3281 (w), 1665 (w), 1633 (m), 1557 (w), 1408 (w), 1315 (m), 1271 (m), 1127 (m), 1099 (s), 969 (w), 833 (m), 741 (w), 703 (m).

HRMS (ESI) m/z: $[M + H]^+$ Calcd for $C_{12}H_{13}F_3NO^+$ 244.0944; Found 244.0944.

(*E*)-*N*-methyl-3-(3,3,3-trifluoroprop-1-en-1-yl)-[1,1'-biphenyl]-4-carboxamide (5c)

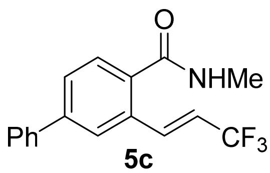

5c

The crude product was purified by flash column chromatography on silica gel to afford **5c** (23.2 mg, 76% yield) as a white solid.

MP: 193 – 194 °C

^{1}H NMR (400 MHz, $CDCl_3$) δ 7.73 (d, J = 1.8 Hz, 1H), 7.67 (dq, J = 16.2, 2.3 Hz, 1H), 7.63 – 7.52 (m, 4H), 7.51 – 7.37 (m, 3H), 6.22 (dq, J = 16.1, 6.4 Hz, 1H), 5.96 (s, 1H), 3.03 (d, J = 4.9 Hz, 3H).

^{13}C NMR (101 MHz, $CDCl_3$) δ 169.1, 143.7, 139.6, 135.8 (q, J = 6.9 Hz), 135.2, 132.8, 129.2, 128.4, 128.2, 127.3, 125.9, 123.4 (q, J = 269.4 Hz), 118.5 (q, J = 34.0 Hz), 27.1.

^{19}F NMR (376 MHz, $CDCl_3$) δ –63.41 (dd, J = 6.4, 2.3 Hz).

IR (neat) cm^{-1} υ: 3291 (w), 2158 (w), 1667 (w), 1629 (m), 1545 (w), 1410 (w), 1303 (m), 1269 (m), 1113 (s), 970 (w), 839 (w), 762 (s), 699 (s).

HRMS (ESI) m/z: $[M + Na]^+$ Calcd for $C_{17}H_{14}F_3NNaO^+$ 328.0920; Found 328.0917.

(*E*)-4-methoxy-*N*-methyl-2-(3,3,3-trifluoroprop-1-en-1-yl)benzamide (5d)

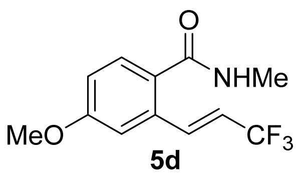

5d

The crude product was purified by flash column chromatography on silica gel to afford **5d** (15.9 mg, 61% yield) as a white solid.

MP: 151 – 152 °C

^{1}H NMR (400 MHz, $CDCl_3$) δ 7.65 (dq, J = 16.0, 2.2 Hz, 1H), 7.43 (d, J = 8.5 Hz, 1H), 7.02 (d, J = 2.5 Hz, 1H), 6.90 (dd, J = 8.5, 2.6 Hz, 1H), 6.12 (dq, J = 16.1, 6.4 Hz, 1H), 5.83 (s, 1H), 3.86 (s, 3H), 2.98 (d, J = 4.9 Hz, 3H).

^{13}C NMR (101 MHz, $CDCl_3$) δ 169.0, 161.1, 136.1 (q, J = 6.8 Hz), 134.4, 129.4, 129.0, 123.4 (q, J = 269.2 Hz), 118.3 (q, J = 34.0 Hz), 115.0, 112.5, 55.6, 27.0.

^{19}F NMR (376 MHz, $CDCl_3$) δ -63.42 (dd, J = 6.4, 2.3 Hz).

IR (neat) cm^{-1} υ: 3234 (m), 1649 (s), 1592 (s), 1541 (s), 1418 (s), 1307 (s), 1307 (s), 1209 (s), 1096 (s), 1018 (m), 953 (m), 792 (w), 760 (w).

HRMS (ESI) m/z: $[M + H]^+$ Calcd for $C_{12}H_{13}F_3NO_2^+$ 260.0893; Found 260.0896.

(*E*)-4-fluoro-*N*-methyl-2-(3,3,3-trifluoroprop-1-en-1-yl)benzamide (5e)

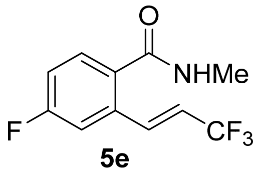

5e

The crude product was purified by flash column chromatography on silica gel to afford **5e** (18.2 mg, 74% yield) as a white solid.

MP: 148 – 149 °C

^{1}H NMR (400 MHz, $CDCl_3$) δ 7.50 (d, J = 16.0 Hz, 1H), 7.42 – 7.37 (m, 1H), 7.20 – 7.16 (m, 1H), 7.01 (t, J = 8.1 Hz, 1H), 6.13 – 6.02 (m, 1H), 5.85 (s, 1H), 2.92 (d, J = 4.8 Hz, 3H).

^{13}C NMR (101 MHz, $CDCl_3$) δ 168.4, 163.6 (d, J = 251.1 Hz), 134.9 (d, J = 8.2 Hz), 134.7 (m), 132.8 (d, J = 3.3 Hz), 129.8 (d, J = 8.6 Hz), 123.1 (q, J = 269.6 Hz), 119.4 (q, J = 34.3 Hz), 116.8 (d, J = 21.7 Hz), 114.0 (d, J = 22.7 Hz), 27.1.

^{19}F NMR (376 MHz, $CDCl_3$) δ -63.68 (d, J = 8.6 Hz), -109.0 – -109.1 (m).

IR (neat) cm^{-1} υ: 3294 (w), 1669 (w), 1631 (m), 1585 (w), 1552 (m), 1492 (w), 1419 (w), 1323 (m), 1271 (s), 1222 (w), 1170 (w), 1112 (s), 972 (m), 843 (w), 725 (w), 687 (m).

HRMS (APCI) m/z: $[M + H]^+$ Calcd for $C_{11}H_{10}F_4NO^+$ 248.0693; Found 248.0695.

(E)-4-chloro-*N*-methyl-2-(3,3,3-trifluoroprop-1-en-1-yl)benzamide (5f)

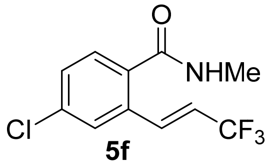

5f

The crude product was purified by flash column chromatography on silica gel to afford **5f** (17.2 mg, 65% yield) as a white solid.

MP: 140 – 142 °C

^{1}H NMR (400 MHz, $CDCl_3$) δ 7.53 (dq, J = 16.0, 2.2 Hz, 2H), 7.53 (d, J = 2.0 Hz, 1H), 7.40 (d, J = 8.2 Hz, 1H), 7.35 (dd, J = 8.2, 2.0 Hz, 1H), 6.16 (dq, J = 16.1, 6.3 Hz, 1H), 5.94 (s, 1H), 2.99 (d, J = 4.8 Hz, 3H).

^{13}C NMR (101 MHz, $CDCl_3$) δ 168.3, 136.8, 134.8, 134.5 (q, J = 6.9 Hz), 134.1, 129.7, 129.0, 127.2, 123.1 (q, J = 269.6 Hz), 119.4 (q, J = 34.3 Hz), 27.1.

^{19}F NMR (376 MHz, $CDCl_3$) δ -63.69 (dd, J = 6.3, 2.4 Hz).

IR (neat) cm^{-1} υ: 3292 (w), 1665 (w), 1633 (m), 1572 (w), 1563 (m), 1484 (w), 1414 (w), 1314 (s), 1286 (m), 1255 (m), 1103 (s), 971 (m), 917 (m), 836 (m), 693 (s).

HRMS (APCI/QTOF) m/z: $[M + H]^+$ Calcd for $C_{11}H_{10}ClF_3NO^+$ 264.0398; Found 264.0403.

(*E*)-4-bromo-*N*-methyl-2-(3,3,3-trifluoroprop-1-en-1-yl)benzamide (5g)

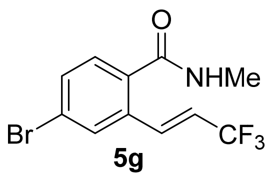

5g

The crude product was purified by flash column chromatography on silica gel to afford **5g** (19.4 mg, 65% yield) as a white solid.

MP: 142 – 144 °C

^{1}H NMR (400 MHz, $CDCl_3$) δ 7.60 (s, 1H), 7.46 – 7.39 (m, 2H), 7.24 (d, J = 8.1 Hz, 1H), 6.12 – 6.02 (m, 1H), 5.75 (s, 1H), 2.90 (d, J = 4.9 Hz, 3H).

^{13}C NMR (101 MHz, $CDCl_3$) δ 168.3, 135.2, 134.4 (q, J = 7.0 Hz), 134.2, 132.7, 130.2, 129.1, 124.9, 123.0 (q, J = 269.5 Hz), 119.5 (q, J = 34.2 Hz), 27.1.

^{19}F NMR (376 MHz, $CDCl_3$) δ –63.69 (d, J = 6.4 Hz).

IR (neat) cm^{-1} υ: 3283 (w), 2161 (w), 1666 (w), 1630 (m), 1562 (m), 1415 (w), 1315 (s), 1258 (m), 1108 (s), 967 (w), 901 (w), 829 (w), 723 (w), 699 (m).

HRMS (ESI/QTOF) m/z: $[M + H]^+$ Calcd for $C_{11}H_{10}BrF_3NO^+$ 307.9892; Found 307.9892.

(*E*)-4-cyano-*N*-methyl-2-(3,3,3-trifluoroprop-1-en-1-yl)benzamide (5h)

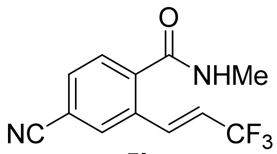

5h

The crude product was purified by flash column chromatography on silica gel to afford **5h** (17.4 mg, 68% yield) as a white solid.

MP: 136 – 138°C

^{1}**H NMR** (400 MHz, $CDCl_3$) δ 7.84 (d, J = 1.4 Hz, 1H), 7.68 (dd, J = 8.0, 1.5 Hz, 1H), 7.58 (d, J = 7.9 Hz, 1H), 7.51 (dq, J = 16.2, 2.3 Hz, 1H), 6.23 (dq, J = 16.1, 6.2 Hz, 1H), 5.95 (s, 1H), 3.02 (d, J = 4.9 Hz, 3H).

^{13}**C NMR** (101 MHz, $CDCl_3$) δ 167.4, 140.2, 133.62 (q, J = 3.0 Hz), 133.56, 132.9, 130.9, 128.5, 122.8 (q, J = 269.8 Hz), 120.7 (q, J = 34.5 Hz), 117.5, 114.8, 27.1.

^{19}**F NMR** (376 MHz, $CDCl_3$) δ –63.92 (dd, J = 6.1, 2.4 Hz).

IR (neat) cm^{-1} υ: 3282 (w), 2236 (w), 1641 (m), 1570 (w), 1315 (m), 1269 (m), 1140 (s), 1112 (s), 980 (w), 839 (w), 680 (m).

HRMS (APCI) m/z: $[M + H]^+$ Calcd for $C_{12}H_{10}F_3N_2O^+$ 255.0740; Found 255.0734.

(*E*)-*N*,3-dimethyl-2-(3,3,3-trifluoroprop-1-en-1-yl)benzamide (5i)

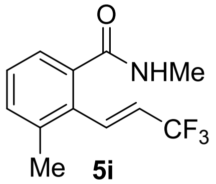

5i

The crude product was purified by flash column chromatography on silica gel to afford **5i** (16.4 mg, 68% yield) as a white solid.

MP: 103 – 104 °C

^{1}**H NMR** (400 MHz, $CDCl_3$) δ 7.28 (dq, J = 16.4, 2.4 Hz, 1H), 7.23 – 7.19 (m, 3H), 5.89 (dq, J = 16.3, 6.3 Hz, 1H), 5.61 (s, 1H), 2.87 (d, J = 4.9 Hz, 3H), 2.28 (s, 3H).

^{13}**C NMR** (101 MHz, $CDCl_3$) δ 170.3, 137.3, 137.0, 134.7 (q, J = 7.0 Hz), 132.0, 131.0, 128.8, 125.4, 123.0 (q, J = 269.4 Hz), 121.8 (q, J = 33.6 Hz). 26.9, 20.6.

^{19}**F NMR** (376 MHz, $CDCl_3$) δ –64.28 (dd, J = 6.6, 2.5 Hz).

IR (neat) cm^{-1} υ: 3302 (w), 1670 (w), 1637 (m), 1545 (m), 1312 (s), 1272 (m), 1108 (s), 969 (m), 877 (w), 785 (w), 760 (w), 722 (w).

HRMS (ESI) m/z: $[M + H]^+$ Calcd for $C_{12}H_{13}F_3NO^+$ 244.0944; Found 244.0944.

(*E*)-3-methoxy-*N*-methyl-2-(3,3,3-trifluoroprop-1-en-1-yl)benzamide (5j)

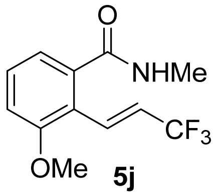

5j

The crude product was purified by flash column chromatography on silica gel to afford **5j** (16.3 mg, 74% yield) as a white solid.

MP: 107 – 108 °C.

^{1}H NMR (400 MHz, $CDCl_3$) δ 7.26 – 7.20 (m, 1H), 7.21 (dq, J = 16.2, 2.3 Hz, 1H), 6.92-6.88 (m, 2H), 6.43 (dq, J = 16.2, 6.6 Hz, 1H), 6.10 (s, 1H), 3.84 (s, 3H), 2.86 (d, J = 4.9 Hz, 3H).

^{13}C NMR (101 MHz, $CDCl_3$) δ 169.9, 158.6, 139.2, 130.8 (q, J = 7.4 Hz), 130.4, 123.7 (q, J = 269.4 Hz), 120.6 (q, J = 33.0 Hz), 119.64, 119.62, 112.3, 55.8, 26.8.

^{19}F NMR (376 MHz, $CDCl_3$) δ –63.91 (dd, J = 6.6, 2.4 Hz).

IR (neat) cm^{-1} υ: 3287 (w), 2361 (w), 1633 (m), 1542 (w), 1469 (w), 1313 (s), 1259 (s), 1112 (s), 1074 (s), 1014 (w), 972 (m), 910 (w), 841 (w), 783 (w), 725 (s).

HRMS (ESI) m/z: $[M + H]^+$ Calcd for $C_{12}H_{13}F_3NO_2^+$ 260.0893; Found 260.0900.

(*E*)-3-fluoro-*N*-methyl-2-(3,3,3-trifluoroprop-1-en-1-yl)benzamide (5k)

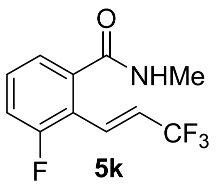

The crude product was purified by flash column chromatography on silica gel to afford **5k** (16.8 mg, 68% yield) as a white solid.

MP: 138 – 139 °C.

^{1}H NMR (400 MHz, $CDCl_3$) δ 7.34 (td, J = 8.0, 5.1 Hz, 1H), 7.26 (dq, J = 16.5, 2.3 Hz, 1H), 7.23-7.21 (m, 1H), 7.20-7.15(m, 1H), 6.42 (dq, J = 16.5, 6.4 Hz, 1H), 5.96 (s, 1H), 2.98 (d, J = 4.9 Hz, 3H).

^{13}C NMR (101 MHz, $CDCl_3$) δ 168.4 (d, J = 3.2 Hz), 161.4 (d, J = 254.5 Hz), 139.2, 130.9 (d, J = 9.6 Hz), 128.8 (q, J = 7.3 Hz), 123.2 (q, J = 269.6 Hz), 123.4 (d, J = 3.6 Hz), 122.6 (qd, J = 33.8, 13.5 Hz), 119.7 (d, J = 12.5 Hz), 27.0.

^{19}F NMR (376 MHz, $CDCl_3$) δ –64.41 (dd, J = 6.3, 2.4 Hz), –110.67 (dd, J = 11.2, 5.2 Hz).

IR (neat) cm^{-1} υ: 3286 (w), 2162 (w), 1667 (w), 1634 (m), 1604 (m), 1543 (m), 1460 (w), 1419 (w), 1320 (s), 1276 (s), 1247 (m), 1205 (m), 1109 (s), 1049 (w), 972 (m), 938 (w), 847 (m), 786 (m), 730 (m), 683 (m).

HRMS (ESI) m/z: $[M + Na]^+$ Calcd for $C_{11}H_9F_4NNaO^+$ 270.0512; Found 270.0516.

(*E*)-3-chloro-*N*-methyl-2-(3,3,3-trifluoroprop-1-en-1-yl)benzamide (5l)

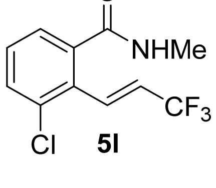

The crude product was purified by flash column chromatography on silica gel to afford **5l** (19.5 mg, 74% yield) as a white solid.

MP: 101 – 102 °C

^{1}H NMR (400 MHz, $CDCl_3$) δ 7.47 (dd, J = 7.4, 2.0 Hz, 1H), 7.36 (dq, J =16.3, 2.3 Hz, 1H), 7.33 – 7.28 (m, 2H), 6.15 (dq, J = 16.3, 6.3 Hz, 1H), 5.78 (s, 1H), 2.93 (d, J = 4.9 Hz, 3H).

^{13}C NMR (101 MHz, $CDCl_3$) δ 168.9, 138.6, 134.6, 132.5 (q, J = 7.2 Hz), 131.3, 130.1, 126.3, 123.0 (q, J = 33.8 Hz), 122.8 (q, J = 270.8 Hz), 27.0.

^{19}F NMR (376 MHz, $CDCl_3$) δ –64.62 (dd, J = 6.3, 2.3 Hz).

IR (neat) cm^{-1} υ: 3268 (w), 2361 (w), 1635 (m), 1542 (m), 1470 (m), 1311 (s), 1258 (s), 1111 (s), 1081 (s), 970 (m), 789 (w), 722 (s).

HRMS (ESI) m/z: $[M + Na]^+$ Calcd for $C_{11}H_9ClF_3NNaO^+$ 286.0217; Found 286.0215.

(*E*)-3-bromo-*N*-methyl-2-(3,3,3-trifluoroprop-1-en-1-yl)benzamide (5m)

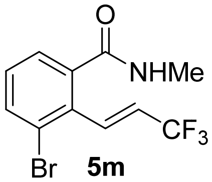

5m

The crude product was purified by flash column chromatography on silica gel to afford **5m** (21.7 mg, 71% yield) as a white solid.

MP: 96 – 97 °C

^{1}H NMR (400 MHz, $CDCl_3$) δ 7.67 (dd, J = 8.0, 1.0 Hz, 1H), 7.40 (dd, J = 7.6, 1.2 Hz, 1H), 7.33 (dq, J = 16.3, 2.3 Hz, 1H), 7.26-7.22 (m, 1H), 6.10 (dq, J = 16.3, 6.3 Hz, 1H), 5.66 (s, 1H), 2.94 (d, J = 4.8 Hz, 3H).

^{13}C NMR (101 MHz, $CDCl_3$) δ 168.9, 138.6, 134.8 (q, J = 7.2 Hz), 134.5, 132.0, 130.3, 127.0, 124.5, 122.9 (q, J = 33.9 Hz), 122.7 (q, J = 268.6 Hz), 27.0.

^{19}F NMR (376 MHz, $CDCl_3$) δ –64.65 (dd, J = 6.3, 2.5 Hz).

IR (neat) cm^{-1} υ: 3287 (w), 1645 (m), 1547 (m), 1430 (w), 1411 (w), 1312 (s), 1269 (m), 1138 (m), 1115 (s), 969 (m), 889 (w), 800 (w), 767 (w), 685 (m).

HRMS (ESI) m/z: $[M + H]^+$ Calcd for $C_{11}H_{10}BrF_3NO^+$ 307.9892; Found 307.9890.

(*E*)-*N*,5-dimethyl-2-(3,3,3-trifluoroprop-1-en-1-yl)benzamide (5n)

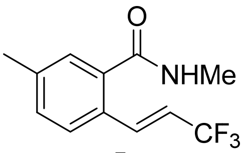

5n

The crude product was purified by flash column chromatography on silica gel to afford **5n** (17.6 mg, 72% yield) as a white solid.

MP: 138-139 °C

^{1}H NMR (400 MHz, $CDCl_3$) δ 7.53 (dq, J = 16.2, 2.3 Hz, 1H), 7.47 (d, J = 7.9 Hz, 1H), 7.35 – 7.14 (m, 2H), 6.13 (dq, J = 16.1, 6.5 Hz, 1H), 5.78 (s, 1H), 3.01 (d, J = 4.7 Hz, 3H), 2.38 (s, 3H).

^{13}C NMR (101 MHz, $CDCl_3$) δ 169.5, 140.2, 136.7, 135.4 (q, J = 7.0 Hz), 131.3, 129.2, 128.2, 127.1, 123.5 (q, J = 269.2 Hz), 117.3 (q, J = 33.7 Hz), 27.0, 21.4.

^{19}F NMR (376 MHz, $CDCl_3$) δ –63.29 (dd, J = 6.5, 2.3 Hz).

IR (neat) cm^{-1} υ: 3296 (w), 2361 (s), 1635 (m), 1544 (w), 1409 (w), 1323 (w), 1272 (m), 1116 (s), 1057 (w), 723 (w).

HRMS (ESI) m/z: $[M + Na]^+$ Calcd for $C_{12}H_{12}F_3NNaO^+$ 266.0763; Found 266.0763.

(*E*)-5-methoxy-*N*-methyl-2-(3,3,3-trifluoroprop-1-en-1-yl)benzamide (5o)

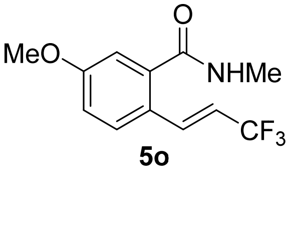

5o

The crude product was purified by flash column chromatography on silica gel to afford **5o** (17.4 mg, 67% yield) as a white solid.

MP: 123 – 124 °C

^{1}H NMR (400 MHz, $CDCl_3$) δ 7.46 (d, J = 8.6 Hz, 1H), 7.39 (dq, J = 16.1, 2.2 Hz, 1H), 6.96 – 6.87 (m, 2H), 6.17 – 6.08 (m, 1H), 6.01 (dq, J = 16.1, 6.5 Hz, 1H), 3.80 (s, 3H), 2.94 (d, J = 4.9 Hz, 3H).

^{13}C NMR (101 MHz, $CDCl_3$) δ 169.1, 160.4, 138.1, 134.7 (q, J = 6.9 Hz), 128.3, 126.2 (d, J = 269.0 Hz), 124.0, 116.1, 115.7 (d, J = 33.7 Hz), 112.6, 55.5, 26.8.

^{19}F NMR (376 MHz, $CDCl_3$) δ –63.04 (dd, J = 6.6, 2.2 Hz).

HRMS (ESI) m/z: $[M + H]^+$ Calcd for $C_{12}H_{13}F_3NO_2^+$ 260.0893; Found 260.0894.

(*E*)-5-chloro-*N*-methyl-2-(3,3,3-trifluoroprop-1-en-1-yl)benzamide (5p)

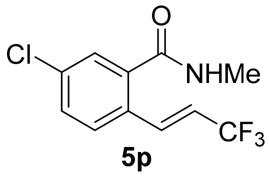

5p

The crude product was purified by flash column chromatography on silica gel to afford **5p** (16.5 mg, 62% yield) as a white solid.

MP: 144 – 145 °C

^{1}H NMR (400 MHz, $CDCl_3$) δ 7.54 – 7.46 (m, 2H), 7.45 – 7.38 (m, 2H), 6.14 (dq, J = 16.1, 6.4 Hz, 1H), 5.96 (s, 1H), 3.00 (d, J = 4.9 Hz, 3H).

^{13}C NMR (101 MHz, $CDCl_3$) δ 167.8, 137.9, 135.8, 134.4 (q, J = 6.9 Hz), 130.7, 130.6, 128.5, 127.8, 123.2 (q, J = 269.4 Hz), 118.7 (q, J = 34.0 Hz), 27.1.

^{19}F NMR (376 MHz, $CDCl_3$) δ – 63.57 (dd, J = 6.3, 2.3 Hz).

IR (neat) cm⁻¹ υ: 3300 (w), 1704 (w), 1669 (w), 1639 (m), 1555 (w), 1318 (m), 1286 (m), 1131 (m), 1105 (s), 971 (m), 877 (m), 820 (m), 721 (m), 695 (m).

HRMS (APCI) m/z: $[M + H]^+$ Calcd for $C_{11}H_{10}ClF_3NO^+$ 264.0398; Found 264.0401.

(*E*)-5-bromo-*N*-methyl-2-(3,3,3-trifluoroprop-1-en-1-yl)benzamide (5q)

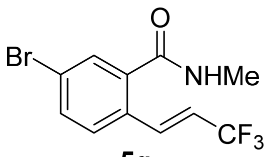

5q

The crude product was purified by flash column chromatography on silica gel to afford **5q** (19.3 mg, 63% yield) as a white solid.

MP: 138 – 139 °C

^{1}H NMR (400 MHz, $CDCl_3$) δ 7.63 – 7.53 (m, 2H), 7.53 – 7.43 (m, 1H), 7.42 (d, J = 8.3 Hz, 1H), 6.15 (dq, J = 16.1, 6.4 Hz, 1H), 5.99 (s, 1H), 2.99 (d, J = 5.0 Hz, 3H).

^{13}C NMR (101 MHz, $CDCl_3$) δ 167.7, 138.1, 134.5 (q, J = 6.9 Hz), 133.6, 131.1, 130.7, 128.6, 123.8, 123.2 (q, J = 270.5 Hz), 118.7 (q, J = 34.2 Hz), 27.1.

^{19}F NMR (376 MHz, $CDCl_3$) δ – 63.60 (dd, J = 6.3, 2.5 Hz).

IR (neat) cm⁻¹ υ: 3268 (w), 2016 (w), 1675 (s), 1602 (s), 1576 (s), 1346 (s), 1302 (s), 1256 (s), 1119 (s), 985 (m), 904 (w), 901 (w), 798 (w).

HRMS (ESI) m/z: $[M + H]^+$ Calcd for $C_{11}H_{10}BrF_3NO^+$ 307.9892; Found 307.9892.

(*E*)-*N*-methyl-3-(3,3,3-trifluoroprop-1-en-1-yl)-2-naphthamide (5r)

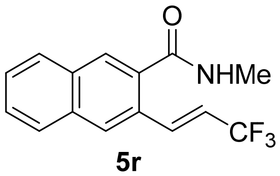

5r

The crude product was purified by flash column chromatography on silica gel to afford **5r** (20.8 mg, 75% yield) as a white solid.

MP: 141 – 142 °C

^{1}H NMR (400 MHz, $CDCl_3$) δ 7.97 (s, 1H), 7.92 (s, 1H), 7.88 – 7.76 (m, 2H), 7.69 (dq, J = 16.0, 2.3 Hz, 1H), 7.62 – 7.49 (m, 2H), 6.23 (dq, J = 16.0, 6.5 Hz, 1H), 6.08 (s, 1H), 3.05 (d, J = 4.9 Hz, 3H).

^{13}C NMR (101 MHz, $CDCl_3$) δ 169.4, 136.2 (q, J = 6.9 Hz), 133.8, 133.7, 132.9, 129.7, 128.3, 128.2, 128.1, 127.9, 127.6, 127.4, 123.4 (q, J = 269.3 Hz), 118.0 (q, J = 33.8 Hz), 27.1.

^{19}F NMR (376 MHz, $CDCl_3$) δ –63.28 (dd, J = 6.5, 2.3 Hz).

IR (neat) cm^{-1} υ: 3294 (w), 2158 (w), 1664 (w), 1624 (s), 1549 (m), 1308 (s), 1270 (m), 1123 (s), 1101 (s), 966 (m), 746 (s), 685 (m).

HRMS (ESIm/z: $[M + H]^+$ Calcd for $C_{15}H_{13}F_3NO^+$ 280.0944; Found 280.0948.

(*E*)-*N*-methyl-1-(3,3,3-trifluoroprop-1-en-1-yl)-2-naphthamide (5s)

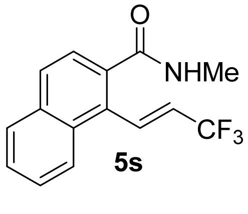

5s

The crude product was purified by flash column chromatography on silica gel to afford **5s** (17.7 mg, 64% yield) as a white solid.

MP: 157 – 158 °C

^{1}H NMR (400 MHz, $CDCl_3$) δ 8.05 – 7.96 (m, 1H), 7.91 – 7.83 (m, 2H), 7.76 (dq, J = 16.3, 2.3 Hz, 1H), 7.62 – 7.56 (m, 2H), 7.53 (d, J = 8.5 Hz, 1H), 6.13 (dq, J = 16.3, 6.4 Hz, 1H), 5.79 (s, 1H), 2.99 (d, J = 4.9 Hz, 3H).

^{13}C NMR (101 MHz, $CDCl_3$) δ 170.1, 134.1 (d, J = 7.0 Hz), 134.0, 133.9, 130.9, 129.6, 129.4, 128.7, 127.7, 127.4, 125.1, 124.3, 123.4 (q, J = 33.7 Hz), 122.9 (q, J = 271.1 Hz), 27.0

^{19}F NMR (376 MHz, $CDCl_3$) δ –64.24 (dd, J = 6.3, 2.4 Hz).

IR (neat) cm^{-1} υ: 3291 (w), 1667 (w), 1629 (m), 1545 (w), 1481 (w), 1410 (w), 1304 (m), 1261 (m), 1114 (s), 970 (w), 911 (w), 839 (w), 762 (m), 699 (s).

HRMS (ESI) m/z: $[M + H]^+$ Calcd for $C_{15}H_{13}F_3NO^+$ 280.0944; Found 280.0943.

(*E*)-N-ethyl-2-(3,3,3-trifluoroprop-1-en-1-yl)benzamide (5t)

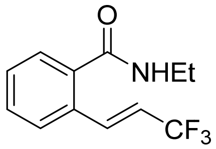

5t

The crude product was purified by flash column chromatography on silica gel to afford **5t** (15.1 mg, 62% yield) as a white solid.

MP: 86-87 °C

^{1}H NMR (400 MHz, $CDCl_3$) δ 7.62 – 7.54 (m, 2H), 7.52 – 7.36 (m, 3H), 6.17 (dq, J = 16.1, 6.4 Hz, 1H), 5.77 (s, 1H), 3.50 (qd, J = 7.3, 5.7 Hz, 2H), 1.25 (t, J = 7.3 Hz, 3H).

^{13}C NMR (101 MHz, $CDCl_3$) δ 168.5, 136.9, 135.6 (q, J = 6.8 Hz), 132.0, 130.6, 129.8, 127.6, 127.1, 123.4 (q, J = 269.2 Hz), 118.3 (q, J = 34.0 Hz), 35.2, 15.0.

^{19}F NMR (376 MHz, $CDCl_3$) δ -63.52 (dd, J = 6.4, 2.3 Hz).

IR (neat) cm^{-1} υ: 3298 (w), 1668 (w), 1630 (m), 1597 (w), 1540 (m), 1314 (m), 1270 (s), 1128 (m), 1105 (s), 971 (m), 877 (w), 751 (m), 695 (m).

HRMS (ESI) m/z: $[M + H]^+$ Calcd for $C_{12}H_{13}F_3NO^+$ 244.0944; Found 244.0943.

(*E*)-*N*-methyl-2-(4,4,4-trifluorobut-2-en-2-yl)benzamide (5u) & *N*-methyl-2-(4,4,4-trifluorobut-1-en-2-yl)benzamide (5u')

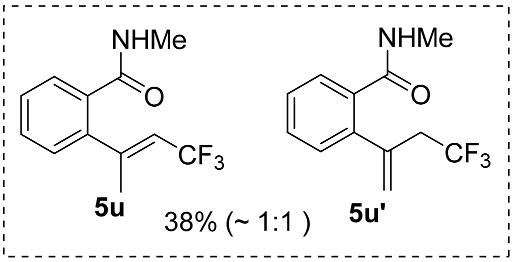

The crude product was purified by flash column chromatography on silica gel to afford **5u**, **5u'** (9.3 mg, 38% yield, **5u**:**5u'** ≈ 1:1) as a white solid.

^{1}H NMR (400 MHz, CDC13) δ 7.47 (dd, *J* = 7.5, 1.5 Hz, 1H), 7.44 (dd, *J* = 7.6, 1.5 Hz, 1H), 7.37 – 7.23 (m, 4H), 7.15 (dd, *J* = 7.5, 1.4 Hz, 1H), 7.13 (dd, *J* = 7.4, 1.6 Hz, 1H), 5.90 (brs, 1H), 5.85 (brs, 1H), 5.60 (qq, *J* = 16.0, 1.4 Hz, 1H), 5.40 (s, 1 H), 5.31 (s, 1H), 3.13 (qd, *J* = 10.8, 1.1 Hz, 2H), 2.87 (d, *J* = 4.8 Hz, 3H), 2.86 (d, *J* = 4.8 Hz, 3.6H), 2.13 (qd, *J* = 2.4, 1.4 Hz, 3H).

^{13}C NMR (101 MHz, $CDCl_3$) *δ* 169.8, 169.3, 150.9 (q, *J* = 5.7 Hz), 140.6, 139.3, 139.0 (q, *J* = 2.9 Hz), 134.66, 134.64, 130.4, 130.3, 129.5, 128.9, 128.6, 128.2, 128.14, 128.07, 127.9, 125.6 (q, *J* = 277.7 Hz), 123.1 (q, *J* = 271.3 Hz), 121.9, 118.0 (q, *J* = 33.9 Hz), 41.2 (q, *J* = 28.9 Hz), 26.8, 26.79, 19.7.

^{19}F NMR (376 MHz, $CDCl_3$) *δ* -58.12, -64.47.

IR (neat) cm^{-1} υ: 3240 (w), 1631 (m), 1564 (w), 1552 (m), 1412 (w), 1350 (w), 1317 (w), 1253 (s), 1131 (s), 1117 (s), 1080 (s), 931 (w), 846 (w), 757 (m).

HRMS (ESI) m/z: $[M + Na]^+$ Calcd for $C_{12}H_{12}F_3NNaO^+$ 266.0763; Found 266.0764.

Supplementary Notes

Supplementary Note 1: mechanism study

a) Synthesis and characterization data of 2-alkyl benzohdrazides 3a-D2 & 7.

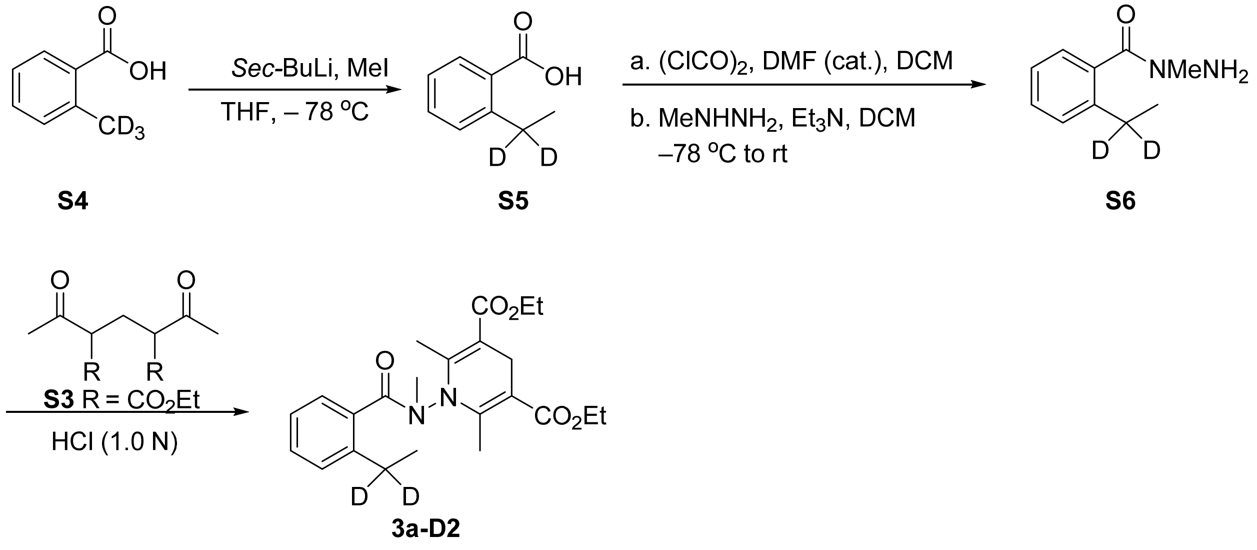

To a solution of **S4** (0.83g, 6.0 mmol, 1.0 euqiv) in THF (40 mL) was added *sec*-BuLi (8.6 mL, 1.4 M in cyclohexane, 2.0 equiv) at –78 °C. The reaction mixture was stirred at –78 °C for 1 h, then MeI (1.1 mL, 18.0 mmol, 3.0 equiv) was added to the above reaction mixture. The reaction mixture was stirred for 2 hours, and aqueous NH_4Cl solution was added into the mixture. The mixture was extracted with EtOAc. The organic extracts were washed with Na_2CO_3 solution and brine, dried over Na_2SO_4. The solvent was removed under reduced pressure. The residue was purified by a short column flash chromatography (SiO_2, eluent: EtOAc) to give **S5**, which can be used directly in the next step.

Following the Studer's procedure,[1] the **3a-D2** was prepared.

^{1}H NMR (400 MHz, Acetone-d_6) δ 7.49 – 7.42 (m, 1.5 H), 7.40 – 7.27 (m, 2H), 7.18 – 7.06 (m, 0.5 H), 4.19 (q, J = 7.1 Hz)/4.11 (qd, J = 7.1, 3.7 Hz)* (4 H), 3.47, 3.13 (ABq, J = 18.4 Hz)/ 3.19 (d, J = 17.0 Hz)*, 1.66 (dhept, J = 17.0, 1.7 Hz)* (2H), 3.22/3.49* (s, 3H), 2.40 (t, J = 1.2 Hz)/2.37 (d, J = 1.7 Hz)* (6H), 1.29 (t, J = 7.1 Hz)/1.21 (t, J = 7.1 Hz)* (6H), 1.25 (brs)/1.20 (brs) (3H). (Signals marked with an asterisk (*) correspond to the peaks assigned to the minor rotamer.).

^{13}C NMR (101 MHz, Acetone-d_6) (major rotamer) δ 170.1, 167.0, 148.1, 141.2, 134.4, 130.0, 129.4, 126.1, 126.0, 102.2, 59.6, 40.8, 24.9, 15.1, 13.9, 13.9.

^{13}**C NMR** (101 MHz, Acetone-d_6) (minor rotamer) δ 172.3, 166.3, 149.6, 141.6, 134.1, 129.3, 128.6, 125.4, 124.8, 103.2, 59.6, 40.0, 22.8, 14.9, 14.7, 13.8.

IR (neat) cm^{-1} υ: 2860 (w), 1698 (m), 1668 (s), 1445 (w), 1388 (m), 1295 (m), 1216 (s), 1199 (s), 1134 (m), 1103 (s), 1024 (s), 760 (m), 740 (s), 699 (m).

HRMS (ESI) m/z: $[M + H]^+$ Calcd for $C_{23}H_{29}D_2N_2O_5^+$ 417.2353; Found 417.2352.

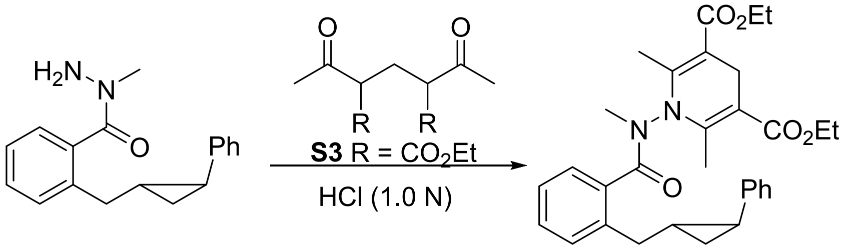

S7 **7**

Following the Studer's procedure,[1] compound **7** was prepared from compound **S7**.

diethyl 2,6-dimethyl-1-(*N*-methyl-2-((2-phenylcyclopropyl)methyl)benzamido)-1,4-dihydropyridine-3,5-dicarboxylate (7)

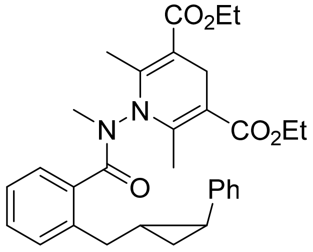

7

^{1}**H NMR** (400 MHz, Acetone-d_6) δ 7.59 (d, J = 7.7 Hz, 0.7H), 7.47 (q, J = 7.6 Hz, 1H), 7.37 (dt, J = 14.8, 7.4 Hz, 1.7H), 7.23 (t, J = 7.5 Hz, 1.6H), 7.14-7.06 (m, 3H), 4.18 (q, J = 7.1 Hz, 3H)/4.14 – 4.01 (m, 1H)*, 3.47, 3.12 (ABq, J = 18.4 Hz, 1.4H)/3.21*, 1.65* (d, J = 17.0 Hz, 0.6H), 3.21/3.49* (s, 3H), 2.92 – 2.80/2.78-2.60* (m, 2H), 2.41/2.37* (s, 3H), 2.39 (s, 3H), 1.96 – 1.87 (m, 1H), 1.49-1.35 (m, 1H), 1.28 (t, J = 7.2 Hz)/1.01 (t, J = 6.9 Hz)* (3H), 1.24 – 1.19 (m, 2H), 1.01 (t, J = 6.9 Hz, 3H). (Signals marked with an asterisk (*) correspond to the peaks assigned to the minor rotamer.).

^{13}C NMR (101 MHz, Acetone-d_6) (major rotamer) δ 170.1, 167.0, 148.0, 148.0, 143.2, 138.8, 134.5, 129.9, 128.2, 126.3, 126.0, 125.6, 125.2, 102.2, 59.6, 40.8, 36.9, 25.0, 23.8, 23.2, 15.6, 14.1, 14.0, 13.9.

^{13}C NMR (101 MHz, Acetone-d_6) (minor rotamer) δ 172.4, 166.3, 149.6, 149.6, 143.4, 139.2, 134.3, 129.2, 129.0, 128.2, 125.6, 125.5, 125.22, 125.17, 103.4, 59.64, 40.1, 36.9, 23.8, 23.4, 22.9, 15.7, 14.8, 14.8, 13.8, 13.8.

IR (neat) cm^{-1} υ: 2980 (w), 1704 (m), 1667 (s), 1601 (w), 1443 (w), 1387 (m), 1365 (w), 1291 (m), 1280 (m), 1213 (s), 1112 (s), 1049 (s), 1001 (m), 972 (w), 886 (m), 875 (w), 770 (m), 750 (s), 721 (m).

HRMS (ESI) m/z: $[M + H]^+$ Calcd for $C_{31}H_{37}N_2O_5^+$ 517.2697; Found 517.2697.

b) Control experiments of γ-azidation of 2-alkyl benzohydrazides

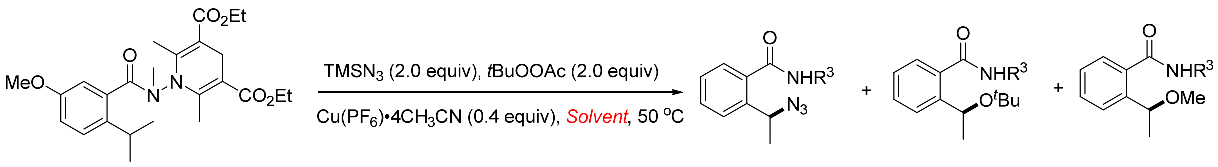

Condition A: A screw cap tube was charged with $Cu(PF_6)•4CH_3CN$ (14.9 mg, 0.04 mmol, 0.4 equiv), substrate **3k** (0.1 mmol, 1.0 equiv), $TMSN_3$ (26.5 µl, 0.2 mmol, 2.0 equiv) and *t*BuOH (2.0 mL). The mixture was stirred at 50 °C for 2 minutes, then *t*BuOOAc (32.2 µL, 0.2 mmol, 2.0 equiv) was added to the above mixture. After being stirred for 2 hours at 50 °C under N_2 atmosphere, the reaction mixture was quenched with water, extracted with EtOAc. The organic extracts were washed with brine, dried over Na_2SO_4. The solvent was removed under reduced pressure. The residue was purified by flash chromatography (SiO_2, eluent: PE/EtOAc = 2/1) to give **4k** (16.8 mg, 72% yield) as an oil and **4k'** (5.6 mg, 21% yield) as an oil.

Condition B: A screw cap tube was charged with $Cu(PF_6)•4CH_3CN$ (14.9 mg, 0.04 mmol, 0.4 equiv), substrate **3k** (0.1 mmol, 1.0 equiv), $TMSN_3$ (26.5 µl, 0.2 mmol, 2.0 equiv) and *t*BuOH (1.0 mL)/MeOH (1.0 mL) . The mixture was stirred at 50 °C for 2 minutes, then *t*BuOOAc (32.2 µL, 0.2 mmol, 2.0 equiv) was added to the above mixture. After being stirred for 2 hours at 50 °C under N_2 atmosphere, the

reaction mixture was quenched with water, extracted with EtOAc. The organic extracts were washed with brine, dried over Na_2SO_4. The solvent was removed under reduced pressure. The residue was purified by flash chromatography (SiO_2, eluent: PE/EtOAc = 2/1) to give **4k** (15.2 mg, 65% yield) as an oil and **4k'** (1.7 mg, 6% yield) as an oil.

c) Radical clock experiment of γ-azidation of 2-alkyl benzohydrazides.

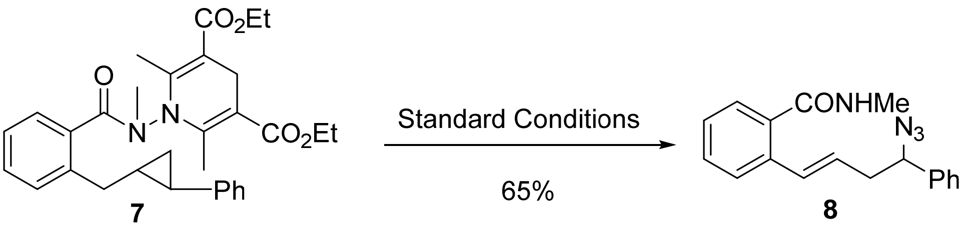

A screw cap tube was charged with $Cu(PF_6)$•$4CH_3CN$ (14.9 mg, 0.04 mmol, 0.4 equiv), substrate **7** (0.1 mmol, 1.0 equiv), $TMSN_3$ (26.5 μ l, 0.2 mmol, 2.0 equiv) and *t*BuOH (2.0 mL). The mixture was stirred at 50 °C for 2 minutes, then *t*BuOOAc (32.2 uL, 0.2 mmol, 2.0 equiv) was added to the above mixture. After being stirred for 2 hours at 50 °C under N_2 atmosphere, the reaction mixture was quenched with water, extracted with EtOAc. The organic extracts were washed with brine, dried over Na_2SO_4. The solvent was removed under reduced pressure. The residue was purified by flash chromatography (SiO_2, eluent: PE/EtOAc = 2/1) to give **8** (19.8 mg, 65% yield)**.**

(*E*)-2-(4-azido-4-phenylbut-1-en-1-yl)-N-methylbenzamide (8)

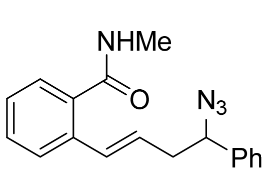

^{1}H NMR (400 MHz, $CDCl_3$) δ 7.48 – 7.23 (m, 9H), 6.80 (d, J = 15.8 Hz, 1H), 6.10 (dt, J = 15.2, 7.2 Hz, 1H), 5.84 (s, 1H), 4.62 (t, J = 7.1 Hz, 1H), 2.96 (d, J = 4.9 Hz, 3H), 2.80-2.67 (m, 2H).

^{13}C NMR (101 MHz, $CDCl_3$) δ 169.9, 139.1, 135.2, 135.0, 131.0, 130.1, 128.8, 128.4, 128.2, 127.6, 127.4, 126.9, 126.5, 65.9, 40.1, 26.8.

IR (neat) cm^{-1} υ: 3061 (w), 2095 (s), 2022 (w), 1635 (m), 1535 (m), 1475 (w), 1409 (w), 1309 (m), 1240 (m), 1159 (w), 1035 (w), 966 (m), 752 (s), 698 (s).

HRMS (ESI) m/z: $[M + Na]^+$ Calcd for $C_{18}H_{18}N_4NaO^+$ 329.1373; Found 329.1374.

d) Kinetic isotope effect of γ-azidation of 2-alkyl benzohydrazides.

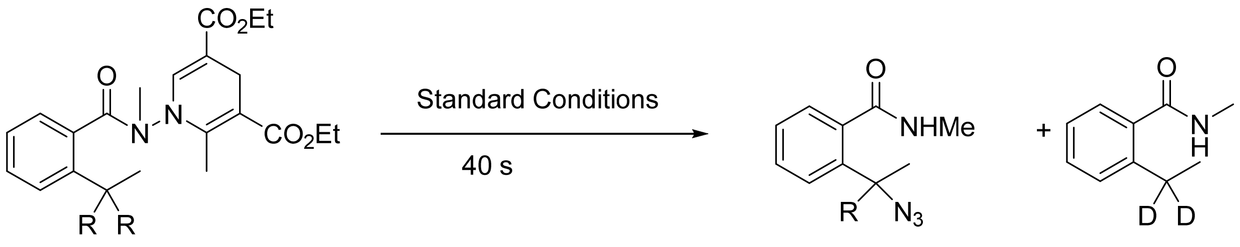

Two sets of reactions were carried out in parallel under the standard conditions, and quenched with aqueous Na_2CO_3 in 40 s. The **4a** and **4a-D1** were isolated in 66% and 25% yield, respectively. Note: **4a-D1** was not pure, which was contaminated with amide **4a-D2**.

2-(1-azidoethyl-1-d)-*N*-methylbenzamide (4a-D1)

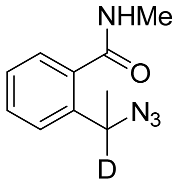

^{1}H NMR (400 MHz, $CDCl_3$) δ 7.54 (dd, J = 7.9, 1.4 Hz, 1H), 7.49 (td, J = 7.5, 1.5 Hz, 1H), 7.40 (dd, J = 7.7, 1.5 Hz, 1H), 7.33 (td, J = 7.4, 1.4 Hz, 1H), 6.10 (s, 1H), 3.00 (d, J = 4.9 Hz, 3H), 1.57 (s, 3H).

^{13}C NMR (101 MHz, $CDCl_3$) δ 169.8, 139.5, 135.4, 130.6, 127.9, 127.0, 126.7, 26.8, 21.4. *Signals corresponding to the deuterium substituted carbon was not resolvable due to its anticipated weak intensity.*

HRMS (ESI/QTOF) m/z: $[M + Na]^+$ Calcd for $C_{10}H_{11}[_2H]N_4NaO^+$ 228.0966; Found 228.0965.

Amide 4-D2 ^{1}H NMR (400 MHz, $CDCl_3$) δ 7.32 – 7.30 (m, 2H), 7.27 (dd, J = 7.8, 1.3 Hz, 1H), 7.20 (td, J = 7.4, 1.4 Hz, 1H), 5.84 (s, 1H), 2.99 (d, J = 6.4 Hz, 3H), 1.22 (s, 3H).

Amide 4-D2 ^{13}C NMR (101 MHz, $CDCl_3$) δ 129.9, 129.4, 126.6, 125.7, 26.6, 15.7. *Signals corresponding to the deuterium substituted carbon was not resolvable due to its anticipated weak intensity*

IR (neat) cm^{-1} υ: 3272 (w), 2938 (w), 2089 (m), 1633 (m), 1543 (m), 1409 (w), 1310 (w), 1138 (w), 803 (w), 743 (w), 720 (s), 674 (w).

e) Control experiments of oxidative δ-trifluoromethylation of 2-alkyl benzohydrazides

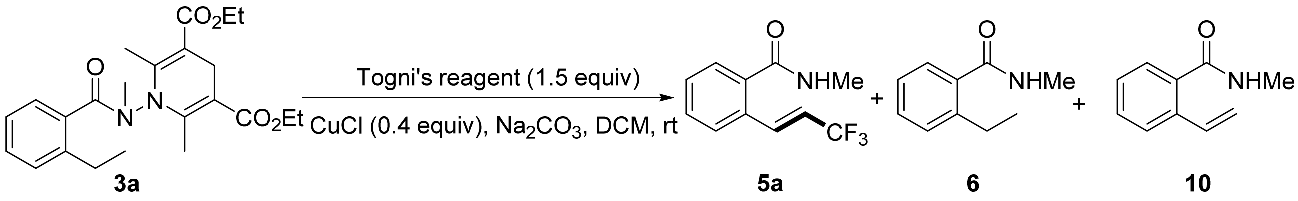

To a screw cap tube charged with CuCl (4.0 mg, 0.04 mmol) and Na_2CO_3 (31.8 mg, 0.3 mmol, 3.0 equiv) was added a solution of Togni's reagent (47.4 mg, 0.15 mmol, 1.5 equiv) in DCM (0.5 mL) under argon atmosphere. After stirring for 2 min, substrate **3a** (0.1 mmol, 1.0 equiv) in DCM (0.5 mL) was added to the above mixture. After being stirred for 4 hours at room temperature under argon atmosphere, the reaction mixture was quenched with water, extracted with EtOAc. The organic extracts were washed with Na_2CO_3 solution and brine, dried over Na_2SO_4. The solvent was removed under reduced pressure. The residue was purified by flash chromatography (SiO_2, eluent: PE/EtOAc = 2/1) to give **5a** (9.5 mg, 42% yield), **10** (1.9 mg, 12% yield), **6** (0.7 mg, 4% yield).

2-ethyl-*N*-methylbenzamide (6)

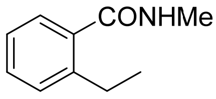

6

The physical and spectroscopic data were in accordance with those reported in the literature.[3]

^{1}H NMR (400 MHz, $CDCl_3$) δ 7.24 (td, *J* = 7.5, 1.5 Hz, 1H), 7.19 (dd, *J* = 7.3, 1.2 Hz, 1H), 7.16 (d, *J* = 7.8 Hz, 1H), 7.08 (td, *J* = 7.5, 1.3 Hz, 1H), 5.95 (s, 1H), 2.85 (d, *J* = 4.9 Hz, 3H), 2.68 (q, *J* = 7.6 Hz, 2H), 1.13 (t, *J* = 7.6 Hz, 3H).

^{13}C NMR (101 MHz, $CDCl_3$) δ 171.0, 142.2, 136.3, 129.8, 129.3, 126.7, 125.6, 26.6, 26.3, 15.8.

***N*-methyl-2-vinylbenzamide (10)**

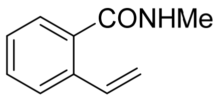

10

The physical and spectroscopic data were in accordance with those reported in the literature.[2]

^{1}H NMR (400 MHz, $CDCl_3$) δ 7.55 (dd, *J* = 7.8, 1.1 Hz, 1H), 7.46 – 7.34 (m, 2H), 7.30 – 7.22 (m, 1H), 7.02 (dd, *J* = 17.5, 10.9 Hz, 1H), 5.88 (s, 1H), 5.70 (dd, *J* = 17.5, 1.1 Hz, 1H), 5.33 (dd, *J* = 10.9, 1.1 Hz, 1H), 2.97 (d, *J* = 4.9 Hz, 3H).

^{13}C NMR (101 MHz, $CDCl_3$) δ 170.1, 135.8, 135.4, 134.5, 130.1, 127.7, 127.4, 126.2, 116.7, 26.8.

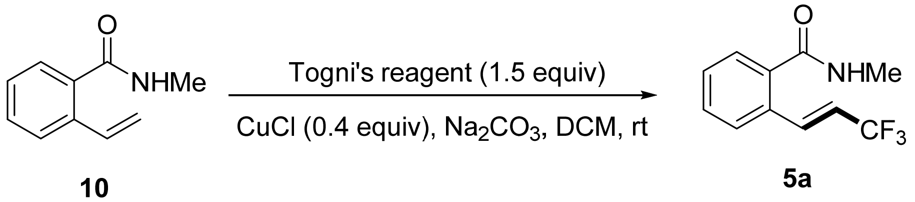

To a screw cap tube charged with CuCl (4.0 mg, 0.04 mmol) and Na_2CO_3 (31.8 mg, 0.3 mmol, 3.0 equiv) was added a solution of Togni's reagent (63.2 mg, 0.2 mmol, 2.0 equiv) in DCM (0.5 mL) under argon atmosphere. After stirring for 2 min, compound **10** (16.1 mg, 0.1 mmol, 1.0 equiv) in DCM (0.5 mL) was added to the above mixture. After being stirred for 4 hours at room temperature under argon atmosphere, the reaction mixture was quenched with water, extracted with EtOAc. The organic extracts were washed with Na_2CO_3 solution and brine, dried over Na_2SO_4. The solvent was removed under reduced pressure. The residue was purified by flash chromatography (SiO_2, eluent: PE/EtOAc = 2/1) to give **5a** (20.4 mg, 89% yield).

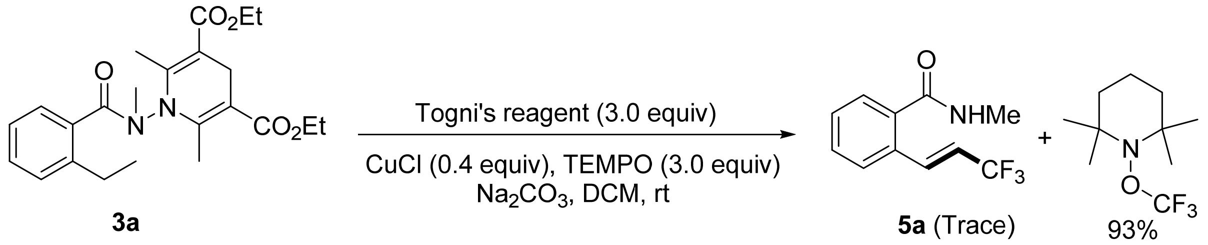

To a screw cap tube charged with CuCl (4.0 mg, 0.04 mmol) and Na_2CO_3 (31.8 mg, 0.3 mmol, 3.0 equiv) was added a solution of Togni's reagent (94.8 mg, 0.3 mmol, 3.0 equiv) in DCM (0.5 mL) under argon atmosphere. After stirring for 2 min, compound **3a** (41.4 mg, 0.1 mmol, 1.0 equiv) and TEMPO (46.8 mg, 0.3 mmol, 3.0 equiv) in DCM (0.5 mL) was added to the above mixture. After being stirred for 4 hours at room temperature under argon atmosphere, the reaction mixture was quenched with water, extracted with EtOAc. The organic extracts were washed with Na_2CO_3 solution and brine, dried over Na_2SO_4. The solvent was removed under reduced pressure. The yield of **TEMPO-CF₃**[4] was determined by ^{19}F NMR analysis (PhF was internal reference) as 94% yield.

d) KIE Experiments of oxidative δ-trifluoromethylation of 2-ethyl benzohdrazides

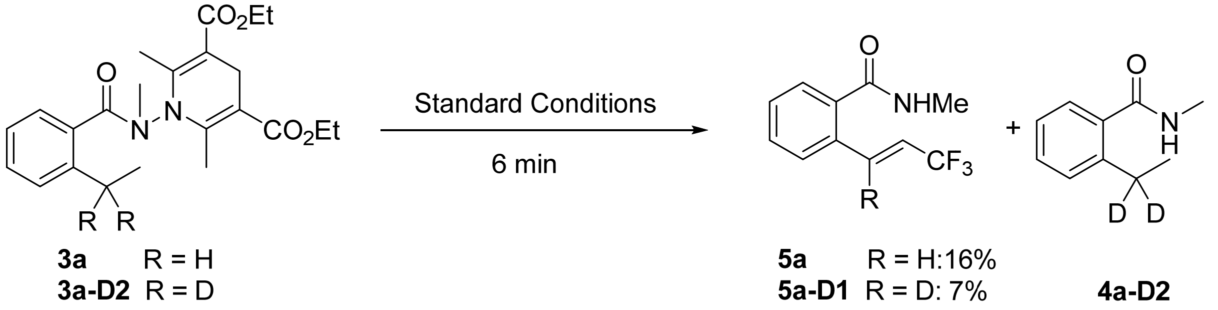

Two sets of reactions were carried out in parallel under the standard conditions, and quenched with aqueous Na_2CO_3 in 6 min. **5a** and **5a-D1** were isolated in 16% and 7% yield, respectively. Note: **5a-D1** was not pure, which was contaminated with amide **4a-D2**.

(*E*)-*N*-methyl-2-(3,3,3-trifluoroprop-1-en-1-yl-1-d)benzamide (5a-D1)

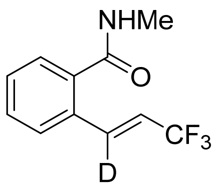

^{1}H NMR (400 MHz, $CDCl_3$) δ 7.49 (dd, J = 8.0, 1.4 Hz, 1H), 7.41 – 7.36 (m, 2H), 7.34 – 7.29 (m, 1H), 6.08 (qt, J = 6.4, 2.2 Hz, 1H), 5.89 (s, 1H), 2.92 (d, J = 4.9 Hz, 3H).

^{13}C NMR (101 MHz, $CDCl_3$) δ 169.2, 136.5, 135.2 (m), 131.9, 130.4, 129.7, 127.4, 127.0, 123.3 (q, J = 269.2 Hz), 117.9 (q, J = 34.0 Hz), 26.9.

IR (neat) cm^{-1} υ: 3293 (w), 1667 (w), 1543 (w), 1471 (w), 1410 (w), 1302 (m), 1261 (m), 1115 (s), 970 (w), 915 (w), 836 (w), 761 (m), 699 (s).

HRMS (ESI) m/z: $[M + H]^+$ Calcd for $C_{11}H_{10}[_2H]F_3NO^+$ 231.0850; Found 231.0851.

c) Isoindolinone (11) formation

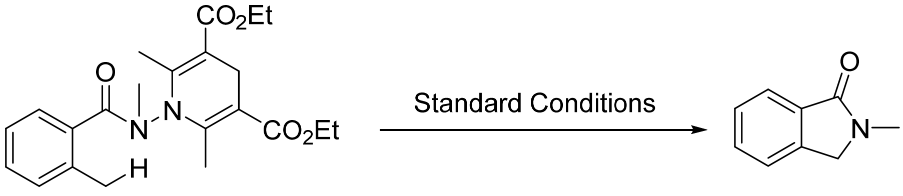

To a screw cap tube charged with CuCl (4.0 mg, 0.04 mmol) and Na_2CO_3 (31.8 mg, 0.3 mmol, 3.0 equiv) was added a solution of Togni's reagent (94.8 mg, 0.3 mmol, 3.0 equiv) in DCM (0.5 mL) under argon atmosphere. After stirring for 2 min, compound **3r** (40.0 mg, 0.1 mmol, 1.0 equiv) in DCM (0.5 mL) was added to the above mixture. After being stirred for 4 hours at room temperature under argon atmosphere, the reaction mixture was quenched with water, extracted with EtOAc. The organic extracts were washed with Na_2CO_3 solution and brine, dried over Na_2SO_4. The solvent was removed under reduced pressure. The residue was purified by flash chromatography (SiO_2, eluent: PE/EtOAc = 2/1) to give **11** (5.3 mg, 36% yield). The physical and spectroscopic data were in accordance with those reported in the literature.[5]

^{1}**H NMR** (400 MHz, CDCl3): 3.22 (s, 3H), 4.38 (s, 2H), 7.47 (t, J = 8.1 Hz, 2H), 7.54 (t, J = 7.4 Hz, 1H), 7.82 (d, J = 7.5 Hz, 1H).

Supplementary Note 2: transformation of amides 4a

a)

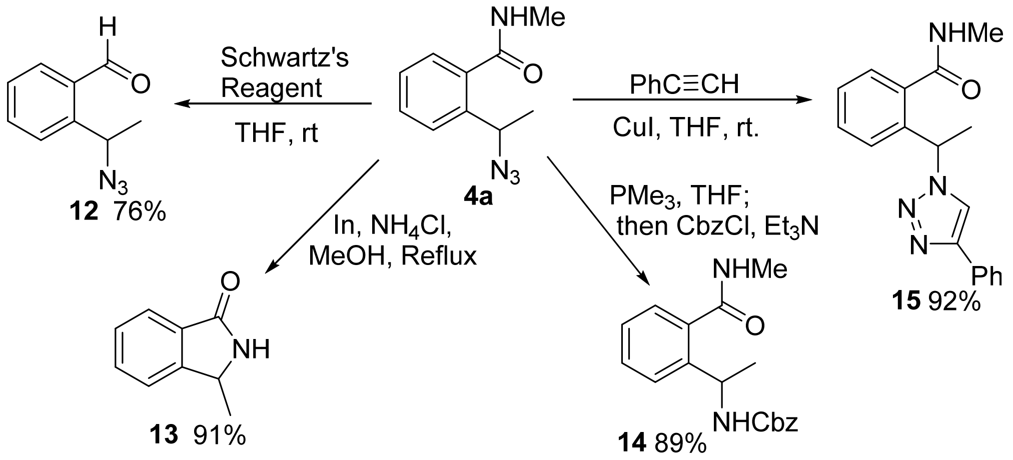

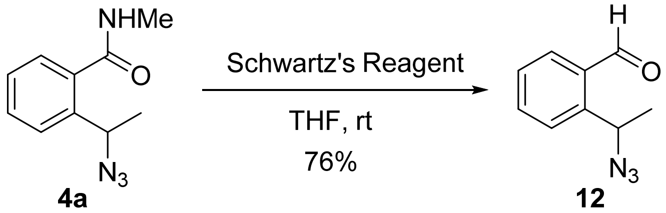

To a solution of **4a** (20.4 mg, 0.1 mmol, 1.0 euqiv) in THF (2.0 mL) was added Schwartz's reagent (35.0 mg, 0.12 mmol, 1.2 equiv) at room temperature. The reaction mixture was stirred at room temperature overnight, then the solvent was removed under reduced pressure. The residue was purified by flash chromatography (SiO_2, eluent: PE/EtOAc = 3/1) to give **12** (13.2 mg, 76% yield) as an oil**.**

2-(1-azidoethyl)benzaldehyde (12)

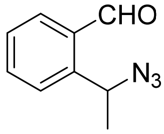

^{1}**H NMR** (400 MHz, $CDCl_3$) δ 10.16 (s, 1H), 7.85 – 7.79 (m, 1H), 7.70 – 7.61 (m, 2H), 7.52 (ddd, J = 7.6, 6.7, 2.1 Hz, 1H), 5.79 (q, J = 6.7 Hz, 1H), 1.53 (d, J = 6.7 Hz, 3H).

^{13}**C NMR** (101 MHz, $CDCl_3$) δ 193.1, 143.5, 134.8, 134.5, 132.7, 128.3, 127.1, 56.6, 22.2.

IR (neat) cm^{-1} υ: 3220 (w), 1688 (s), 1616 (w), 1469 (w), 1416 (w), 1356 (w), 1308 (w), 1142 (w), 1099 (m), 798 (w), 761 (m), 716 (m), 703 (s).

HRMS (ESI) m/z: $[M + H]^+$ Calcd for $C_9H_{10}N_3O^+$ 176.0818; Found 176.0816

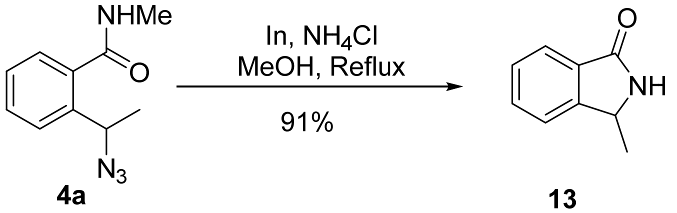

4a **13**

To a solution of **4a** (20.4 mg, 0.1 mmol, 1.0 euqiv) and NH_4Cl (32.1 mg, 0.6 mmol, 6.0 equiv) in MeOH (3.0 mL) was added In (68.9 mg, 0.6 mmol, 6.0 equiv) at room temperature. The reaction mixture was stirred under reflux for 24 hours, then quenched with water, extracted with EtOAc. The organic extracts were washed with Na_2CO_3 solution and brine, dried over Na_2SO_4. The solvent was removed under reduced pressure. The residue was purified by flash chromatography (SiO_2, eluent: PE/EtOAc = 2/1) to give **13** (13.5 mg, 91% yield), the physical and spectroscopic data were in accordance with those reported in the literature[6].

3-methylisoindolin-1-one (13)

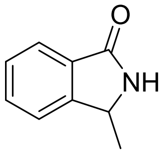

^{1}**H NMR** (400 MHz, $CDCl_3$) δ 8.11 (s, 1H), 7.84 (d, J = 7.5 Hz, 1H), 7.55 (td, J = 7.5, 1.2 Hz, 1H), 7.49 – 7.39 (m, 2H), 4.70 (q, J = 6.7 Hz, 1H), 1.50 (d, J = 6.8 Hz, 3H).

^{13}**C NMR** (101 MHz, $CDCl_3$) δ 171.3, 149.1, 131.91, 131.87, 128.1, 123.7, 122.3, 52.8, 20.4.

HRMS (ESI) m/z: $[M + H]^+$ Calcd for $C_9H_{10}NO^+$ 148.0757; Found 148.0754.

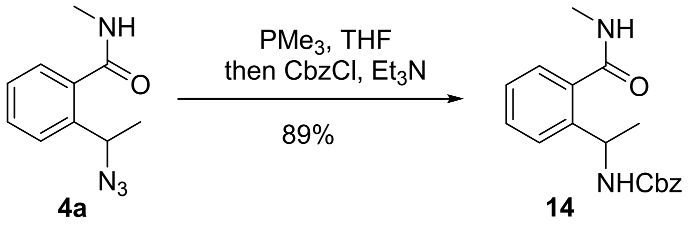

To a solution of **4a** (20.4 mg, 0.1 mmol, 1.0 euqiv) in THF (2.0 mL) was added PMe_3 (1.0 M in THF, 2.0 mL, 2.0 equiv) at room temperature. The reaction mixture was stirred at room temperature overnight. Et_3N (30 μL, 0.3 mmol) and CbzCl (29.0 uL, 0.2 mmol, 2.0 equiv) were added to the above mixture at 0 °C. After stirring for 1 h, the reaction mixture was quenched with water, extracted with EtOAc. The organic extracts were washed with Na_2CO_3 solution and brine, dried over Na_2SO_4. The solvent was removed under reduced pressure. The residue was purified by flash chromatography (SiO_2, eluent: PE/EtOAc = 3/1) to give **14** (27.7 mg, 89% yield) as colorless oil.

benzyl (1-(2-(methylcarbamoyl)phenyl)ethyl)carbamate (14)

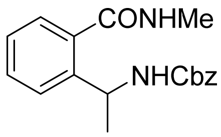

^{1}H NMR (400 MHz, $CDCl_3$) δ 7.85 – 6.98 (m, 9H), 5.90 (d, J = 6.9 Hz, 1H), 5.28 – 4.88 (m, 3H), 2.99 (d, J = 4.7 Hz, 3H), 1.44 (d, J = 6.9 Hz, 3H).

^{13}C NMR (101 MHz, $CDCl_3$) δ 170.8, 156.2, 141.4, 136.3, 135.9, 130.4, 128.6, 128.3, 128.15, 128.12, 127.5, 126.7, 66.9, 48.9, 26.8, 23.1.

IR (neat) cm^{-1} υ: 3278 (w), 2968 (w), 2160 (w), 1698 (s), 1638 (s), 1530 (s), 1455 (m), 1313 (m), 1250 (s), 1049 (s), 1005 (m), 755 (s), 731 (s), 697 (s).

HRMS (ESI) m/z: $[M + H]^+$ Calcd for $C_{18}H_{21}N_2O_3^+$ 313.1547; Found 313.1544.

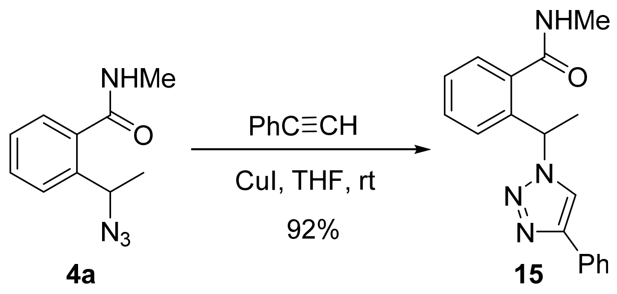

To a solution of **4a** (20.4 mg, 0.1 mmol, 1.0 euqiv) and CuI (3.8 mg, 0.02 mmol, 0.02 equiv) in THF (1.0 mL) was added ethynylbenzene (33 uL mg, 0.3 mmol, 3.0 equiv) at room temperature. The reaction mixture was stirred at room temperature for 24 hours, then quenched with water, and extracted with EtOAc. The organic extracts were washed with Na_2CO_3 solution and brine, dried over Na_2SO_4. The solvent was removed under reduced pressure. The residue was purified by flash chromatography (SiO_2, eluent: PE/EtOAc = 1/1) to give **15** (28.1 mg, 92% yield).

***N*-methyl-2-(1-(4-phenyl-1H-1,2,3-triazol-1-yl)ethyl)benzamide (15)**

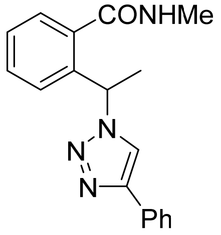

^{1}H NMR (400 MHz, $CDCl_3$) δ 7.96 (s, 1H), 7.74 (d, J = 6.9 Hz, 2H), 7.40 – 7.30 (m, 5H), 7.29 – 7.18 (m, 2H), 6.73 (d, J = 5.0 Hz, 1H), 6.34 (q, J = 7.0 Hz, 1H), 2.93 (d, J = 4.8 Hz, 3H), 1.98 (d, J = 7.0 Hz, 3H).

^{13}C NMR (101 MHz, $CDCl_3$) δ 169.9, 147.3, 138.9, 135.3, 130.8, 130.6, 128.8, 128.3, 128.1, 127.3, 126.8, 125.6, 119.8, 56.3, 26.8, 21.2.

IR (neat) cm^{-1} υ: 3291 (w), 2938 (w), 1639 (m), 1542 (m), 1484 (w), 1410 (w), 1307 (w), 1225 (w), 1157 (w), 1077 (w), 1030 (w), 911 (w), 763 (s), 729 (s), 720 (s), 694 (s).

HRMS (ESI) m/z: $[M + H]^+$ Calcd for $C_{18}H_{19}N_4O^+$ 307.1553; Found 307.1545.

b)

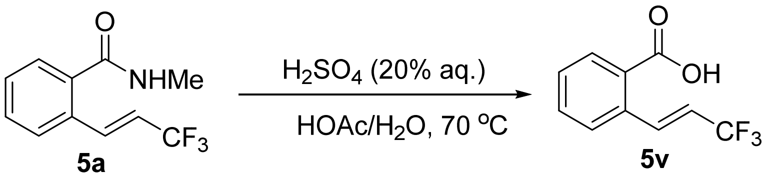

To a solution of **5a** (22.9 mg, 0.01 mmol, 1.0 euqiv) in HOAc (1.0 mL) and water (1.0 mL) was added H_2SO_4 (0.5 mL, 20% aq.) at room temperature. The reaction mixture was stirred at 70 °C for 24 h. The mixture was extracted with EtOAc. The organic extracts were washed with brine, dried over Na_2SO_4. The solvent was removed under reduced pressure. The residue was purified by a short column flash chromatography (SiO_2, eluent: EtOAc/PE = 1:1) to give **5v** (15.2 mg, 71% yield) as a white solid.

MP: 103 – 104 °C

^{1}H NMR (400 MHz, $CDCl_3$) δ 12.20 (s, 1H), 8.17 (dd, J = 7.9, 1.4 Hz, 1H), 8.12 (dq, J = 16.0, 2.3 Hz, 1H), 7.66-7.61 (m, 1H), 7.57 (dd, J = 7.9, 1.4 Hz, 1H), 7.51 (td, J = 7.6, 1.5 Hz, 1H), 6.09 (dq, J = 16.0, 6.4 Hz, 1H).

^{13}C NMR (101 MHz, $CDCl_3$) δ 172.5, 137.5 (q, J = 7.0 Hz), 136.7, 133.8, 132.0, 129.6, 128.4, 128.0, 123.3 (q, J = 269.4 Hz), 118.9 (q, J = 33.8 Hz).

^{19}F NMR (376 MHz, $CDCl_3$) δ –63.65 (dd, J = 6.3, 2.5 Hz).

IR (neat) cm^{-1} υ: 3247 (w), 2164 (w), 1687 (s), 1572 (w), 1489 (w), 1415 (m), 1308 (m), 1267 (s), 1107 (s), 1080 (s), 966 (m), 928 (m), 749 (s).

HRMS (ESI) m/z: $[M – H]^–$ Calcd for $C_{10}H_6F_3O_2^–$ 215.0325; Found 215.0324.

Supplementary Note 3: crystallographic data for 5a

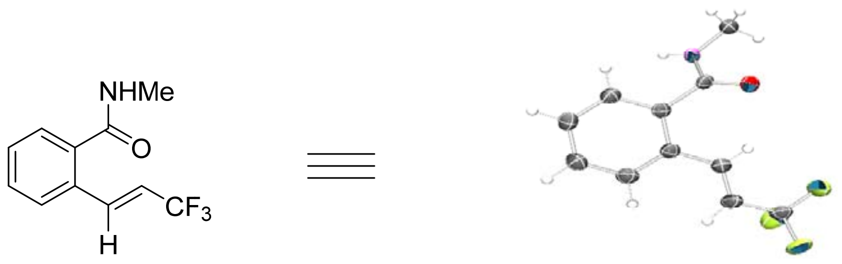

***Table S7* Crystal data and structure refinement for 5a**

Identification code	baox1188e-a	
Empirical formula	$C_{11}H_{10}F_3NO$	
Formula weight	229.20	
Temperature	140.00(10) K	
Wavelength	1.54184 Å	
Crystal system	Monoclinic	
Space group	$P2_1/c$	
Unit cell dimensions	a = 14.2553(4) Å	α = 90°.
	b = 4.95361(10) Å	β = 110.625(3)°.
	c = 16.0604(4) Å	γ = 90°.
Volume	1061.42(5) $Å^3$	
Z	4	
Density (calculated)	1.434 Mg/m^3	
Absorption coefficient	1.118 mm^{-1}	
F(000)	472	
Crystal size	0.435 x 0.223 x 0.189 mm^3	
Theta range for data collection	5.648 to 75.096°.	
Index ranges	$-17 \leq h \leq 17$, $-5 \leq k \leq 3$, $-19 \leq l \leq 19$	
Reflections collected	6516	

Independent reflections	2123 [R_{int} = 0.0169]
Completeness to θ = 67.684°	99.9 %
Absorption correction	Gaussian
Max. and min. transmission	1.000 and 0.458
Refinement method	Full-matrix least-squares on F^2
Data / restraints / parameters	2123 / 0 / 186
Goodness-of-fit on F^2	1.051
Final *R* indices [$I > 2\sigma(I)$]	R_1 = 0.0347, wR_2 = 0.0942
R indices (all data)	R_1 = 0.0370, wR_2 = 0.0966
Extinction coefficient	0.0036(5)
Largest diff. peak and hole	0.331 and -0.273 e.Å^{-3}

CCDC No.: CCDC 1866223

Supplementary References:

1. Chou, C.-M.; Guin, J.; Mück-Lichtenfeld, C.; Grimme, S.; Studer, A. Radical-Transfer Hydroamination of Olefins with N-Aminated Dihydropyridines. *Chem. Asian J.* **2011***, 6,* 1197.

2. Talbot, E. P. A.; Fernandes, T. de A.; McKenna, J. M.; Toste, F. D. Asymmetric Palladium-Catalyzed Directed Intermolecular Fluoroarylation of Styrenes. *J. Am. Chem. Soc.* **2014**, *136*, 4101.

3. Havlik, S. E.; Simmons, J. M.; Winton, V. J.; Johnson, J. B. Nickel-Mediated Decarbonylative Cross-Coupling of Phthalimides with in Situ Generated Diorganozinc Reagents. *J. Org. Chem.* **2011**, *76*, 3588.

4. Zhu, Z.-Z.; Chen, K.; Yu, L.-Z.; Tang, X.-Y.; Shi, M. Copper(I)-Catalyzed Intramolecular Trifluoromethylation of Methylenecyclopropanes. *Org. Lett.* **2015**, *17*, 5994.

5. Dang, T. T.; Zhu, Y.; Ngiam, J. S. Y.; Ghosh, S. C.; Chen, A.; Seayad, A. M. Palladium Nanoparticles Supported on ZIF-8 As an Efficient Heterogeneous Catalyst for Aminocarbonylation. *ACS Catal.* **2013**, *3*, 1406.

6. Ruan, Y. P.; Chen, M. D.; He, M. Z.; Zhou, X.; Huang, P. Q. A Practical Two-Step Synthesis of 3-Alkyl-2,3-dihydro-1H-isoindolin-1-ones. *Synth. Commun.* **2004**, *34*, 853.
